# Difluoromethylborates and Muonium for the Study of Isonitrile Insertion Affording Phenanthridines via Imidoyl Radicals

**DOI:** 10.1021/acs.joc.3c00056

**Published:** 2023-06-23

**Authors:** Kakeru Konagaya, Yu-En Huang, Kazuki Iwami, Tetsuya Fujino, Rikutaro Abe, Reuben Parchment-Morrison, Kenji M. Kojima, Iain McKenzie, Shigekazu Ito

**Affiliations:** † Department of Applied Chemistry, School of Materials and Chemical Technology, Tokyo Institute of Technology, 2-12-1-H-113 Ookayama, Meguro-ku, Tokyo 152-8552, Japan; ‡ School of Physics and Astronomy, Cardiff University, Queen’s Building, The Parade, Cardiff CF24 3AA, U.K.; § Centre for Molecular and Materials Science, TRIUMF, 4004 Wesbrook Mall, Vancouver, British Columbia V6T 2A3, Canada; ∥ Stewart Blusson Quantum Matter Institute, 2355 East Mall, Vancouver, British Columbia V6T 1Z4, Canada; ⊥ Department of Chemistry, Simon Fraser University, 8888 University Drive, Burnaby, British Columbia V5A 1S6, Canada; # Department of Physics and Astronomy, University of Waterloo, 200 University Avenue West, Waterloo, Ontario N2L 3G1, Canada

## Abstract

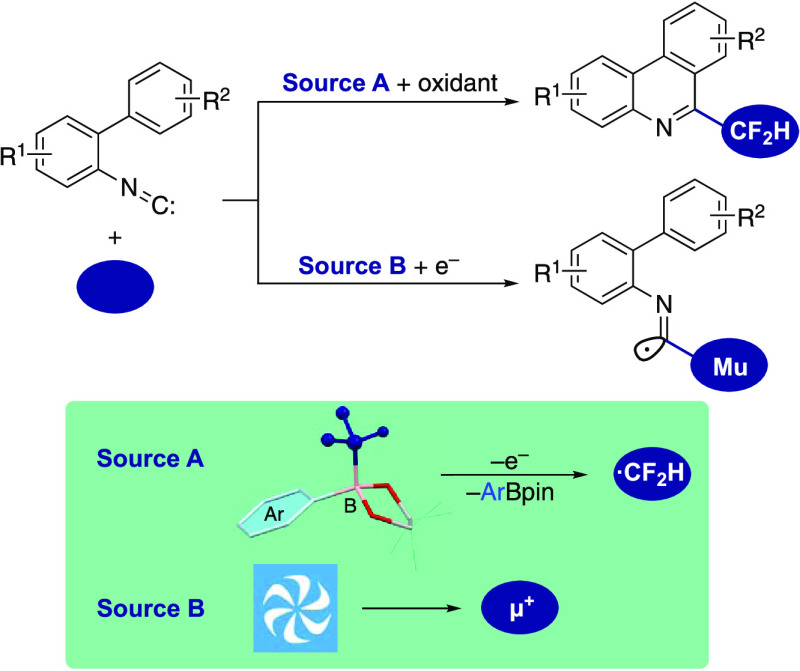

The 6-(difluoromethyl)phenanthridine unit is a highly attractive fluoroalkyl-substituted planar nitrogen heterocycle in pharmaceutical and agrochemical research. In this paper, we report that difluoromethylborates can be used as a source of difluoromethyl radicals for isonitrile insertion, leading to 6-(difluoromethyl)phenanthridines. Tuning the aryl substituents in the difluoromethylborates and oxidizing reagents enabled the synthesis of 6-(difluoromethyl)phenanthridines through the generation of difluoromethyl radical and spontaneous intramolecular cyclization of the CF_2_H-imidoyl radical intermediates. The presence of difluoromethyl radicals was experimentally confirmed, and the reaction mechanisms including imidoyl radical and prompt cyclization reactions could be supported theoretically. Furthermore, we obtained valuable information about the imidoyl radical intermediate by performing transverse-field muon spin rotation (TF-μSR) measurements of 2-isocyano-4′-methoxy-1,1′-biphenyl and using density functional theory (DFT) calculations to interpret the spectra. Muonium, a simple free radical, preferentially adds to the carbon atom of the isonitrile unit, yielding the corresponding imidoyl radical. The temperature dependence of the muon hyperfine coupling constant and the spin relaxation of the muoniated radical signal are compatible with the intramolecular cyclization of biaryl-substituted imidoyl radicals on the μs time scale.

## Introduction

**Figure 1:**
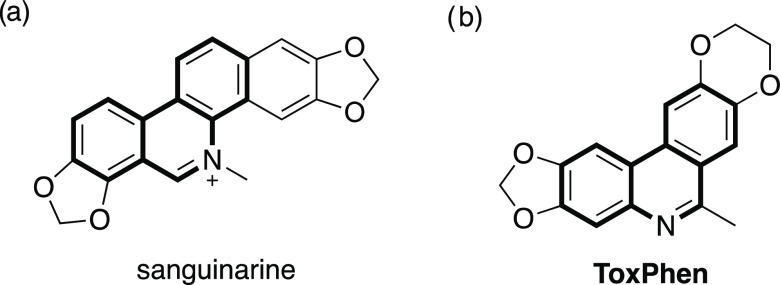
(a) Structure of sanguinarine containing the phenanthridine skeleton. (b) An antitumor phenanthridine derivative **ToxPhen** via extremely rapid conversion of the biaryl precursor inside living cells of KB cancer spheroids.

Phenanthridines (benzo[c]quinolines) are planar fused aromatic N-heterocyclic molecules, and the molecular skeleton is found in several biologically active alkaloids^1^ including sanguinarine^2^ (Figure 1a) and chelerythrine.^3^ This has led to phenanthridine derivatives being of great interest in organic synthesis.^4^ The cytotoxic phenanthridine **ToxPhen** can be rapidly generated inside living cells of KB cancer spheroids (epidermal carcinoma) via bioorthogonal intramolecular imination of the nontoxic biaryl precursor (Figure 1b).^5^ The in-cell generation of **ToxPhen** indicates that intramolecular cyclizations of the biaryl precursors are reliable processes for the exploration of functional phenanthridine derivatives.

**Figure 2:**
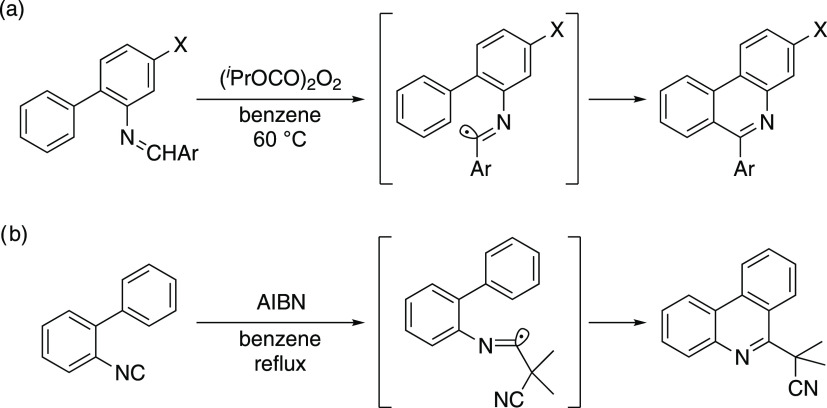
Intramolecular cyclization reactions of biaryl-substituted imidoyl radicals affording phenanthridines via (a) hydrogen abstraction and (b) isonitrile insertion.

Use of radicals for organic synthesis has been of interest and accordingly has been applied to phenanthridines. Figure 2 displays pioneering examples of radical-based synthesis of phenanthridines via intramolecular cyclization of imidoyl radical intermediates.^6^ In 1985, Leardini et al. reported hydrogen abstraction of biarylimines generating imidoyl radicals, which were converted into the corresponding phenanthridines via intramolecular cyclization and hydrogen abstraction (Figure 2a).^7^ As precursors of imidoyl radicals leading to phenanthridines, 2-isocyano-1,1′-biphenyls have also been utilized.^8^Figure 2b shows the radical isonitrile insertion process reported by Nanni et al. involving the AIBN-derived 2-cyanopropyl radical.^9^

In pharmaceutical and agrochemical studies, the use of fluorine-containing molecular units becomes an essential approach,^10−12^ and thus the synthesis of fluorinated phenanthridines has been quite important. Difluoromethyl (CF_2_H) has been an attractive fluoroalkyl group as a lipophilic bioisostere for hydroxy (OH), amino (NH_2_), and thiohydroxy (SH) groups.^13,14^Table 1 shows the reported synthesis of 6-(difluoromethyl)phenanthridines via radical isonitrile insertion. As shown in entries 1–3, the use of photoredox catalysts and electron-accepting difluoromethyl reagents has been established.^15−17^ On the other hand, a contrastive process using difluoromethyl(trimethyl)silane with silver and fluoride additives as oxidant and desilylation reagent, respectively, was reported recently (entry 4).^18^ These synthetic methods using the difluoromethyl radical should be convenient. However, in taking the importance of difluoromethylated phenanthridines in drug discovery into account, it would be desirable to develop novel and complementary synthetic methods for producing 6-(difluoromethyl)phenanthridines by radical isonitrile insertion. In addition, understanding the reaction mechanism by characterizing the short-lived intermediates should be meaningful to the synthesis. So far, electron spin resonance (ESR) measurements at low temperatures and theoretical studies on imidoyl radicals have been carried out,^19,20^ but studies on the hypothesized imidoyl radical intermediates at temperatures comparable to the reaction conditions used in this paper, ∼40 °C, have been scarce, probably because of the prompt intramolecular cyclization process.

**Table 1:**
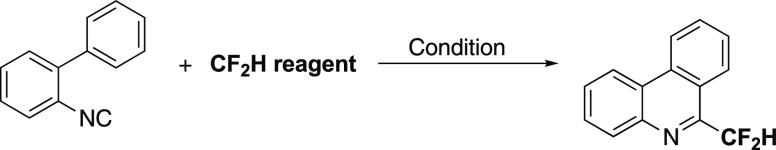
Reported Synthesis of 6-(Difluoromethyl)-phenanthridines by Radical Isonitrile Insertion

entry	CF_2_H reagent	condition	refs
1	HCF_2_SO_2_Cl	*fac*-Ir(ppy)_3_ 26 W fluorescent light Na_2_CO_3_, H_2_O, dioxane, RT	(15)
2	HCF_2_SO_2_Ar (**I**)	[Ru(bpy)_3_]Cl_2_·6H_2_O 6 W blue LED Na_2_CO_3_, DMSO, RT	(16)
3	HF_2_C-S^+^Ar_2_ BF_4_^–^ (**II**)	*fac*-Ir(ppy)_3_ 12 W blue LED KOH, PEG 600, DCM, RT	(17)
4	HF_2_C-SiMe_3_	PivOAg, PivCl, CsF DMF, 80 °C	(18)

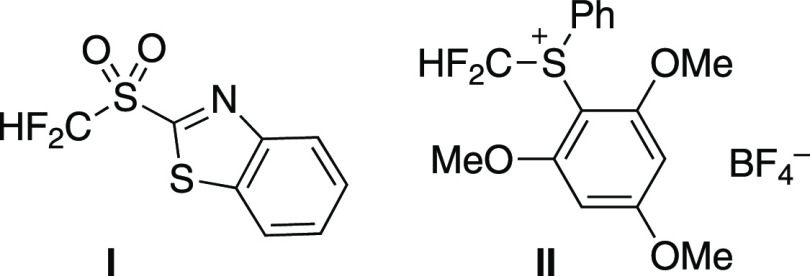

**Figure 3:**
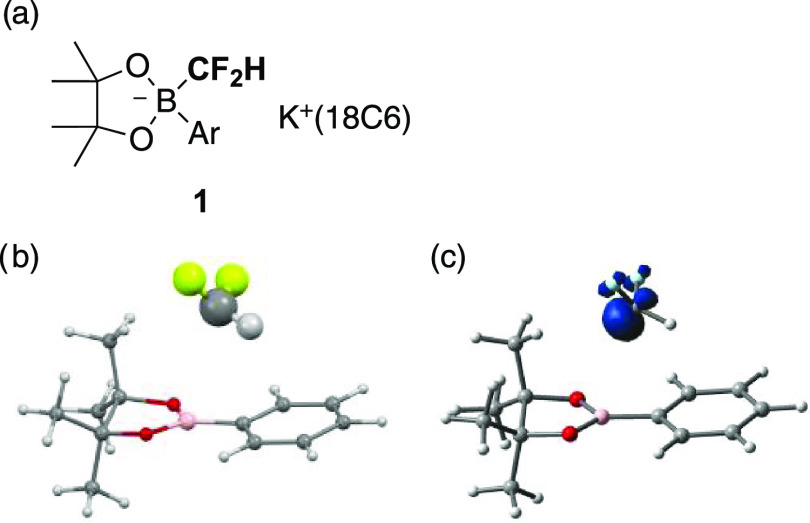
(a) Formula of aryl-substituted difluoromethylborates **1**. 18C6 means 18-crown-6 ether. (b) A DFT-optimized structure of one-electron oxidized **1A** (Ar = Ph) at UωB97XD/6-311+G(d). The B···CF_2_H distance is 3.37 Å. (c) Spin density distribution (iso = 0.03) over the one-electron oxidized **1A**.

We previously reported the synthesis, isolation, and structure determination of phenyl-substituted difluoromethylborate **1A** (Figure 3: Ar = Ph) as an air- and moisture-tolerant crystalline compound.^21^ The electron-abundant structure of **1** is promising to generate difluoromethyl radical (HF_2_C^•^) under appropriate oxidation conditions. Preliminary DFT calculations suggest that one-electron oxidation of **1A** induces considerable elongation of the B–CF_2_H bond and accumulation of spin density over the CF_2_H carbon center (Figure 3b,c). Thus, the difluoromethylborates should be useful for the synthesis of 6-(difluoromethyl)phenanthridines by radical isonitrile insertion through the somophilic process.^22^ Fortunately, our subsequent studies confirmed the possible exchange of the phenyl group in **1A** to other aryl substituents leading to various difluoromethylborates **1**, which should be applicable to develop novel procedures of radical isonitrile insertion affording 6-(difluoromethyl)phenanthridines.

In this paper, we discuss the synthetic utility of difluoromethylborates **1** via optimization of the difluoromethyl radical sources **1** for radical isonitrile insertion and check the scope of substrates for providing 6-(difluoromethyl)phenanthridines. We have performed mechanistic studies of the isonitrile insertion process using a radical scavenger, and these are accompanied by DFT calculations.

As pointed out above, mechanistic studies of isonitrile insertion have been limited so far. The presence of difluoromethyl radical has been proved by trapping with nitroxide radicals, but the subsequent reaction intermediates in the isonitrile insertion have been scarcely investigated. It would be desirable to obtain clear information about the imidoyl radical intermediates, but observing these molecules is difficult due to their short lifetimes. We have used a magnetic resonance technique called muon spin rotation (μSR) spectroscopy to observe the imidoyl radical. This technique involves implanting a beam of 100% spin-polarized positive (μ^+^) muons into the sample.^23^ The muon lifetime of 2.2 μs makes it convenient for studying fast chemical reactions and short-lived paramagnetic species.^24^ The 100% initial polarization of the positive muon, combined with single-particle detection, gives μSR much higher sensitivity per spin than either NMR or ESR. A fraction of the implanted muons will form muonium [Mu = μ^+^e^–^], which behaves chemically like a light isotope of atomic hydrogen (H). Another fraction of the implanted muons will end up in diamagnetic environments, which cannot be distinguished as the short lifetime of the muon leads to the uncertainty in the precession frequency being larger than any chemical shift. Mu adds to unsaturated bonds in organic molecules.^25^ Transverse-field muon spin rotation (TF-μSR) studies show that Mu adds to the isonitrile functional group of 2-isocyano-4′-methoxy-1,1′-biphenyl as well as to the phenyl rings. This is the first time an imidoyl radical has been characterized by μSR, and it is one of only a few muoniated σ-radicals observed. This is closely related to the reaction of Mu with N-heterocyclic carbenes where Mu was been observed to add exclusively to the carbeneic carbon.^26^ In addition, the kinetics of isonitrile insertion processes are discussed in relation to the temperature dependence of the decay of the muoniated imidoyl radical signal.

## Results and Discussion

### Optimization of Difluoromethylborate for Radical Isonitrile Insertion

**Scheme 1:**
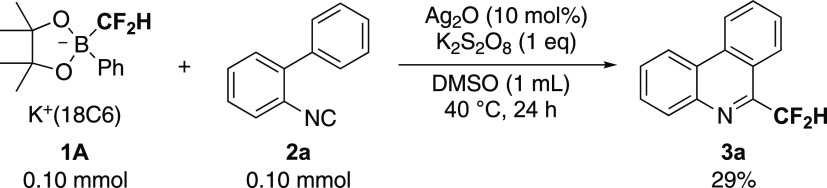
Radical Isonitrile Insertion Generating 6-(Difluoromethyl)phenanthridine Using Phenyl-Substituted Difluoromethylborate **1A**

We started by screening the chemical oxidation conditions for radical cyclization of **2a** using **1A**. As displayed in Table S1, we concluded that a combination of silver oxide (Ag_2_O)^27^ and potassium peroxodisulfate (K_2_S_2_O_8_) was suitable to initiate radical isonitrile insertion with **1A** affording 6-(difluoromethyl)phenanthridine **3a**. Also, screening of solvents and temperature confirmed that the reaction in DMSO at 40 °C was appropriate (Scheme 1 and Table S1). Decomposition of **2a** generating small amounts of the formamide and amine was also observed. Chloranil, DDQ, TCNQ (7,7,8,8-tetracyanoquinodimethane), and Mn(OAc)_3_·2H_2_O^28^ did not give the desired product. Attempted synthesis of **3a** using F_3_B–CF_2_H·K^+^(18C6)^21^ was also unsuccessful.

**Scheme 2:**
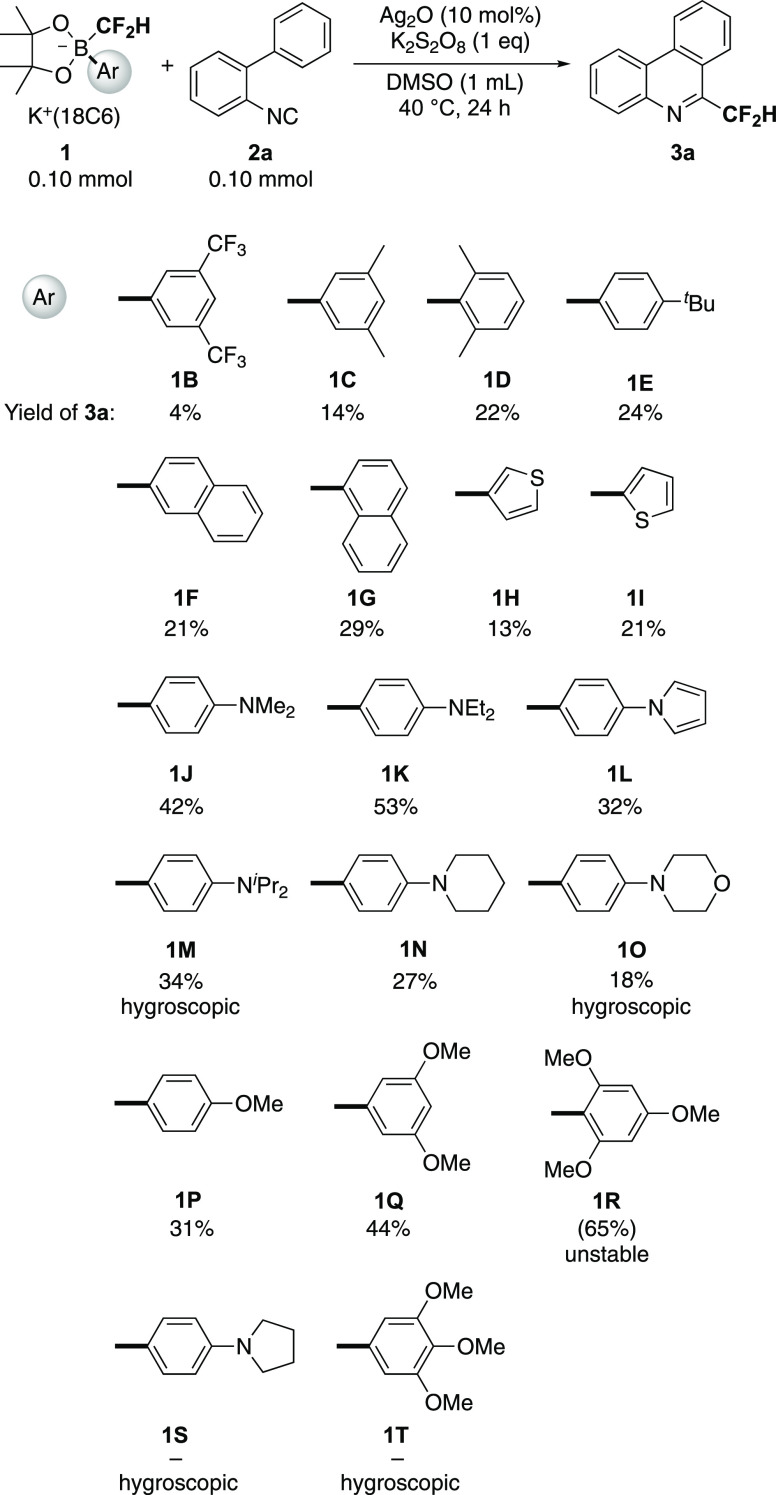
Screening of the Ar Group in **1** for Radical Isonitrile Insertion Affording **3a** Yields were determined by ^19^F NMR using benzotrifluoride (BTF) as an internal standard.

Next, we attempted to improve the yield of **3a** in Scheme 1. It is plausible that an increase of electron density around the boron atom of **1** facilitates the generation of the difluoromethyl radical from **1** upon oxidation. According to the reported procedures,^21^ we synthesized difluoromethylborates **1B-T**, which were used in the radical isonitrile insertion reactions to produce **3a** (Scheme 2). The electron-withdrawing CF_3_ group in **1B** reduced the yield of **3a**. The xylyl groups in **1C** and **1D** also resulted in lower yields. The *p*-*t*-butylphenyl unit in **1E** gave **3a** in a comparable yield with **1A**. Naphthyl groups in **1F** and **1G** also gave **3a** in comparable yields with **1A**. Heteroaromatic thienyl groups in **1H** and **1I** did not improve the yield. We found that the dimethylamino and diethylamino groups in **1J** and **1K** were quite effective and provided **3a** in 42 and 53% yields, respectively. On the other hand, pyrrole-substituted **1L** gave a comparable yield of **3a** with **1A**. Diisopropylamino-substituted **1M** was considered because of its potent electron-donating property but was found to not be convenient for the synthesis of **3a** due it being hygroscopic. The piperidyl and morpholino groups in **1N** and **1O** produced lower yields of **3a** compared with that in **1A**. In addition, the morpholino-substituted **1O** was difficult to work with as it was hygroscopic. The *p*-methoxyphenyl-substituted **1P** gave a comparable yield to **1A**, and the use of methoxy substituent was promising. The dimethoxyphenyl group in **1Q** improved the yield of **3a** compared with that of **1A**. The 2,4,6-trimethoxyphenyl group in **1R** gave **3a** in a 65% crude yield, but **1R** was highly hygroscopic and unstable, and there were difficulties in isolating **3a**. Thus, **1R** was deemed to be inconvenient for further synthetic works. Although *p*-pyrrolidinylphenyl- and 3,4,5-trimethoxyphenyl-substituted **1S** and **1T** were considered promising due to their high electron-donating property, they were very hygroscopic and were not appropriate for the synthesis of **3a**. Consequently, we concluded that the diethylamino-substituted derivative **1K** is most appropriate for the subsequent studies of radical isonitrile insertion to produce 6-(difluoromethyl)phenanthridines.

To discuss the efficiency of the ^•^CF_2_H generation from **1** in detail, we carried out electrochemical measurements. Table S2 summarizes the oxidation potentials determined by differential pulse voltammetry (DPV) and the highest occupied molecular orbital (HOMO) levels of **1**, indicating that the appropriate HOMO levels of **1** should be requisite to synthesize **3a** under the chemical oxidation condition (Table S2 and Figure S1). The excessively high HOMO levels of difluoromethylborates were not necessarily suitable for the synthesis of 6-(difluoromethyl)phenanthridines. In fact, the highly electron-donating substituents showed undesirable hygroscopic character as well as instability. As for the aminophenyl-substituted derivatives **1J**, **1K**, and **1N**, the lower oxidation potentials (0.14–0.19 V vs SCE) caused by the lone pair and aryl unit (π-HOMO) were also observed. As shown in Figure S2, the spin density in one-electron oxidized **1J** is localized over the aryl unit, and the difluoromethyl unit is still bound to the boron. The possible two-electron oxidation process in the amino-substituted derivatives might improve the yields of **3** by using excess amounts of K_2_S_2_O_8_ (vide infra). However, the effective two-electron oxidation of **1J** for releasing difluoromethyl radical should be of higher energy (Figure S3). Although detailed investigations are desirable, it is plausible that the chemical oxidation with equimolar K_2_S_2_O_8_ would be enough to release sufficient difluoromethyl radical even from the aminophenyl-substituted difluoromethylborates.

### Substrate Scope

**Scheme 3:**
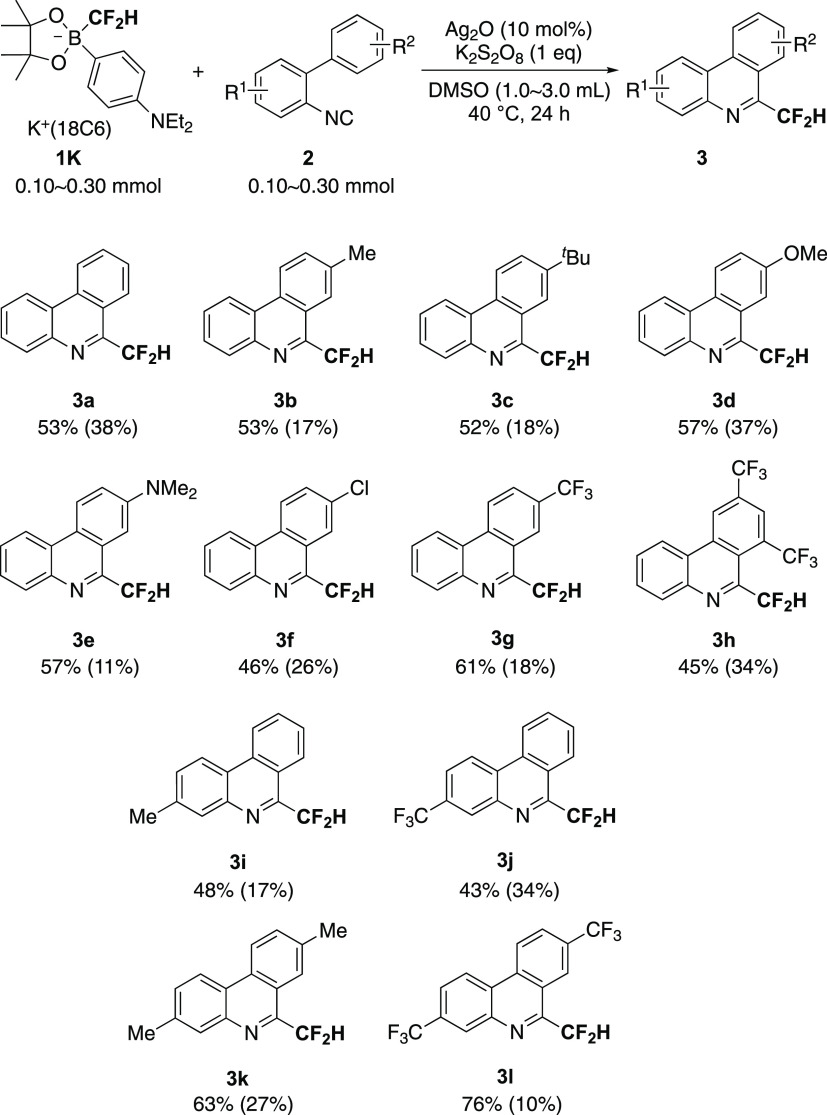
Synthesis of 6-(Difluoromethyl)phenanthridines **3** by Radical Isonitrile Insertion with **1K**^,^ Yields were determined by ^19^F NMR using benzotrifluoride (BTF) as an internal standard. Isolated yields of 3 are shown in parentheses

As discussed above, *p*-diethylaminophenyl-substituted borate **1K** should be convenient as a source of difluoromethyl radical because of its high stability. Next, we examined the effect of substrates on the radical isonitrile insertion with an equimolar amount of **1K** affording 6-(difluoromethyl)phenanthridines **3** (Scheme 3). Almost all of the isocyanophenyl substrates **2a**–**l** could be converted into the corresponding substituted 6-(difluoromethyl)phenanthridines **3a**–**l** without a significant change of yields. Because separating the unreacted isonitrile **2** was quite difficult by chromatography, the yields were estimated based on ^19^F NMR. Purification was accomplished by subsequent chromatography or recrystallization although several 6-(difluoromethyl)phenanthridines such as **3c**, **3d**, **3f**, **3g**, **3i**, and **3k** included the corresponding reactants **2** even after the extensive purification (see the Supporting Information). Both electron-donating and -withdrawing groups in **2** are tolerant. Thus, the difluoromethylborate unit in **1** is one of the reliable sources of the difluoromethyl radical for the synthesis of 6-(difluoromethyl)phenanthridines. The attempted gram-scale reaction using 1.60 mmol of **1K** and **2d** gave **3d** in a 52% yield (determined by ^19^F NMR), and the product could be purified by recrystallization. The isolated yield of the pure form was at least 25%, and a crude mixture containing a trace amount of starting materials was obtained (ca. 20%). Use of 2 equiv of K_2_S_2_O_8_ slightly improved the NMR-based yields of **3** (**3d**: 64%, **3f**: 61%).

### Mechanistic Studies of Radical Isonitrile Insertion Affording 6-(Difluoromethyl)phenanthridines

**Scheme 4:**
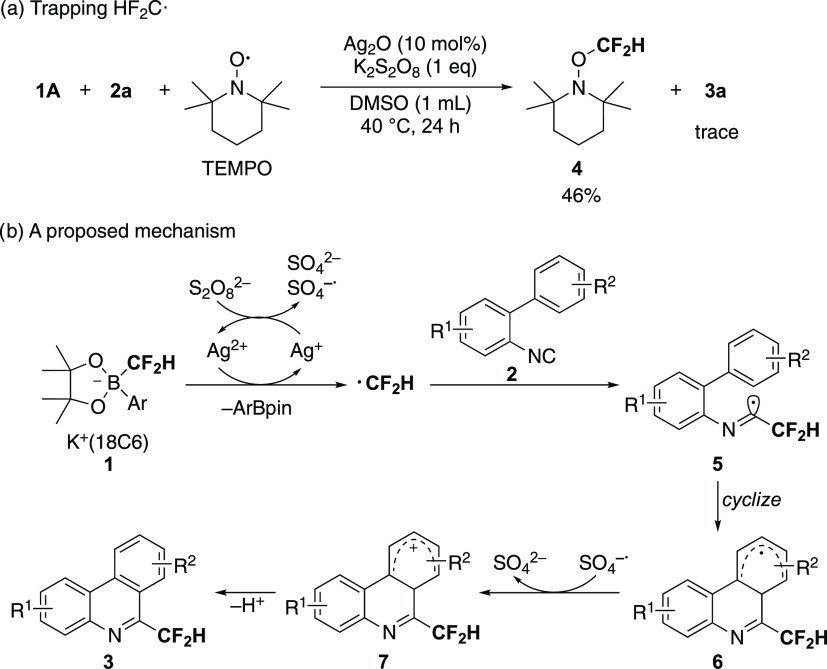
(a) Trapping Difluoromethyl Radical with TEMPO. (b) A Proposed Reaction Mechanism of Radical Isonitrile Insertion Affording **3**

Scheme 4a supports the generation of the difluoromethyl radical from **1a** upon oxidation. The stable nitroxide radical 2,2,6,6-tetramethyl-1-piperidinoxyl (TEMPO) is a functional scavenger of HF_2_C^•^, and a 1:1:1 mixture of **1A**, **2a**, and TEMPO gave **4** predominantly. Scheme 4b shows a proposed reaction mechanism on the basis of this result and previous reports.^27^ Peroxydisulfate oxides Ag(I) and Ag(II) oxidizes **1** to generate a difluoromethyl radical together with ArBpin. The resulting arylboronate ArBpin was observed in the reaction mixture. The difluoromethyl radical adds to the carbon atom of the isonitrile group in **2**, providing the imidoyl radical **5**, and subsequent intramolecular cyclization of **5** results in the paramagnetic precursor **6**. Single-electron oxidation of the cyclohexadienyl radical unit in **6** provides the cyclohexadienyl cation derivative **7**, and deprotonative aromatization furnishes 6-(difluoromethyl)phenanthridine **3**.

**Figure 4:**
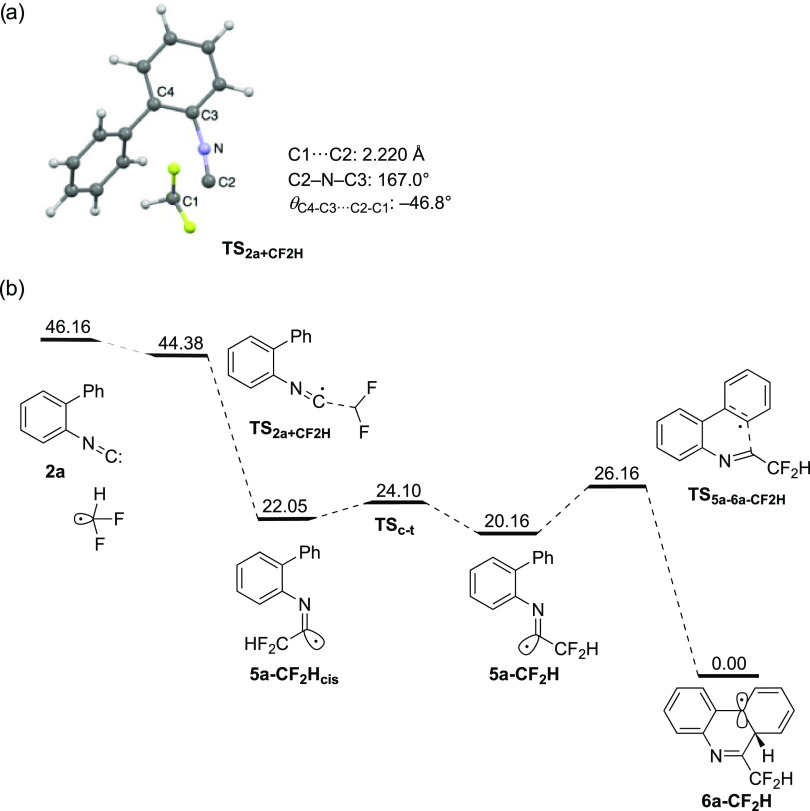
(a) Drawing of the transition state for CF_2_H radical addition to **2a** (**TS**_**2a+CF2H**_) [UωB97XD/6–311G(d,p) SMD = DMSO]. (b) An enthalpy (Δ*H*, kcal/mol) diagram of the radical isonitrile insertion from **2a** and difluoromethyl radical leading to **6a-CF**_**2**_**H** through the imidoyl radical **5a-CF**_**2**_**H** [UωB97XD/6-311G(d,p) SMD = DMSO, 313.0 K, 1.0 atm].

DFT calculations were performed to obtain information about the radical cyclization process from **5** to **6**. Figure 4a shows an optimized transition state (TS) of CF_2_H radical addition to **2a** (**TS**_**2a+CF2H**_). The structure is like that of CH_3_ radical addition to 1-isocyano-2-vinylbenzene.^29^ A potential energy surface (PES) scan for **TS**_**2a+CF2H**_ indicated the predominant formation of cis-configured imidoyl radical **5a-CF**_**2**_**H**_**cis**_ (Figure S4). Figure 4b displays an enthalpy diagram of CF_2_H radical addition to **2a** leading to a three-cyclic paramagnetic **6a-CF**_**2**_**H**. The energy of activation (Δ*H*^‡^) for CF_2_H radical addition to **2a** is negative, indicating the prereaction complexes with an entropic penalty.^29^ It should be mentioned that the Gibbs free energy of activation (Δ*G*^‡^) for CF_2_H radical addition to **2a** of 10.3 kcal/mol is comparable to CF_3_ or CH_3_ radical addition to isocyanobenzene [8.6 kcal/mol for CF_3_, 12.6 kcal/mol for CH_3_, UM06-2X(D3)/6-311++G(d,p)].^29^ The initially generated cis-imidoyl radical **5a-CF**_**2**_**H**_**cis**_ should be isomerized to **5a-CF**_**2**_**H**. The radical cyclization step from **5a-CF**_**2**_**H** to **6a-CF**_**2**_**H** through **TS**_**5a-6a-CF2H**_ requires an activation energy of 6.0 kcal/mol (313.0 K, 1 atm). The reactants (**2a** and CF_2_H radical) and the TS for radical addition (**TS**_**2a+CF2H**_) have considerably larger energies compared with the TS for cyclization (**TS**_**5a-6a-CF2H**_), indicating that the cyclization reaction proceeds quite rapidly after the generation and addition of the CF_2_H radical.

### Observation of the Imidoyl Radical Intermediate in the Isonitrile Insertion via Addition of Muonium

It would be desirable to confirm that radical addition occurs primarily at the carbon of the isonitrile functional group and that this generates the imidoyl radical. In addition, it would be desirable to understand the radical isonitrile insertion process. This would aid in the development of synthetic technologies toward functional nitrogen heterocycles. As noted in the Introduction section, Mu is a free radical that can add to unsaturated bonds to create paramagnetic intermediates, which can be characterized by μSR. We have studied the muoniated radicals produced by Mu addition to a biphenylisonitrile compound in order to learn more about the proposed imidoyl radical intermediate. There have been no previous reports of μSR measurement of imidoyl radicals.

There are competing pathways for Mu addition, and the identity and yield of the muoniated products depend on the number of sites and the activation barrier.^23d^ We are assuming that the pre-exponential factor would be the same as we are considering Mu adding at different sites on the same molecule. As a first step, we have calculated the structures and energies of the species involved in Mu addition to the simplified structure **2a**. We then calculated the enthalpies of activation for Mu addition at the isonitrile group and for the subsequent cyclization and for Mu addition to the phenyl rings.

**Figure 5:**
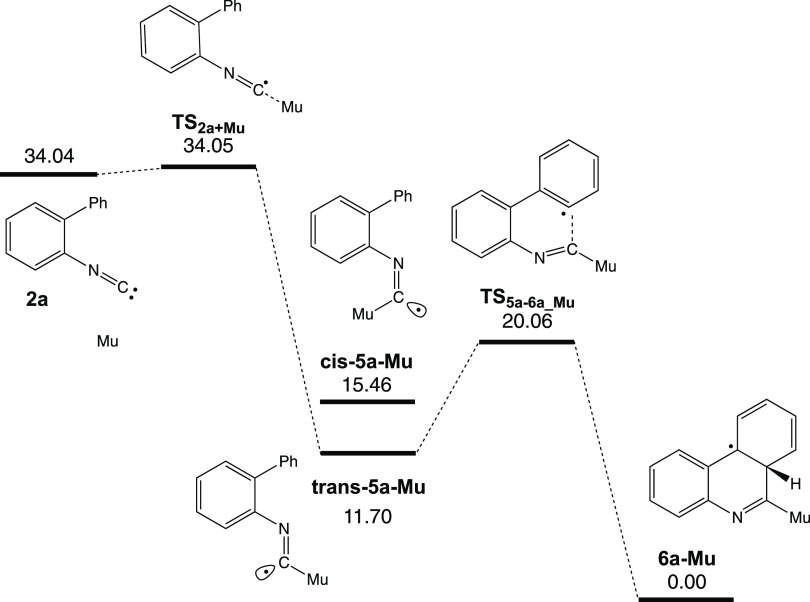
Enthalpy (Δ*H*, kcal/mol) diagram of Mu addition to the isonitrile group of **2a**, leading to **6a-Mu** through imidoyl radical **trans-5a-Mu** [UB3LYP/6-311+G(d,p)]. The vibrational frequencies and energies were computed with 0.1134 amu for muonium.

We first considered Mu addition to the carbon of the isonitrile group (Figure 5). The energies and vibrational frequencies of these structures were computed using a mass of 0.1134 amu for muonium. Mu addition at the carbon of the isonitrile group forms the imidoyl radical **trans-5a-Mu**, which in turn can undergo a cyclization reaction to form **6a-Mu**. The calculated enthalpy of activation (Δ^‡^*H*) for Mu addition to the carbon of the isonitrile group is 0.01 kcal/mol. The Arrhenius activation, *E*_a_, equals Δ^‡^*H* + R*T* and is 0.61 kcal/mol. This cis isomer (**cis-5a-Mu**) is 3.76 kcal/mol higher in energy than the trans isomer and is likely present only in very low concentrations. The cyclization reaction involves an approximately 180° rotation about the C_phenyl_–N bond and torsional motion of the ortho-substituted phenyl ring. The barrier to cyclization (**TS**_**5a-6a_Mu**_) was calculated to be ∼14 kcal/mol lower than the combined energy of Mu and **2a**, which could result in rapid reaction, similar to what was observed for the reaction of Mu with ketene.^30^ Alternative DFT calculations at the UωB97XD/6-311G(d,p) level gave the comparable results with Figure 5 (Figure S10).

**Figure 6:**
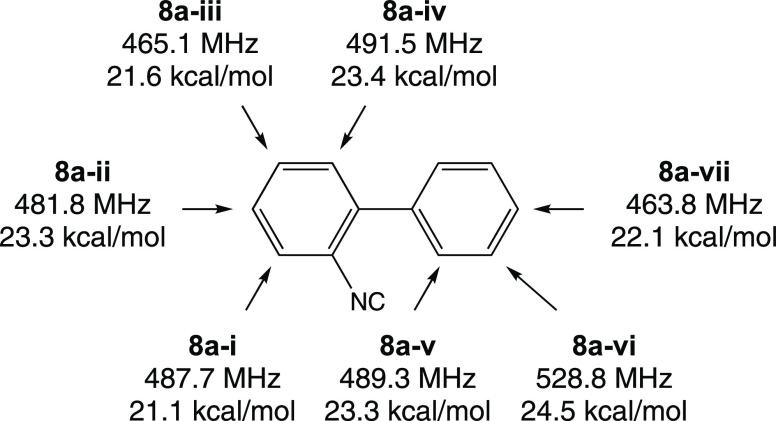
Calculated muon hyperfine coupling constants and relative enthalpies [UB3LYP/6-311+G(d,p)] with respect to **6a-Mu** of the muoniated cyclohexadienyl radicals formed by Mu addition to **2a**. The arrows indicated the position of Mu addition. The muon hfcs were obtained by scaling the average hfc of the protons in the methylene group by a factor of 1.20 to account for the vibrational averaging due to the light mass of the muon and then multiplying by a factor of 3.183 to account for the muon’s larger gyromagnetic ratio compared with the proton.

We also calculated the structures and energies of the muoniated cyclohexadienyl (CHD) radicals that could form by Mu addition to **2a** (Figure 6). We have assumed that addition only occurs at the secondary aromatic carbons as numerous experiments have shown that this is strongly preferred compared with the addition at the tertiary carbons.^31,32^ The lowest-energy muoniated CHD radical (**8a**-**i**) is ∼9.4 kcal/mol higher in energy than **trans-5a-Mu**.

**Figure 7:**
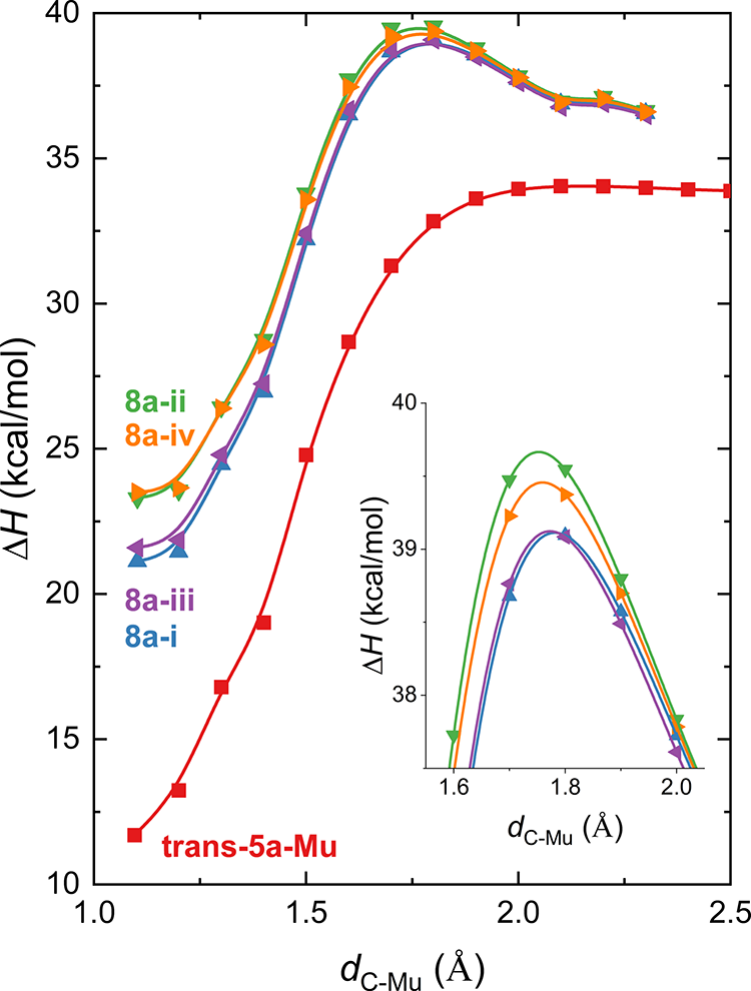
Distinguished coordinate reaction paths (Δ*H*, kcal/mol) for Mu addition to the isonitrile group of **2a** yielding **trans-5a-Mu** and Mu addition to the phenyl ring yielding radicals **8a-i** through **8a-iv**. The inset shows the region near the transition states for the formation of the muoniated CHD radicals.

We calculated the barriers for Mu addition at the secondary carbons of the phenyl ring to generate radicals **8a-i** through **8a-iv**. The enthalpy of activation for Mu addition at the isonitrile group is considerably lower than that for the formation of muoniated CHD radicals (Figure 7). This indicates that the formation of **trans-5a-Mu** will be more rapid than the CHD radicals and that **trans-5a-Mu** will be the main product. The enthalpies of activation for the formation of **8a-i** and **8a-iii** are lower than those for the formation of **8a-ii** and **8a-iv**. This means that **8a-i** and **8a-iii** will be formed in greater yield than the other CHD radicals. The calculated Δ^‡^*H* for the formation of **8a-i** and **8a-iii** are ∼5.1 kcal/mol, which gives *E*_a_ values of 5.7 kcal/mol. The calculated *E*_a_ for Mu addition to benzene is 6.0 kcal/mol while the experimental value in the gas phase is 1.6 ± 0.1 kcal/mol.^33^ The difference between the experimental and calculated values is likely due to the importance of tunneling.

The muon hyperfine coupling constants (*A*_μ_) of the possible Mu adducts of **2a** were calculated using the UB3LYP/6-311+G(d,p) method. A factor of 3.183 was included to account for the larger gyromagnetic ratio (γ_μ_) of the muon compared with that of the proton (γ_p_). The light mass of the muon affects the vibrational modes of a radical and leads to a C-Mu bond being about 5% longer than the corresponding C–H bond.^34^ This has a large effect on the hyperfine coupling constants (hfcs) of a muoniated radical. We have accounted for the light mass of the muon in two ways. For the calculation of *A*_μ_ of **trans-5a-Mu**, **cis-5a-Mu**, and **6a-Mu**, we determined the vibrationally averaged hyperfine parameters using the Fermi keyword in Gaussian 16. This means there are no empirical factors included in the calculation, but it is very computationally expensive. **Trans-5a-Mu** has an *A*_μ_ of 707.8 MHz at 300 K, **cis-5a-Mu** has an *A*_μ_ of 875.1 MHz, while **6a-Mu** has an *A*_μ_ of −5.1 MHz. For the Mu adducts of the biphenyl ring, we used a less computationally expensive method in which we scaled the average hfc of the protons in the methylene group by a factor of 1.20 to account for the vibrational averaging and then multiplied by a factor of 3.183 to account for the muon’s larger gyromagnetic ratio. This approach has been successful in calculating *A*_μ_ for muoniated cyclohexadienyl-type radicals, such as the Mu adducts of the polyaromatic hydrocarbons pyrene and fluoranthene.^31,32^ This also accounts for the motion of the two phenyl rings. The *A*_μ_ values of the Mu adducts of the biphenyl ring range from 463.8 to 528.8 MHz and are shown in Figure 6. These values are in line with the measured values for the Mu adducts of the liquid crystal 5CB (4-*n*-pentyl-4′-cyanobiphenyl), which contains a biphenyl ring and a cyano substituent, where the muon hfcs range from 446.6 to 491.2 MHz.^35^ There is also good agreement with the Mu adducts of biphenyl where the ortho, meta, and para isomers have muon hfcs of 463.9, 515.4, and 422.1 MHz, respectively.^36^

TF-μSR experiments were performed on a deoxygenated 0.49 M solution of 2-isocyano-4′-methoxy-1,1′-biphenyl (**2d**) in THF to determine what radicals were formed by Mu addition. This compound was chosen for μSR measurements due to its stability. The measurements were performed on the M20 beamline at TRIUMF with the Helios spectrometer. The experimental setup for TF-μSR measurements is shown in Figure S5. Two sets of orthogonal detectors were used, which allows us to distinguish positive and negative precession frequencies via a complex Fourier transformation.

**Figure 8:**
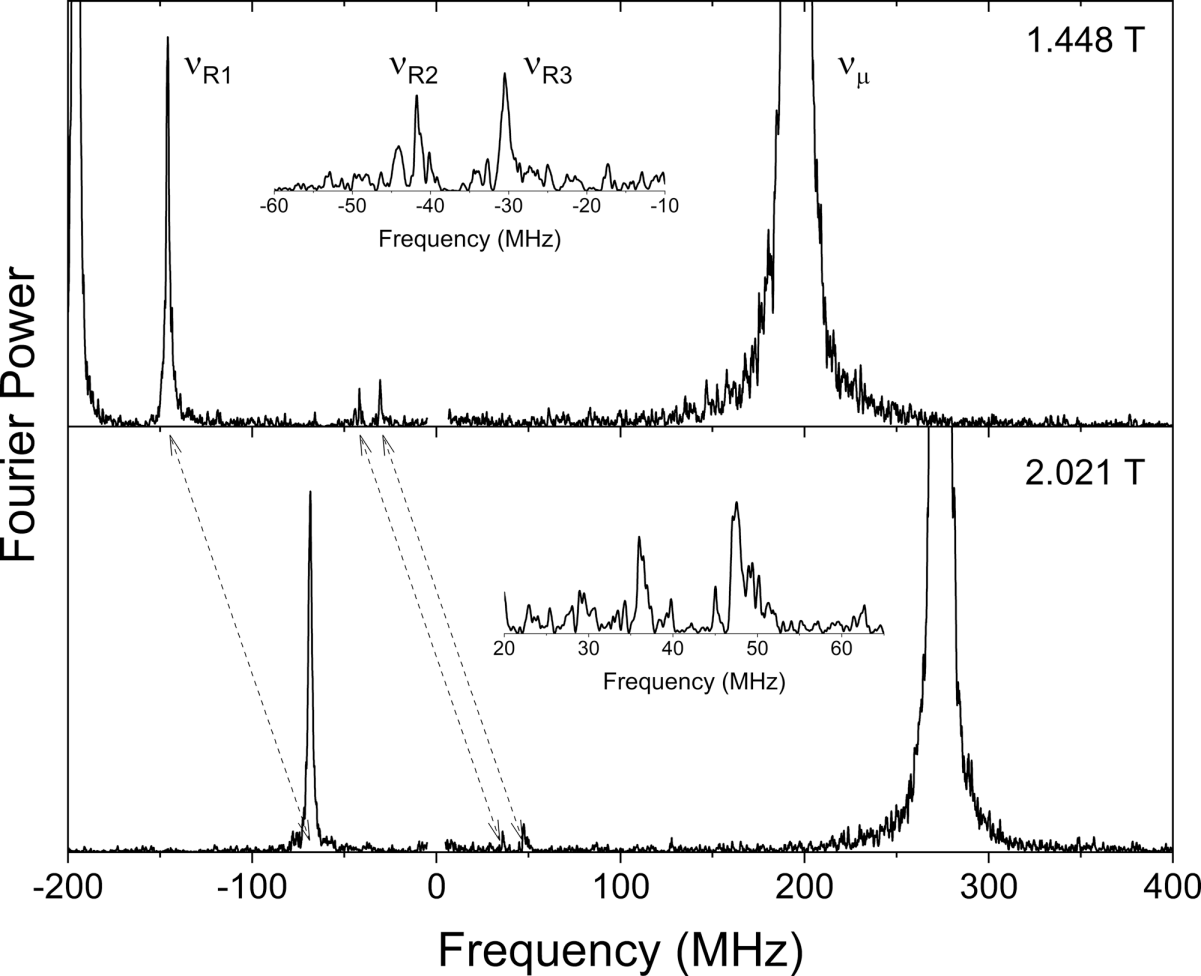
TF-μSR spectrum of a THF solution (0.49 M) of **2d** at 298.7 K. Top panel: the diamagnetic signal (ν_μ_) at 195.6 MHz corresponds to the transverse magnetic field of 1.448 T. The reflection of this signal is observed at −195.6 MHz. The lowest paramagnetic signal (ν_R1_: −146.0 MHz) is due to the imidoyl radical **5d-Mu** generated from **2d**. The small paramagnetic signals (ν_R2_: −41.7 MHz; ν_R3_: −30.5 MHz) are due to cyclohexadienyl (CHD) radicals formed by Mu addition to the benzene rings of **2d**. The higher-field paramagnetic signals are not observed due to the time resolution property of the spectrometer. The region around zero frequency has not been displayed due to an artifact from the Fourier transformation. Bottom panel: the diamagnetic signal (ν_μ_) at 273.9 MHz corresponds to the transverse magnetic field of 2.021 T. The radical signals have shifted to −68.6, 36.2, and 47.3 MHz.

TF-μSR spectra of a THF solution of **2d** at 298.7 K are shown in Figure 8. Muons in a diamagnetic environment process at the muon Larmor frequency ν_μ_ = γ_μ_*B*, where γ_μ_ is 135.5 MHz/T and *B* is the applied magnetic field. The chemical shift of diamagnetic muons cannot be measured due to the short lifetime of the muon. Muons in free radicals are subject to the local field due to the unpaired electron—the hyperfine interaction. In high transverse fields, each type of radical gives rise to two precession signals at
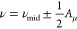
1where
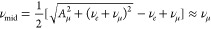
2ν_e_ is the electron frequency. *A*_μ_ can be determined either from the difference between the two radical precession signals or when the higher-frequency signal is not observed due to the time resolution of the spectrometer, as in this case, by twice the difference between one of the radical signals and the diamagnetic signal ν_μ_.

TF-μSR measurements were performed with two transverse magnetic fields in order to confirm whether the weaker signals were real or artifacts as real radical signals would shift by the same amount as the diamagnetic signal.^23d^ The magnetic fields of 1.448 T and 2.021 T correspond to ν_μ_ of 195.6 and 273.9 MHz, respectively. The weak radical signals were confirmed to be real as they shifted by ∼78 MHz, which is the same amount ν_μ_ shifted by. Three types of muoniated radicals were observed, which we have labeled R1, R2, and R3. In each case, we only observed the low-frequency precession signal, which we have labeled ν_R1_, ν_R2_, and ν_R3_. The muon hfcs were determined using eqs 1 and 2. The largest radical signal, ν_R1_, corresponds to a radical with *A*_μ_ = 683.9 ± 0.7 MHz. The small radical signals ν_R2_ and ν_R3_ correspond to muon hfcs of 474.6 and 452.2 MHz, respectively.

**Figure 9:**
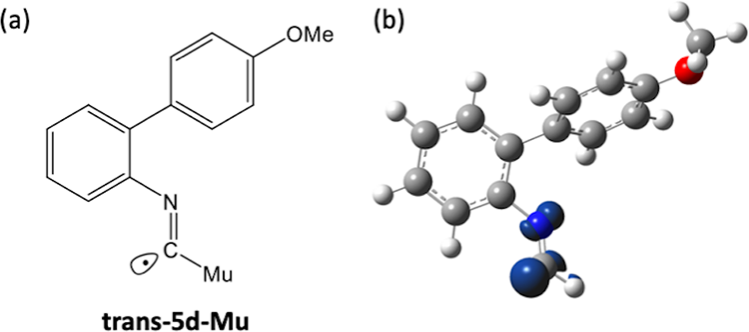
(a) Structure of **trans-5d-Mu**. (b) A DFT-optimized structure of imidoyl radical **trans-5d-Mu** at the UB3LYP/6-311+G(d,p) level. The conformational effects around the MeO group are negligible. Spin density distribution (iso = 0.02) showing most of the spin density in a σ molecular orbital.

The assignment of the radicals to a particular structure is based on the similarity between the experimental and calculated *A*_μ_ values. We have assumed that the methoxy substituent would not have a significant effect on the hyperfine parameters and that there would be negligible differences between the radicals based on **2a** and **2d**. We can immediately conclude that radical R1 is not the cyclized product **6d-Mu** as this calculated to have a very small *A*_μ_ value. Nor can it be a muoniated CHD radical as *A*_μ_ is significantly larger than the range of calculated or experimental muon hfcs for this type of radical. R1 cannot be the **cis-5d-Mu** radical as that has a significantly larger *A*_μ_ value and is a higher energy structure. We have assigned R1 to be the imidoyl radical **trans-5d-Mu** (Figure 9a). There is a very good agreement between the experimental value and calculated values.

**Figure 10:**
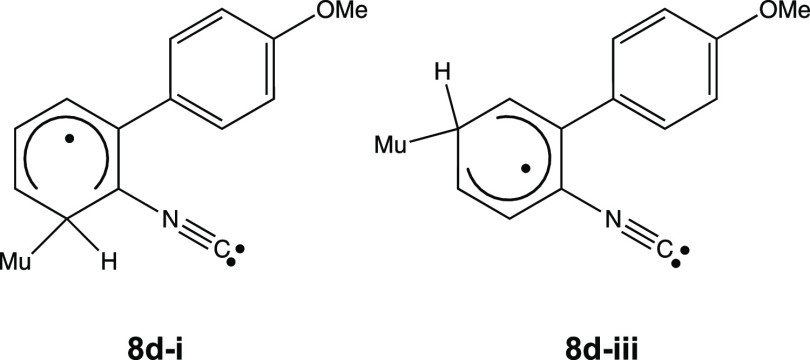
Assigned structures for muoniated CHD radicals generated by Mu addition to the phenyl rings of **2d**.

We have assigned R2 and R3 to be muoniated CHD radicals generated by muonium addition to the benzene ring of **2d**. The lowest-energy structures are formed by Mu addition at the ortho and para positions with respect to the isonitrile group. Based on the values of the muon hfc, the relative energies of the isomers, and the lower addition barriers, we assign R2 to be **8d-i** and R3 to be **8d-iii** (Figure 10). The other muoniated CHD radicals have significantly higher energies. The analog of **8a-vii** does not form, even though it has a relatively low energy, as the methoxy substituent of **2d** blocks Mu addition at that site.

The relative yield of each type of radical was determined from the amplitude of the radical signals obtained from time domain fits of the spectra. We used a weighted average of all four histograms. The major product is the imidoyl radical **trans-5d-Mu** with a relative yield of 78 ± 5% at 298.7 K. The muoniated CHD radicals **8d-i** and **8d-iii** are minor products, with relative yields of 12 ± 3 and 10 ± 3%, respectively. Our expectation is that the rate constant for Mu addition to the biphenyl rings should be similar to that Mu addition to benzene. This has a value of (8.5 ± 1.3) × 10^9^ M^–1^ s^–1^ in methanol.^37^ The large relative yield of **trans-5d-Mu** compared with the muoniated CHD radicals indicates that Mu addition to the carbon of the isonitrile group is significantly faster than this, i.e., on the order of 10^10^ M^–1^ s^–1^, which is close to the diffusion-controlled limit.

Muoniated radicals are only observed in a TF-μSR spectrum if the radical is formed within a few nanoseconds. Generally, the initial radical to be formed is the one observed by TF-μSR, except when the subsequent step, such as in the loss of CO from the muoniated acyl radical to give the CH_2_Mu radical,^30^ is on a similar time scale. This occurs when the barrier to the second reaction lies below the barrier to Mu addition. In contrast, when the barrier to the second reaction is greater than that of the barrier to Mu addition, one observes the initial muoniated radical. This was observed for Mu addition to isocyanate compounds.^38^ We suggest that excess energy is distributed in vibrational modes of **trans-5a-Mu** that are not related to the cyclization reaction.

### Temperature Effects on the Muoniated Imidoyl Radical

**Figure 11:**
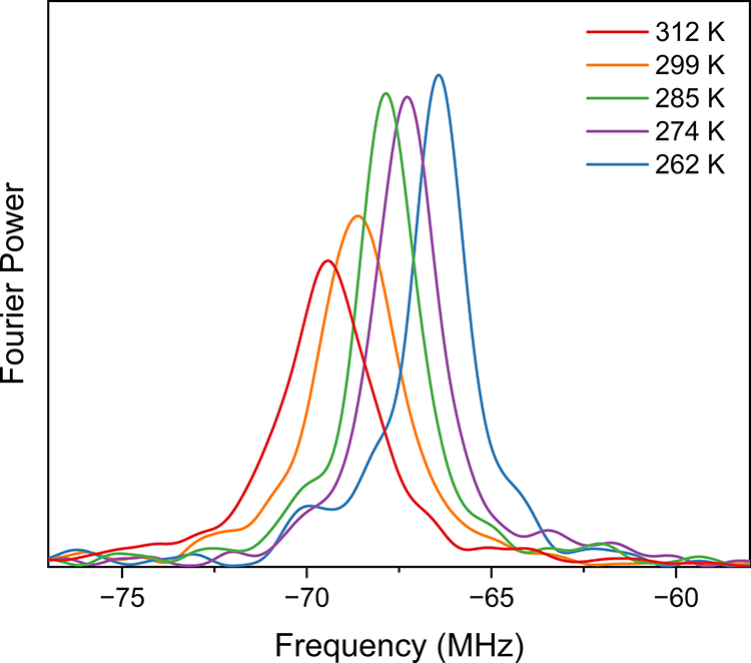
Temperature dependence of the paramagnetic ν_R1_ signal of **trans-5d-Mu** in the TF-μSR spectra of a THF solution (0.49 M) of **2d**.

The signal due to the imidoyl radical **trans-5d-Mu** in the TF-μSR spectrum is shown in Figure 11 at several temperatures between 262 and 312 K. It is apparent by eye that the amplitude, position, and width of the radical signal change with temperature. The muon hfc was determined from the peak position, and the width is related to the decay of the radical signal, λ. The temperature dependence of these parameters, as well as the amplitude, is shown in Figure 11.

**Figure 12:**
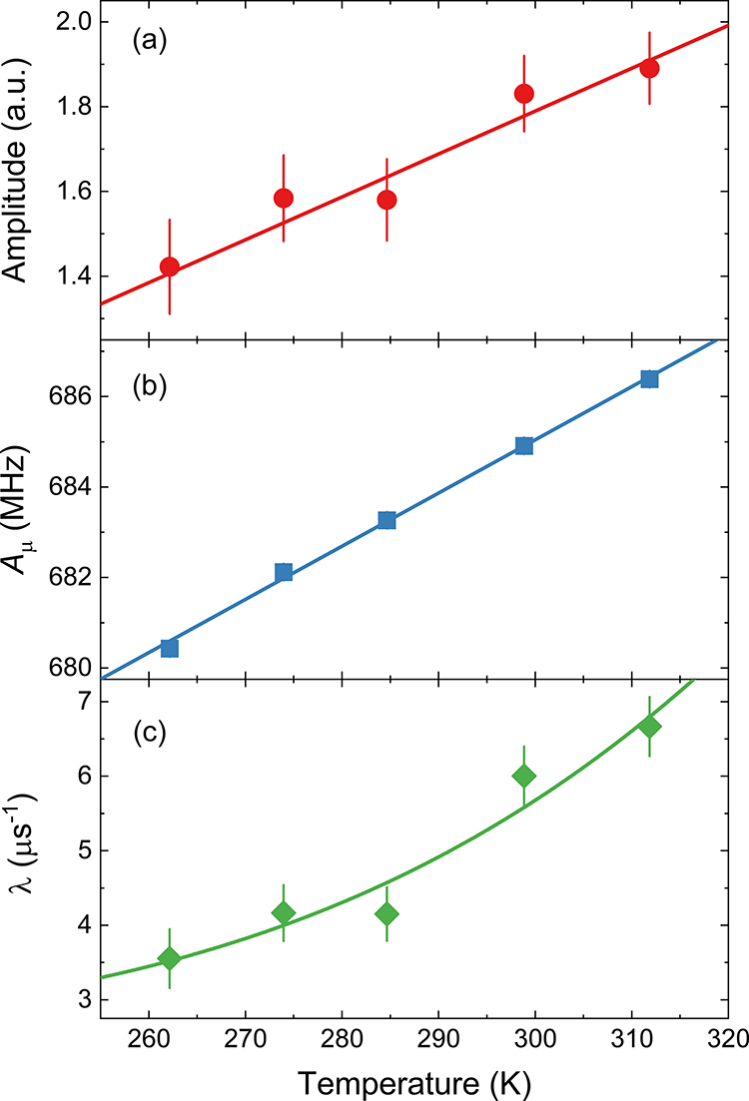
Temperature dependence of the (a) amplitude, (b) muon hfc, and (c) relaxation rate of **trans-5d-Mu** in THF solution.

The amplitude of the radical signal of **trans-5d-Mu**, as determined from fits in the time domain, increases with increasing temperature, as do those of the muoniated CHD radicals **8d-i** and **8d-iii**. The amplitude of the peak in the Fourier transform spectrum (Figure 12a) appears to go down but this is due to the width of the signal increasing. The amplitude of each signal is proportional to the polarization transferred from Mu to the radical (*P*_R_). In a high transverse field, the muon polarization transferred from Mu, where ω_Mu_ = 2π × 4463 MHz in vacuum, to a radical with precession frequency ω_R_ = 2π*A*_μ_, has the form
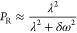
3where λ = *k*_Mu_[**2d**], *k*_Mu_ is the second-order rate constant for Mu addition, [**2d**] is the concentration of **2d**, and δω = ω_Mu_ – ω_R_.^39^ The dependence of the polarization transferred to the radical from Mu on the reaction rate is due to dephasing of the muon spin in Mu prior to reaction. The muon hfc of **trans-5d-Mu** increases by only about 1%, whereas the amplitude increases by ∼40%. The concentration of **2d** does not change, so this means the increase in the amplitude of the radical signals comes from the rate constant for Mu addition to **2d** increasing with temperature. The increased rate is due to two factors that we cannot disentangle with our current data: (1) the reaction is an activated process that can be described by the Arrhenius equation and (2) the viscosity of THF decreases with increasing temperature, which will increase the diffusion rates of both Mu and **2d**.

**Figure 13:**
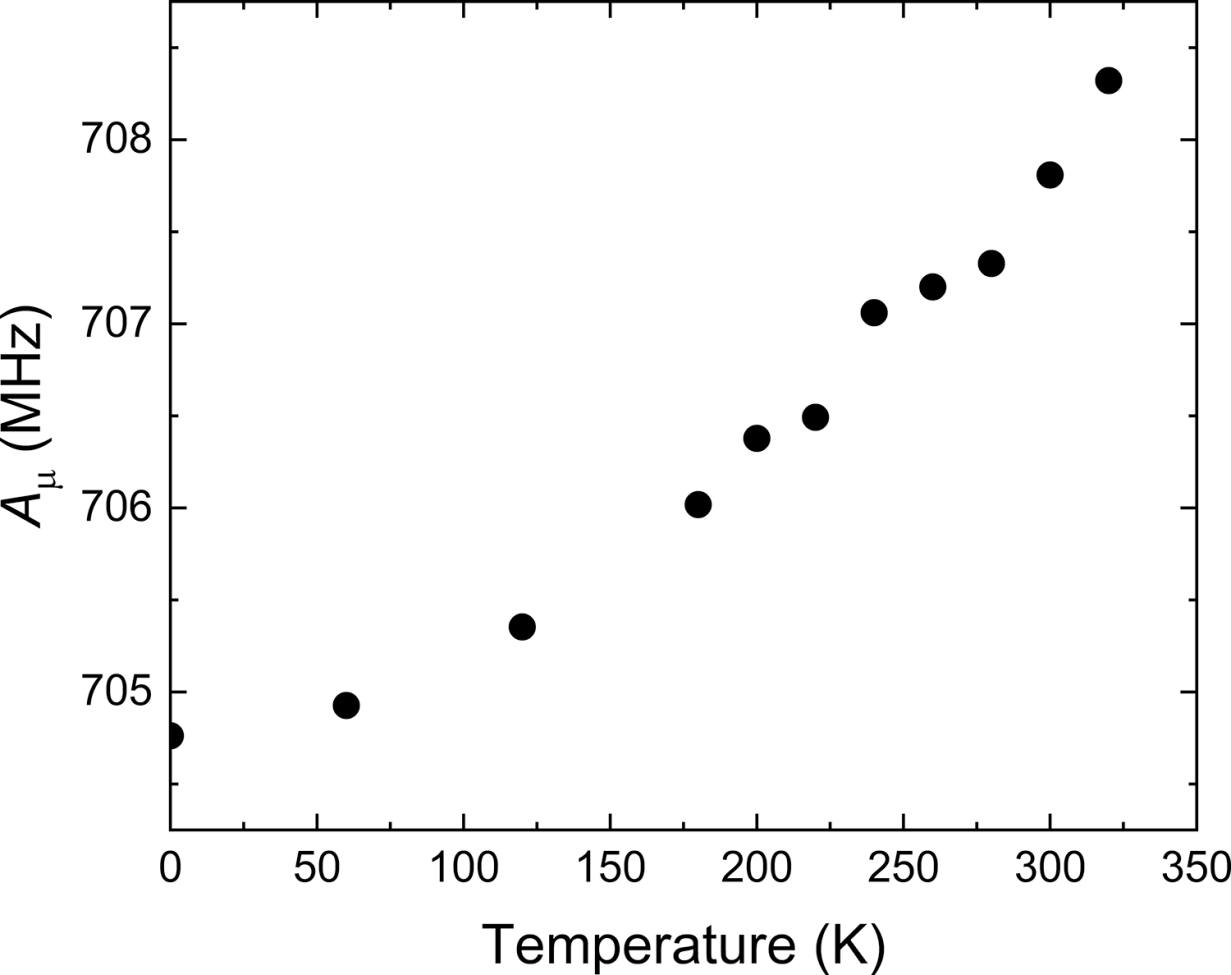
Temperature dependence of the calculated vibrationally averaged muon hfc of **trans-5d-Mu** calculated using the property Fermi keyword and anharmonic vibrational frequencies in Gaussian 16.

The muon hfc of **trans-5d-Mu** increases roughly linearly with temperature with d*A*_μ_/d*T* ∼ 0.12 MHz K^–1^ (Figure 12b). DFT calculations that included vibrational averaging were performed to explain the temperature dependence of *A*_μ_ and to determine what information this provides about dynamics of the radical. The phenyl ring at the ortho position of **trans-5a-Mu** in the preferred orientation of the −N=C–Mu group is being pushed slightly out of the plane of the phenyl ring. There are several low-energy vibrational modes that bring the −N=C-Mu group out of the plane of the phenyl ring, where the *A*_μ_ value is larger. The calculated, vibrationally averaged *A*_μ_ of **trans-5a-Mu** gets larger with increasing temperature (Figure 13), but d*A*_μ_/d*T* around room temperature is ∼0.025 MHz K^–1^ or only ∼20% that of the experimental value. We suggest that the vibrational averaging does not properly account for the large amplitude and low-energy vibrations that cause the observed temperature dependence of *A*_μ_. We also considered the thermal population of the cis structure, but the high energy of this structure means that this would contribute ∼0.007 MHz K^–1^ to d*A*_μ_/d*T*. The increase of *A*_μ_ indicates that the amplitude of torsional motion of the −N=C–Mu group, which is the first step in the intramolecular cyclization, is increasing with increasing temperature.

The TF-μSR spectra were fit in the time domain to determine the relaxation rate of the radical signal, λ. Spin relaxation contributes to the width of the radical precession signal, and this can be affected by motion of the radical, but this typically leads to narrower lines at higher temperatures due to more rapid reorientation. The observed broadening of the TF-μSR signal is most likely due to chemical reactions on the μs time scale. There is only one muoniated radical in the sample at a time, so the broadening cannot be due to radical–radical reactions. This suggests that the change of λ with temperature is due to the isonitrile insertion process. Analyzing the temperature dependence of λ provides information about the kinetics of the intramolecular cyclization.^40−42^ Based on the data at the five temperatures from 262 to 312 K in Figure 12c, a preliminary analysis of the reaction kinetics was attempted. The line drawn in Figure 12c is the least-squares fit to
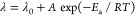
5The fit gives an activation energy *E*_a_ of 5.1 ± 3.7 kcal/mol. The estimated *E*_a_ includes the large deviation due to the lack of a wide enough temperature range but would indicate comparable reaction kinetics with the muonium addition to diallylether, causing the radical rearrangement upon cyclization (4.0 ± 0.5 kcal/mol).^40−42^ The estimated *E*_a_ is about a factor of two smaller than the calculated *E*_a_ for **TS**_**5a-6a_Mu**_, which is ∼9.0 kcal/mol. Further measurements over a wider temperature range and with better statistics will be performed to determine this value more accurately.

## Conclusions

In this paper, we developed useful difluoromethylborates **1** for radical isonitrile insertion of 2-isocyano-1,1′-biphenyls **2** providing 6-(difluoromethyl)phenanthridines **3**. Tuning the aryl substituent in **1** was effective in improving the yield of **3**. The diethylaminophenyl-substituted derivative **1K** was employed for the subsequent synthetic study because of its stability, whereas other promising electron-rich difluoromethylborates are hygroscopic and unstable. The reaction conditions using the chemical oxidation conditions were relatively simple and mild. Thus, using an equimolar amount of difluoromethylborate **1** can be an alternative strategy for the synthesis of 6-(difluoromethyl)phenanthridines although the synthetic procedures should be updated to improve the isolated yields of **3**. The radical isonitrile insertion pathways were studied by using DFT calculations. As for the aminophenyl-substituted borates such as **1J** and **1K**, detailed analytical studies on the oxidation process generating difluoromethyl radical will be of further interest. Besides the chemical oxidation condition, it will be possible to use photocatalytic and electrochemical methods to generate synthetically useful difluoromethyl radical from difluoromethylborates. Attempted organic syntheses based on radical reactions with **1** under various oxidative conditions are underway.

So far, experimental information about the structure and dynamics of imidoyl radicals has been limited. In this study, we examined 2-isocyano-4′-methoxy-1,1′-biphenyl (**2d**) using TF-μSR. Muonium preferentially added to the carbon atom of the isonitrile functional group to give the imidoyl radical **trans-5d-Mu** with minor amounts of addition to the ortho and para carbons of the aromatic ring, generating cyclohexadienyl radicals. **Trans-5d-Mu** is a proposed intermediate in the cyclization reaction. The temperature dependence of the muoniated imidoyl radical signal in the TF-μSR spectra indicated intramolecular radical cyclization on the μs time scale with the rate increasing with temperature. Further μSR studies on isonitriles are planned to understand the effect of different substituents on the cyclization process. This should be important for developing synthetic technologies.

## Data Availability

The data underlying this study are available in the published article and its Supporting Information.

## Supporting Information Available

Experimental procedures and analytical data (1H, ^13^C, ^11^B, ^19^F NMR, HRMS for new compounds), electrochemical analyses, DFT calculations, and principles of TF-μSR (PDF)
