# Supplementary material for: Difluoromethylborates and Muonium for the Study of Isonitrile Insertion Affording Phenanthridines via Imidoyl Radicals
Source: J Org Chem. 2023 Jun 23;88(13):8042–54. doi: 10.1021/acs.joc.3c00056 (PMC10337039; doi:10.1021/acs.joc.3c00056)
Supplement: Supplementary file 1 — jo3c00056_si_001.pdf [file jo3c00056_si_001.pdf]

Supporting Information

Difluoromethylborates and Muonium for Study of Isonitrile Insertion Affording Phenanthridines via Imidoyl Radicals

Kakeru Konagaya,[a] Yu-En Huang,[a] Kazuki Iwami,[a] Tetsuya Fujino,[a] Rikutaro Abe,[a] Reuben Parchment-Morrison,[b,c] Kenji M. Kojima,[c,d] Iain McKenzie,[c,e,f]* and Shigekazu Ito[a]*

[a] Department of Applied Chemistry, School of Materials and Chemical Technology, Tokyo Institute of Technology, 2-12-1-H-113 Ookayama, Meguro-ku, Tokyo 152-8552, Japan

[b] School of Physics and Astronomy, Cardiff University, Queen's Building, The Parade, Cardiff CF24 3AA, United Kingdom

[c] Centre for Molecular and Materials Science, TRIUMF, 4004 Wesbrook Mall, Vancouver, BC, V6T 2A3, Canada

[d] Stewart Blusson Quantum Matter Institute, 2355 East Mall, Vancouver, BC, V6T 1Z4, Canada

[e] Department of Chemistry, Simon Fraser University, 8888 University Drive, Burnaby, BC, V5A 1S6, Canada

[f] Department of Physics and Astronomy, University of Waterloo, 200 University Avenue West, Waterloo, ON, N2L 3G1, Canada

Table of Contents

Experimental section ·· 3

Screening of reaction conditions for isonitrile insertion with CF_2H radical ······· 20

Electrochemical properties and efficiency of isonitrile insertion ···················· 22

Theoretical studies for oxidation of aminophenyl-substituted difluoromethylborates ·· 24

Theoretical studies for CF_2H radical addition to 2-isocyano-1,1'-biphenyl ········ 25

A brief introduction to transverse-field muon spin rotation (TF-μSR) ············· 26

DFT calculation data ·· 28

Additional DFT calculations·· 68

Voltammograms of electrochemical measurements ································· 90

Copies of NMR spectra ··· 98

Experimental Section

General

All experiments were carried out under inert atmosphere (nitrogen or argon) unless otherwise noted. ^{1}H NMR, ^{13}C{^{1}H} NMR, and ^{19}F NMR spectra were measured on a Bruker Avance Neo (400 MHz) spectrometer. Chemical shifts of ^{1}H NMR were expressed in parts per million downfield from $CHCl_3$ as an internal standard (δ = 7.26) in $CDCl_3$. Chemical shifts of ^{13}C NMR were expressed in parts per million relative to the central line of the triplet (δ = 77.10) for $CDCl_3$, central line of the septet (δ = 39.52) for acetone-d_6, or central line of the septet (δ = 39.52) for CD_2Cl_2. Chemical shifts of ^{19}F NMR were expressed in parts per million downfield from benzotrifluoride as an internal standard (δ = –63.24) in $CDCl_3$. Important NMR data were tabulated in following order: multiplicity (s: singlet, d: doublet, t: triplet, q: quartet, m: multiplet, br: broad) and coupling constant *J* (Hz). Melting points were recorded on an As One ATM-02. Mass spectra were measured on a JEOL JMS-T100LC spectrometer. Analytical thin layer chromatography (TLC) was performed on a glass plate pre-coated with silica gel (Merck Kieselgel 60 F_{254}, layer thickness 0.25 mm). Visualization was accomplished by UV light (254 nm) and anisaldehyde, Column chromatography was performed on KANTO Silica Gel 60N (spherical, neutral). Electrochemical analyses were measured on a BAS Electrochemical Analyzer Model 620Ds. All experiments were carried out under inert atmosphere (N_2 or argon) unless otherwise noted.

Synthesis of aryl boronic acid pinacol ester: Typical procedure

Method A: From aryl boronic acid

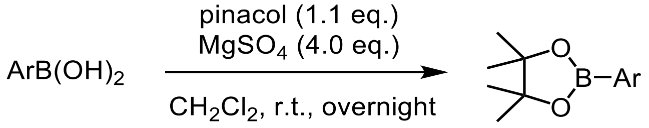

1'

Aryl boronic acid (2 mmol), pinacol (260 mg, 2.2 mmol) and $MgSO_4$ (962 mg, 8 mmol) were dissolved in 4 mL CH_2Cl_2 and the mixture was stirred overnight at room temperature. The precipitation was filtered off, and the solvent was removed in vacuo. Purification by silica-gel column chromatography (hexane/AcOEt 80:1 ~ 20:1) gave the title compound. **1B'**, **1C'**, **1E'-I'**, **1P'** and **1Q'** were synthesized according to this procedure. When the Ar group was 2,6-dimethylphenyl leading to **1D'**, toluene was used as solvent and the mixture was refluxed at 120 °C by using an oil bath.

Method B: Miyaura borylation

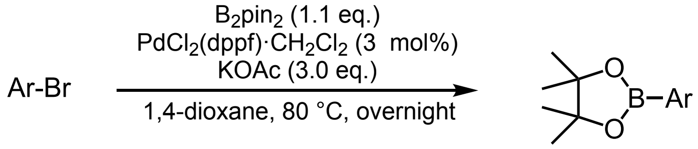

1'

Aryl bromide (2.2 mmol), B_2pin_2 (559 mg, 2.2 mmol), $PdCl_2(dppf)\cdot CH_2Cl_2$ (16 mg, 0.02 mmol) and KOAc (589 mg, 6 mmol) were dissolved in 4 mL 1,4-dioxane. The mixture was refluxed overnight at 80 °C by using an oil bath. The precipitation was filtered off, and the solvent was removed in vacuo. Purification by silica-gel column chromatography (hexane/AcOEt 80:1 ~ 20:1) gave the title compound. **1L'**, **1N'**, **1O'** and **1S'** were synthesized according to this procedure.

Method C: From trimethyl borate

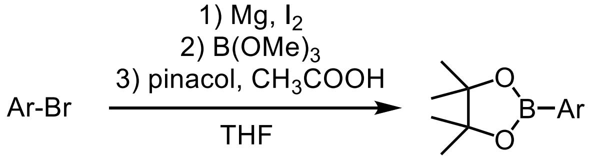

1'

Magnesium turnings (267 mg, 11 mmol) was dried in vacuo using heatgun, and added THF 4 mL. Aryl bromide (10 mmol) dissolved in THF 10 mL was added and iodine was added. After stirring for 40 min at room temperature, THF 30 mL was added and the

suspension was cooled to –78 °C. Trimethyl borate (1.7 mL, 15 mmol) was added and the mixture was stirred overnight at room temperature. Then pinacol (1300 mg, 11 mmol) dissolved in THF 8 mL was added. After stirring for 15 min, acetic acid (6 mL) was added and the mixture was stirred for 4 h. After all volatiles were removed in vacuo, the residue was dissolved in CH_2Cl_2 and wash with H_2O. The organic layer was dried over anhydrous Na_2SO_4 and filtered. After the solvent was removed in vacuo, purification by silica-gel column chromatography (hexane/AcOEt 80:1 ~ 20:1) gave the title compound. **1J’**, **1K’**, **1M’** and **1T’** were synthesized according to this procedure.

Preparation of 4-bromo-*N*,*N*-diethylaniline

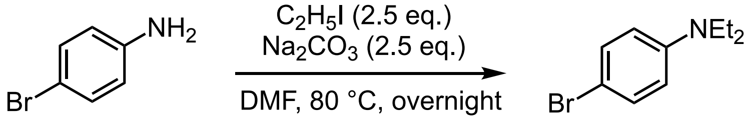

4-Bromoaniline (3441 mg, 20 mmol) and Na_2CO_3 (5300 mg, 50 mmol) were dissolved in 20 mL DMF. Then C_2H_5I (4.06 mL, 50 mmol) was added to the solution and the mixture was refluxed overnight at 80 °C by using an oil bath. Water was added to the reaction mixture and the aqueous phase was extracted with CH_2Cl_2 three times. The organic layer was dried over anhydrous Na_2SO_4 and filtered. After all volatiles were removed in vacuo, the resulting suspension was poured on to short column to remove DMF, and the solvent was removed in vacuo. Purification by silica-gel column chromatography (hexane/EtOAc = 100/1) gave the title compound as a colorless solid (4058 mg, 89%).

^{1}H NMR ($CDCl_3$, 400 MHz) δ 7.26 (d, $^2J_{HH}$ = 9.2 Hz, 2H), 6.54 (d, $^2J_{HH}$ = 9.2 Hz, 2H), 3.31 (q, $^2J_{HH}$ = 7.1 Hz, 4H), 1.14 (t, $^2J_{HH}$ = 7.0 Hz, 6H).

Preparation of N,N-diethyl-4-(4,4,5,5-tetramethyl-1,3,2-dioxaborolan-2-yl)aniline (1K’)

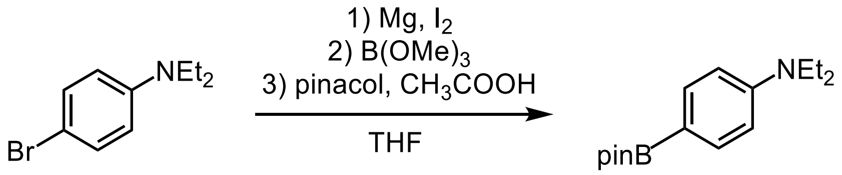

Magnesium turnings (462 mg, 19 mmol) was dried in vacuo with a heat gun, and added THF (6 mL). 4-Bromo-*N*,*N*-diethylaniline (4061 mg, 17.8 mmol) dissolved in THF (18 mL) was added and iodine was added. After stirring for 40 min at room temperature, THF (45 mL) was added and the suspension was cooled to –78 °C. Trimethyl borate (3 mL,

26.7 mmol) was added and the mixture was stirred overnight at room temperature. Then pinacol (2103 mg, 17.8 mmol) dissolved in THF (12 mL) was added. After stirring for 15 min, acetic acid (9 mL) was added and the mixture was stirred for 4 h. After all volatiles were removed in vacuo, the residue was dissolved in CH_2Cl_2 and wash with H_2O. The organic layer was dried over anhydrous Na_2SO_4 and filtered. After the solvent was removed in vacuo, purification by silica-gel column chromatography (hexane/EtOAc = 100/1 to 40/1) gave the title compound as a colorless solid (3888 mg, 79%).
^{1}H NMR ($CDCl_3$, 400 MHz) δ 7.66 (d, $^2J_{HH}$ = 8.8 Hz, 2H), 6.64 (d, $^2J_{HH}$ = 8.8 Hz, 2H), 3.38 (q, $^2J_{HH}$ = 7.1 Hz, 4H), 1.31 (s, 12H), 1.16 (t, $^2J_{HH}$ = 7.0 Hz, 6H).

Synthesis of difluoromethylborate (1): Typical procedure
18-Crown-6 ether (264 mg, 1.0 mmol) and solid potassium *tert*-butoxide (112 mg, 1.0 mmol) were dissolved in 2 mL of THF and cooled to –50 °C. Then (difluoromethyl)trimethylsilane (280 µL, 2.0 mmol) was added and the mixture was stirred for 20 min. Arylboronic acid pinacol ester (1.0 mmol) dissolved in 0.5 mL of THF was added and the solution was stirred at 0 °C for 1 h. The solution was poured into 20 mL hexane and stirred at room temperature for 5 min. The suspension was filtered and washed with hexane (20 mL x 2) twice. The residue was dried in vacuo and purified by recrystallization to afford difluoromethylborate **1** as a solid compound.

The hygroscopic difluoroborates **1M**, **1O**, and **1R-T** were qualitatively characterized by ^{19}F NMR.

2-(Difluoromethyl)-4,4,5,5-tetramethyl-2-phenyl-1,3,2-dioxaborolan-2-uide 18-crown-6-ether complex (1A)[ref]

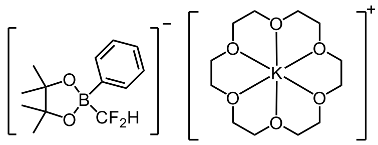

Recrystallization from toluene to afforded title compound as a colorless solid (452 mg, 82%).
^{1}H NMR ($CDCl_3$, 400 MHz) δ 7.67-7.69 (m, 2H), 7.09-7.13 (m, 2H), 6.97-7.01 (m, 1H), 5.68 (t, $^2J_{HF}$ = 50.6 Hz, 1H), 3.55 (s, 24H), 1.19 (s, 6H), 0.98 (s, 6H); ^{19}F NMR ($CDCl_3$, 376 MHz) δ -132.0 (d, $^2J_{FH}$ = 50.6 Hz, 2F); ^{11}B NMR ($CDCl_3$, 128 MHz) δ 2.89 (brs); ^{13}C{^{1}H} NMR ($CDCl_3$, 101 MHz) δ 132.5, 125.8, 123.6, 78.1, 69.8, 26.4, 26.3. (Spectra of CF_2H and C_{ipso} were silent.) HRMS (ESI$^-$-TOF) m/z: [borate]$^-$ calcd for $C_{13}H_{18}BF_2O_2$ 255.1368, found: 255.1276. HRMS (ESI$^+$-TOF) m/z: [18-C-6K]$^+$ calcd for $C_{12}H_{24}O_6K$

303.1210, found 303.1176.

[ref] Yokawa, A.; Ito, S. *Chem. Asian J.* **2020**, *15*, 3432-3436.

2-(3,5-Bis(trifluoromethyl)phenyl)-2-(difluoromethyl)-4,4,5,5-tetramethyl-1,3,2-dioxaborolan-2-uide 18-crown-6-ether complex (1B)

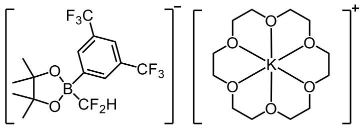

Recrystallization from toluene to afforded title compound as a colorless solid (375 mg, 54%).

^{1}H NMR (CDCl$_3$, 400 MHz) δ 8.17 (s, 1H), 7.50 (s, 1H), 5.65 (t, $^{2}J_{HF}$ = 50.4 Hz, 1H), 3.49 (s, 24H), 1.19 (s, 6H), 0.93 (s, 6H); ^{19}F NMR (CDCl$_3$, 376 MHz) δ -61.8 (s, 6F), -132.4 (m, 2F); ^{11}B NMR (CDCl$_3$, 128 MHz) δ 2.59 (brs); ^{13}C{^{1}H} NMR (CDCl$_3$, 101 MHz) δ 132.6, 129.1, 128.3, 127.6 (q, J_{CF} = 31.2 Hz), 123.7, 120.9, 117.6, 78.5, 69.7, 26.2, 26.0. (Spectra of CF$_2$H and CF$_3$ were silent.) HRMS (ESI$^-$-TOF) m/z: [borate]$^-$ calcd for $C_{15}H_{16}BF_8O_2$ 391.1116, found 391.1158.

2-(Difluoromethyl)-2-(3,5-dimethylphenyl)-4,4,5,5-tetramethyl-1,3,2-dioxaborolan-2-uide 18-crown-6-ether complex (1C)

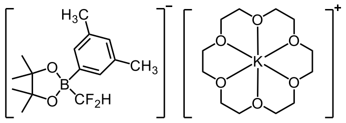

Recrystallization from toluene to afforded title compound as a colorless solid (141 mg, 24%).

^{1}H NMR (CDCl$_3$, 400 MHz) δ 7.31 (s, 2H), 6.65 (s, 1H), 5.64 (t, $^{2}J_{HF}$ = 50.4 Hz, 1H), 3.56 (s, 24H), 2.23 (s, 6H), 1.19 (s, 6H), 0.99 (s, 6H); ^{19}F NMR (CDCl$_3$, 376 MHz) δ -132.4 (d, $^{2}J_{FH}$ = 48.9 Hz, 2F); ^{11}B NMR (CDCl$_3$, 128 MHz) δ 2.89 (brs); ^{13}C{^{1}H} NMR (CDCl$_3$, 101 MHz) δ 134.0, 130.4, 125.5, 78.0, 69.7, 26.4, 21.6. (Spectra of CF$_2$H and C_{ipso} were silent.) HRMS (ESI$^-$-TOF) m/z: [borate]$^-$ calcd for $C_{15}H_{22}BF_2O_2$ 283.1681, found 283.1562.

2-(Difluoromethyl)-2-(2,6-dimethylphenyl)-4,4,5,5-tetramethyl-1,3,2-dioxaborolan-2-uide 18-crown-6-ether complex (1D)

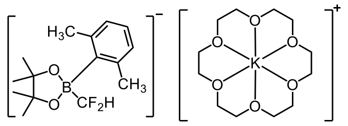

Recrystallization from CH_2Cl_2 and hexane to afforded title compound as a colorless solid (223 mg, 38%).

^{1}H NMR ($CDCl_3$, 400 MHz) δ 6.73–6.81 (m, 3H), 5.74 (t, $^2J_{HF}$ = 50.4 Hz, 1H), 3.51 (s, 24H), 2.61 (s, 6H), 1.18 (s, 6H), 0.95 (s, 6H); ^{19}F NMR ($CDCl_3$, 376 MHz) δ -132.0 (d, $^2J_{FH}$ = 48.9 Hz, 2F); ^{11}B NMR ($CDCl_3$, 128 MHz) δ 4.31 (brs); ^{13}C{^{1}H} NMR ($CDCl_3$, 101 MHz) δ 142.8, 126.7, 123.6, 77.6, 69.9, 26.2, 25.0. (Spectra of CF_2H and C_{ipso} were silent.) HRMS (ESI$^-$-TOF) m/z: [borate]$^-$ calcd for $C_{15}H_{22}BF_2O_2$ 283.1681, found 283.1591.

2-(4-(*tert*-Butyl)phenyl)-2-(difluoromethyl)-4,4,5,5-tetramethyl-1,3,2-dioxaborolan-2-uide 18-crown-6-ether complex (1E)

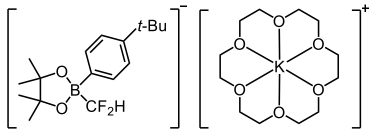

Recrystallization from toluene to afforded title compound as a colorless solid (289 mg, 47%).

^{1}H NMR ($CDCl_3$, 400 MHz) δ 7.57 (d, $^3J_{HH}$ = 8.1 Hz, 2H), 7.12 (d, $^3J_{HH}$ = 8.2 Hz, 2H), 5.69 (t, $^2J_{HF}$ = 50.6 Hz, 1H), 3.54 (s, 24H), 1.26 (s, 9H), 1.18 (s, 6H), 0.99 (s, 6H); ^{19}F NMR ($CDCl_3$, 376 MHz) δ -132.0 (d, $^2J_{FH}$ = 50.6 Hz, 2F); ^{11}B NMR ($CDCl_3$, 128 MHz) δ 3.12 (brs); ^{13}C{^{1}H} NMR ($CDCl_3$, 101 MHz) δ 145.4, 132.1, 122.6, 78.1, 69.8, 34.1, 31.8, 26.5. (Spectra of CF_2H and C_{ipso} were silent.) HRMS (ESI$^-$-TOF) m/z: [borate]$^-$ calcd for $C_{17}H_{26}BF_2O_2$ 311.1994, found 311.1895.

2-(Difluoromethyl)-4,4,5,5-tetramethyl-2-(naphthalen-2-yl)-1,3,2-dioxaborolan-2-uide 18-crown-6-ether complex (1F)

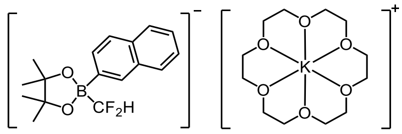

Recrystallization from toluene to afforded title compound as a colorless solid (365 mg, 60%).

^{1}H NMR ($CDCl_3$, 400 MHz) δ 8.14 (s, 1H), 7.95 (d, $^3J_{HH}$ = 8.1 Hz, 1H), 7.73 (d, $^3J_{HH}$ = 8.0 Hz, 1H), 7.67 (d, $^3J_{HH}$ = 7.9 Hz, 1H), 7.58 (d, $^3J_{HH}$ = 8.1 Hz, 1H), 7.18-7.27 (m, 2H), 5.76 (t, $^2J_{HF}$ = 51.0 Hz, 1H), 3.37 (s, 24H), 1.23 (s, 6H), 0.99 (s, 6H); ^{19}F NMR ($CDCl_3$,

376 MHz) δ -131.4 (d, $^2J_{FH}$ = 50.5 Hz, 2F); ^{11}B NMR ($CDCl_3$, 128 MHz) δ 3.01 (brs); $^{13}C\{^1H\}$ NMR ($CDCl_3$, 101 MHz) δ 133.6, 132.7, 132.1, 130.6, 127.8, 127.2, 124.0, 123.5, 122.8, 78.2, 69.7, 26.4, 26.2. (Spectra of CF_2H and C_{ipso} was silent.) HRMS (ESI$^-$-TOF) m/z: [borate]$^-$ calcd for $C_{17}H_{20}BF_2O_2$ 305.1524, found 305.1438.

2-(Difluoromethyl)-4,4,5,5-tetramethyl-2-(naphthalen-1-yl)-1,3,2-dioxaborolan-2-uide 18-crown-6-ether complex (1G)

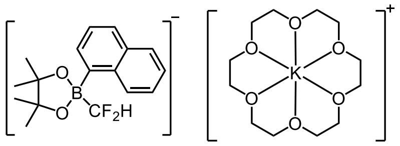

Recrystallization from toluene to afforded title compound as a colorless solid (402 mg, 66%).

^{1}H NMR ($CDCl_3$, 400 MHz) δ 9.02 (d, $^3J_{HH}$ = 8.2 Hz, 1H), 7.93 (d, $^3J_{HH}$ = 6.8 Hz, 1H), 7.62 (d, $^3J_{HH}$ = 8.6 Hz, 1H), 7.47 (d, $^3J_{HH}$ = 8.0 Hz, 1H), 7.21-7.32 (m, 3H), 5.82 (t, $^2J_{HF}$ = 50.8 Hz, 1H), 3.33 (s, 24H), 1.25 (s, 6H), 0.96 (s, 6H); ^{19}F NMR ($CDCl_3$, 376 MHz) δ -131.1 (d, $^2J_{FH}$ = 48.8 Hz, 2F); ^{11}B NMR ($CDCl_3$, 128 MHz) δ 3.81 (brs); $^{13}C\{^1H\}$ NMR ($CDCl_3$, 101 MHz) δ 138.1, 133.4, 132.6, 130.4, 127.1, 125.1, 123.9, 123.3, 122.4, 78.1, 69.7, 31.8, 26.5, 25.8. (Spectra of CF_2H and C_{ipso} were silent.) HRMS (ESI$^-$-TOF) m/z: [borate]$^-$ calcd for $C_{17}H_{20}BF_2O_2$ 305.1524, found: 305.1454.

2-(Difluoromethyl)-4,4,5,5-tetramethyl-2-(thiophen-3-yl)-1,3,2-dioxaborolan-2-uide 18-crown-6-ether complex (1H)

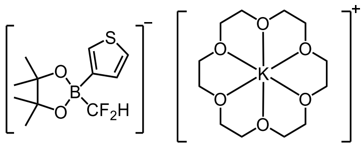

Recrystallization from toluene to afforded title compound as a colorless solid (282 mg, 50%).

^{1}H NMR ($CDCl_3$, 400 MHz) δ 7.35 (d, $^3J_{HH}$ = 4.6 Hz, 1H), 7.22-7.23 (m, 1H), 7.04-7.06 (m, 1H), 5.66 (t, $^2J_{HF}$ = 50.8 Hz, 1H), 3.55 (s, 24H), 1.16 (s, 6H), 1.03 (s, 6H); ^{19}F NMR ($CDCl_3$, 376 MHz) δ -132.1 (d, $^2J_{FH}$ = 50.2 Hz, 2F); ^{11}B NMR ($CDCl_3$, 128 MHz) δ 2.61 (brs); $^{13}C\{^1H\}$ NMR ($CDCl_3$, 101 MHz) δ 133.6, 123.8, 120.8, 78.1, 69.8, 26.5, 26.3. (Spectra of CF_2H and C_{ipso} were silent.) HRMS (ESI$^-$-TOF) m/z: [borate]$^-$ calcd for $C_{11}H_{16}BF_2O_2S$ 261.0932, found 261.0859.

2-(Difluoromethyl)-4,4,5,5-tetramethyl-2-(thiophen-2-yl)-1,3,2-dioxaborolan-2-uide 18-crown-6-ether complex (1I)

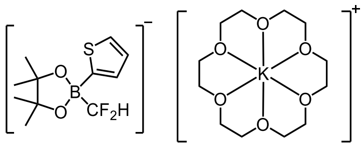

Recrystallization from toluene to afforded title compound as a colorless solid (265 mg, 47%).

^{1}H NMR ($CDCl_3$, 400 MHz) δ 7.14-7.16 (m, 2H), 6.97-6.99 (m, 1H), 5.67 (t, $^2J_{HF}$ = 50.6 Hz, 1H), 3.56 (s, 24H), 1.17 (s, 6H), 1.08 (s, 6H); ^{19}F NMR ($CDCl_3$, 376 MHz) δ -132.4 (d, $^2J_{FH}$ = 49.0 Hz, 2F); ^{11}B NMR ($CDCl_3$, 128 MHz) δ 2.61 (brs); ^{13}C{^{1}H} NMR ($CDCl_3$, 101 MHz) δ 127.0, 126.5, 123.3, 78.4, 69.8, 26.5, 26.4. (Spectra of CF_2H and C_{ipso} were silent.) HRMS (ESI$^-$-TOF) m/z: [borate]$^-$ calcd for $C_{11}H_{16}BF_2O_2S$ 261.0932, found 261.0861.

2-(Difluoromethyl)-2-(4-(dimethylamino)phenyl)-4,4,5,5-tetramethyl-1,3,2-dioxaborolan-2-uide 18-crown-6-ether complex (1J)

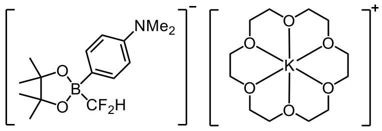

Crude product was washed with Et_2O to afforded title compound as a colorless solid (354 mg, 59%).

^{1}H NMR ($CDCl_3$, 400 MHz) δ 7.55 (d, $^3J_{HH}$ = 8.4 Hz, 2H), 6.65 (d, $^3J_{HH}$ = 8.5 Hz, 2H), 5.67 (t, $^2J_{HF}$ = 50.8 Hz, 1H), 3.55 (s, 24H), 2.84 (s, 6H), 1.18 (s, 6H), 0.99 (s, 6H); ^{19}F NMR ($CDCl_3$, 376 MHz) δ -131.9 (d, $^2J_{FH}$ = 50.9 Hz, 2F); ^{11}B NMR ($CDCl_3$, 128 MHz) δ 3.14 (brs); ^{13}C{^{1}H} NMR ($CDCl_3$, 101 MHz) δ 148.0, 133.0, 112.3, 78.0, 69.8, 41.5, 26.5, 26.4. (Spectra of CF_2H and C_{ipso} were silent.) HRMS (ESI$^-$-TOF) m/z: [borate]$^-$ calcd for $C_{15}H_{23}BF_2NO_2$ 298.1790, found 298.1704.

2-(4-(Diethylamino)phenyl)-2-(difluoromethyl)-4,4,5,5-tetramethyl-1,3,2-dioxaborolan-2-uide 18-crown-6-ether complex (1K)

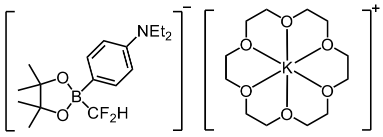

Crude product was washed with Et_2O to afforded title compound as a colorless solid (327 mg, 52%).

^{1}H NMR ($CDCl_3$, 400 MHz) δ 7.50 (d, $^3J_{HH}$ = 8.4 Hz, 2H), 6.58 (d, $^3J_{HH}$ = 8.5 Hz, 2H), 5.67 (t, $^2J_{HF}$ = 50.7 Hz, 1H), 3.56 (s, 24H), 3.25 (q, $^3J_{HH}$ = 7.0 Hz, 6H), 1.17 (s, 6H), 1.09

(t, $^3J_{HH}$ = 7.0 Hz, 6H), 1.00 (s, 6H); ^{19}F NMR ($CDCl_3$, 376 MHz) δ -131.8 (d, $^2J_{FH}$ = 50.6 Hz, 2F); ^{11}B NMR ($CDCl_3$, 128 MHz) δ 3.12 (brs); ^{13}C{^{1}H} NMR ($CDCl_3$, 101 MHz) δ 145.3, 133.2, 112.0, 78.0, 69.8, 44.5, 26.5, 13.0. (Spectra of CF_2H and C_{ipso} were silent.) HRMS (ESI$^-$-TOF) m/z: [borate]$^-$ calcd for $C_{17}H_{27}BF_2NO_2$ 326.2103, found 326.2020.

2-(4-(1*H*-Pyrrol-1-yl)phenyl)-2-(difluoromethyl)-4,4,5,5-tetramethyl-1,3,2-dioxaborolan-2-uide 18-crown-6-ether complex (1L)

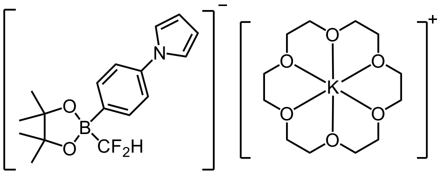

Recrystallization from toluene to afforded title compound as a colorless solid (262 mg, 42%).

^{1}H NMR ($CDCl_3$, 400 MHz) δ 7.73 (d, $^3J_{HH}$ = 8.2 Hz, 2H), 7.16 (d, $^3J_{HH}$ = 8.3 Hz, 2H), 7.05 (t, $^3J_{HH}$ = 2.2 Hz, 2H), 6.25 (t, $^3J_{HH}$ = 2.2 Hz, 2H), 5.69 (t, $^2J_{HF}$ = 50.8 Hz, 1H), 3.53 (s, 24H), 1.19 (s, 6H), 1.00 (s, 6H); ^{19}F NMR ($CDCl_3$, 376 MHz) δ -131.8 (d, $^2J_{FH}$ = 51.2 Hz, 2F); ^{11}B NMR ($CDCl_3$, 128 MHz) δ 2.89 (brs); ^{13}C{^{1}H} NMR ($CDCl_3$, 101 MHz) δ 137.2, 133.4, 119.3, 118.3, 108.9, 78.2, 69.8, 26.4, 26.3. (Spectra of CF_2H and C_{ipso} were silent.) HRMS (ESI$^-$-TOF) m/z: [borate]$^-$ calcd for $C_{17}H_{21}BF_2NO_2$ 320.1633, found 320.1538.

2-(Difluoromethyl)-4,4,5,5-tetramethyl-2-(4-(piperidin-1-yl)phenyl)-1,3,2-dioxaborolan-2-uide 18-crown-6-ether complex (1N)

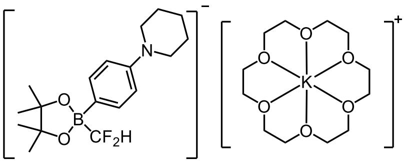

Crude product was washed with Et_2O to afforded title compound as a colorless solid (284 mg, 44%).

^{1}H NMR ($CDCl_3$, 400 MHz) δ 7.54 (d, $^3J_{HH}$ = 8.3 Hz, 2H), 6.76 (d, $^3J_{HH}$ = 8.4 Hz, 2H), 5.67 (t, $^2J_{HF}$ = 50.7 Hz, 1H), 3.57 (s, 24H), 3.03-3.05 (m, 4H), 1.66-1.68 (m , 4H), 1.49-1.52 (m, 2H), 1.17 (s, 6H), 0.98 (s, 6H); ^{19}F NMR ($CDCl_3$, 376 MHz) δ -131.9 (d, $^2J_{FH}$ = 50.3 Hz, 2F); ^{11}B NMR ($CDCl_3$, 128 MHz) δ 3.30 (brs); ^{13}C{^{1}H} NMR ($CDCl_3$, 101 MHz) δ 149.2, 132.9, 115.3, 78.0, 69.9, 51.7, 31.6, 26.5, 24.5, 22.7, 14.1. (Spectra of CF_2H and C_{ipso} were silent.) HRMS (ESI$^-$-TOF) m/z: [borate]$^-$ calcd for $C_{18}H_{27}BF_2NO_2$ 338.2103, found 338.1988.

2-(Difluoromethyl)-2-(4-methoxyphenyl)-4,4,5,5-tetramethyl-1,3,2-dioxaborolan-2-uide 18-crown-6-ether complex (1P)

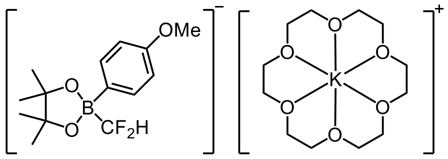

Recrystallization from toluene to afforded title compound as a colorless solid (253 mg, 43%).

^{1}H NMR (CDCl$_3$, 400 MHz) δ 7.59 (d, $^{3}J_{HH}$ = 8.4 Hz, 2H), 6.72 (d, $^{3}J_{HH}$ = 8.5 Hz, 2H), 5.66 (t, $^{2}J_{HF}$ = 50.7 Hz, 1H), 3.75 (s, 3H), 3.55 (s, 24H), 1.18 (s, 6H), 0.99 (s, 6H); ^{19}F NMR (CDCl$_3$, 376 MHz) δ -131.9 (d, $^{2}J_{FH}$ = 49.7 Hz, 2F); ^{11}B NMR (CDCl$_3$, 128 MHz) δ 2.80 (brs); ^{13}C{^{1}H} NMR (CDCl$_3$, 101 MHz) δ 156.6, 132.3, 111.5, 78.1, 69.8, 54.8, 26.4, 26.3. (Spectra of CF$_2$H and C$_{ipso}$ were silent.) m/z: [borate]$^-$ HRMS (ESI$^-$-TOF) m/z: [borate]$^-$ calcd for $C_{14}H_{20}BF_2O_3$ 285.1474, found 285.1363.

2-(Difluoromethyl)-2-(3,5-dimethoxyphenyl)-4,4,5,5-tetramethyl-1,3,2-dioxaborolan-2-uide 18-crown-6-ether complex (1Q)

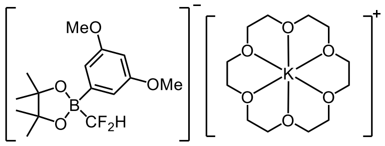

Recrystallization from toluene to afforded title compound as a colorless solid (309 mg, 50%).

^{1}H NMR (CDCl$_3$, 400 MHz) δ 6.92 (d, $^{4}J_{HH}$ = 2.4 Hz, 2H), 6.15 (t, $^{4}J_{HH}$ = 2.4 Hz, 1H), 5.66 (t, $^{2}J_{HF}$ = 50.7 Hz, 1H), 3.75 (s, 6H), 3.54 (s, 24H), 1.18 (s, 6H), 0.99 (s, 6H); ^{19}F NMR (CDCl$_3$, 376 MHz) δ -132.1 (d, $^{2}J_{FH}$ = 50.3 Hz, 2F); ^{11}B NMR (CDCl$_3$, 128 MHz) δ 2.90 (brs); ^{13}C{^{1}H} NMR (CDCl$_3$, 101 MHz) δ 158.8, 109.6, 97.2, 78.1, 69.8, 55.0, 26.4, 26.2. (Spectra of CF$_2$H and C$_{ipso}$ were silent.) HRMS (ESI$^-$-TOF) m/z: [borate]$^-$ calcd for $C_{15}H_{22}BF_2O_4$ 315.1579, found 315.1458.

Synthesis of 2-amino-1,1'-biphenyl derivatives: Typical procedure

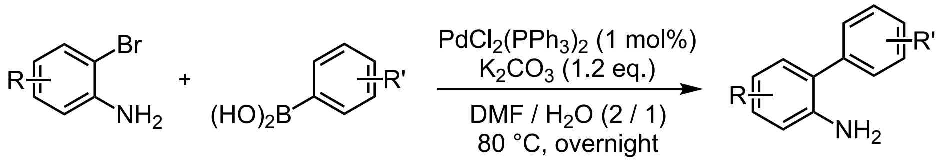

A 2-bromoaniline derivative (5 mmol), aryl boronic acid (6 mmol), PdCl$_2$(PPh$_3$)$_2$ (35 mg,

0.05 mmol) and K_2CO_3 (829 mg, 6 mmol) were dissolved in DMF (5 mL) and H_2O (2.5 mL). Then the mixture was refluxed overnight at 80 °C by using an oil bath. The resulting suspension was extracted with CH_2Cl_2 three times. The organic layer was dried over anhydrous Na_2SO_4 and filtered. After volatiles were removed in vacuo, the resulting suspension was poured into short column to remove DMF, and the solvent was removed in vacuo. Purification by silica-gel column chromatography (hexane/AcOEt 20:1 ~ 5:1) gave the title compound.

Synthesis of 2-isocyano-1,1'-biphenyl derivatives (2): Typical procedure

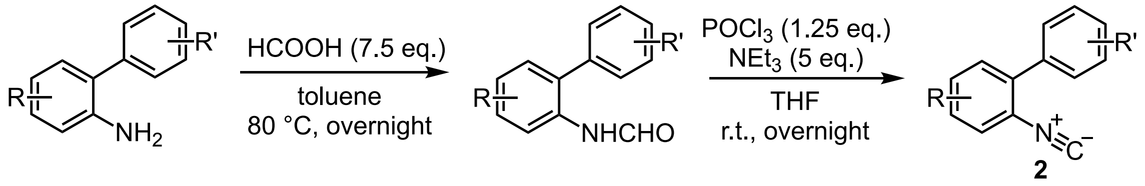

A 2-amino-1,1'-biphenyl derivative was dissolved in toluene (4 mL) and formic acid (560 µL, 15 mmol) was added. The mixture was refluxed overnight at 80 °C by using an oil bath. After all volatiles were removed in vacuo, THF (4mL) and triethylamine (1.4 mL, 10 mmol) were added. Then $POCl_3$ (248 µL, 2.5 mmol) dissolved in THF (1 mL) was added dropwise in 1 h at 0 °C. The mixture was stirred for 2 h at 0 °C and stirred overnight at room temperature. The reaction was quenched by saturated aqueous $NaHCO_3$ and extracted with ethyl acetate three times. The organic layer was dried over anhydrous Na_2SO_4 and filtered. After the solvent was removed in vacuo, purification by silica-gel column chromatography (hexane/AcOEt = 80:1) gave the title compound.

Synthesis of 6-difluoromethylphenanthridines (3): Typical procedure for determining the ^{19}F NMR-based yield

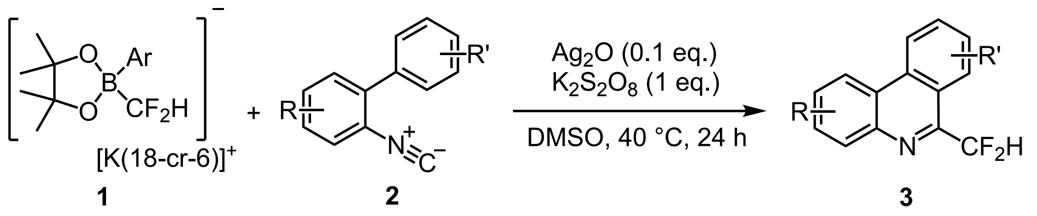

Difluoroborate **1K** (62.9 mg, 0.10 mmol), 2-isocyanobiphenyl **2** (0.10 mmol), silver oxide (2.3 mg, 0.010 mmol) and potassium persulfate (27.0 mg, 0.10 mmol) in DMSO (1.0 mL) was stirred for 24 h at 40 °C by using an oil bath. The resulting suspension was poured on to short column to remove DMSO, and the solvent was removed in vacuo. Purification by silica-gel column chromatography (hexane/AcOEt) gave the title compound **3** sometimes containing the starting material **2**. The yields were determined based on ^{19}F

NMR using 0.10 mmol of benzenetrifluoride (BTF).

Isolation and characterization of 6-(difluoromethyl)pheantridines (3)

6-(Difluoromethyl)phenanthridine (3a)[3_ref. 1-4]

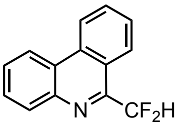

(^{19}F NMR yield: 53%)

Reaction of **1K** (0.20 mmol), **2a** (0.20 mmol), Ag_2O (0.020 mmol) and $K_2S_2O_8$ (0.20 mmol) in DMSO (2.0 mL) followed by silica-gel column chromatography (hexane/AcOEt = 80:1) afforded 16.5 mg of **3a** (38% yield) as a yellow solid.

^{1}H NMR ($CDCl_3$, 400 MHz) δ 8.71 (d, *J* = 8.2 Hz, 1H), 8.64–8.57 (m, 2H), 8.23–8.19 (m, 1H), 7.93 (t, *J* = 7.7 Hz, 1H), 7.82–7.74 (m, 3H), 7.03 (t, *J* = 54.4 Hz, 1H); ^{13}C{^{1}H} NMR ($CDCl_3$, 101 MHz) δ 151.7 (t, *J* = 26.6 Hz), 142.8, 134.1, 131.5, 130.9, 129.4, 129.0, 128.1, 126.78 (t, *J* = 4.3 Hz), 125.3, 122.7, 122.5, 118.7 (t, *J* = 244.4 Hz).

6-(Difluoromethyl)-8-methylphenanthridine (3b)[3_ref. 2,3]

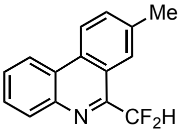

(^{19}F NMR yield: 53%)

Reaction of **1K** (0.30 mmol), **2b** (0.30 mmol), Ag_2O (0.030 mmol) and $K_2S_2O_8$ (0.30 mmol) in DMSO (3.0 mL) followed by silica-gel column chromatography (hexane/AcOEt = 80:1) and recrystallization from hexane afforded 12.1 mg of **3b** (17%) as a yellow solid.

^{1}H NMR ($CDCl_3$, 400 MHz) δ 8.58−8.55 (m, 2H), 8.34 (s, 1H), 8.21−8.15 (m, 1H), 7.77 −7.71 (m, 3H), 7.02 (t, *J* = 54.4 Hz, 1H), 2.63 (s, 3H); ^{13}C{^{1}H} NMR ($CDCl_3$, 101 MHz) δ 151.4 (t, *J* = 26.4 Hz), 142.5, 138.3, 133.4, 132.0, 130.9, 129.0, 128.9, 126.1 (t, *J* = 4.1 Hz), 125.4, 122.9, 122.6, 122.3, 118.8 (t, *J* = 244.4 Hz), 22.2.

8-(*tert*-Butyl)-6-(difluoromethyl)phenanthridine (3c)[3_ref. 2,3]

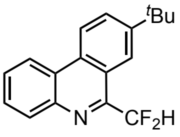

(^{19}F NMR yield: 52%)

Reaction of **1K** (0.20 mmol), **2c** (0.20 mmol), Ag_2O (0.020 mmol) and $K_2S_2O_8$ (0.20 mmol) in DMSO (2.0 mL) followed by silica-gel column chromatography (hexane/AcOEt = 80:1) afforded 10.0 mg of **3c** (18%, purity 97%) as a yellow solid

including 0.5% of **2c**.

^{1}H NMR ($CDCl_3$, 400 MHz) δ 8.63 (d, J = 8.8 Hz, 1H), 8.60–8.54 (m, 2H), 8.21–8.17 (m, 1H), 8.00 (dd, J = 8.8, 2.0 Hz, 1H), 7.75 (tt, J = 6.9, 4.7 Hz, 2H), 7.07 (t, J = 54.4 Hz, 1H), 1.49 (s, 9H); ^{13}C{^{1}H} NMR ($CDCl_3$, 101 MHz) δ 151.8 (t, J = 26.4 Hz), 151.3, 142.6, 132.0, 130.8, 130.0, 129.0, 128.8, 125.3, 122.8 (t, J = 2.1 Hz), 122.5, 122.4, 122.2 (t, J = 4.4 Hz), 118.9 (t, J = 244.4 Hz), 35.6, 31.6.

6-(Difluoromethyl)-8-methoxyphenanthridine (3d)[3_ref. 2,3]

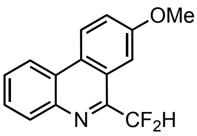

(^{19}F NMR yield: 57%)

Reaction of **1K** (0.10 mmol), **2d** (0.10 mmol), Ag_2O (0.010 mmol) and $K_2S_2O_8$ (0.10 mmol) in DMSO (1.0 mL) followed by silica-gel column chromatography (hexane/AcOEt = 80:1) afforded 8.5 mg of **3d** (35%, purity 97%) as a colorless solid including 1% of **2d**.

^{1}H NMR ($CDCl_3$, 400 MHz) δ 8.61 (d, J = 9.1 Hz, 1H), 8.55–8.51 (m, 1H), 8.19–8.15 (m, 1H), 7.91 (d, J = 2.3 Hz, 1H), 7.76–7.70 (m, 2H), 7.55 (dd, J = 9.1, 2.6 Hz, 1H), 7.01 (t, J = 54.4 Hz, 1H), 4.02 (s, 3H); ^{13}C{^{1}H} NMR ($CDCl_3$, 101 MHz) δ 159.2, 150.7 (t, J = 26.5 Hz), 142.0, 130.9, 129.0, 128.6, 128.4, 125.5, 124.3, 124.12 (t, J = 2.0 Hz), 122.7, 122.0, 119.0 (t, J = 244.3 Hz), 106.2 (t, J = 4.7 Hz), 55.9.

6-(Difluoromethyl)-N,N-dimethylphenanthridin-8-amine (3e)

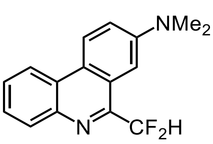

(^{19}F NMR yield: 47%)

Reaction of **1K** (0.1 mmol), **2e** (0.1 mmol), Ag_2O (0.1 mmol) and $K_2S_2O_8$ (0.1 mmol) in DMSO (1 mL) followed by silica-gel column chromatography (hexane/AcOEt = 80:1) and recrystallization from hexane afforded 3.0 mg of **3e** (11%) as a yellow solid.

^{1}H NMR ($CDCl_3$, 400 MHz) δ 8.52 (d, $^{3}J_{HH}$ = 9.2 Hz, 1H), 8.45 (d, $^{3}J_{HH}$ = 8.7 Hz, 1H), 8.11 (d, $^{3}J_{HH}$ = 8.0 Hz, 1H), 7.62-7.67 (m, 3H), 7.44 (dd, $^{4}J_{HH}$ = 2.7 Hz, $^{3}J_{HH}$ = 9.2 Hz, 1H), 6.98 (t, $^{2}J_{HF}$ = 54.5 Hz, 1H), 3.15 (s, 6H); ^{19}F NMR ($CDCl_3$, 376 MHz) δ -112.6 (d, $^{2}J_{FH}$ = 55.9 Hz, 2F); ^{13}C{^{1}H} NMR ($CDCl_3$, 101 MHz) δ 150.4 (t, J = 26.2 Hz), 141.0, 149.5, 130.4, 128.4, 127.0, 125.6, 124.5, 124.1, 123.3, 121.2, 118.82 (t, J = 249.5 MHz), 118.76, 105.2 (t, J = 4.4 Hz), 40.5. HRMS (ESI^{+}-TOF) m/z $[M+H]^{+}$ calcd for $C_{16}H_{15}F_2N_2$ 273.1203, found 273.1229.

8-Chloro-6-(difluoromethyl)phenanthridine (3f)[3_ref. 3]

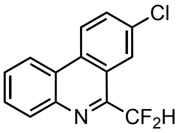

(^{19}F NMR yield: 46%)

Reaction of **1K** (0.10 mmol), **2f** (0.10 mmol), Ag_2O (0.010 mmol) and $K_2S_2O_8$ (0.10 mmol) in DMSO (1.0 mL) followed by silica-gel column chromatography (hexane/AcOEt = 80:1) and recrystallization from hexane afforded 6.9 mg of **3f** (26%, purity 92%) as a colorless solid including 2% of **2f**.

^{1}H NMR ($CDCl_3$, 400 MHz) δ 8.63 (d, J = 8.9 Hz, 1H), 8.58–8.55 (m, 2H), 8.22–8.20 (m, 1H), 7.87 (dd, J = 8.9, 2.1 Hz, 1H), 7.84–7.76 (m, 2H), 6.99 (t, J = 54.3 Hz, 1H); ^{13}C{^{1}H} NMR ($CDCl_3$, 101 MHz) δ 150.7 (t, J = 27.0 Hz), 142.7, 134.3, 132.5, 132.2, 131.1, 129.8, 129.4, 126.1 (t, J = 4.7 Hz), 124.8, 124.5, 123.5 (t, J = 2.1 Hz), 122.4, 118.5 (t, J = 244.4 Hz).

6-(Difluoromethyl)-8-(trifluoromethyl)phenanthridine (3g)[3_ref. 2,3]

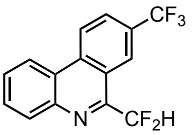

(^{19}F NMR yield: 61%)

Reaction of **1K** (0.15 mmol), **2g** (0.15 mmol), Ag_2O (0.015 mmol) and $K_2S_2O_8$ (0.15 mmol) in DMSO (1.5 mL) followed by silica-gel column chromatography (hexane/AcOEt = 80:1) and recrystallization from hexane afforded 8.1 mg of **3g** (18%, purity 87%) as a colorless solid including 2% of **2g**.

^{1}H NMR ($CDCl_3$, 400 MHz) δ 8.86 (s, 1H), 8.81 (d, J = 8.7 Hz, 1H), 8.64 (dd, J = 7.9, 1.7 Hz, 1H), 8.25 (dd, J = 8.3, 1.6 Hz, 1H), 8.10 (dd, J = 8.8, 1.9 Hz, 1H), 7.90–7.81 (m, 2H), 7.03 (t, J =54.2 Hz, 1H); ^{13}C{^{1}H} NMR ($CDCl_3$, 101 MHz) δ 151.6 (t, J = 27.0 Hz), 143.4, 136.2, 131.2, 130.7, 129.8, 129.6, 127.5 (q, J = 3.2 Hz), 124.4 (q, J = 4.3 Hz), 124.4, 124.1 (q, J =273.6 Hz), 123.9, 122.9, 122.0 (t, J = 2.1 Hz), 118.5 (t, J = 244.5 Hz).

6-(Difluoromethyl)-7,9-bis(trifluoromethyl)phenanthridine (3h)

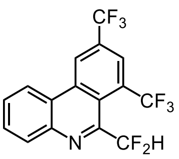

(^{19}F NMR yield: 45%)

Reaction of **1K** (0.1 mmol), **2h** (0.1 mmol), Ag_2O (0.1 mmol) and $K_2S_2O_8$ (0.1 mmol) in DMSO (1 mL) followed by silica-gel column chromatography (hexane/AcOEt = 80:1) and recrystallization from hexane afforded 12.5 mg of **3h** (34%) as a colorless solid.

^{1}H NMR ($CDCl_3$, 400 MHz) δ 9.18 (s, 1H), 8.59 (d, $^{3}J_{HH}$ = 7.9 Hz, 1H), 8.35 (d, $^{3}J_{HH}$ = 9.1 Hz, 1H), 8.29 (s, 1H), 7.81-7.92 (m, 2H), 7.43 (t, $^{2}J_{HF}$ = 53.6 Hz, 1H); ^{19}F NMR ($CDCl_3$, 376 MHz) δ –55.3 (t, $^{2}J_{FF}$ = 7.6 Hz, 3F), –63.0 (s, 3F), –112.8 (dq, $^{2}J_{FF}$ = 7.6 Hz, $^{2}J_{FH}$ = 53.5 Hz, 2F); ^{13}C{^{1}H} NMR ($CDCl_3$, 101 MHz) δ 148.5 (t, J = 20.5 Hz), 142.5, 135.5, 131.5, 131.14, 131.12, 130.2, 127.5 (q, J = 32.6 Hz), 124.7 (q, J = 4.0 Hz), 124.3–124.0 (m), 123.7 (q, J = 274.8 Hz), 123.2, 122.9 (q, J = 274.2 Hz), 122.1, 120.5, 109.4 (tq, J = 10.3 Hz, J = 240.6 Hz); HRMS (ESI^{+}-TOF) m/z [M+H]$^{+}$ calcd for $C_{16}H_8F_8N$ 366.0529, found 366.0577.

6-(Difluoromethyl)-3-methylphenanthridine (3i)[3_ref. 2,3]

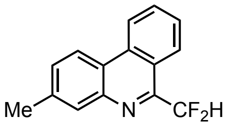

(^{19}F NMR yield: 48%)

Reaction of **1K** (0.20 mmol), **2i** (0.20 mmol), Ag_2O (0.020 mmol) and $K_2S_2O_8$ (0.20 mmol) in DMSO (2.0 mL) followed by silica-gel column chromatography (hexane/AcOEt = 80:1) and recrystallization from hexane afforded 8.1 mg of **3i** (17%, purity 93%) as a yellow solid including 1% of **2i**.

^{1}H NMR ($CDCl_3$, 400 MHz) δ 8.64 (d, J = 8.4 Hz, 1H), 8.56 (d, J = 8.4 Hz, 1H), 8.48 (d, J = 8.4 Hz, 1H), 7.99 (s, 1H), 7.88 (t, J = 7.7 Hz, 1H), 7.72 (t, J = 7.7 Hz, 1H), 7.58 (d, J = 8.4 Hz, 1H), 7.01 (t, J = 54.4 Hz, 1H), 2.61 (s, 3H); ^{13}C{^{1}H} NMR ($CDCl_3$, 101 MHz) δ 151.7 (t, J = 26.4 Hz), 143.0 (t, J = 1.7 Hz), 139.7, 134.2, 131.5, 130.7, 130.4, 127.6, 126.7 (t, J = 4.2 Hz), 123.0, 122.6, 122.5 (t, J = 2.0 Hz), 122.3, 118.8 (t, J = 244.4 Hz), 21.9.

6-(Difluoromethyl)-3-(trifluoromethyl)phenanthridine (3j)[3_ref. 2,3]

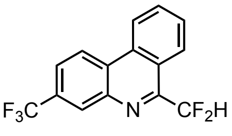

(^{19}F NMR yield: 43%)

Reaction of **1K** (0.20 mmol), **2j** (0.20 mmol), Ag_2O (0.020 mmol) and $K_2S_2O_8$ (0.20 mmol) in DMSO (2.0 mL) followed by silica-gel column chromatography (hexane/AcOEt = 80:1) afforded 20.4 mg of **3j** (34%) as a yellow solid.

^{1}H NMR ($CDCl_3$, 400 MHz) δ 8.73 (d, J = 8.5 Hz, 2H), 8.64 (dd, J =8.5, 1.6 Hz, 1H), 8.51 (s, 1H), 7.99 (t, J = 7.7 Hz, 1H), 7.96 (d, J = 8.3 Hz, 1H), 7.86 (t, J = 7.7 Hz, 1H), 7.02 (t, J = 54.3 Hz, 1H); ^{13}C{^{1}H} NMR ($CDCl_3$, 101 MHz) δ 153.2 (t, J = 26.8 Hz), 142.1, 133.4, 132.2, 131.3 (q, J = 33.3 Hz), 129.3, 128.5 (q, J = 4.4 Hz), 127.6, 127.0 (t, J = 4.4 Hz), 124.8 (q, J = 3.4 Hz), 124.2 (q, J = 273.5 Hz), 123.6, 123.3, 123.1, 118.4 (t,

J = 244.2 Hz).

6-(Difluoromethyl)-3,8-dimethylphenanthridine (3k)

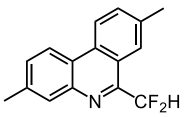

(^{19}F NMR yield: 63%)

Reaction of **1K** (0.20 mmol), **2k** (0.20 mmol), Ag_2O (0.020 mmol) and $K_2S_2O_8$ (0.20 mmol) in DMSO (2.0 mL) followed by silica-gel column chromatography (hexane/AcOEt = 80:1) and recrystallization from hexane afforded 10.8 mg of **3k** (23%, purity 97%) as a yellow solid including 0.7% of **2k**.

^{1}H NMR ($CDCl_3$, 400 MHz) δ 8.53 (d, $^3J_{HH}$ = 8.5 Hz, 1H), 8.44 (d, $^3J_{HH}$ = 8.4 Hz, 1H), 8.32 (s, 1H), 7.97 (s, 1H), 7.71 (dd, $^4J_{HH}$ = 1.5 Hz, $^3J_{HH}$ = 8.5 Hz, 1H), 7.56 (dd, $^4J_{HH}$ = 1.6 Hz, $^3J_{HH}$ = 8.4 Hz, 1H), 6.99 (t, $^2J_{HF}$ = 54.4 Hz, 1H), 2.62 (s, 3H), 2.60 (s, 3H); ^{19}F NMR ($CDCl_3$, 376 MHz) δ -110.7 (dd, $^2J_{FH}$ = 2.3 Hz, $^2J_{FH}$ = 54.2 Hz, 2F); ^{13}C{^{1}H} NMR ($CDCl_3$, 101 MHz) δ 151.0 (t, J = 26.3 Hz), 142.3, 138.8, 137.4, 133.0, 131.8, 130.3, 130.0, 125.7 (t, J = 4.1 Hz), 122.8, 122.3 (d, J = 1.8 Hz), 122.1, 121.7, 118.5 (t, J = 244.3 Hz), 21.9, 21.5. HRMS (ESI$^+$-TOF) m/z [M+H]$^+$ calcd for $C_{16}H_{14}F_2N$ 258.1094, found 258.1160.

6-(Difluoromethyl)-3,8-bis(trifluoromethyl)phenanthridine (3l)

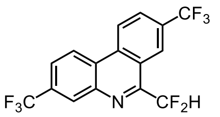

(^{19}F NMR yield: 63%)

Reaction of **1K** (0.10 mmol), **2l** (0.10 mmol), Ag_2O (0.010 mmol) and $K_2S_2O_8$ (0.10 mmol) in DMSO (1.0 mL) followed by silica-gel column chromatography (hexane/AcOEt = 80:1) and recrystallization from hexane afforded 3.7 mg of **3l** (10%) as a colorless solid.

^{1}H NMR ($CDCl_3$, 400 MHz) δ 8.91 (s, 1H), 8.85 (d, $^3J_{HH}$ = 8.8 Hz, 1H), 8.76 (d, $^3J_{HH}$ = 8.6 Hz, 1H), 8.56 (s, 1H), 8.18 (dd, $^4J_{HH}$ = 1.6 Hz, $^3J_{HH}$ = 8.7 Hz, 1H), 8.03 (dd, $^4J_{HH}$ = 1.7 Hz, $^3J_{HH}$ = 8.6 Hz, 1H), 7.04 (t, $^2J_{HF}$ = 54.1 Hz, 1H); ^{19}F NMR ($CDCl_3$, 376 MHz) δ –62.56 (s, 3F), –62.58 (s, 3F), –110.2 (d, $^2J_{FH}$ = 54.0 Hz, 2F); ^{13}C{^{1}H} NMR ($CDCl_3$, 101 MHz) δ 152.7 (t, J = 27.4 Hz), 142.5, 135.2, 132.2 (q, J = 33.5 Hz), 130.9 (q, J = 33.3 Hz), 128.5 (q, J = 4.3 Hz), 127.8 (q, J = 3.4 Hz), 126.3, 125.2 (q, J = 3.5 Hz), 124.3 (q, J = 4.7 Hz), 124.0, 123.7, 123.64 (q, J = 273.9 Hz), 123.56 (q, J = 273.9 Hz), 122.4 (t, J = 2.1 Hz), 117.8 (t, J = 244.9 Hz). HRMS (ESI$^-$-TOF) m/z [M+H]$^-$ calcd for $C_{16}H_6F_8N$ 364.0373, found 364.0370.

References for the reported 6-(difluoromethyl)phenanthridines:

[**3**_ref. 1] Zhang, Z.; Tang, X.; Dolbier, Jr. R. *Org. Lett.* **2015**, *17*, 4401-4403.

[**3**_ref. 2] Rong, J.; Deng, L.; Tan, P.; Ni, C.; Gu, Y.; Hu, J. *Angew. Chem. Int. Ed.* **2016**, *55*, 2743-2747.

[**3**_ref. 3] Qin, W.-B.; Xiong, W.; Li, X.; Chen, J.-Y.; Lin, L.-T.; Wong, H. N. C.; Liu, G.-K. *J. Org. Chem.* **2020**, *85*, 10479-10487.

[**3**_ref. 4] Yang, J.; Zhu, S.; Wang, F.; Qing, F.-L.; Chu, L. *Angew. Chem. Int. Ed.* **2021**, *60*, 4300-4306.

Gram scale reaction of 2-isocyano-4'-methoxy-1,1'-biphenyl (2d) affording 3d

Difluoroborate **1K** (1007 mg, 1.60 mmol), 2-isocyano-4'-methoxy-1,1'-biphenyl (**2d**, 335 mg, 1.60 mmol), silver oxide (37 mg, 0.16 mmol) and potassium persulfate (433 mg, 1.60 mmol) in DMSO (16 mL) was stirred for 24 h at 40 °C by using an oil bath. The resulting suspension was poured on to short column to remove DMSO, and the solvent was removed in vacuo. The reaction mixture was monitored by ^{19}F NMR using benzotrifluoride (BTF) to provide the yield of 52%. Purification by silica-gel column chromatography (hexane/AcOEt 80:1) gave a crude compound (200 mg). Recrystallization from hexane afforded 103 mg of **3d** (25% isolated yield).

Screening of reaction conditions for isonitrile insertion with CF_2H radical

Table S1.

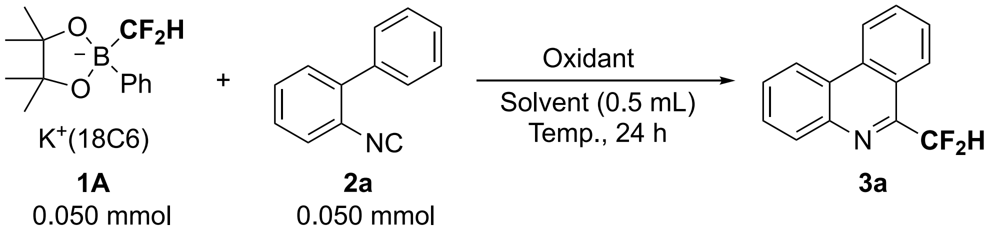

Entry	Oxidant	Solvent	Temp.[a] / °C	Yield / % [b]
1	Ag_2O	CH_2Cl_2 / H_2O (50:1)	50	trace
2	Ag_2O	CH_2Cl_2 / H_2O (50:1)	120	5
3	Ag_2O, $K_2S_2O_8$	DMF	80	11
4	Ag_2O, $K_2S_2O_8$	DMF	50	18
5	Ag_2O, $K_2S_2O_8$	DMF	40	24
6	Ag_2O, $K_2S_2O_8$	DMF	RT	0
	Ag_2O, $K_2S_2O_8$	DMF	40	0
7	$K_2S_2O_8$	DMF / H_2O (1:1)	40	10
8	Ag_2O, $K_2S_2O_8$	DMSO	40	29
9	Ag_2O, $K_2S_2O_8$	DMSO / H_2O (1:1)	40	3
10	Ag_2O, $K_2S_2O_8$	MeCN	40	3
11	Ag_2O, $K_2S_2O_8$	MeCN / H_2O (1:1)	40	20
12	Ag_2O, $K_2S_2O_8$	DMA	40	0
13	Ag_2O, $K_2S_2O_8$	MeOH	40	0
14	Ag_2O, $K_2S_2O_8$	$MeNO_2$	40	0
15	Ag_2O, $K_2S_2O_8$	NMP [c]	40	0
16	Ag_2O, $K_2S_2O_8$	CH_2Cl_2	40	trace
17	Ag_2O, $K_2S_2O_8$	CH_2Cl_2 / H_2O (1:1)	40	6

18	Ag_2O, $K_2S_2O_8$	$CHCl_3$	40	0
19	Ag_2O, $K_2S_2O_8$	Sulfolane [d)]	40	15
20	$AgNO_3$, $K_2S_2O_8$	DMSO	40	19
21	PivOAg, $K_2S_2O_8$	DMSO	40	17
22	Chloranil	DMSO	40	0
23	DDQ	DMSO	40	0
24	TCNQ	DMSO	40	0
25	$Mn(OAc)_3 \cdot 2H_2O$	DMSO	40	0

a) Heating was carried out by using an oil bath.

b) Determined by ^{19}F NMR analysis using BTF as an internal standard.

c) *N*-Methylpyrrolidone.

d)

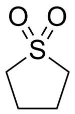

Electrochemical properties and efficiency of isonitrile insertion

Table S2.

Compound	V. *vs.* SCE / V	HOMO / eV [a)]	Yield of **3a** / %
**1A**	0.794	-5.234	29
**1B**	1.618	-6.058	4
**1C**	0.762	-5.202	14
**1D**	0.630	–5.070	22
**1E**	0.762	-5.202	24
**1F**	0.758	-5.198	21
**1G**	0.626	-5.066	29
**1H**	0.862	-5.302	13
**1I**	0.858	-5.298	21
**1J**	0.718 [b)]	-5.158	42
**1K**	0.686 [b)]	-5.126	53
**1L**	0.738	-5.178	32
**1M**	- [c)]	-	34
**1N**	0.682 [b)]	-5.122	27
**1O**	- [c)]	-	18
**1P**	0.754	-5.194	31
**1Q**	0.654	-5.094	44
**1R**	- [c)]	-	(65)
**1S**	- [c)]	-	-

a) SCE = –4.44 eV to vacuum. [Ref)]

b) Lower oxidation potential mainly due to the lone pair was observed. **1J**: 0.190 V (-4.630 eV), **1K**: 0.142 V (-4.582 eV), **1N**: 0.182 V (-4.622 eV).

c) Measurements were impossible due to the hygroscopic character.

Ref) Meng, H.; Zheng, L.; Lovinger, A. J.; Wand, B.-C.; Patten, P. G. V.; Bao, Z. Oligofluorene−thiophene derivatives as high-performance semiconductors for organic thin film transistors, *Chem. Mater.* **2003**, *15*, 1778-1787.

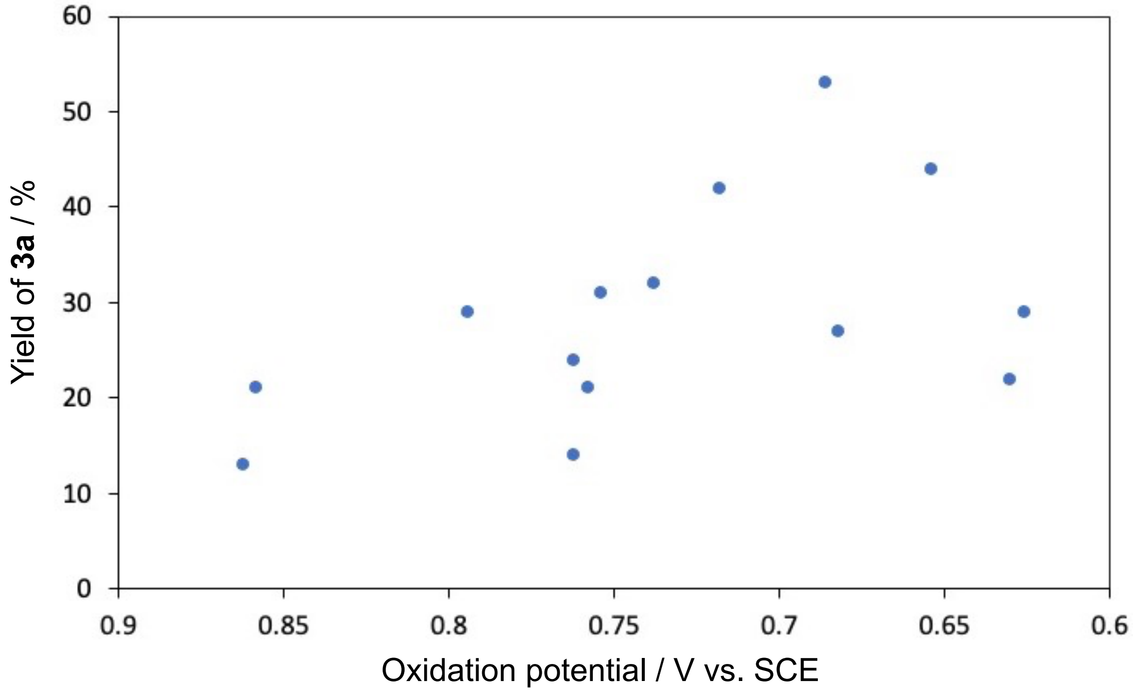

Figure S1. Correlation between the oxidation potentials of **1** and the yield of **3a** in Scheme 2. Data of **1B** is excluded.

Theoretical studies for oxidation of aminophenyl-substituted difluoromethylborates

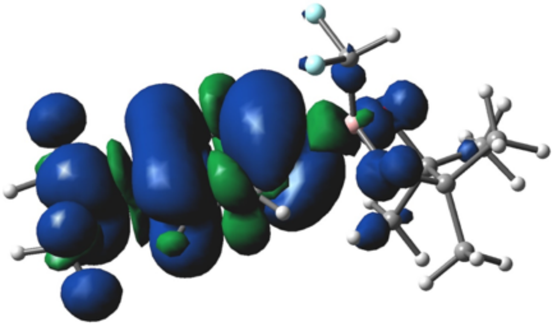

Figure S2. Spin density distribution (iso = 0.0005) over the one-electron oxidized **1J**. [UωB97XD/6-311G(d,p)]. The HF_2C–B distance is 1.629 Å.

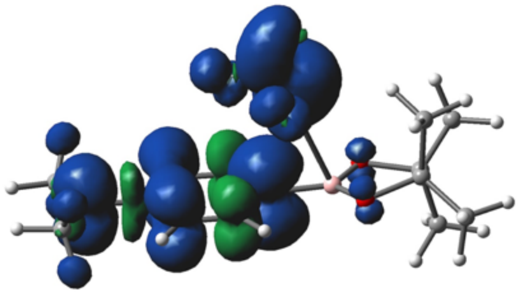

Figure S3. Spin density distribution (iso = 0.002) over the two-electron oxidized **1J** of triplet [UωB97XD/6-311G(d,p)]. The $HF_2C\cdots B$ distance is 3.491Å. The singlet state of 2e-oxidized **1J** caused collapse of the B-Aryl linkage.

Theoretical studies for CF_2H radical addition to 2-isocyano-1,1'-biphenyl

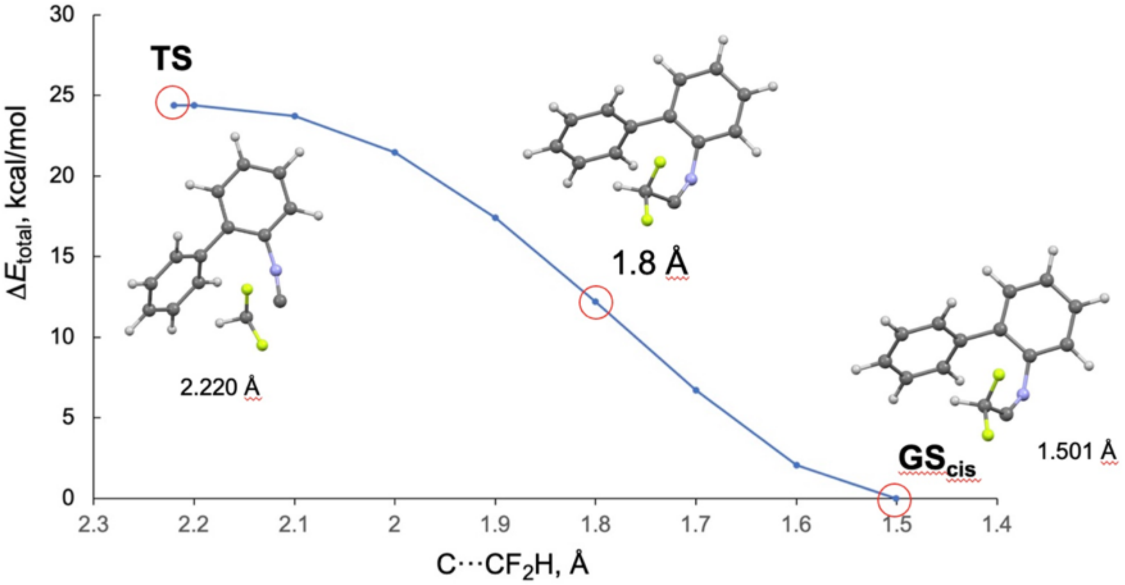

Figure S4. A PES scan for CF_2H radical addition to **2a** [UωB97XD/6-311G(d,p), SMD = DMSO].

A brief introduction to transverse-field muon spin rotation (TF-µSR)

In a TF-µSR experiment, muons from a spin-polarized beam are stopped in a sample and the elapsed time between individual muon stops and the detection of their decay positrons is recorded. A magnetic field is applied to the sample in a direction perpendicular to the beam polarization. Since the probability of detecting a decay positron in a particular direction depends on the orientation of the muon spin at the moment of its decay, the positron count oscillates in time as the muon spins precession past the detector. These oscillations are imposed on the lifetime histogram of the muon (τ_μ = 2.197 microseconds). The time spectrum is Fourier transformed to display a frequency spectrum.
Muons in free radicals are subject to the local field due to the unpaired electron – the hyperfine interaction. At high fields (well satisfied in the experiments reported here) the muon precession frequency in a free radical can have two values, corresponding to the two spin orientations of the unpaired electron:

$$\upsilon_{R1} = \upsilon_{mid} - \frac{1}{2}A_\mu \qquad (S1)$$

$$\upsilon_{R2} = \upsilon_{mid} + \frac{1}{2}A_\mu \qquad (S2)$$

where

$$\upsilon_{mid} = \frac{1}{2}\left[\sqrt{A_\mu^2 + \left(\nu_e + \nu_\mu\right)^2} - \nu_e + \nu_\mu\right] \qquad (S3)$$

ν_e and ν_μ are the electron and muon Larmor frequencies, and the muon hyperfine constant, A_μ is readily found from the difference of the two precession frequencies ν_{R1} and ν_{R2}.

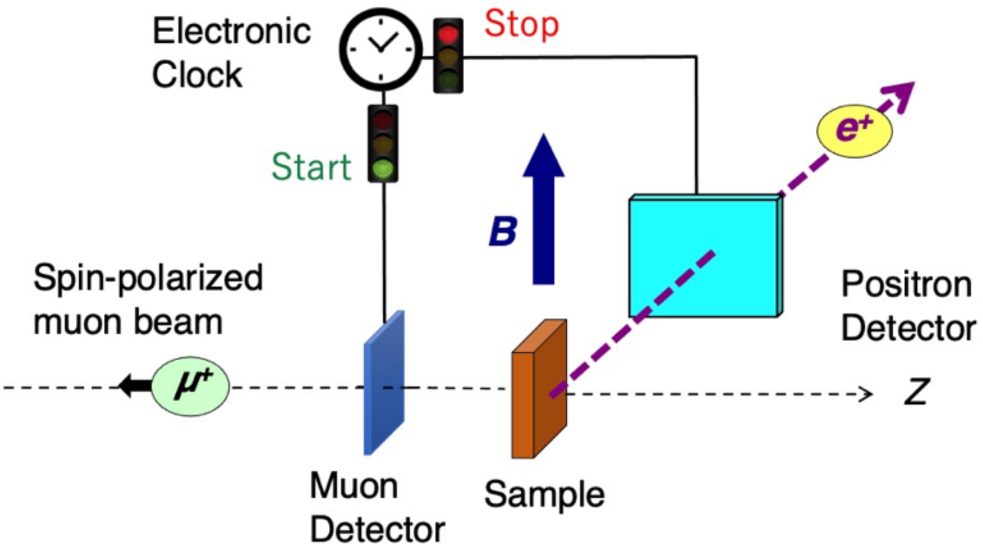

Figure S5. Setup of a TF-μSR experiment. Only one positron detector is shown, and other three detectors are omitted.

DFT calculation data

Density Functional Theory (DFT) calculations were carried out using Gaussian 09 (B.01) and Gaussian 16 (C.01) package.

Reference:

Gaussian 09, Revision B.01, M. J. Frisch, G. W. Trucks, H. B. Schlegel, G. E. Scuseria, M. A. Robb, J. R. Cheeseman, G. Scalmani, V. Barone, B. Mennucci, G. A. Petersson, H. Nakatsuji, M. Caricato, X. Li, H. P. Hratchian, A. F. Izmaylov, J. Bloino, G. Zheng, J. L. Sonnenberg, M. Hada, M. Ehara, K. Toyota, R. Fukuda, J. Hasegawa, M. Ishida, T. Nakajima, Y. Honda, O. Kitao, H. Nakai, T. Vreven, J. A. Montgomery, Jr., J. E. Peralta, F. Ogliaro, M. Bearpark, J. J. Heyd, E. Brothers, K. N. Kudin, V. N. Staroverov, T. Keith, R. Kobayashi, J. Normand, K. Raghavachari, A. Rendell, J. C. Burant, S. S. Iyengar, J. Tomasi, M. Cossi, N. Rega, J. M. Millam, M. Klene, J. E. Knox, J. B. Cross, V. Bakken, C. Adamo, J. Jaramillo, R. Gomperts, R. E. Stratmann, O. Yazyev, A. J. Austin, R. Cammi, C. Pomelli, J. W. Ochterski, R. L. Martin, K. Morokuma, V. G. Zakrzewski, G. A. Voth, P. Salvador, J. J. Dannenberg, S. Dapprich, A. D. Daniels, O. Farkas, J. B. Foresman, J. V. Ortiz, J. Cioslowski, D. J. Fox, Gaussian 09, Revision B.01, Gaussian, Inc., Wallingford CT, 2010.

Gaussian 16, Revision C.01, M. J. Frisch, G. W. Trucks, H. B. Schlegel, G. E. Scuseria, M. A. Robb, J. R. Cheeseman, G. Scalmani, V. Barone, G. A. Petersson, H. Nakatsuji, X. Li, M. Caricato, A. V. Marenich, J. Bloino, B. G. Janesko, R. Gomperts, B. Mennucci, H. P. Hratchian, J. V. Ortiz, A. F. Izmaylov, J. L. Sonnenberg, D. Williams-Young, F. Ding, F. Lipparini, F. Egidi, J. Goings, B. Peng, A. Petrone, T. Henderson, D. Ranasinghe, V. G. Zakrzewski, J. Gao, N. Rega, G. Zheng, W. Liang, M. Hada, M. Ehara, K. Toyota, R. Fukuda, J. Hasegawa, M. Ishida, T. Nakajima, Y. Honda, O. Kitao, H. Nakai, T. Vreven, K. Throssell, J. A. Montgomery, Jr., J. E. Peralta, F. Ogliaro, M. J. Bearpark, J. J. Heyd, E. N. Brothers, K. N. Kudin, V. N. Staroverov, T. A. Keith, R. Kobayashi, J. Normand, K. Raghavachari, A. P. Rendell, J. C. Burant, S. S. Iyengar, J. Tomasi, M. Cossi, J. M. Millam, M. Klene, C. Adamo, R. Cammi, J. W. Ochterski, R. L. Martin, K. Morokuma, O. Farkas, J. B. Foresman, and D. J. Fox, Gaussian, Inc., Wallingford CT, 2016.

[pinB(Ph)CF_2H]$^-$

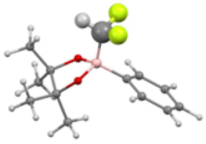

E(RwB97XD) = -881.318117879 A.U.

Center Number	Atomic Number	Atomic Type	Coordinates (Angstroms) X	Y	Z
1	1	0	2.764950	-0.956285	2.667359
2	9	0	-0.736815	2.798806	0.933251
3	8	0	0.855593	0.130125	-1.196186
4	8	0	0.865436	0.216812	1.154250
5	9	0	-0.750107	2.660106	-1.270660
6	1	0	1.749161	-2.142439	1.843366
7	6	0	-0.058588	2.226766	-0.140354
8	1	0	0.910362	2.741953	-0.179490
9	6	0	1.763820	-0.832133	-0.724181
10	1	0	-1.708613	-0.151287	-2.082059
11	5	0	0.046703	0.594728	-0.034720
12	6	0	2.090701	-0.314823	0.720583
13	6	0	3.152026	0.797181	0.700791
14	1	0	4.156788	0.412047	0.491542
15	1	0	2.909072	1.553640	-0.048492
16	1	0	3.164560	1.283863	1.680203
17	6	0	-1.429289	-0.086897	0.040870
18	6	0	-2.026954	-0.415062	1.262282
19	1	0	-1.481320	-0.204783	2.178457
20	6	0	-3.283164	-1.012145	1.329817
21	1	0	-3.720578	-1.254535	2.296340
22	6	0	-3.981985	-1.301574	0.161150
23	1	0	-4.962249	-1.770398	0.207707
24	6	0	2.966401	-0.885674	-1.662777
25	1	0	3.751939	-1.541113	-1.268033
26	1	0	2.656004	-1.277854	-2.636251
27	1	0	3.387078	0.108534	-1.822824
28	6	0	-3.408826	-0.985357	-1.067433
29	1	0	-3.943268	-1.208595	-1.988744
30	6	0	2.537814	-1.403319	1.694402
31	1	0	3.438744	-1.916046	1.336363
32	6	0	1.092069	-2.215007	-0.698161
33	1	0	1.800176	-3.012536	-0.445257
34	1	0	0.265288	-2.240010	0.014061
35	1	0	0.678183	-2.418901	-1.689731
36	6	0	-2.153313	-0.386054	-1.118448

```
Temperature   298.150 Kelvin.  Pressure   1.00000 Atm
 Zero-point correction=                           0.294740 (Hartree/Particle)
 Thermal correction to Energy=                    0.312430
 Thermal correction to Enthalpy=                  0.313374
 Thermal correction to Gibbs Free Energy=         0.249319
 Sum of electronic and zero-point Energies=           -881.023378
 Sum of electronic and thermal Energies=              -881.005688
 Sum of electronic and thermal Enthalpies=            -881.004744
 Sum of electronic and thermal Free Energies=         -881.068799
```

$[pinB(Ph)CF_2H]^{\cdot}$

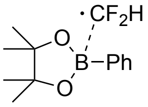

E(UwB97XD) = -881.208457872 A.U.

Center Number	Atomic Number	Atomic Type	Coordinates (Angstroms) X	Y	Z
1	1	0	3.080577	0.309856	2.696845
2	9	0	-1.064769	3.113763	-1.372324
3	8	0	0.965110	-0.817561	-1.023556
4	8	0	0.907641	-0.314280	1.202395
5	9	0	-1.430840	2.760648	0.753887
6	1	0	3.195285	-1.327628	2.047423
7	6	0	-1.393941	2.211428	-0.452829
8	1	0	-2.274191	1.620364	-0.701951
9	6	0	2.344697	-0.668888	-0.619492
10	1	0	-1.433382	-1.602581	-1.932505
11	5	0	0.193052	-0.706902	0.102431
12	6	0	2.212160	0.102366	0.740283
13	6	0	2.157058	1.618474	0.567490
14	1	0	3.129067	2.026313	0.279131
15	1	0	1.422611	1.908792	-0.187280
16	1	0	1.859083	2.074325	1.513727
17	6	0	-1.337940	-0.966486	0.119718
18	6	0	-2.093048	-0.700221	1.267105
19	1	0	-1.590478	-0.354790	2.165142
20	6	0	-3.474354	-0.852212	1.263529
21	1	0	-4.047169	-0.637181	2.159988
22	6	0	-4.122252	-1.274975	0.106834
23	1	0	-5.201527	-1.392285	0.100711
24	6	0	3.096397	0.081500	-1.705644
25	1	0	4.124890	0.284672	-1.392738
26	1	0	3.133840	-0.522473	-2.614992
27	1	0	2.613891	1.027783	-1.949866
28	6	0	-3.386209	-1.550702	-1.041866
29	1	0	-3.890761	-1.883608	-1.943395
30	6	0	3.247986	-0.270388	1.786898
31	1	0	4.257007	-0.050524	1.425818
32	6	0	2.915805	-2.076049	-0.458487
33	1	0	3.978462	-2.051121	-0.204759
34	1	0	2.387402	-2.636467	0.316563
35	1	0	2.800595	-2.615728	-1.400806
36	6	0	-2.005025	-1.396876	-1.032535

Temperature 298.150 Kelvin. Pressure 1.00000 Atm
Zero-point correction= 0.295241 (Hartree/Particle)
Thermal correction to Energy= 0.314461
Thermal correction to Enthalpy= 0.315406
Thermal correction to Gibbs Free Energy= 0.243720
Sum of electronic and zero-point Energies= -880.913217
Sum of electronic and thermal Energies= -880.893996
Sum of electronic and thermal Enthalpies= -880.893052
Sum of electronic and thermal Free Energies= -880.964738

[pinB(4-$NMe_2C_6H_4$)CF_2H]$^-$

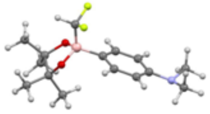

E(RwB97XD) = -1015.30103320 A.U.

Center Number	Atomic Number	Atomic Type	Coordinates (Angstroms) X	Y	Z
1	1	0	-3.389442	-1.620094	-2.593422
2	9	0	-0.796626	2.915018	-1.133325
3	8	0	-1.827652	0.127898	1.182686
4	8	0	-1.805503	0.038504	-1.167857
5	9	0	-0.814126	2.944023	1.075019
6	1	0	-2.137549	-2.483427	-1.692022
7	6	0	-1.365450	2.288624	-0.026566
8	1	0	-2.425039	2.578186	-0.038067
9	6	0	-2.491338	-1.043091	0.780142
10	1	0	0.761969	0.617138	2.080668
11	5	0	-1.110445	0.670028	-0.007167
12	6	0	-2.896076	-0.712676	-0.699977
13	6	0	-4.171306	0.144755	-0.761586
14	1	0	-5.072795	-0.434280	-0.530572
15	1	0	-4.105070	0.980072	-0.061117
16	1	0	-4.265131	0.552128	-1.772146
17	6	0	0.475833	0.315865	-0.018168
18	6	0	1.155475	-0.049210	-1.180239
19	1	0	0.600056	-0.090076	-2.113089
20	6	0	2.509418	-0.373149	-1.191746
21	1	0	2.968884	-0.644348	-2.134879
22	6	0	3.262704	-0.354893	-0.010131
23	6	0	-3.669441	-1.293484	1.719089
24	1	0	-4.286857	-2.128747	1.368261
25	1	0	-3.293094	-1.539167	2.716651
26	1	0	-4.293523	-0.402536	1.805048
27	6	0	2.597498	0.020200	1.165286
28	1	0	3.125542	0.064733	2.110281
29	6	0	-3.077507	-1.938499	-1.593803
30	1	0	-3.842146	-2.615075	-1.193847
31	6	0	-1.528961	-2.240934	0.854462
32	1	0	-2.039832	-3.190031	0.656456
33	1	0	-0.706366	-2.123915	0.146612
34	1	0	-1.099898	-2.276181	1.859693
35	6	0	1.243626	0.341008	1.146887
36	7	0	4.630769	-0.723896	-0.001233
37	6	0	5.413626	-0.293096	1.135709
38	1	0	6.444291	-0.631476	1.007998
39	1	0	5.418066	0.802431	1.269853
40	1	0	5.036911	-0.742301	2.057425
41	6	0	5.328866	-0.655927	-1.266064
42	1	0	4.912666	-1.371159	-1.979566
43	1	0	5.289530	0.345175	-1.728518
44	1	0	6.375874	-0.925464	-1.110887

Temperature 298.150 Kelvin. Pressure 1.00000 Atm
Zero-point correction= 0.367328 (Hartree/Particle)
Thermal correction to Energy= 0.389273
Thermal correction to Enthalpy= 0.390218
Thermal correction to Gibbs Free Energy= 0.316320
Sum of electronic and zero-point Energies= -1014.933705
Sum of electronic and thermal Energies= -1014.911760
Sum of electronic and thermal Enthalpies= -1014.910816
Sum of electronic and thermal Free Energies= -1014.984713

$[\mathrm{pinB(4\text{-}NMe_2C_6H_4)CF_2H}]^{\cdot}$

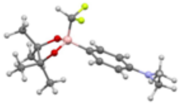

E(UwB97XD) = -1015.15535051 A.U.

Center Number	Atomic Number	Atomic Type	Coordinates (Angstroms) X	Y	Z
1	1	0	-3.468126	-1.511823	-2.595480
2	9	0	-0.516655	2.811632	-1.125225
3	8	0	-1.799457	0.136818	1.180800
4	8	0	-1.786953	0.045152	-1.169613
5	9	0	-0.571045	2.841545	1.083461
6	1	0	-2.282509	-2.463853	-1.695880
7	6	0	-1.202704	2.302597	-0.031120
8	1	0	-2.205936	2.743225	-0.061604
9	6	0	-2.530141	-1.005600	0.775031
10	1	0	0.673176	0.481510	2.106832
11	5	0	-1.135289	0.676468	-0.013957
12	6	0	-2.927668	-0.649404	-0.698880
13	6	0	-4.137340	0.292306	-0.748997
14	1	0	-5.071859	-0.225325	-0.513135
15	1	0	-4.012036	1.118528	-0.046140
16	1	0	-4.212152	0.707940	-1.756511
17	6	0	0.442130	0.263967	-0.018299
18	6	0	1.118540	-0.089267	-1.214366
19	1	0	0.530699	-0.138601	-2.123880
20	6	0	2.460309	-0.335180	-1.238778
21	1	0	2.947723	-0.563967	-2.176845
22	6	0	3.223011	-0.275572	-0.029062
23	6	0	-3.706972	-1.196243	1.724461
24	1	0	-4.370991	-1.994109	1.376501
25	1	0	-3.337276	-1.465488	2.717330
26	1	0	-4.280357	-0.273973	1.818866
27	6	0	2.539609	0.022233	1.192548
28	1	0	3.084999	0.059129	2.125772
29	6	0	-3.181431	-1.853682	-1.597625
30	1	0	-3.990394	-2.477332	-1.203650
31	6	0	-1.617919	-2.239215	0.839742
32	1	0	-2.165072	-3.167239	0.650447
33	1	0	-0.807169	-2.163348	0.109837
34	1	0	-1.176072	-2.293488	1.838039
35	6	0	1.197526	0.263531	1.184014
36	7	0	4.551139	-0.501826	-0.038527
37	6	0	5.331616	-0.427671	1.192296
38	1	0	6.380606	-0.590444	0.958864
39	1	0	5.229714	0.555936	1.655791
40	1	0	5.011911	-1.192444	1.904438
41	6	0	5.245234	-0.835927	-1.277763
42	1	0	4.841845	-1.752465	-1.714288
43	1	0	5.156167	-0.025243	-2.004867
44	1	0	6.299350	-0.992264	-1.063783

Temperature 298.150 Kelvin. Pressure 1.00000 Atm
Zero-point correction= 0.368555 (Hartree/Particle)
Thermal correction to Energy= 0.390992
Thermal correction to Enthalpy= 0.391937
Thermal correction to Gibbs Free Energy= 0.316225
Sum of electronic and zero-point Energies= -1014.786795
Sum of electronic and thermal Energies= -1014.764358
Sum of electronic and thermal Enthalpies= -1014.763414
Sum of electronic and thermal Free Energies= -1014.839125

[pinB(4-NMe$_2$C$_6$H$_4$)CF$_2$H]$^+$ triplet

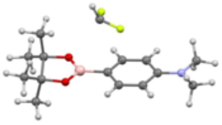

E(UwB97XD) = -1014.93130231 A.U.

Center Number	Atomic Number	Atomic Type	Coordinates (Angstroms) X	Y	Z
1	1	0	4.098987	1.079806	2.362873
2	9	0	-0.022954	2.859987	0.102994
3	8	0	1.868059	-1.116031	-0.777363
4	8	0	1.861451	0.198961	1.094682
5	9	0	-1.244898	1.988173	-1.485506
6	1	0	3.981284	-0.683984	2.401718
7	6	0	-0.056428	2.494883	-1.168940
8	1	0	0.307440	3.248067	-1.866043
9	6	0	3.266872	-1.004003	-0.367860
10	1	0	-0.592208	-1.726328	-1.580390
11	5	0	1.142394	-0.493180	0.180881
12	6	0	3.230788	0.241157	0.587716
13	6	0	3.372641	1.570204	-0.147824
14	1	0	4.394086	1.717705	-0.503677
15	1	0	2.695166	1.621972	-1.004182
16	1	0	3.121397	2.381463	0.537319
17	6	0	-0.425118	-0.554644	0.219114
18	6	0	-1.144326	0.096096	1.243088
19	1	0	-0.595391	0.612257	2.021194
20	6	0	-2.511812	0.107576	1.254292
21	1	0	-3.033732	0.626027	2.045822
22	6	0	-3.240728	-0.546229	0.211217
23	6	0	4.124818	-0.832240	-1.607661
24	1	0	5.165572	-0.652459	-1.325969
25	1	0	4.089442	-1.743057	-2.207971
26	1	0	3.782038	-0.002433	-2.225231
27	6	0	-2.510200	-1.221639	-0.816983
28	1	0	-3.030703	-1.731867	-1.614803
29	6	0	4.189183	0.173203	1.762130
30	1	0	5.219907	0.102827	1.405027
31	6	0	3.612030	-2.305031	0.349279
32	1	0	4.666742	-2.330589	0.630095
33	1	0	3.010073	-2.438494	1.251049
34	1	0	3.414898	-3.142503	-0.322074
35	6	0	-1.142563	-1.219938	-0.796274
36	7	0	-4.584525	-0.523400	0.195302
37	6	0	-5.339748	-1.160078	-0.886497
38	1	0	-6.400167	-0.996164	-0.721654
39	1	0	-5.063489	-0.724297	-1.848140
40	1	0	-5.149067	-2.234994	-0.902042
41	6	0	-5.342698	0.145958	1.255038
42	1	0	-5.100593	-0.288084	2.226502
43	1	0	-5.119744	1.214827	1.264410
44	1	0	-6.403897	0.011469	1.069698

Temperature 298.150 Kelvin. Pressure 1.00000 Atm
Zero-point correction= 0.368248 (Hartree/Particle)
Thermal correction to Energy= 0.391876
Thermal correction to Enthalpy= 0.392820
Thermal correction to Gibbs Free Energy= 0.311115
Sum of electronic and zero-point Energies= -1014.563054
Sum of electronic and thermal Energies= -1014.539426
Sum of electronic and thermal Enthalpies= -1014.538482
Sum of electronic and thermal Free Energies= -1014.620187

Isonitrile (**2a**)

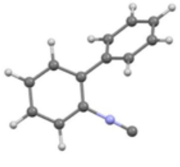

E(RwB97XD) = -555.453103309 A.U.

Center Number	Atomic Number	Atomic Type	Coordinates (Angstroms) X	Y	Z
1	6	0	0.637839	-0.380639	0.076443
2	6	0	1.270725	-1.608743	0.276960
3	6	0	2.654616	-1.716220	0.294502
4	6	0	3.446936	-0.588788	0.108511
5	6	0	2.849815	0.643365	-0.101972
6	6	0	1.460259	0.738236	-0.118587
7	1	0	0.659676	-2.490249	0.434768
8	1	0	3.115902	-2.683218	0.458073
9	1	0	4.527387	-0.667507	0.123748
10	1	0	3.441709	1.535901	-0.263838
11	7	0	0.896375	1.983821	-0.370670
12	1	0	-1.056237	-1.852978	-1.409564
13	6	0	-0.845403	-0.298295	0.060623
14	6	0	-1.529876	0.600285	0.882767
15	6	0	-2.918170	0.644046	0.874176
16	6	0	-3.640741	-0.206186	0.043647
17	6	0	-2.967177	-1.105844	-0.775046
18	6	0	-1.578481	-1.154150	-0.764780
19	1	0	-0.975234	1.259451	1.541352
20	1	0	-3.436968	1.341846	1.522018
21	1	0	-4.724454	-0.168274	0.036034
22	1	0	-3.523669	-1.770849	-1.426117
23	6	0	0.442534	3.034456	-0.594208

Temperature 298.150 Kelvin. Pressure 1.00000 Atm
Zero-point correction= 0.180974 (Hartree/Particle)
Thermal correction to Energy= 0.191656
Thermal correction to Enthalpy= 0.192600
Thermal correction to Gibbs Free Energy= 0.143997
Sum of electronic and zero-point Energies= -555.272130
Sum of electronic and thermal Energies= -555.261448
Sum of electronic and thermal Enthalpies= -555.260504
Sum of electronic and thermal Free Energies= -555.309107

Temperature 313.000 Kelvin. Pressure 1.00000 Atm
Zero-point correction= 0.180974 (Hartree/Particle)
Thermal correction to Energy= 0.192679
Thermal correction to Enthalpy= 0.193671
Thermal correction to Gibbs Free Energy= 0.141550
Sum of electronic and zero-point Energies= -555.272130
Sum of electronic and thermal Energies= -555.260424
Sum of electronic and thermal Enthalpies= -555.259433
Sum of electronic and thermal Free Energies= -555.311553

·CF_2H radical

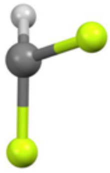

E(UwB97XD) = -238.312139440 A.U

Center Number	Atomic Number	Atomic Type	Coordinates (Angstroms) X	Y	Z
1	6	0	0.029957	0.513575	0.000000
2	1	0	-0.718977	1.304329	0.000000
3	9	0	0.029957	-0.243654	1.093781
4	9	0	0.029957	-0.243654	-1.093781

```
Temperature   298.150 Kelvin.   Pressure   1.00000 Atm
 Zero-point correction=                           0.019239 (Hartree/Particle)
 Thermal correction to Energy=                    0.022337
 Thermal correction to Enthalpy=                  0.023281
 Thermal correction to Gibbs Free Energy=        -0.005784
 Sum of electronic and zero-point Energies=          -238.292900
 Sum of electronic and thermal Energies=             -238.289803
 Sum of electronic and thermal Enthalpies=           -238.288859
 Sum of electronic and thermal Free Energies=        -238.317923

Temperature   313.000 Kelvin.   Pressure   1.00000 Atm
 Zero-point correction=                           0.019239 (Hartree/Particle)
 Thermal correction to Energy=                    0.022529
 Thermal correction to Enthalpy=                  0.023520
 Thermal correction to Gibbs Free Energy=        -0.007237
 Sum of electronic and zero-point Energies=          -238.292900
 Sum of electronic and thermal Energies=             -238.289611
 Sum of electronic and thermal Enthalpies=           -238.288619
 Sum of electronic and thermal Free Energies=        -238.319377
```

Isonitrile + CF_2H TS (**$TS_{2a\text{-}CF2H}$**)

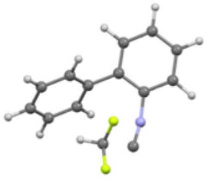

E(UwB97XD) = -793.768484925 A.U

Center Number	Atomic Number	Atomic Type	Coordinates (Angstroms) X	Y	Z
1	6	0	1.255706	-0.652062	0.047060
2	6	0	2.274989	-1.423946	-0.509563
3	6	0	3.574496	-0.942795	-0.606870
4	6	0	3.883934	0.332328	-0.147031
5	6	0	2.891673	1.125265	0.407661
6	6	0	1.590278	0.635735	0.497121
7	1	0	2.039310	-2.421295	-0.863938
8	1	0	4.346993	-1.567769	-1.039820
9	1	0	4.895916	0.712416	-0.221395
10	1	0	3.104702	2.125601	0.764806
11	7	0	0.605570	1.459832	1.016562
12	1	0	-0.281878	-1.504479	-1.992849
13	6	0	-0.133994	-1.171256	0.125129
14	6	0	-0.803535	-1.253358	1.347890
15	6	0	-2.108740	-1.724379	1.404648
16	6	0	-2.761492	-2.116550	0.240009
17	6	0	-2.099053	-2.043463	-0.980325
18	6	0	-0.791037	-1.576177	-1.037870
19	1	0	-0.299530	-0.948646	2.258714
20	1	0	-2.616348	-1.787006	2.360769
21	1	0	-3.782051	-2.480408	0.284738
22	1	0	-2.602335	-2.345832	-1.891985
23	6	0	-0.382244	2.052942	1.268399
24	6	0	-1.852072	1.762177	-0.370307
25	1	0	-2.378763	0.828904	-0.174667
26	9	0	-1.120961	1.739153	-1.487872
27	9	0	-2.645534	2.832282	-0.369462

Temperature 298.150 Kelvin. Pressure 1.00000 Atm
Imaginary = -322.6429 cm-1

Zero-point correction=	0.200780	(Hartree/Particle)
Thermal correction to Energy=	0.215311	
Thermal correction to Enthalpy=	0.216255	
Thermal correction to Gibbs Free Energy=	0.156988	
Sum of electronic and zero-point Energies=	-793.567705	
Sum of electronic and thermal Energies=	-793.553174	
Sum of electronic and thermal Enthalpies=	-793.552230	
Sum of electronic and thermal Free Energies=	-793.611497	

Temperature 313.000 Kelvin. Pressure 1.00000 Atm
Imaginary = -322.6432 cm-1

Zero-point correction=	0.200780	(Hartree/Particle)
Thermal correction to Energy=	0.216618	
Thermal correction to Enthalpy=	0.217609	
Thermal correction to Gibbs Free Energy=	0.154003	
Sum of electronic and zero-point Energies=	-793.567705	
Sum of electronic and thermal Energies=	-793.551867	
Sum of electronic and thermal Enthalpies=	-793.550875	
Sum of electronic and thermal Free Energies=	-793.614482	

Isonitrile + CF_2H cis (**5a-CF_2H_{cis}**)

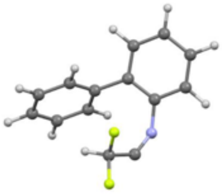

E(UwB97XD) = -793.807371257 A.U

Center Number	Atomic Number	Atomic Type	Coordinates (Angstroms) X	Y	Z
1	6	0	1.048912	-0.779286	0.063963
2	6	0	1.957841	-1.660619	-0.524667
3	6	0	3.312631	-1.366576	-0.595728
4	6	0	3.791521	-0.174482	-0.063842
5	6	0	2.912927	0.715313	0.534595
6	6	0	1.550444	0.427585	0.579316
7	1	0	1.586285	-2.596976	-0.926777
8	1	0	3.994335	-2.072260	-1.056242
9	1	0	4.848500	0.061093	-0.109022
10	1	0	3.265278	1.648384	0.958464
11	7	0	0.709953	1.383866	1.187158
12	1	0	-0.551665	-1.408580	-2.009458
13	6	0	-0.394994	-1.127196	0.115729
14	6	0	-1.089600	-1.151391	1.327711
15	6	0	-2.443548	-1.459879	1.357401
16	6	0	-3.121268	-1.750203	0.177433
17	6	0	-2.435031	-1.740424	-1.031693
18	6	0	-1.079892	-1.432496	-1.062486
19	1	0	-0.567811	-0.927539	2.251844
20	1	0	-2.969691	-1.476305	2.305418
21	1	0	-4.179678	-1.984869	0.201549
22	1	0	-2.955600	-1.965842	-1.955907
23	6	0	-0.318346	1.970497	0.915005
24	6	0	-1.204712	1.993008	-0.295546
25	1	0	-1.985746	1.232413	-0.225135
26	9	0	-0.479750	1.785496	-1.436682
27	9	0	-1.784160	3.216761	-0.421983

```
Temperature   298.150 Kelvin.  Pressure   1.00000 Atm
 Zero-point correction=                           0.204880 (Hartree/Particle)
 Thermal correction to Energy=                    0.218620
 Thermal correction to Enthalpy=                  0.219564
 Thermal correction to Gibbs Free Energy=         0.162628
 Sum of electronic and zero-point Energies=          -793.602492
 Sum of electronic and thermal Energies=             -793.588751
 Sum of electronic and thermal Enthalpies=           -793.587807
 Sum of electronic and thermal Free Energies=        -793.644743

Temperature   313.000 Kelvin.  Pressure   1.00000 Atm
 Zero-point correction=                           0.204879 (Hartree/Particle)
 Thermal correction to Energy=                    0.219906
 Thermal correction to Enthalpy=                  0.220897
 Thermal correction to Gibbs Free Energy=         0.159767
 Sum of electronic and zero-point Energies=          -793.602487
 Sum of electronic and thermal Energies=             -793.587460
 Sum of electronic and thermal Enthalpies=           -793.586469
 Sum of electronic and thermal Free Energies=        -793.647600
```

Isonitrile + CF_2H cis to trans TS (**$TS_{c\text{-}t}$**)

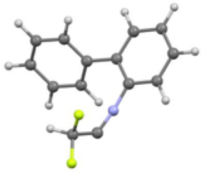

E(UwB97XD) = -793.802563954 A.U

Center Number	Atomic Number	Atomic Type	Coordinates (Angstroms) X	Y	Z
1	6	0	-0.624375	1.265780	-0.084480
2	6	0	-1.122606	2.518807	0.264786
3	6	0	-0.317016	3.651829	0.272123
4	6	0	1.029161	3.545835	-0.071080
5	6	0	1.565038	2.319487	-0.417124
6	6	0	0.748571	1.174651	-0.417954
7	1	0	-2.172633	2.604945	0.522804
8	1	0	-0.739845	4.613015	0.539374
9	1	0	1.665451	4.423503	-0.068549
10	1	0	2.611938	2.214480	-0.675314
11	7	0	1.297263	-0.016020	-0.746582
12	1	0	-2.192604	0.413070	1.935842
13	6	0	-1.504772	0.070529	-0.072294
14	6	0	-1.602924	-0.767518	-1.186853
15	6	0	-2.439661	-1.876025	-1.164634
16	6	0	-3.189618	-2.165331	-0.029158
17	6	0	-3.101800	-1.335546	1.082966
18	6	0	-2.266117	-0.225160	1.061562
19	1	0	-1.033793	-0.543484	-2.082479
20	1	0	-2.509489	-2.513030	-2.039504
21	1	0	-3.841284	-3.031998	-0.012398
22	1	0	-3.680208	-1.554577	1.973830
23	6	0	1.813304	-1.101020	-0.848625
24	6	0	2.268584	-1.957769	0.309568
25	1	0	1.486701	-2.668364	0.582837
26	9	0	2.581279	-1.201623	1.404103
27	9	0	3.395423	-2.638013	-0.031124

Temperature 298.150 Kelvin. Pressure 1.00000 Atm
Imaginary = -69.3582 cm-1
Zero-point correction= 0.203671 (Hartree/Particle)
Thermal correction to Energy= 0.217101
Thermal correction to Enthalpy= 0.218045
Thermal correction to Gibbs Free Energy= 0.160396
Sum of electronic and zero-point Energies= -793.598893
Sum of electronic and thermal Energies= -793.585463
Sum of electronic and thermal Enthalpies= -793.584519
Sum of electronic and thermal Free Energies= -793.642168

Temperature 313.000 Kelvin. Pressure 1.00000 Atm
Imaginary = -69.4122 cm-1
Zero-point correction= 0.203663 (Hartree/Particle)
Thermal correction to Energy= 0.218359
Thermal correction to Enthalpy= 0.219350
Thermal correction to Gibbs Free Energy= 0.157279
Sum of electronic and zero-point Energies= -793.598897
Sum of electronic and thermal Energies= -793.584201
Sum of electronic and thermal Enthalpies= -793.583210
Sum of electronic and thermal Free Energies= -793.645281

Isonitrile + CF_2H stable (**5a-CF_2H**)

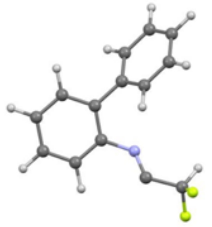

E(UwB97XD) = -793.810479791 A.U

Center Number	Atomic Number	Atomic Type	Coordinates (Angstroms) X	Y	Z
1	6	0	-1.115708	1.000762	-0.071916
2	6	0	-2.008154	2.065022	-0.222748
3	6	0	-1.564168	3.379280	-0.258906
4	6	0	-0.206095	3.658190	-0.144198
5	6	0	0.700323	2.620947	0.004032
6	6	0	0.250238	1.300220	0.037095
7	1	0	-3.066460	1.851540	-0.324951
8	1	0	-2.279088	4.185471	-0.377368
9	1	0	0.148032	4.682386	-0.168636
10	1	0	1.760441	2.819569	0.113439
11	7	0	1.156010	0.243466	0.259045
12	1	0	-3.108815	0.037592	1.462826
13	6	0	-1.624509	-0.394642	-0.032083
14	6	0	-1.083884	-1.389766	-0.851393
15	6	0	-1.590423	-2.683070	-0.818606
16	6	0	-2.641958	-3.002220	0.034024
17	6	0	-3.187893	-2.018554	0.851433
18	6	0	-2.685044	-0.723556	0.816163
19	1	0	-0.273897	-1.148245	-1.530152
20	1	0	-1.165760	-3.442019	-1.466494
21	1	0	-3.035809	-4.012234	0.060428
22	1	0	-4.005456	-2.259601	1.521927
23	6	0	2.330528	0.122083	-0.073345
24	6	0	3.210471	-1.042858	0.287305
25	1	0	2.717137	-1.784315	0.918792
26	9	0	4.315268	-0.576108	0.929263
27	9	0	3.630872	-1.644497	-0.858626

```
Temperature   298.150 Kelvin.  Pressure   1.00000 Atm
 Zero-point correction=                           0.204760 (Hartree/Particle)
 Thermal correction to Energy=                    0.218718
 Thermal correction to Enthalpy=                  0.219662
 Thermal correction to Gibbs Free Energy=         0.161136
 Sum of electronic and zero-point Energies=           -793.605720
 Sum of electronic and thermal Energies=              -793.591762
 Sum of electronic and thermal Enthalpies=            -793.590818
 Sum of electronic and thermal Free Energies=         -793.649344

Temperature   313.000 Kelvin.  Pressure   1.00000 Atm
 Zero-point correction=                           0.204760 (Hartree/Particle)
 Thermal correction to Energy=                    0.219999
 Thermal correction to Enthalpy=                  0.220991
 Thermal correction to Gibbs Free Energy=         0.158188
 Sum of electronic and zero-point Energies=           -793.605720
 Sum of electronic and thermal Energies=              -793.590480
 Sum of electronic and thermal Enthalpies=            -793.589489
 Sum of electronic and thermal Free Energies=         -793.652292
```

Isonitrile+CF_2H cyclize TS (**TS$_{5a\text{-}6a\text{-}CF2H}$**)

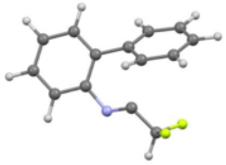

E(UwB97XD) = -793.798831187 A.U.

Center Number	Atomic Number	Atomic Type	Coordinates (Angstroms) X	Y	Z
1	6	0	-1.506122	0.487398	0.120703
2	6	0	-2.718294	1.170021	0.225062
3	6	0	-3.928303	0.491434	0.162081
4	6	0	-3.946183	-0.887297	-0.028010
5	6	0	-2.753082	-1.579021	-0.174534
6	6	0	-1.536578	-0.905450	-0.094462
7	1	0	-2.704283	2.243517	0.380722
8	1	0	-4.859050	1.038119	0.262621
9	1	0	-4.888913	-1.419839	-0.078244
10	1	0	-2.745042	-2.646886	-0.361556
11	7	0	-0.359274	-1.646825	-0.347406
12	1	0	-0.646833	2.585339	-1.373476
13	6	0	-0.203329	1.176627	0.176624
14	6	0	0.802559	0.646162	1.029876
15	6	0	2.089887	1.230035	1.018261
16	6	0	2.385790	2.244258	0.127711
17	6	0	1.399332	2.744616	-0.726455
18	6	0	0.112948	2.210419	-0.695467
19	1	0	0.502829	0.070045	1.899320
20	1	0	2.842150	0.868254	1.709173
21	1	0	3.382279	2.671814	0.107007
22	1	0	1.633794	3.546688	-1.416911
23	6	0	0.762649	-1.288841	0.050378
24	6	0	2.050337	-1.958950	-0.308093
25	1	0	1.919763	-2.944350	-0.760252
26	9	0	2.818618	-2.083421	0.811878
27	9	0	2.737888	-1.158067	-1.171724

Temperature 298.150 Kelvin. Pressure 1.00000 Atm
Imaginary = -422.4602 cm-1

Zero-point correction=	0.203680	(Hartree/Particle)
Thermal correction to Energy=	0.216685	
Thermal correction to Enthalpy=	0.217629	
Thermal correction to Gibbs Free Energy=	0.162524	
Sum of electronic and zero-point Energies=	-793.595151	
Sum of electronic and thermal Energies=	-793.582147	
Sum of electronic and thermal Enthalpies=	-793.581202	
Sum of electronic and thermal Free Energies=	-793.636307	

Temperature 313.000 Kelvin. Pressure 1.00000 Atm
Imaginary = -422.4630 cm-1

Zero-point correction=	0.203680	(Hartree/Particle)
Thermal correction to Energy=	0.217923	
Thermal correction to Enthalpy=	0.218914	
Thermal correction to Gibbs Free Energy=	0.159748	
Sum of electronic and zero-point Energies=	-793.595151	
Sum of electronic and thermal Energies=	-793.580908	
Sum of electronic and thermal Enthalpies=	-793.579917	
Sum of electronic and thermal Free Energies=	-793.639083	

Isonitrile+CF_2H cyclize (**6a-CF_2H**)

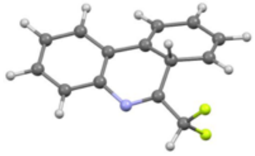

E(UwB97XD) = -793.842244263 A.U.

Center Number	Atomic Number	Atomic Type	Coordinates (Angstroms) X	Y	Z
1	6	0	1.526615	0.459743	-0.066273
2	6	0	2.780171	1.085579	-0.126561
3	6	0	3.947368	0.340925	-0.082283
4	6	0	3.896511	-1.048362	0.037799
5	6	0	2.668245	-1.683954	0.122448
6	6	0	1.488191	-0.944347	0.065847
7	1	0	2.838927	2.161984	-0.240839
8	1	0	4.905714	0.843618	-0.148797
9	1	0	4.812480	-1.626856	0.071630
10	1	0	2.597759	-2.759384	0.240903
11	7	0	0.278158	-1.644351	0.222687
12	1	0	0.975432	3.063515	0.559912
13	6	0	0.260258	1.169877	-0.109916
14	6	0	-0.921725	0.360678	-0.583064
15	6	0	-2.242233	1.049983	-0.391658
16	6	0	-2.317257	2.345308	-0.011687
17	6	0	-1.148591	3.100953	0.290848
18	6	0	0.105799	2.491850	0.252892
19	1	0	-0.798603	0.207580	-1.677176
20	1	0	-3.141752	0.499385	-0.634849
21	1	0	-3.288662	2.820355	0.075691
22	1	0	-1.239297	4.136204	0.596375
23	6	0	-0.820000	-1.055580	-0.032107
24	6	0	-2.051086	-1.899815	0.174423
25	1	0	-1.794006	-2.927798	0.424739
26	9	0	-2.820372	-1.899691	-0.957912
27	9	0	-2.829482	-1.383105	1.172285

Temperature 298.150 Kelvin. Pressure 1.00000 Atm
Zero-point correction= 0.205684 (Hartree/Particle)
Thermal correction to Energy= 0.218401
Thermal correction to Enthalpy= 0.219345
Thermal correction to Gibbs Free Energy= 0.165269
Sum of electronic and zero-point Energies= -793.636560
Sum of electronic and thermal Energies= -793.623843
Sum of electronic and thermal Enthalpies= -793.622899
Sum of electronic and thermal Free Energies= -793.676976

Temperature 313.000 Kelvin. Pressure 1.00000 Atm
Zero-point correction= 0.205684 (Hartree/Particle)
Thermal correction to Energy= 0.219643
Thermal correction to Enthalpy= 0.220634
Thermal correction to Gibbs Free Energy= 0.162544
Sum of electronic and zero-point Energies= -793.636560
Sum of electronic and thermal Energies= -793.622601
Sum of electronic and thermal Enthalpies= -793.621610
Sum of electronic and thermal Free Energies= -793.679700

2a ub3lyp/6-311+g(d,p)

Center Number	Atomic Number	Atomic Type	Coordinates (Angstroms) X	Y	Z
1	6	0	-0.536200	3.076023	-0.590235
2	7	0	-0.940064	1.996667	-0.366785
3	6	0	-1.470885	0.738725	-0.117922
4	6	0	-2.864377	0.611422	-0.093709
5	6	0	-0.629451	-0.376478	0.070596
6	6	0	-3.446033	-0.631653	0.115517
7	1	0	-3.471062	1.494574	-0.249821
8	6	0	-1.250229	-1.616961	0.273085
9	6	0	-2.634592	-1.750428	0.296873
10	1	0	-4.525295	-0.724919	0.134908
11	1	0	-0.623585	-2.486243	0.434351
12	1	0	-3.078544	-2.724835	0.464441
13	6	0	0.854652	-0.285862	0.053020
14	6	0	1.596546	-1.146821	-0.767662
15	6	0	1.542328	0.619291	0.871989
16	6	0	2.988410	-1.101244	-0.772726
17	1	0	1.078077	-1.842033	-1.418574
18	6	0	2.933741	0.659872	0.870659
19	1	0	0.988408	1.286333	1.521237
20	6	0	3.661493	-0.198178	0.047751
21	1	0	3.545671	-1.767463	-1.421669
22	1	0	3.449426	1.363193	1.514568
23	1	0	4.744927	-0.161526	0.044633

Zero-point correction= 0.178617 (Hartree/Particle)
Thermal correction to Energy= 0.189498
Thermal correction to Enthalpy= 0.190442
Thermal correction to Gibbs Free Energy= 0.141274
Sum of electronic and zero-point Energies= -555.472588
Sum of electronic and thermal Energies= -555.461707
Sum of electronic and thermal Enthalpies= -555.460763
Sum of electronic and thermal Free Energies= -555.509931

TS_{2a+Mu} ub3lyp/6-311+g(d,p)

Center Number	Atomic Number	Atomic Type	Coordinates (Angstroms) X	Y	Z
1	6	0	-0.527423	3.010856	-0.685378
2	1	0	0.068332	4.220475	0.964792
3	7	0	-0.938633	1.938510	-0.436066
4	6	0	-1.470751	0.692205	-0.149840
5	6	0	-2.865183	0.568135	-0.120675
6	6	0	-0.630551	-0.419206	0.069898
7	6	0	-3.448196	-0.667305	0.124662
8	1	0	-3.470650	1.447436	-0.301306
9	6	0	-1.253702	-1.651973	0.307618
10	6	0	-2.638351	-1.782149	0.336414
11	1	0	-4.527566	-0.758135	0.148299
12	1	0	-0.628608	-2.517421	0.493544
13	1	0	-3.083887	-2.750552	0.532051
14	6	0	0.853311	-0.330439	0.050358
15	6	0	1.595058	-1.218943	-0.740794
16	6	0	1.541266	0.601322	0.839044
17	6	0	2.986881	-1.174649	-0.746496
18	1	0	1.076478	-1.934971	-1.368640
19	6	0	2.932760	0.640665	0.836949
20	1	0	0.988318	1.290044	1.466190
21	6	0	3.660201	-0.245049	0.043684
22	1	0	3.543989	-1.862615	-1.372470
23	1	0	3.448430	1.364820	1.457278
24	1	0	4.743667	-0.209463	0.040057

Zero-point correction= 0.181132 (Hartree/Particle)
Thermal correction to Energy= 0.192411
Thermal correction to Enthalpy= 0.193355
Thermal correction to Gibbs Free Energy= 0.142995
Sum of electronic and zero-point Energies= -555.972762
Sum of electronic and thermal Energies= -555.961483
Sum of electronic and thermal Enthalpies= -555.960539
Sum of electronic and thermal Free Energies= -556.010899

Trans-5a-Mu ub3lyp/6-311+g(d,p)

Center Number	Atomic Number	Atomic Type	Coordinates (Angstroms) X	Y	Z
1	6	0	-1.378512	2.939448	-0.738693
2	1	0	-0.903939	3.927162	-0.726915
(Iso=0.113429,NMagM=8.890597)					
3	7	0	-0.925645	1.946594	-0.167008
4	6	0	-1.451689	0.645011	-0.033607
5	6	0	-2.837033	0.440662	0.007076
6	6	0	-0.562500	-0.443003	0.079472
7	6	0	-3.355685	-0.839499	0.146963
8	1	0	-3.489893	1.300902	-0.079510
9	6	0	-1.114906	-1.725911	0.203732
10	6	0	-2.490405	-1.929233	0.241107
11	1	0	-4.429244	-0.986411	0.180485
12	1	0	-0.444220	-2.572198	0.297699
13	1	0	-2.884979	-2.932384	0.355054
14	6	0	0.917150	-0.280898	0.052598
15	6	0	1.691018	-1.059766	-0.818622
16	6	0	1.573845	0.609011	0.914466
17	6	0	3.080221	-0.951637	-0.831917
18	1	0	1.198480	-1.741777	-1.502891
19	6	0	2.961894	0.711982	0.905544
20	1	0	0.993299	1.214901	1.598659
21	6	0	3.720914	-0.065820	0.031423
22	1	0	3.659872	-1.556763	-1.520185
23	1	0	3.452483	1.399559	1.585633
24	1	0	4.801777	0.018776	0.023782

Zero-point correction= 0.207553 (Hartree/Particle)
Thermal correction to Energy= 0.218763
Thermal correction to Enthalpy= 0.219714
Thermal correction to Gibbs Free Energy= 0.168619
Sum of electronic and zero-point Energies= -556.008182
Sum of electronic and thermal Energies= -555.996972
Sum of electronic and thermal Enthalpies= -555.996022
Sum of electronic and thermal Free Energies= -556.047117

Temperature: 300K

Isotropic Fermi Contact Couplings

	Atom	a.u.	MegaHertz	Gauss	10(-4) cm-1
1	C(13)	0.18106	203.54491	72.62989	67.89527
2	H(1)	0.04974	**707.80829**	252.56360	236.09943
3	N(14)	0.03664	11.83980	4.22474	3.94933
4	C(13)	0.03810	42.83629	15.28505	14.28865
5	C(13)	0.00181	2.03869	0.72746	0.68004
6	C(13)	0.00265	2.97834	1.06275	0.99347

7	C(13)	-0.00135	-1.51615	-0.54100	-0.50573
8	H(1)	-0.00062	-2.75992	-0.98481	-0.92061
9	C(13)	-0.00147	-1.65079	-0.58904	-0.55065
10	C(13)	0.00257	2.88714	1.03020	0.96305
11	H(1)	0.00096	4.30348	1.53559	1.43549
12	H(1)	0.00011	0.51312	0.18309	0.17116
13	H(1)	-0.00034	-1.54050	-0.54969	-0.51385
14	C(13)	0.00144	1.61839	0.57748	0.53984
15	C(13)	-0.00115	-1.28995	-0.46029	-0.43028
16	C(13)	-0.00007	-0.07488	-0.02672	-0.02498
17	C(13)	0.00195	2.19434	0.78300	0.73195
18	H(1)	0.00025	1.10522	0.39437	0.36866
19	C(13)	0.00216	2.43203	0.86781	0.81124
20	H(1)	0.00041	1.82181	0.65007	0.60769
21	C(13)	-0.00178	-2.00369	-0.71497	-0.66836
22	H(1)	-0.00028	-1.24469	-0.44413	-0.41518
23	H(1)	-0.00028	-1.26429	-0.45113	-0.42172
24	H(1)	0.00027	1.18894	0.42424	0.39659

Cis-5a-Mu ub3lyp/6-311+g(d,p)

Center Number	Atomic Number	Atomic Type	Coordinates (Angstroms) X	Y	Z
1	6	0	1.190630	2.950159	0.759422
2	1	0	1.850736	3.075245	1.633396
(Iso=0.113429,NMagM=8.890597)					
3	7	0	0.945349	1.972284	0.074594
4	6	0	1.450508	0.658283	-0.011331
5	6	0	2.835657	0.458209	-0.056969
6	6	0	0.561768	-0.434289	-0.088033
7	6	0	3.358296	-0.825654	-0.160465
8	1	0	3.491481	1.320154	-0.017608
9	6	0	1.120035	-1.716977	-0.173894
10	6	0	2.496681	-1.919192	-0.213442
11	1	0	4.432110	-0.968714	-0.200096
12	1	0	0.451877	-2.568118	-0.237263
13	1	0	2.892640	-2.924406	-0.299468
14	6	0	-0.917941	-0.274699	-0.056781
15	6	0	-1.688507	-1.056456	0.815259
16	6	0	-1.578155	0.616300	-0.915033
17	6	0	-3.077866	-0.952508	0.830729
18	1	0	-1.193610	-1.738072	1.498254
19	6	0	-2.966377	0.715192	-0.903183
20	1	0	-1.000884	1.229556	-1.595125
21	6	0	-3.722147	-0.066947	-0.030207
22	1	0	-3.654744	-1.560158	1.519113
23	1	0	-3.459382	1.404661	-1.579472
24	1	0	-4.803161	0.015345	-0.020315

Zero-point correction= 0.206014 (Hartree/Particle)
Thermal correction to Energy= 0.217272
Thermal correction to Enthalpy= 0.218222
Thermal correction to Gibbs Free Energy= 0.167214
Sum of electronic and zero-point Energies= -556.002248
Sum of electronic and thermal Energies= -555.990989
Sum of electronic and thermal Enthalpies= -555.990039
Sum of electronic and thermal Free Energies= -556.041047

Temperature: 300K

Isotropic Fermi Contact Couplings

	Atom	a.u.	MegaHertz	Gauss	10(-4) cm-1
1	C(13)	0.42123	473.54377	168.97219	157.95720
2	H(1)	0.06150	**875.06675**	312.24558	291.89085
3	N(14)	0.24859	80.31927	28.65991	26.79162
4	C(13)	0.05429	61.03645	21.77932	20.35957
5	C(13)	0.00347	3.89541	1.38998	1.29937
6	C(13)	0.00040	0.44884	0.16016	0.14972

7	C(13)	-0.00122	-1.36642	-0.48757	-0.45579
8	H(1)	-0.00054	-2.39164	-0.85340	-0.79776
9	C(13)	-0.00166	-1.86652	-0.66602	-0.62260
10	C(13)	0.00385	4.32384	1.54285	1.44228
11	H(1)	0.00073	3.25625	1.16191	1.08617
12	H(1)	0.00027	1.21867	0.43485	0.40650
13	H(1)	-0.00069	-3.09812	-1.10549	-1.03342
14	C(13)	0.00305	3.43136	1.22439	1.14458
15	C(13)	-0.00261	-2.93011	-1.04554	-0.97738
16	C(13)	-0.00304	-3.42015	-1.22040	-1.14084
17	C(13)	0.00404	4.53981	1.61992	1.51432
18	H(1)	0.00052	2.33427	0.83292	0.77863
19	C(13)	0.00413	4.64107	1.65605	1.54810
20	H(1)	0.00061	2.71111	0.96739	0.90433
21	C(13)	-0.00376	-4.22286	-1.50682	-1.40859
22	H(1)	-0.00057	-2.54452	-0.90795	-0.84876
23	H(1)	-0.00058	-2.57483	-0.91876	-0.85887
24	H(1)	0.00055	2.48077	0.88520	0.82749

TS$_{5a\text{-}6a_Mu}$ ub3lyp/6-311+g(d,p)

Center Number	Atomic Number	Atomic Type	Coordinates (Angstroms) X	Y	Z
1	6	0	3.552250	-0.236055	0.041304
2	6	0	2.784685	0.891927	-0.222677
3	6	0	1.389035	0.825257	-0.180817
4	6	0	0.741402	-0.400864	0.116248
5	6	0	1.536919	-1.532286	0.336415
6	6	0	2.926238	-1.454741	0.310000
7	6	0	-0.731517	-0.449288	0.138587
8	6	0	-1.432060	0.576728	0.848325
9	6	0	-0.518926	2.199183	-0.252763
10	6	0	-2.851923	0.598357	0.796904
11	1	0	-3.393118	1.327651	1.389494
12	6	0	-3.539685	-0.303457	0.003983
13	6	0	-2.846259	-1.292418	-0.708662
14	6	0	-1.453009	-1.356878	-0.634673
15	1	0	4.633954	-0.170406	0.021101
16	1	0	3.247666	1.837906	-0.477705
17	1	0	1.051687	-2.475566	0.562373
18	1	0	3.519370	-2.341467	0.502275
19	1	0	-1.140410	3.002260	-0.657419
(Iso=0.113429,NMagM=8.890597)					
20	1	0	-4.622415	-0.263413	-0.045334
21	1	0	-3.388659	-2.003568	-1.320564
22	1	0	-0.916701	-2.104897	-1.208843
23	1	0	-0.943804	1.035083	1.701018
24	7	0	0.658505	1.966231	-0.574207

Zero-point correction= 0.207537 (Hartree/Particle)
Thermal correction to Energy= 0.217427
Thermal correction to Enthalpy= 0.218371
Thermal correction to Gibbs Free Energy= 0.171392
Sum of electronic and zero-point Energies= -555.993681
Sum of electronic and thermal Energies= -555.983790
Sum of electronic and thermal Enthalpies= -555.982846
Sum of electronic and thermal Free Energies= -556.029826

6a-Mu ub3lyp/6-311+g(d,p)

Center Number	Atomic Number	Atomic Type	Coordinates (Angstroms) X	Y	Z
1	6	0	3.563626	-0.187762	-0.033231
2	6	0	2.769532	0.946235	-0.152521
3	6	0	1.376195	0.850438	-0.098210
4	6	0	0.754307	-0.417867	0.067797
5	6	0	1.580539	-1.554354	0.164087
6	6	0	2.962682	-1.441947	0.121256
7	6	0	-0.693879	-0.466366	0.103554
8	6	0	-1.376817	0.826518	0.486255
9	6	0	-0.613755	2.021851	-0.059155
10	6	0	-2.848432	0.857362	0.190360
11	1	0	-3.360422	1.810292	0.278460
12	6	0	-3.529656	-0.275856	-0.114755
13	6	0	-2.855314	-1.524796	-0.251931
14	6	0	-1.460500	-1.585463	-0.178046
15	1	0	4.643456	-0.101150	-0.067289
16	1	0	3.205475	1.927422	-0.298421
17	1	0	1.130863	-2.530227	0.305821
18	1	0	3.577083	-2.330234	0.215452
19	1	0	-1.174897	2.942400	-0.226985
(Iso=0.113429,NMagM=8.890597)					
20	1	0	-4.600263	-0.233299	-0.284773
21	1	0	-3.421213	-2.416238	-0.493146
22	1	0	-0.967699	-2.524139	-0.406773
23	1	0	-1.268785	0.928307	1.593787
24	7	0	0.637891	2.034130	-0.298412

Zero-point correction= 0.212372 (Hartree/Particle)
Thermal correction to Energy= 0.222155
Thermal correction to Enthalpy= 0.223099
Thermal correction to Gibbs Free Energy= 0.176278
Sum of electronic and zero-point Energies= -556.025663
Sum of electronic and thermal Energies= -556.015879
Sum of electronic and thermal Enthalpies= -556.014935
Sum of electronic and thermal Free Energies= -556.061756

Temperature: 298K

Isotropic Fermi Contact Couplings

	Atom	a.u.	MegaHertz	Gauss	10(-4) cm-1
1	C(13)	0.00911	10.24340	3.65510	3.41683
2	C(13)	-0.00686	-7.71312	-2.75223	-2.57282
3	C(13)	0.01029	11.57290	4.12950	3.86030
4	C(13)	-0.01975	-22.20683	-7.92395	-7.40740
5	C(13)	0.01230	13.82855	4.93437	4.61271
6	C(13)	-0.00837	-9.41447	-3.35932	-3.14033

7	C(13)	0.03922	44.09472	15.73409	14.70841
8	C(13)	-0.02717	-30.54938	-10.90078	-10.19018
9	C(13)	0.04304	48.37973	17.26309	16.13774
10	C(13)	0.03279	36.86308	13.15366	12.29620
11	H(1)	-0.00498	-22.24303	-7.93687	-7.41948
12	C(13)	-0.02553	-28.70606	-10.24304	-9.57531
13	C(13)	0.03505	39.40019	14.05897	13.14249
14	C(13)	-0.02954	-33.20334	-11.84778	-11.07544
15	H(1)	-0.00164	-7.34866	-2.62219	-2.45125
16	H(1)	0.00059	2.62644	0.93718	0.87609
17	H(1)	-0.00169	-7.54303	-2.69154	-2.51608
18	H(1)	0.00076	3.38486	1.20780	1.12907
19	H(1)	-0.00036	**-5.18014**	-1.84840	-1.72791
20	H(1)	0.00203	9.07575	3.23845	3.02734
21	H(1)	-0.00601	-26.85292	-9.58179	-8.95717
22	H(1)	0.00254	11.35125	4.05041	3.78637
23	H(1)	0.04565	204.04722	72.80912	68.06283
24	N(14)	-0.00279	-0.90285	-0.32216	-0.30116

8a-i ub3lyp/6-311+g(d,p)

Center Number	Atomic Number	Atomic Type	Coordinates (Angstroms) X	Y	Z
1	6	0	-0.567523	3.081893	-0.595077
2	7	0	-0.898334	1.974401	-0.366460
3	6	0	-1.405174	0.725146	-0.112132
4	6	0	-2.910864	0.647955	-0.087147
5	6	0	-0.584991	-0.377693	0.072318
6	6	0	-3.435133	-0.736391	0.127204
7	1	0	-3.297246	1.328505	0.690435
8	6	0	-1.190487	-1.639838	0.272838
9	6	0	-2.602036	-1.796366	0.292465
10	1	0	-4.511300	-0.866572	0.149499
11	1	0	-0.553549	-2.499498	0.437044
12	1	0	-3.014281	-2.787009	0.451613
13	6	0	0.901264	-0.284151	0.055212
14	6	0	1.644538	-1.131402	-0.777740
15	6	0	1.586029	0.612852	0.884504
16	6	0	3.036210	-1.078396	-0.786215
17	1	0	1.127673	-1.822871	-1.433960
18	6	0	2.977441	0.660455	0.880560
19	1	0	1.029093	1.269240	1.542096
20	6	0	3.707152	-0.182909	0.044443
21	1	0	3.595185	-1.733762	-1.444722
22	1	0	3.491435	1.357771	1.532371
23	1	0	4.790436	-0.141377	0.039389
24	1	0	-3.307673	1.067834	-1.025950

Atom 7 (Iso=0.113429,NMagM=8.890597)
Zero-point correction= 0.209715 (Hartree/Particle)
Thermal correction to Energy= 0.221149
Thermal correction to Enthalpy= 0.222093
Thermal correction to Gibbs Free Energy= 0.171080
Sum of electronic and zero-point Energies= -555.993618
Sum of electronic and thermal Energies= -555.982184
Sum of electronic and thermal Enthalpies= -555.981240
Sum of electronic and thermal Free Energies= -556.032253

Atom 24 (Iso=0.113429,NMagM=8.890597)
Zero-point correction= 0.209812 (Hartree/Particle)
Thermal correction to Energy= 0.221247
Thermal correction to Enthalpy= 0.222191
Thermal correction to Gibbs Free Energy= 0.171176
Sum of electronic and zero-point Energies= -555.993521
Sum of electronic and thermal Energies= -555.982086
Sum of electronic and thermal Enthalpies= -555.981142
Sum of electronic and thermal Free Energies= -556.032157

Isotropic Fermi Contact Couplings

	Atom	a.u.	MegaHertz	Gauss	10(-4) cm-1
1	C(13)	0.00669	7.51911	2.68301	2.50811

2	N(14)	-0.02409	-7.78240	-2.77695	-2.59593
3	C(13)	0.03234	36.35719	12.97315	12.12745
4	C(13)	-0.02795	-31.42232	-11.21226	-10.48136
5	C(13)	-0.03186	-35.81315	-12.77902	-11.94598
6	C(13)	0.02457	27.62267	9.85646	9.21393
7	H(1)	0.02930	**130.96504**	46.73158	43.68524
8	C(13)	0.03445	38.73177	13.82046	12.91953
9	C(13)	-0.02811	-31.60073	-11.27593	-10.54087
10	H(1)	-0.00551	-24.60805	-8.78076	-8.20836
11	H(1)	-0.00738	-32.96724	-11.76353	-10.99669
12	H(1)	0.00192	8.57376	3.05933	2.85990
13	C(13)	0.00517	5.80753	2.07227	1.93718
14	C(13)	-0.00414	-4.65019	-1.65930	-1.55114
15	C(13)	-0.00482	-5.42175	-1.93462	-1.80850
16	C(13)	0.00057	0.63853	0.22784	0.21299
17	H(1)	0.00034	1.50985	0.53875	0.50363
18	C(13)	0.00038	0.42520	0.15172	0.14183
19	H(1)	0.00021	0.95607	0.34115	0.31891
20	C(13)	-0.00062	-0.69149	-0.24674	-0.23066
21	H(1)	-0.00012	-0.51779	-0.18476	-0.17272
22	H(1)	-0.00012	-0.52719	-0.18811	-0.17585
23	H(1)	0.00019	0.85795	0.30614	0.28618
24	H(1)	0.02783	**124.38371**	44.38320	41.48994

Average A_p = 127.644 MHz
Isotope effect 1.20
Average A_μ = 487.7 MHz

8a-ii ub3lyp/6-311+g(d,p)

Center Number	Atomic Number	Atomic Type	Coordinates (Angstroms) X	Y	Z
1	6	0	-0.510024	3.113279	-0.583844
2	7	0	-0.913209	2.036524	-0.350622
3	6	0	-1.446924	0.774654	-0.099296
4	6	0	-2.808121	0.667258	-0.065578
5	6	0	-0.571693	-0.357887	0.050922
6	6	0	-3.504799	-0.636751	0.145165
7	1	0	-3.405126	1.558446	-0.216068
8	6	0	-1.212136	-1.629069	0.202804
9	6	0	-2.558755	-1.790779	0.253346
10	1	0	-4.141860	-0.582357	1.045759
11	1	0	-0.574911	-2.498253	0.319845
12	1	0	-2.979607	-2.779882	0.397098
13	6	0	0.896063	-0.274954	0.042539
14	6	0	1.650702	-1.258171	-0.627250
15	6	0	1.595434	0.737445	0.725471
16	6	0	3.040532	-1.225151	-0.621644
17	1	0	1.139914	-2.037655	-1.180832
18	6	0	2.985752	0.763736	0.734546
19	1	0	1.049927	1.495824	1.270989
20	6	0	3.716457	-0.213762	0.060265
21	1	0	3.597063	-1.985915	-1.157652
22	1	0	3.500216	1.550092	1.275300
23	1	0	4.800094	-0.187388	0.065510
24	1	0	-4.228175	-0.807667	-0.670264

Atom 10 (Iso=0.113429,NMagM=8.890597)
Zero-point correction= 0.209673 (Hartree/Particle)
Thermal correction to Energy= 0.221054
Thermal correction to Enthalpy= 0.221999
Thermal correction to Gibbs Free Energy= 0.171283
Sum of electronic and zero-point Energies= -555.990116
Sum of electronic and thermal Energies= -555.978735
Sum of electronic and thermal Enthalpies= -555.977791
Sum of electronic and thermal Free Energies= -556.028507

Atom 24 (Iso=0.113429,NMagM=8.890597)
Zero-point correction= 0.209785 (Hartree/Particle)
Thermal correction to Energy= 0.221168
Thermal correction to Enthalpy= 0.222112
Thermal correction to Gibbs Free Energy= 0.171393
Sum of electronic and zero-point Energies= -555.990005
Sum of electronic and thermal Energies= -555.978622
Sum of electronic and thermal Enthalpies= -555.977678
Sum of electronic and thermal Free Energies= -556.028396

Isotropic Fermi Contact Couplings

Atom	a.u.	MegaHertz	Gauss	10(-4) cm-1

1	C(13)	-0.00171	-1.92136	-0.68559	-0.64090
2	N(14)	0.00678	2.18916	0.78115	0.73023
3	C(13)	-0.03420	-38.44458	-13.71798	-12.82373
4	C(13)	0.02528	28.42330	10.14214	9.48099
5	C(13)	0.03863	43.42271	15.49430	14.48426
6	C(13)	-0.02604	-29.27439	-10.44583	-9.76488
7	H(1)	-0.00555	-24.80994	-8.85280	-8.27570
8	C(13)	-0.02936	-33.00268	-11.77618	-11.00851
9	C(13)	0.02393	26.90120	9.59902	8.97327
10	H(1)	0.02919	**130.48676**	46.56092	43.52570
11	H(1)	0.00190	8.49475	3.03114	2.83354
12	H(1)	-0.00530	-23.71195	-8.46101	-7.90946
13	C(13)	-0.02108	-23.69492	-8.45494	-7.90377
14	C(13)	0.01578	17.73877	6.32963	5.91702
15	C(13)	0.01634	18.36387	6.55269	6.12553
16	C(13)	-0.00448	-5.03898	-1.79803	-1.68082
17	H(1)	-0.00121	-5.42931	-1.93731	-1.81102
18	C(13)	-0.00451	-5.06898	-1.80874	-1.69083
19	H(1)	-0.00122	-5.46343	-1.94949	-1.82240
20	C(13)	0.00575	6.45926	2.30482	2.15458
21	H(1)	0.00061	2.74743	0.98035	0.91644
22	H(1)	0.00062	2.76007	0.98486	0.92066
23	H(1)	-0.00122	-5.46706	-1.95078	-1.82362
24	H(1)	0.02725	**121.81464**	43.46649	40.63299

Average A_p = 126.1507 MHz
Isotope effect 1.20
Average A_μ = 481.8 MHz

8a-iii ub3lyp/6-311+g(d,p)

Center Number	Atomic Number	Atomic Type	Coordinates (Angstroms) X	Y	Z
1	6	0	-0.408051	3.091930	-0.596415
2	7	0	-0.851917	2.022002	-0.374726
3	6	0	-1.415246	0.801444	-0.122984
4	6	0	-2.841011	0.712597	-0.087201
5	6	0	-0.601113	-0.371047	0.061949
6	6	0	-3.470751	-0.471373	0.103195
7	1	0	-3.406458	1.626763	-0.225981
8	6	0	-1.224991	-1.570794	0.251824
9	6	0	-2.711528	-1.748281	0.279366
10	1	0	-4.554093	-0.511757	0.125484
11	1	0	-0.616470	-2.453917	0.413064
12	1	0	-3.009957	-2.238029	1.221885
13	6	0	0.887409	-0.295940	0.049172
14	6	0	1.624440	-1.119287	-0.811526
15	6	0	1.578608	0.563006	0.913305
16	6	0	3.017368	-1.089129	-0.805828
17	1	0	1.100886	-1.774119	-1.499101
18	6	0	2.970305	0.589848	0.921483
19	1	0	1.026829	1.206015	1.588637
20	6	0	3.694484	-0.235192	0.062014
21	1	0	3.571829	-1.727806	-1.484302
22	1	0	3.489328	1.257153	1.600179
23	1	0	4.778260	-0.208883	0.066131
24	1	0	-3.016276	-2.476130	-0.493041

Atom 12 (Iso=0.113429,NMagM=8.890597)
Zero-point correction= 0.209717 (Hartree/Particle)
Thermal correction to Energy= 0.221134
Thermal correction to Enthalpy= 0.222079
Thermal correction to Gibbs Free Energy= 0.171084
Sum of electronic and zero-point Energies= -555.992654
Sum of electronic and thermal Energies= -555.981236
Sum of electronic and thermal Enthalpies= -555.980292
Sum of electronic and thermal Free Energies= -556.031287

Atom 24 (Iso=0.113429,NMagM=8.890597)
Zero-point correction= 0.209613 (Hartree/Particle)
Thermal correction to Energy= 0.221029
Thermal correction to Enthalpy= 0.221973
Thermal correction to Gibbs Free Energy= 0.170981
Sum of electronic and zero-point Energies= -555.992758
Sum of electronic and thermal Energies= -555.981342
Sum of electronic and thermal Enthalpies= -555.980398
Sum of electronic and thermal Free Energies= -556.031390

Isotropic Fermi Contact Couplings

	Atom	a.u.	MegaHertz	Gauss	10(-4) cm-1
1	C(13)	0.00998	11.21968	4.00346	3.74248

2	N(14)	-0.03131	-10.11723	-3.61008	-3.37474
3	C(13)	0.04034	45.35441	16.18358	15.12860
4	C(13)	-0.02856	-32.10768	-11.45682	-10.70997
5	C(13)	-0.02958	-33.25561	-11.86643	-11.09288
6	C(13)	0.02271	25.52719	9.10874	8.51496
7	H(1)	0.00188	8.40446	2.99892	2.80343
8	C(13)	0.02421	27.21144	9.70972	9.07676
9	C(13)	-0.02500	-28.10701	-10.02928	-9.37549
10	H(1)	-0.00514	-22.99207	-8.20414	-7.66933
11	H(1)	-0.00532	-23.80000	-8.49243	-7.93883
12	H(1)	0.02657	**118.78561**	42.38566	39.62262
13	C(13)	0.00427	4.80240	1.71361	1.60191
14	C(13)	-0.00356	-4.00545	-1.42924	-1.33607
15	C(13)	-0.00425	-4.77747	-1.70472	-1.59359
16	C(13)	0.00041	0.46438	0.16570	0.15490
17	H(1)	0.00026	1.15900	0.41356	0.38660
18	C(13)	0.00018	0.20434	0.07291	0.06816
19	H(1)	0.00016	0.72378	0.25826	0.24143
20	C(13)	-0.00047	-0.52416	-0.18703	-0.17484
21	H(1)	-0.00010	-0.43589	-0.15554	-0.14540
22	H(1)	-0.00009	-0.40401	-0.14416	-0.13476
23	H(1)	0.00014	0.64406	0.22982	0.21483
24	H(1)	0.02791	**124.73832**	44.50974	41.60823

Average A_p = 121.762 MHz
Isotope effect 1.20
Average A_μ = 465.1 MHz

8a-iv ub3lyp/6-311+g(d,p)

Center Number	Atomic Number	Atomic Type	Coordinates (Angstroms) X	Y	Z
1	6	0	-0.570877	3.132620	-0.577885
2	7	0	-0.921543	2.035629	-0.352891
3	6	0	-1.434643	0.763745	-0.113177
4	6	0	-2.848361	0.666653	-0.061628
5	6	0	-0.598259	-0.329601	0.028214
6	6	0	-3.464787	-0.587665	0.149514
7	1	0	-3.435631	1.565226	-0.191730
8	6	0	-1.226156	-1.697122	0.199108
9	6	0	-2.721087	-1.719284	0.275801
10	1	0	-4.546765	-0.637099	0.203133
11	1	0	-0.900068	-2.359225	-0.621042
12	1	0	-3.199000	-2.680780	0.427684
13	6	0	0.874934	-0.258653	0.028131
14	6	0	1.634500	-1.233239	-0.645176
15	6	0	1.567904	0.739901	0.735611
16	6	0	3.025070	-1.195818	-0.633250
17	1	0	1.136245	-2.014806	-1.206386
18	6	0	2.958121	0.768811	0.755664
19	1	0	1.015371	1.487787	1.288517
20	6	0	3.694538	-0.194732	0.068144
21	1	0	3.586014	-1.949164	-1.174965
22	1	0	3.467219	1.546108	1.314211
23	1	0	4.778093	-0.167485	0.081472
24	1	0	-0.796058	-2.173669	1.094919

Atom 11 (Iso=0.113429,NMagM=8.890597)
Zero-point correction= 0.209568 (Hartree/Particle)
Thermal correction to Energy= 0.221049
Thermal correction to Enthalpy= 0.221993
Thermal correction to Gibbs Free Energy= 0.170763
Sum of electronic and zero-point Energies= -555.990004
Sum of electronic and thermal Energies= -555.978524
Sum of electronic and thermal Enthalpies= -555.977579
Sum of electronic and thermal Free Energies= -556.028810

Atom 24 (Iso=0.113429,NMagM=8.890597)
Zero-point correction= 0.209784 (Hartree/Particle)
Thermal correction to Energy= 0.221270
Thermal correction to Enthalpy= 0.222214
Thermal correction to Gibbs Free Energy= 0.170976
Sum of electronic and zero-point Energies= -555.989788
Sum of electronic and thermal Energies= -555.978303
Sum of electronic and thermal Enthalpies= -555.977359
Sum of electronic and thermal Free Energies= -556.028597

Isotropic Fermi Contact Couplings

	Atom	a.u.	MegaHertz	Gauss	10(-4) cm-1
1	C(13)	-0.00202	-2.27241	-0.81085	-0.75799

2	N(14)	0.00736	2.37873	0.84879	0.79346
3	C(13)	-0.03480	-39.12142	-13.95949	-13.04950
4	C(13)	0.03548	39.88688	14.23263	13.30483
5	C(13)	0.02887	32.45647	11.58128	10.82631
6	C(13)	-0.02945	-33.11080	-11.81476	-11.04457
7	H(1)	-0.00771	-34.45803	-12.29548	-11.49396
8	C(13)	-0.02807	-31.56057	-11.26160	-10.52747
9	C(13)	0.02663	29.93641	10.68205	9.98571
10	H(1)	0.00201	8.99316	3.20898	2.99979
11	H(1)	0.03087	**137.97138**	49.23162	46.02230
12	H(1)	-0.00587	-26.24166	-9.36368	-8.75328
13	C(13)	-0.01512	-16.99993	-6.06600	-5.67057
14	C(13)	0.00776	8.72839	3.11451	2.91148
15	C(13)	0.01184	13.30662	4.74813	4.43861
16	C(13)	-0.00321	-3.60597	-1.28670	-1.20282
17	H(1)	-0.00074	-3.32183	-1.18531	-1.10804
18	C(13)	-0.00236	-2.65376	-0.94693	-0.88520
19	H(1)	-0.00073	-3.28343	-1.17161	-1.09523
20	C(13)	0.00345	3.87522	1.38277	1.29263
21	H(1)	0.00045	1.99794	0.71291	0.66644
22	H(1)	0.00039	1.74642	0.62317	0.58254
23	H(1)	-0.00069	-3.08655	-1.10136	-1.02956
24	H(1)	0.02671	**119.37511**	42.59601	39.81925

Average A_p = 128.6732 MHz
Isotope effect 1.20
Average A_μ = 491.5 MHz

8a-v ub3lyp/6-311+g(d,p)

Center Number	Atomic Number	Atomic Type	Coordinates (Angstroms) X	Y	Z
1	6	0	-0.675281	3.082630	-0.658253
2	7	0	-1.031564	1.996115	-0.392441
3	6	0	-1.516620	0.726338	-0.110780
4	6	0	-2.905691	0.571039	-0.042340
5	6	0	-0.639293	-0.372906	0.035649
6	6	0	-3.459617	-0.684770	0.162669
7	1	0	-3.532298	1.445090	-0.168930
8	6	0	-1.241190	-1.629929	0.232806
9	6	0	-2.619860	-1.790722	0.293307
10	1	0	-4.535636	-0.798331	0.216698
11	1	0	-0.596283	-2.489528	0.369734
12	1	0	-3.038621	-2.777020	0.456906
13	6	0	0.833475	-0.271147	0.000865
14	6	0	1.568069	-1.166496	-0.747307
15	6	0	1.542845	0.740112	0.875015
16	6	0	2.974868	-1.167213	-0.761916
17	1	0	1.041979	-1.883335	-1.369724
18	6	0	3.039143	0.674254	0.803063
19	1	0	1.217341	1.763345	0.643423
20	6	0	3.693752	-0.230217	0.027991
21	1	0	3.506580	-1.873876	-1.386916
22	1	0	3.594195	1.392561	1.397072
23	1	0	4.778335	-0.236242	0.002917
24	1	0	1.217749	0.598690	1.921291

Atom 19 (Iso=0.113429,NMagM=8.890597)
Zero-point correction= 0.210024 (Hartree/Particle)
Thermal correction to Energy= 0.221483
Thermal correction to Enthalpy= 0.222427
Thermal correction to Gibbs Free Energy= 0.171225
Sum of electronic and zero-point Energies= -555.989914
Sum of electronic and thermal Energies= -555.978455
Sum of electronic and thermal Enthalpies= -555.977511
Sum of electronic and thermal Free Energies= -556.028713

Atom 24 (Iso=0.113429,NMagM=8.890597)
Zero-point correction= 0.209562 (Hartree/Particle)
Thermal correction to Energy= 0.221018
Thermal correction to Enthalpy= 0.221962
Thermal correction to Gibbs Free Energy= 0.170766
Sum of electronic and zero-point Energies= -555.990376
Sum of electronic and thermal Energies= -555.978920
Sum of electronic and thermal Enthalpies= -555.977976
Sum of electronic and thermal Free Energies= -556.029172

Isotropic Fermi Contact Couplings

	Atom	a.u.	MegaHertz	Gauss	10(-4) cm-1
1	C(13)	0.00104	1.17440	0.41906	0.39174

2	N(14)	-0.00248	-0.79969	-0.28535	-0.26675
3	C(13)	0.01191	13.38388	4.77570	4.46438
4	C(13)	-0.00247	-2.77840	-0.99140	-0.92677
5	C(13)	-0.01608	-18.07519	-6.44968	-6.02924
6	C(13)	0.00298	3.35570	1.19740	1.11934
7	H(1)	0.00038	1.69507	0.60484	0.56542
8	C(13)	0.01384	15.55988	5.55215	5.19022
9	C(13)	-0.00136	-1.53419	-0.54744	-0.51175
10	H(1)	-0.00066	-2.94048	-1.04924	-0.98084
11	H(1)	-0.00066	-2.96531	-1.05810	-0.98912
12	H(1)	0.00038	1.70980	0.61010	0.57033
13	C(13)	0.02975	33.44768	11.93496	11.15695
14	C(13)	-0.03093	-34.77251	-12.40770	-11.59886
15	C(13)	-0.02774	-31.18237	-11.12665	-10.40132
16	C(13)	0.03399	38.21157	13.63484	12.74601
17	H(1)	0.00214	9.56942	3.41461	3.19201
18	C(13)	0.02475	27.82931	9.93019	9.28286
19	H(1)	0.02878	**128.63663**	45.90075	42.90856
20	C(13)	-0.02824	-31.74921	-11.32891	-10.59040
21	H(1)	-0.00734	-32.82833	-11.71397	-10.95035
22	H(1)	-0.00549	-24.53337	-8.75412	-8.18345
23	H(1)	0.00189	8.43043	3.00819	2.81209
24	H(1)	0.02994	**133.83571**	47.75591	44.64279

Center Number	Atomic Number	Atomic Type	Coordinates (Angstroms) X	Y	Z
1	6	0	-0.708283	3.117629	-0.570100
2	7	0	-1.036830	2.015907	-0.332026
3	6	0	-1.513045	0.735888	-0.085202
4	6	0	-2.902545	0.574268	-0.063089
5	6	0	-0.632974	-0.364812	0.081044
6	6	0	-3.462985	-0.683494	0.109252
7	1	0	-3.525758	1.449211	-0.199649
8	6	0	-1.245845	-1.626009	0.225770
9	6	0	-2.624392	-1.788766	0.248586
10	1	0	-4.539937	-0.799155	0.125023
11	1	0	-0.618472	-2.498628	0.354192
12	1	0	-3.044633	-2.779122	0.381014
13	6	0	0.833373	-0.252067	0.085696
14	6	0	1.632806	-1.366974	-0.556812
15	6	0	1.497700	0.801259	0.682889
16	6	0	3.116866	-1.153035	-0.557703
17	1	0	1.421856	-2.329736	-0.059357
18	6	0	2.897761	0.905792	0.687196
19	1	0	0.928486	1.574545	1.181927
20	6	0	3.693455	-0.078648	0.039861
21	1	0	3.726479	-1.907211	-1.044091
22	1	0	3.369415	1.746826	1.179839
23	1	0	4.772426	0.033584	0.028273
24	1	0	1.276600	-1.527851	-1.587316

Atom 17 (Iso=0.113429,NMagM=8.890597)
Zero-point correction= 0.209636 (Hartree/Particle)
Thermal correction to Energy= 0.221075
Thermal correction to Enthalpy= 0.222019
Thermal correction to Gibbs Free Energy= 0.170848
Sum of electronic and zero-point Energies= -555.990174
Sum of electronic and thermal Energies= -555.978735
Sum of electronic and thermal Enthalpies= -555.977791
Sum of electronic and thermal Free Energies= -556.028962

Atom 24 (Iso=0.113429,NMagM=8.890597)
Zero-point correction= 0.209856 (Hartree/Particle)
Thermal correction to Energy= 0.221299
Thermal correction to Enthalpy= 0.222243
Thermal correction to Gibbs Free Energy= 0.171067
Sum of electronic and zero-point Energies= -555.989954
Sum of electronic and thermal Energies= -555.978511
Sum of electronic and thermal Enthalpies= -555.977567
Sum of electronic and thermal Free Energies= -556.028743

Isotropic Fermi Contact Couplings

	Atom	a.u.	MegaHertz	Gauss	10(-4) cm-1
1	C(13)	0.00046	0.51985	0.18550	0.17340
2	N(14)	-0.00379	-1.22531	-0.43722	-0.40872
3	C(13)	0.01247	14.01675	5.00153	4.67549
4	C(13)	-0.00428	-4.80682	-1.71519	-1.60338
5	C(13)	-0.01727	-19.41055	-6.92617	-6.47466
6	C(13)	0.00447	5.02654	1.79359	1.67667
7	H(1)	0.00050	2.22510	0.79397	0.74221
8	C(13)	0.00834	9.37683	3.34589	3.12777
9	C(13)	-0.00428	-4.81287	-1.71735	-1.60540
10	H(1)	-0.00093	-4.17745	-1.49062	-1.39345
11	H(1)	-0.00097	-4.32722	-1.54406	-1.44340
12	H(1)	0.00056	2.51132	0.89610	0.83769
13	C(13)	0.02970	33.38965	11.91426	11.13759
14	C(13)	-0.02739	-30.79609	-10.98881	-10.27247
15	C(13)	-0.03072	-34.53068	-12.32141	-11.51819
16	C(13)	0.02436	27.38777	9.77264	9.13558
17	H(1)	0.03038	**135.77641**	48.44840	45.29013
18	C(13)	0.03330	37.43452	13.35757	12.48681
19	H(1)	0.00219	9.78627	3.49198	3.26435
20	C(13)	-0.02767	-31.10314	-11.09837	-10.37489
21	H(1)	-0.00536	-23.94668	-8.54477	-7.98775
22	H(1)	-0.00720	-32.19747	-11.48886	-10.73992
23	H(1)	0.00187	8.35135	2.97997	2.78571
24	H(1)	0.02554	**114.15108**	40.73194	38.07670

Average A_p = 128.100 MHz
Isotope effect 1.20
Average A_μ = 489.3 MHz

8a-vi ub3lyp/6-311+g(d,p)

Center Number	Atomic Number	Atomic Type	Coordinates (Angstroms) X	Y	Z
1	6	0	-0.552162	3.066137	-0.614446
2	7	0	-0.969317	1.993323	-0.385737
3	6	0	-1.508074	0.740478	-0.126552
4	6	0	-2.902700	0.621527	-0.101442
5	6	0	-0.674142	-0.376697	0.076628
6	6	0	-3.490786	-0.615642	0.123230
7	1	0	-3.504747	1.505996	-0.267830
8	6	0	-1.300362	-1.611546	0.291295
9	6	0	-2.685504	-1.736826	0.318419
10	1	0	-4.570577	-0.703037	0.142906
11	1	0	-0.677422	-2.483122	0.455576
12	1	0	-3.135217	-2.706760	0.496512
13	6	0	0.813404	-0.293055	0.057277
14	6	0	1.538191	-1.151751	-0.819992
15	6	0	1.483410	0.563694	0.887776
16	6	0	2.953959	-1.120944	-0.837630
17	1	0	1.001233	-1.812570	-1.488836
18	6	0	2.978123	0.669211	0.906514
19	1	0	0.931531	1.204670	1.565829
20	6	0	3.660884	-0.277155	-0.033727
21	1	0	3.482121	-1.784427	-1.514869
22	1	0	3.276660	1.708719	0.678410
23	1	0	4.744985	-0.267159	-0.066455
24	1	0	3.351216	0.519848	1.934814

Atom 22 (Iso=0.113429,NMagM=8.890597)
Zero-point correction= 0.209061 (Hartree/Particle)
Thermal correction to Energy= 0.220546
Thermal correction to Enthalpy= 0.221490
Thermal correction to Gibbs Free Energy= 0.170386
Sum of electronic and zero-point Energies= -555.988270
Sum of electronic and thermal Energies= -555.976785
Sum of electronic and thermal Enthalpies= -555.975841
Sum of electronic and thermal Free Energies= -556.026944

Atom 24 (Iso=0.113429,NMagM=8.890597)
Zero-point correction= 0.209170 (Hartree/Particle)
Thermal correction to Energy= 0.220657
Thermal correction to Enthalpy= 0.221601
Thermal correction to Gibbs Free Energy= 0.170495
Sum of electronic and zero-point Energies= -555.988160
Sum of electronic and thermal Energies= -555.976674
Sum of electronic and thermal Enthalpies= -555.975730
Sum of electronic and thermal Free Energies= -556.026836

Isotropic Fermi Contact Couplings

	Atom	a.u.	MegaHertz	Gauss	10(-4) cm-1
1	C(13)	0.00087	0.97781	0.34891	0.32616

2	N(14)	0.00112	0.36275	0.12944	0.12100
3	C(13)	-0.00476	-5.34604	-1.90760	-1.78325
4	C(13)	0.00055	0.61307	0.21876	0.20450
5	C(13)	0.00493	5.54199	1.97752	1.84861
6	C(13)	-0.00063	-0.71330	-0.25452	-0.23793
7	H(1)	-0.00012	-0.52737	-0.18818	-0.17591
8	C(13)	-0.00467	-5.24454	-1.87138	-1.74939
9	C(13)	0.00028	0.31917	0.11389	0.10646
10	H(1)	0.00016	0.71576	0.25540	0.23875
11	H(1)	0.00025	1.11668	0.39846	0.37249
12	H(1)	-0.00010	-0.45666	-0.16295	-0.15233
13	C(13)	-0.03177	-35.71390	-12.74361	-11.91287
14	C(13)	0.03712	41.73417	14.89179	13.92102
15	C(13)	0.02789	31.35339	11.18767	10.45837
16	C(13)	-0.03063	-34.43051	-12.28566	-11.48478
17	H(1)	-0.00790	-35.31649	-12.60180	-11.78031
18	C(13)	-0.02883	-32.41323	-11.56585	-10.81189
19	H(1)	-0.00596	-26.62618	-9.50088	-8.88154
20	C(13)	0.02722	30.59821	10.91820	10.20646
21	H(1)	0.00207	9.23486	3.29523	3.08042
22	H(1)	0.03245	**145.03489**	51.75205	48.37843
23	H(1)	-0.00595	-26.58517	-9.48625	-8.86786
24	H(1)	0.02981	**133.25024**	47.54700	44.44749

Center Number	Atomic Number	Atomic Type	Coordinates (Angstroms) X	Y	Z
1	6	0	-0.525277	3.053541	-0.616829
2	7	0	-0.955840	1.986417	-0.384894
3	6	0	-1.505923	0.738483	-0.127715
4	6	0	-2.900396	0.625198	-0.108677
5	6	0	-0.676979	-0.382378	0.073827
6	6	0	-3.493148	-0.611534	0.110028
7	1	0	-3.498942	1.511977	-0.275529
8	6	0	-1.306307	-1.614816	0.287770
9	6	0	-2.692832	-1.735581	0.305952
10	1	0	-4.573297	-0.695397	0.125712
11	1	0	-0.685356	-2.486462	0.457746
12	1	0	-3.146749	-2.704274	0.480130
13	6	0	0.810820	-0.292523	0.061301
14	6	0	1.531115	-1.056414	-0.816827
15	6	0	1.479950	0.562379	0.984762
16	6	0	3.029608	-1.034917	-0.868456
17	1	0	1.013346	-1.693179	-1.526548
18	6	0	2.893666	0.626377	0.995596
19	1	0	0.906004	1.156913	1.683268
20	6	0	3.651560	-0.112852	0.136473
21	1	0	3.424213	-2.058704	-0.738431
22	1	0	3.379806	1.283155	1.709580
23	1	0	4.733756	-0.044748	0.161250
24	1	0	3.362967	-0.763979	-1.886156

Atom 21 (Iso=0.113429,NMagM=8.890597)
Zero-point correction= 0.209062 (Hartree/Particle)
Thermal correction to Energy= 0.220562
Thermal correction to Enthalpy= 0.221506
Thermal correction to Gibbs Free Energy= 0.170315
Sum of electronic and zero-point Energies= -555.988119
Sum of electronic and thermal Energies= -555.976618
Sum of electronic and thermal Enthalpies= -555.975674
Sum of electronic and thermal Free Energies= -556.026866

Atom 24 (Iso=0.113429,NMagM=8.890597)
Zero-point correction= 0.209092 (Hartree/Particle)
Thermal correction to Energy= 0.220594
Thermal correction to Enthalpy= 0.221538
Thermal correction to Gibbs Free Energy= 0.170346
Sum of electronic and zero-point Energies= -555.988088
Sum of electronic and thermal Energies= -555.976587
Sum of electronic and thermal Enthalpies= -555.975642
Sum of electronic and thermal Free Energies= -556.026835

Isotropic Fermi Contact Couplings

	Atom	a.u.	MegaHertz	Gauss	10(-4) cm-1
1	C(13)	0.00145	1.63556	0.58361	0.54556
2	N(14)	0.00116	0.37401	0.13345	0.12476
3	C(13)	-0.00494	-5.55274	-1.98136	-1.85219
4	C(13)	0.00047	0.52810	0.18844	0.17616
5	C(13)	0.00487	5.47950	1.95522	1.82776
6	C(13)	-0.00058	-0.64769	-0.23111	-0.21605
7	H(1)	-0.00011	-0.50766	-0.18114	-0.16934
8	C(13)	-0.00469	-5.27519	-1.88232	-1.75961
9	C(13)	0.00022	0.24649	0.08795	0.08222
10	H(1)	0.00014	0.60451	0.21570	0.20164
11	H(1)	0.00021	0.94833	0.33839	0.31633
12	H(1)	-0.00011	-0.48971	-0.17474	-0.16335
13	C(13)	-0.03169	-35.63050	-12.71385	-11.88506
14	C(13)	0.02708	30.44657	10.86409	10.15588
15	C(13)	0.03773	42.41519	15.13479	14.14818
16	C(13)	-0.02860	-32.15001	-11.47192	-10.72409
17	H(1)	-0.00579	-25.88218	-9.23541	-8.63337
18	C(13)	-0.03109	-34.94552	-12.46943	-11.65657
19	H(1)	-0.00800	-35.77540	-12.76555	-11.93339
20	C(13)	0.02765	31.08872	11.09323	10.37008
21	H(1)	0.03063	**136.91693**	48.85537	45.67057
22	H(1)	0.00209	9.33476	3.33087	3.11374
23	H(1)	-0.00604	-27.00338	-9.63548	-9.00736
24	H(1)	0.03100	**138.54394**	49.43592	46.21328

Average A_p = 138.4365 MHz
Isotope effect 1.20
Average A_μ = 528.8 MHz

8a-vii ub3lyp/6-311+g(d,p)

Center Number	Atomic Number	Atomic Type	Coordinates (Angstroms) X	Y	Z
1	6	0	-0.704250	3.101737	-0.586174
2	7	0	-1.050954	2.006833	-0.341922
3	6	0	-1.534357	0.730950	-0.089274
4	6	0	-2.923435	0.572465	-0.039523
5	6	0	-0.655392	-0.373139	0.050148
6	6	0	-3.481186	-0.686072	0.136680
7	1	0	-3.548350	1.448839	-0.158282
8	6	0	-1.264390	-1.635430	0.211758
9	6	0	-2.641773	-1.795055	0.256598
10	1	0	-4.557718	-0.800293	0.175332
11	1	0	-0.627245	-2.501654	0.341507
12	1	0	-3.062578	-2.783827	0.398815
13	6	0	0.806017	-0.264955	0.033461
14	6	0	1.592946	-1.279705	-0.602072
15	6	0	1.504829	0.795584	0.692994
16	6	0	2.950302	-1.247407	-0.615078
17	1	0	1.082020	-2.082288	-1.123022
18	6	0	2.861258	0.857558	0.724102
19	1	0	0.936623	1.556503	1.212029
20	6	0	3.731254	-0.156703	0.049417
21	1	0	3.503633	-2.021916	-1.135799
22	1	0	3.349157	1.666053	1.258058
23	1	0	4.443273	-0.586917	0.774757
24	1	0	4.386924	0.338701	-0.688167

Atom 23 (Iso=0.113429,NMagM=8.890597)
Zero-point correction= 0.209804 (Hartree/Particle)
Thermal correction to Energy= 0.221149
Thermal correction to Enthalpy= 0.222093
Thermal correction to Gibbs Free Energy= 0.171547
Sum of electronic and zero-point Energies= -555.991832
Sum of electronic and thermal Energies= -555.980487
Sum of electronic and thermal Enthalpies= -555.979543
Sum of electronic and thermal Free Energies= -556.030089

Atom 24 (Iso=0.113429,NMagM=8.890597)
Zero-point correction= 0.209755 (Hartree/Particle)
Thermal correction to Energy= 0.221099
Thermal correction to Enthalpy= 0.222043
Thermal correction to Gibbs Free Energy= 0.171498
Sum of electronic and zero-point Energies= -555.991882
Sum of electronic and thermal Energies= -555.980538
Sum of electronic and thermal Enthalpies= -555.979593
Sum of electronic and thermal Free Energies= -556.030138

Isotropic Fermi Contact Couplings

	Atom	a.u.	MegaHertz	Gauss	10(-4) cm-1
1	C(13)	0.00087	0.97502	0.34791	0.32523

2	N(14)	-0.00530	-1.71299	-0.61124	-0.57139
3	C(13)	0.01643	18.47283	6.59156	6.16187
4	C(13)	-0.00605	-6.80137	-2.42690	-2.26869
5	C(13)	-0.02207	-24.81165	-8.85341	-8.27628
6	C(13)	0.00622	6.99266	2.49516	2.33250
7	H(1)	0.00068	3.02957	1.08103	1.01056
8	C(13)	0.01617	18.18368	6.48839	6.06542
9	C(13)	-0.00482	-5.41338	-1.93163	-1.80571
10	H(1)	-0.00134	-5.98898	-2.13702	-1.99771
11	H(1)	-0.00125	-5.57928	-1.99083	-1.86105
12	H(1)	0.00068	3.01828	1.07700	1.00679
13	C(13)	0.03646	40.98742	14.62533	13.67193
14	C(13)	-0.02847	-32.00839	-11.42139	-10.67685
15	C(13)	-0.02854	-32.08608	-11.44911	-10.70277
16	C(13)	0.02289	25.73156	9.18166	8.58312
17	H(1)	0.00181	8.07486	2.88131	2.69348
18	C(13)	0.02319	26.07252	9.30332	8.69686
19	H(1)	0.00182	8.14946	2.90793	2.71837
20	C(13)	-0.02494	-28.03361	-10.00309	-9.35101
21	H(1)	-0.00509	-22.75387	-8.11915	-7.58987
22	H(1)	-0.00517	-23.11591	-8.24833	-7.71064
23	H(1)	0.02662	**118.98927**	42.45833	39.69055
24	H(1)	0.02771	**123.85023**	44.19284	41.31199

Average A_p = 121.4198 MHz
Isotope effect 1.20
Average A_μ = 463.8 MHz

Trans-5d-Mu ub3lyp/6-311+g(d,p)

Center Number	Atomic Number	Atomic Type	Coordinates (Angstroms) X	Y	Z
1	6	0	2.282455	2.884927	0.824643
2	1	0	1.845965	3.890219	0.818079
3	7	0	1.809691	1.921824	0.219081
4	6	0	2.287374	0.602962	0.073387
5	6	0	3.664198	0.344312	0.078888
6	6	0	1.358949	-0.444915	-0.098361
7	6	0	4.137926	-0.952164	-0.069106
8	1	0	4.346165	1.176108	0.208810
9	6	0	1.867462	-1.745649	-0.232650
10	6	0	3.234074	-2.003627	-0.220150
11	1	0	5.205391	-1.141223	-0.065803
12	1	0	1.169100	-2.562145	-0.375832
13	1	0	3.592708	-3.019315	-0.342378
14	6	0	-0.111187	-0.224053	-0.120876
15	6	0	-0.955242	-1.007093	0.670291
16	6	0	-0.706504	0.736089	-0.957646
17	6	0	-2.342506	-0.850711	0.644724
18	1	0	-0.526336	-1.745177	1.339097
19	6	0	-2.081169	0.897770	-1.000235
20	1	0	-0.081613	1.355039	-1.588761
21	6	0	-2.911912	0.106871	-0.196213
22	1	0	-2.955778	-1.471545	1.283901
23	1	0	-2.537939	1.631184	-1.653947
24	8	0	-4.251029	0.346488	-0.305935
25	6	0	-5.146366	-0.426875	0.481015
26	1	0	-4.966704	-0.280507	1.551712
27	1	0	-6.144789	-0.071250	0.231726
28	1	0	-5.071084	-1.493126	0.241047

Additional DFT calculations

Characterizing the muoniated imidoyl radical

a)

UB3LYP/EPR-II

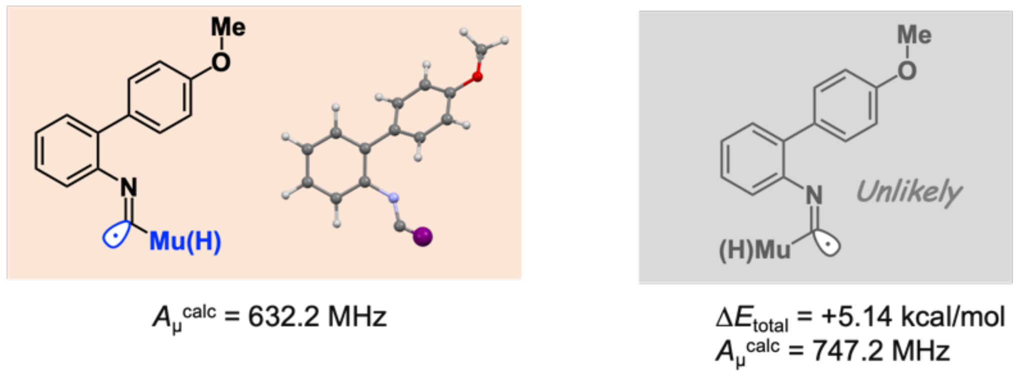

b)

UB3LYP/EPR-II

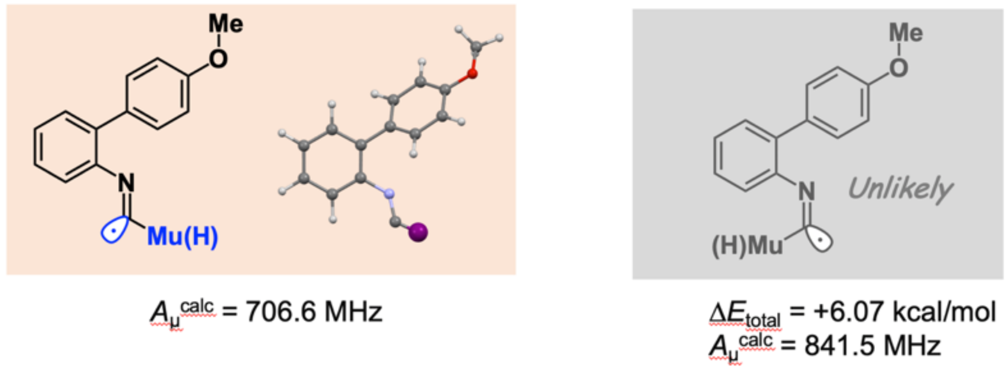

Figure S6. Additional DFT calculations for geometrical isomerism of the muoniated imidoyl radical. a) Muon isotope effect is not considered. b) The C–Mu(H) bonds are 5% elongated.

Characterizing the muoniated cyclohexadienyl radicals from 2d

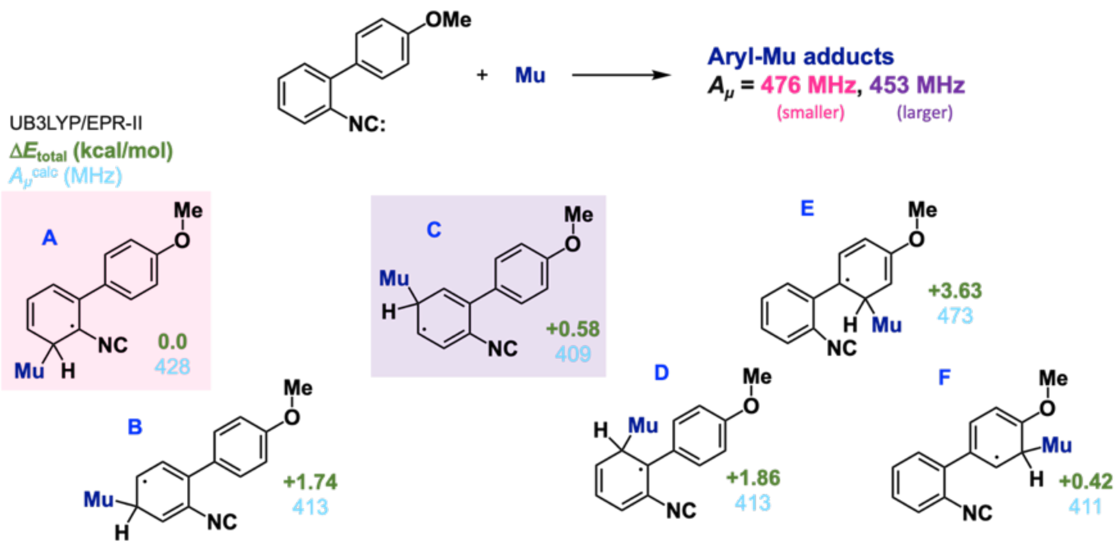

Figure S7. DFT calculations for muoniation at the aromatic ring in **2d**. Hydrogen atom was used in place of muonium. Each calculated muon hfc (A_μ) was obtained on average of two proton hyperfine coupling constants of the CH_2 unit and does not include the muon isotope effect. Although the most stable **A** (= **8d-i**) would be compatible with the experimentally determined A_μ of 476 MHz, the observed signal is smaller due to the substantially larger muonium. Accordingly, **C** (= **8d-iii**) could be assigned for the experimental A_μ of 453 MHz. Radical **F** is theoretically stable compared with **C**, but the steric hindrance of methoxy group would retard addition of muonium affording **C**.

Characterization of muonium addition pathways to 2-isocyano-1,1'-biphenyl

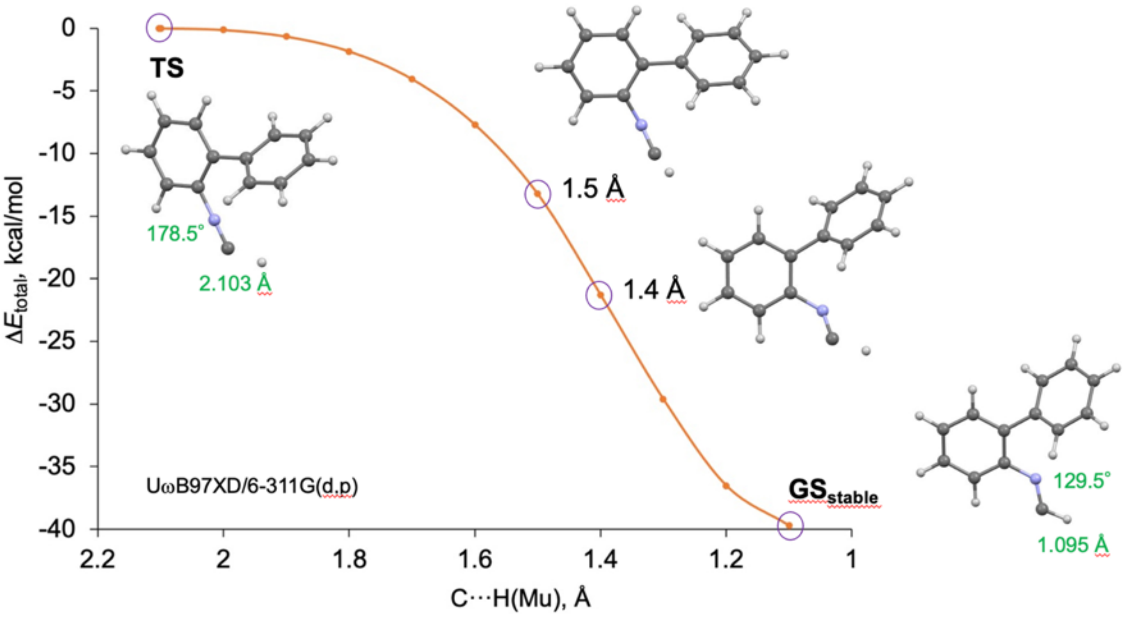

Figure S8. A PES scan for H radical addition to **2a** [UωB97XD/6-311G(d,p)].

Theoretical studies for *E*/*Z* isomerization of the muoniated imidoyl radical

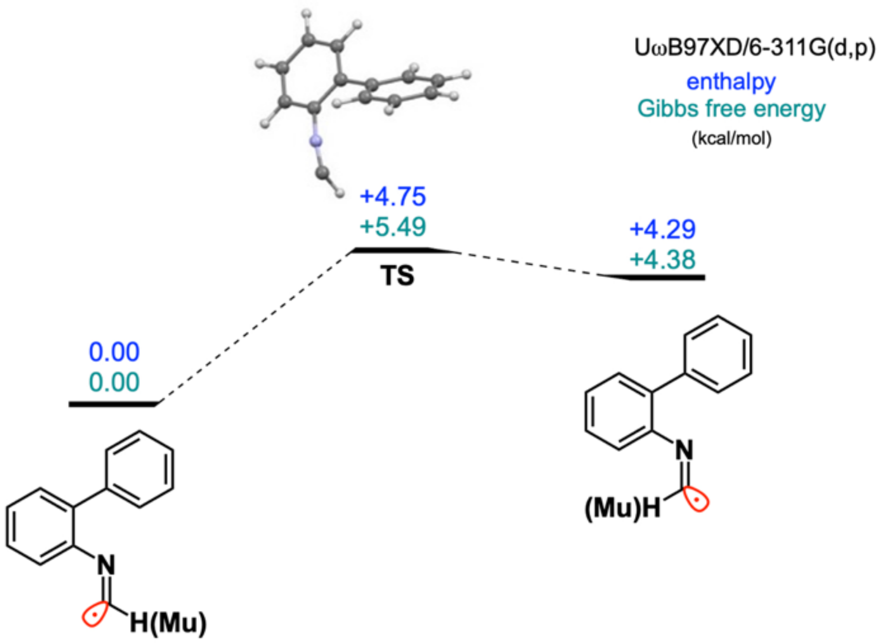

Figure S9. Isomerization from trans- to cis-imidoyl radical [UωB97XD/6-311G(d,p)].

Addition of muonium to 2-isocyano1,1'-biphenyl (2a): UωB97XD/6-311G(d,p)

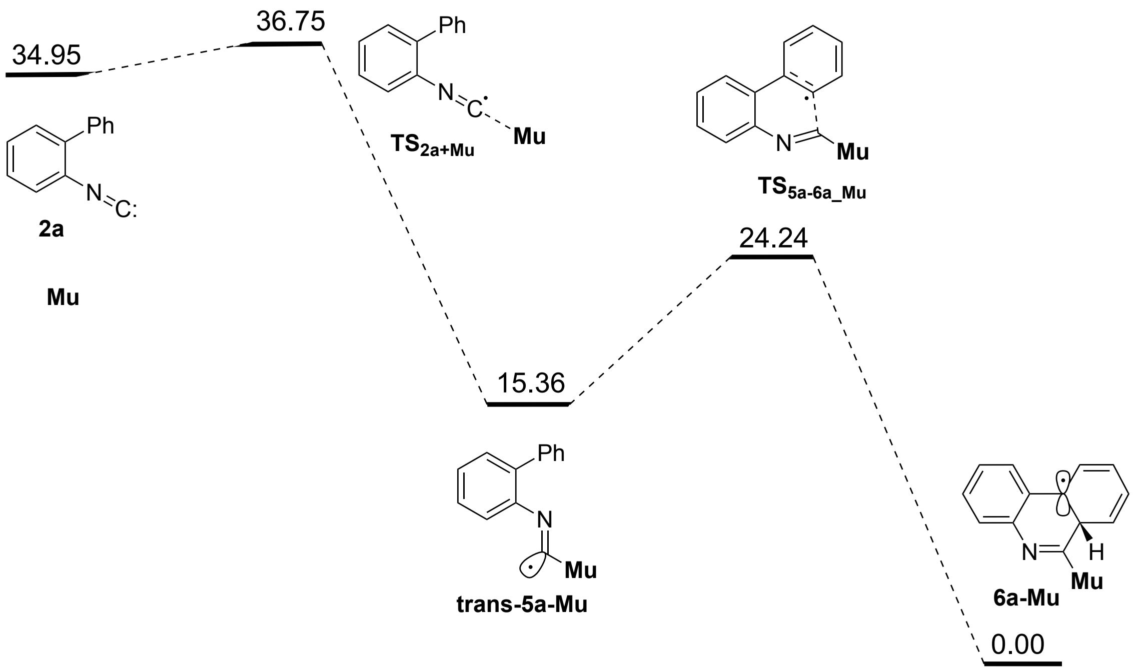

Figure S10. An enthalpy (Δ*H*, kcal/mol) diagram of the radical isonitrile insertion from **2a** and Mu leading to **6a-Mu** through imidoyl radical **trans-5a-Mu** [UωB97XD/6-311G(d,p)]. The C-Mu bond was emulated by 5% elongation from the equilibrium structure of the corresponding H isomer, and the vibrational frequencies and energies were computed with 0.1134 amu for muonium. The cis isomer **cis-5a-Mu** is 3.67 kcal/mol higher in energy.

Appx.

Trans-5d-Mu Conform 1, UB3LYP/EPR-II

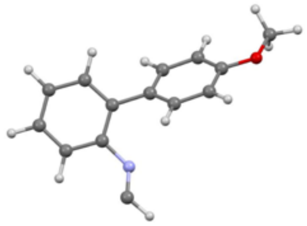

Center Number	Atomic Number	Atomic Type	Coordinates (Angstroms) X	Y	Z
1	6	0	1.352111	-0.456839	0.080341
2	6	0	1.855511	-1.757955	0.300503
3	6	0	3.227937	-2.029873	0.288141
4	6	0	4.145894	-0.990281	0.058645
5	6	0	3.676942	0.307153	-0.160585
6	6	0	2.292433	0.579413	-0.153926
7	1	0	1.151202	-2.558894	0.505962
8	1	0	3.580227	-3.042514	0.463606
9	1	0	5.213726	-1.190382	0.049467
10	1	0	4.363051	1.125701	-0.354618
11	7	0	1.815460	1.878422	-0.458363
12	1	0	-0.571103	-1.931081	-1.143116
13	6	0	-0.117568	-0.224305	0.099451
14	6	0	-0.696184	0.848543	0.815937
15	6	0	-2.077795	1.032840	0.853056
16	6	0	-2.932478	0.144165	0.168772
17	6	0	-2.378877	-0.932615	-0.547948
18	6	0	-0.985749	-1.105888	-0.570352
19	1	0	-0.057630	1.535244	1.361318
20	1	0	-2.518709	1.853341	1.410939
21	8	0	-4.270379	0.409939	0.260843
22	1	0	-3.008235	-1.628735	-1.090024
23	6	0	2.408572	2.970678	-0.424835
24	1	0	1.962196	3.921557	-0.739621
25	6	0	-5.170382	-0.464556	-0.409676
26	1	0	-4.996098	-0.467743	-1.492811
27	1	0	-6.170513	-0.078145	-0.208378
28	1	0	-5.095509	-1.489681	-0.026065

```
Zero-point correction=                           0.220205 (Hartree/Particle)
Thermal correction to Energy=                    0.234174
Thermal correction to Enthalpy=                  0.235119
Thermal correction to Gibbs Free Energy=         0.178076
Sum of electronic and zero-point Energies=           -670.474925
Sum of electronic and thermal Energies=              -670.460956
Sum of electronic and thermal Enthalpies=            -670.460012
Sum of electronic and thermal Free Energies=         -670.517055
```

Appx.

Trans-5d-Mu Conform 2, UB3LYP/EPR-II

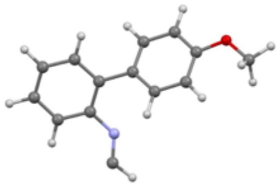

Center Number	Atomic Number	Atomic Type	Coordinates (Angstroms) X	Y	Z
1	6	0	1.386537	-0.448113	0.064138
2	6	0	2.011991	-1.686988	0.327108
3	6	0	3.403444	-1.809138	0.405606
4	6	0	4.217101	-0.676813	0.226611
5	6	0	3.626443	0.561984	-0.033348
6	6	0	2.223042	0.683366	-0.117745
7	1	0	1.386359	-2.559406	0.492102
8	1	0	3.850601	-2.777615	0.611542
9	1	0	5.298642	-0.760594	0.286906
10	1	0	4.231617	1.449365	-0.190548
11	7	0	1.632165	1.923057	-0.465971
12	1	0	-0.282947	-2.143392	-1.251960
13	6	0	-0.097825	-0.378283	-0.009849
14	6	0	-0.833348	0.625288	0.646692
15	6	0	-2.235465	0.660331	0.601044
16	6	0	-2.935195	-0.327493	-0.116614
17	6	0	-2.215093	-1.342349	-0.779246
18	6	0	-0.821601	-1.364052	-0.719934
19	1	0	-0.310641	1.385086	1.217909
20	1	0	-2.761489	1.447487	1.128341
21	8	0	-4.296251	-0.389016	-0.228703
22	1	0	-2.765771	-2.094160	-1.336283
23	6	0	2.096845	3.073926	-0.392066
24	1	0	1.576710	3.970211	-0.750911
25	6	0	-5.064998	0.616907	0.420962
26	1	0	-4.911805	0.601415	1.507169
27	1	0	-6.108340	0.384733	0.202659
28	1	0	-4.829361	1.616157	0.034338

```
Zero-point correction=                           0.220249 (Hartree/Particle)
Thermal correction to Energy=                    0.234191
Thermal correction to Enthalpy=                  0.235135
Thermal correction to Gibbs Free Energy=         0.178193
Sum of electronic and zero-point Energies=           -670.474943
Sum of electronic and thermal Energies=              -670.461001
Sum of electronic and thermal Enthalpies=            -670.460057
Sum of electronic and thermal Free Energies=         -670.516999
```

Appx.

Cis-5d-Mu Conform 1, UB3LYP/EPR-II

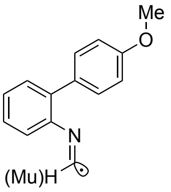

Center Number	Atomic Number	Atomic Type	Coordinates (Angstroms) X	Y	Z
1	6	0	1.342693	-0.453192	0.081641
2	6	0	1.843124	-1.746724	0.346503
3	6	0	3.213875	-2.029737	0.325069
4	6	0	4.134300	-1.007598	0.040475
5	6	0	3.669050	0.285898	-0.216297
6	6	0	2.287568	0.569339	-0.193322
7	1	0	1.136927	-2.534951	0.591029
8	1	0	3.561455	-3.037951	0.531852
9	1	0	5.200268	-1.215678	0.015173
10	1	0	4.364896	1.082068	-0.464400
11	7	0	1.833688	1.869277	-0.537551
12	1	0	-0.586956	-1.985163	-1.049087
13	6	0	-0.125313	-0.214203	0.097857
14	6	0	-0.699652	0.898749	0.753745
15	6	0	-2.080816	1.087317	0.785074
16	6	0	-2.939439	0.163404	0.155060
17	6	0	-2.390116	-0.952879	-0.502493
18	6	0	-0.997870	-1.129564	-0.520028
19	1	0	-0.060167	1.616404	1.255807
20	1	0	-2.517712	1.940600	1.294710
21	8	0	-4.276321	0.436156	0.236426
22	1	0	-3.022427	-1.676352	-1.003736
23	6	0	2.317790	2.993305	-0.401716
24	1	0	3.219159	3.345267	0.126770
25	6	0	-5.179823	-0.469816	-0.385930
26	1	0	-5.006872	-0.530507	-1.467561
27	1	0	-6.178490	-0.070212	-0.204114
28	1	0	-5.107545	-1.473503	0.051178

Zero-point correction= 0.219821 (Hartree/Particle)
Thermal correction to Energy= 0.233795
Thermal correction to Enthalpy= 0.234739
Thermal correction to Gibbs Free Energy= 0.177865
Sum of electronic and zero-point Energies= -670.467110
Sum of electronic and thermal Energies= -670.453136
Sum of electronic and thermal Enthalpies= -670.452192
Sum of electronic and thermal Free Energies= -670.509066

Appx.

Cis-5d-Mu Conform 2, UB3LYP/EPR-II

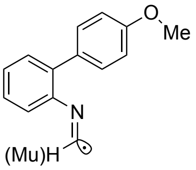

Center Number	Atomic Number	Atomic Type	Coordinates (Angstroms) X	Y	Z
1	6	0	1.377991	-0.445871	0.066158
2	6	0	2.003931	-1.675839	0.366260
3	6	0	3.396327	-1.804233	0.425290
4	6	0	4.212151	-0.685577	0.188047
5	6	0	3.621226	0.548033	-0.101840
6	6	0	2.217617	0.675998	-0.159293
7	1	0	1.377406	-2.538687	0.572849
8	1	0	3.841479	-2.767687	0.656871
9	1	0	5.294354	-0.773635	0.224083
10	1	0	4.237361	1.416897	-0.314325
11	7	0	1.644235	1.918805	-0.534792
12	1	0	-0.298244	-2.213446	-1.132863
13	6	0	-0.105975	-0.375714	-0.000939
14	6	0	-0.838625	0.668456	0.592417
15	6	0	-2.240726	0.702778	0.547813
16	6	0	-2.943839	-0.326684	-0.104534
17	6	0	-2.226952	-1.382598	-0.703885
18	6	0	-0.833846	-1.402448	-0.647162
19	1	0	-0.316114	1.463632	1.112463
20	1	0	-2.763798	1.524155	1.023237
21	8	0	-4.304849	-0.392332	-0.210079
22	1	0	-2.780035	-2.166139	-1.212702
23	6	0	1.993391	3.089134	-0.377713
24	1	0	2.820331	3.536859	0.198000
25	6	0	-5.069846	0.658641	0.369377
26	1	0	-4.918030	0.715333	1.454400
27	1	0	-6.113942	0.417019	0.165470
28	1	0	-4.828566	1.628254	-0.083286

```
Zero-point correction=                           0.219820 (Hartree/Particle)
Thermal correction to Energy=                    0.233786
Thermal correction to Enthalpy=                  0.234730
Thermal correction to Gibbs Free Energy=         0.177895
Sum of electronic and zero-point Energies=          -670.467293
Sum of electronic and thermal Energies=             -670.453327
Sum of electronic and thermal Enthalpies=           -670.452383
Sum of electronic and thermal Free Energies=        -670.509218
```

Appx.

Isonitrile-aromaticMu(H) (**8d-i**), UB3LYP/EPR-II

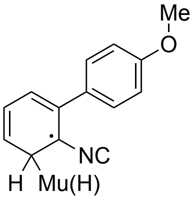

Center Number	Atomic Number	Atomic Type	Coordinates (Angstroms) X	Y	Z
1	6	0	1.371127	-0.379129	0.089671
2	6	0	1.903428	-1.683186	0.296631
3	6	0	3.308890	-1.937584	0.270811
4	6	0	4.214349	-0.931255	0.063218
5	6	0	3.773576	0.489785	-0.151053
6	6	0	2.268741	0.665419	-0.144259
7	1	0	1.215154	-2.495440	0.505047
8	1	0	3.659149	-2.954068	0.430643
9	1	0	5.281734	-1.133719	0.053462
10	1	0	4.218904	1.147064	0.615951
11	7	0	1.846308	1.946601	-0.419461
12	1	0	-0.503916	-1.864818	-1.189846
13	6	0	-0.106375	-0.188348	0.112560
14	6	0	-0.714869	0.842764	0.863395
15	6	0	-2.101034	0.983670	0.904032
16	6	0	-2.927708	0.095070	0.185442
17	6	0	-2.341612	-0.938148	-0.569435
18	6	0	-0.944535	-1.070753	-0.593004
19	1	0	-0.099523	1.532434	1.431714
20	1	0	-2.566355	1.772774	1.486368
21	8	0	-4.271317	0.316454	0.284884
22	1	0	-2.949501	-1.631796	-1.138278
23	6	0	1.606198	3.083653	-0.669686
24	1	0	-5.045488	-1.589645	-0.089557
25	6	0	-5.145817	-0.551074	-0.428359
26	1	0	-4.967050	-0.500962	-1.509438
27	1	0	-6.156135	-0.199714	-0.214829
28	1	0	4.173255	0.874740	-1.103865

Zero-point correction=	0.219356	(Hartree/Particle)
Thermal correction to Energy=	0.233588	
Thermal correction to Enthalpy=	0.234532	
Thermal correction to Gibbs Free Energy=	0.177085	
Sum of electronic and zero-point Energies=	-670.463979	
Sum of electronic and thermal Energies=	-670.449748	
Sum of electronic and thermal Enthalpies=	-670.448803	
Sum of electronic and thermal Free Energies=	-670.506251	

Appx.

Isonitrile-aromaticMu(H) (**8d-ii**), UB3LYP/EPR-II

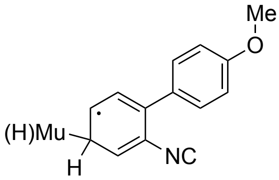

Center Number	Atomic Number	Atomic Type	Coordinates (Angstroms) X	Y	Z
1	6	0	1.357075	-0.357345	0.064650
2	6	0	1.907380	-1.680133	0.217121
3	6	0	3.247281	-1.948478	0.235367
4	6	0	4.285441	-0.868339	0.093651
5	6	0	3.682495	0.491383	-0.114964
6	6	0	2.323056	0.703894	-0.122207
7	1	0	1.209248	-2.499038	0.364947
8	1	0	3.592655	-2.968581	0.380759
9	1	0	4.941908	-0.856544	0.982276
10	1	0	4.343840	1.334341	-0.288145
11	7	0	1.890248	2.007285	-0.380690
12	1	0	-0.522714	-1.995727	-0.992344
13	6	0	-0.100423	-0.163600	0.084202
14	6	0	-0.720139	0.954903	0.702526
15	6	0	-2.105052	1.089348	0.741009
16	6	0	-2.935996	0.109479	0.157472
17	6	0	-2.348874	-1.011430	-0.460578
18	6	0	-0.953913	-1.136495	-0.486965
19	1	0	-0.112977	1.715040	1.179406
20	1	0	-2.568178	1.943868	1.224518
21	8	0	-4.279185	0.334189	0.243595
22	1	0	-2.957294	-1.777400	-0.927109
23	6	0	1.569057	3.121738	-0.616912
24	1	0	-5.040927	-1.610211	0.138196
25	6	0	-5.156308	-0.626885	-0.334079
26	1	0	-4.993882	-0.719521	-1.414924
27	1	0	-6.166497	-0.257467	-0.153018
28	1	0	4.970085	-1.101500	-0.740248

Zero-point correction= 0.219616 (Hartree/Particle)
Thermal correction to Energy= 0.233761
Thermal correction to Enthalpy= 0.234705
Thermal correction to Gibbs Free Energy= 0.177617
Sum of electronic and zero-point Energies= -670.460941
Sum of electronic and thermal Energies= -670.446796
Sum of electronic and thermal Enthalpies= -670.445852
Sum of electronic and thermal Free Energies= -670.502940

Appx.

Isonitrile-aromaticMu(H) (**8d-iii**), UB3LYP/EPR-II

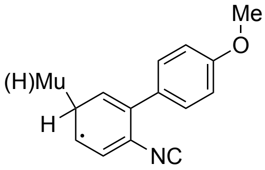

Center Number	Atomic Number	Atomic Type	Coordinates (Angstroms) X	Y	Z
1	6	0	1.385852	-0.371403	0.077117
2	6	0	1.936100	-1.616283	0.280527
3	6	0	3.413666	-1.902834	0.262044
4	6	0	4.262238	-0.679882	0.046764
5	6	0	3.708243	0.551304	-0.154813
6	6	0	2.283613	0.738263	-0.162952
7	1	0	1.271317	-2.450127	0.489757
8	1	0	3.705502	-2.401029	1.202578
9	1	0	5.342580	-0.796079	0.046899
10	1	0	4.334519	1.422162	-0.323938
11	7	0	1.805023	1.994909	-0.437937
12	1	0	-0.479217	-1.782332	-1.302263
13	6	0	-0.094493	-0.190739	0.102405
14	6	0	-0.707033	0.791324	0.913214
15	6	0	-2.094143	0.917019	0.968578
16	6	0	-2.916481	0.063148	0.203910
17	6	0	-2.325814	-0.916753	-0.614811
18	6	0	-0.926854	-1.033294	-0.654628
19	1	0	-0.094080	1.454722	1.515236
20	1	0	-2.563729	1.668250	1.595848
21	8	0	-4.261933	0.266523	0.323318
22	1	0	-2.930739	-1.579186	-1.222610
23	6	0	1.434441	3.098729	-0.683081
24	1	0	-5.020046	-1.619523	-0.167975
25	6	0	-5.132422	-0.562279	-0.438605
26	1	0	-4.961369	-0.441258	-1.515382
27	1	0	-6.144793	-0.235644	-0.197136
28	1	0	3.638877	-2.654409	-0.516011

```
Zero-point correction=                           0.219397 (Hartree/Particle)
Thermal correction to Energy=                    0.233602
Thermal correction to Enthalpy=                  0.234547
Thermal correction to Gibbs Free Energy=         0.177125
Sum of electronic and zero-point Energies=           -670.463019
Sum of electronic and thermal Energies=              -670.448814
Sum of electronic and thermal Enthalpies=            -670.447870
Sum of electronic and thermal Free Energies=         -670.505291
```

Appx.

Isonitrile-aromaticMu(H) (**8d-iv**), UB3LYP/EPR-II

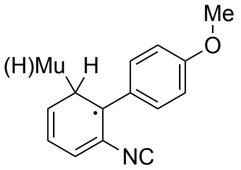

Center Number	Atomic Number	Atomic Type	Coordinates (Angstroms) X	Y	Z
1	6	0	1.375852	-0.333427	0.014044
2	6	0	1.891533	-1.761816	0.129776
3	6	0	3.383205	-1.915367	0.253754
4	6	0	4.233263	-0.846025	0.149156
5	6	0	3.725607	0.465341	-0.068564
6	6	0	2.319503	0.687603	-0.125392
7	1	0	1.391428	-2.252232	0.979552
8	1	0	3.770560	-2.919121	0.406385
9	1	0	5.307612	-0.989852	0.225733
10	1	0	4.390171	1.312756	-0.189642
11	7	0	1.935417	2.009763	-0.359195
12	1	0	-0.534823	-2.044749	-0.896848
13	6	0	-0.084048	-0.142079	0.046291
14	6	0	-0.697984	1.005290	0.618319
15	6	0	-2.081421	1.142509	0.670015
16	6	0	-2.921400	0.135954	0.147472
17	6	0	-2.342642	-1.017967	-0.413075
18	6	0	-0.947800	-1.147064	-0.447980
19	1	0	-0.086629	1.787932	1.049136
20	1	0	-2.536892	2.020368	1.117605
21	8	0	-4.261889	0.367200	0.238918
22	1	0	-2.957045	-1.811418	-0.822001
23	6	0	1.700614	3.149267	-0.578923
24	1	0	-5.032870	-1.576662	0.244396
25	6	0	-5.148973	-0.619459	-0.278465
26	1	0	-4.997454	-0.769557	-1.354334
27	1	0	-6.155571	-0.236068	-0.107136
28	1	0	1.556861	-2.343891	-0.748396

```
Zero-point correction=                           0.219653 (Hartree/Particle)
Thermal correction to Energy=                    0.233824
Thermal correction to Enthalpy=                  0.234768
Thermal correction to Gibbs Free Energy=         0.177437
Sum of electronic and zero-point Energies=           -670.460714
Sum of electronic and thermal Energies=              -670.446544
Sum of electronic and thermal Enthalpies=            -670.445599
Sum of electronic and thermal Free Energies=         -670.502930
```

Appx.

Isonitrile-aromaticMu(H) (**8d-v**), UB3LYP/EPR-II

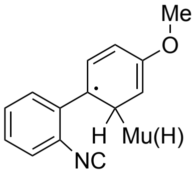

Center Number	Atomic Number	Atomic Type	Coordinates (Angstroms) X	Y	Z
1	6	0	1.415865	-0.376801	0.047076
2	6	0	1.916130	-1.685204	0.259436
3	6	0	3.285108	-1.968921	0.281840
4	6	0	4.222337	-0.937843	0.100972
5	6	0	3.770117	0.366003	-0.117293
6	6	0	2.389344	0.641465	-0.151172
7	1	0	1.202240	-2.482393	0.441356
8	1	0	3.620296	-2.987203	0.456781
9	1	0	5.287757	-1.144580	0.127809
10	1	0	4.467020	1.181753	-0.279415
11	7	0	2.015534	1.948921	-0.450272
12	1	0	-0.453281	-1.850615	-1.164959
13	6	0	-0.046212	-0.148190	0.054478
14	6	0	-0.642879	0.989761	0.858805
15	6	0	-2.144411	1.037602	0.861449
16	6	0	-2.920091	0.114539	0.187365
17	6	0	-2.304692	-0.939297	-0.550980
18	6	0	-0.888854	-1.040953	-0.584250
19	1	0	-0.258029	1.959155	0.511674
20	1	0	-2.635133	1.838735	1.406102
21	8	0	-4.279646	0.279463	0.269074
22	1	0	-2.896131	-1.660992	-1.100027
23	6	0	1.750355	3.069170	-0.728522
24	1	0	-4.986188	-1.661861	-0.071287
25	6	0	-5.114691	-0.633738	-0.432370
26	1	0	-4.932894	-0.601741	-1.513827
27	1	0	-6.138638	-0.313094	-0.234330
28	1	0	-0.273141	0.919128	1.898421

Zero-point correction= 0.219191 (Hartree/Particle)
Thermal correction to Energy= 0.233491
Thermal correction to Enthalpy= 0.234436
Thermal correction to Gibbs Free Energy= 0.176525
Sum of electronic and zero-point Energies= -670.458361
Sum of electronic and thermal Energies= -670.444061
Sum of electronic and thermal Enthalpies= -670.443117
Sum of electronic and thermal Free Energies= -670.501028

Appx.

Isonitrile-aromaticMu(H) (**8d-vi**), UB3LYP/EPR-II

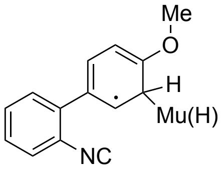

Center Number	Atomic Number	Atomic Type	Coordinates (Angstroms) X	Y	Z
1	6	0	1.448421	-0.380625	0.093541
2	6	0	1.991245	-1.661634	0.322362
3	6	0	3.371798	-1.895902	0.302111
4	6	0	4.260217	-0.838988	0.046245
5	6	0	3.755789	0.442311	-0.194514
6	6	0	2.365257	0.666891	-0.175548
7	1	0	1.308316	-2.478089	0.537863
8	1	0	3.752275	-2.895484	0.491431
9	1	0	5.332563	-1.008828	0.031450
10	1	0	4.417583	1.275515	-0.406858
11	7	0	1.919087	1.955325	-0.457656
12	1	0	-0.402621	-1.813093	-1.278178
13	6	0	-0.030669	-0.184930	0.126201
14	6	0	-0.603874	0.793600	0.913881
15	6	0	-2.092041	1.015177	0.982205
16	6	0	-2.881766	0.036083	0.152726
17	6	0	-2.280325	-0.936038	-0.614738
18	6	0	-0.858232	-1.060840	-0.642824
19	1	0	0.018358	1.447598	1.516409
20	1	0	-2.446431	0.965097	2.025378
21	8	0	-4.219883	0.251808	0.262478
22	1	0	-2.875151	-1.616466	-1.213824
23	6	0	1.577868	3.060751	-0.709310
24	1	0	-4.982884	-1.641026	-0.178989
25	6	0	-5.095746	-0.592773	-0.480277
26	1	0	-4.913325	-0.500357	-1.557442
27	1	0	-6.107049	-0.254622	-0.251638
28	1	0	-2.343824	2.039514	0.655796

Zero-point correction= 0.219546 (Hartree/Particle)
Thermal correction to Energy= 0.233768
Thermal correction to Enthalpy= 0.234712
Thermal correction to Gibbs Free Energy= 0.177227
Sum of electronic and zero-point Energies= -670.463114
Sum of electronic and thermal Energies= -670.448892
Sum of electronic and thermal Enthalpies= -670.447948
Sum of electronic and thermal Free Energies= -670.505433

Isonitrile (**2a**), RωB97XD/6-311G(d,p)

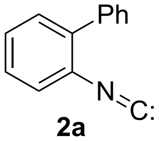

2a

Center Number	Atomic Number	Atomic Type	Coordinates (Angstroms) X	Y	Z
1	6	0	0.636083	-0.376771	0.076191
2	6	0	1.263192	-1.606469	0.283739
3	6	0	2.645117	-1.724958	0.299467
4	6	0	3.441401	-0.602938	0.104914
5	6	0	2.848957	0.630406	-0.108060
6	6	0	1.460034	0.740180	-0.123072
7	1	0	0.643116	-2.479720	0.451617
8	1	0	3.100949	-2.693220	0.469290
9	1	0	4.521397	-0.686831	0.117689
10	1	0	3.444011	1.519843	-0.273103
11	7	0	0.906547	1.988687	-0.367590
12	1	0	-1.051295	-1.836456	-1.420512
13	6	0	-0.846281	-0.293916	0.058929
14	6	0	-1.532436	0.601175	0.880616
15	6	0	-2.919332	0.641288	0.872682
16	6	0	-3.639629	-0.209427	0.042742
17	6	0	-2.965161	-1.104061	-0.778371
18	6	0	-1.577490	-1.147299	-0.768655
19	1	0	-0.977453	1.264569	1.533486
20	1	0	-3.439409	1.339901	1.517823
21	1	0	-4.722968	-0.172767	0.034467
22	1	0	-3.520210	-1.766456	-1.432760
23	6	0	0.461553	3.051178	-0.578600

Temperature 298.150 Kelvin. Pressure 1.00000 Atm
Zero-point correction= 0.180868 (Hartree/Particle)
Thermal correction to Energy= 0.191621
Thermal correction to Enthalpy= 0.192565
Thermal correction to Gibbs Free Energy= 0.143742
Sum of electronic and zero-point Energies= -555.260110
Sum of electronic and thermal Energies= -555.249357
Sum of electronic and thermal Enthalpies= -555.248413
Sum of electronic and thermal Free Energies= -555.297237

TS$_{2a+Mu}$, UωB97XD/6-311G(d,p)

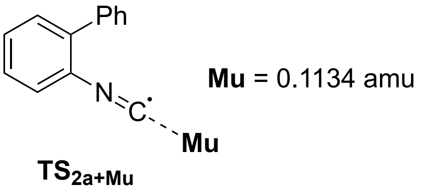

Center Number	Atomic Number	Atomic Type	Coordinates (Angstroms) X	Y	Z	
1	6	0	-0.630138	-0.423134	-0.076155	
2	6	0	-1.252187	-1.665940	-0.205627	
3	6	0	-2.633584	-1.790775	-0.218798	
4	6	0	-3.435160	-0.661691	-0.101467	
5	6	0	-2.848729	0.585152	0.034272	
6	6	0	-1.459878	0.700981	0.047894	
7	1	0	-0.628308	-2.545847	-0.313978	
8	1	0	-3.085047	-2.769677	-0.328571	
9	1	0	-4.514729	-0.750698	-0.113461	
10	1	0	-3.448121	1.480836	0.138662	
11	7	0	-0.913213	1.963075	0.214241	
12	1	0	1.063555	-1.791969	1.498226	
13	6	0	0.851622	-0.332512	-0.062545	
14	6	0	1.534146	0.519482	-0.931852	
15	6	0	2.920836	0.566049	-0.926194	
16	6	0	3.644679	-0.235746	-0.051878	
17	6	0	2.973982	-1.087474	0.816580	
18	6	0	1.586553	-1.136821	0.809779	
19	1	0	0.976735	1.144222	-1.619888	
20	1	0	3.437901	1.231185	-1.608067	
21	1	0	4.727837	-0.194124	-0.045945	
22	1	0	3.531760	-1.711838	1.505131	
23	6	0	-0.475545	3.039119	0.370652	
24	1	0	-0.328682	3.706246	2.360244	0.1134 amu

Imaginary = -1426.2260 cm^{-1}

```
Temperature   298.150 Kelvin.  Pressure   1.00000 Atm
Zero-point correction=                           0.182529 (Hartree/Particle)
Thermal correction to Energy=                    0.194114
Thermal correction to Enthalpy=                  0.195058
Thermal correction to Gibbs Free Energy=         0.144021
Sum of electronic and zero-point Energies=           -555.756940
Sum of electronic and thermal Energies=              -555.745355
Sum of electronic and thermal Enthalpies=            -555.744411
Sum of electronic and thermal Free Energies=         -555.795447
```

trans-5a-Mu, UωB97XD/6-311G(d,p)

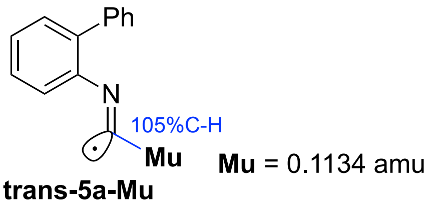

Center Number	Atomic Number	Atomic Type	Coordinates (Angstroms) X	Y	Z	
1	6	0	0.566083	-0.454037	0.071827	
2	6	0	1.128083	-1.708247	0.320356	
3	6	0	2.501647	-1.896522	0.335503	
4	6	0	3.349616	-0.819891	0.101824	
5	6	0	2.816570	0.434401	-0.142432	
6	6	0	1.434167	0.623324	-0.155775	
7	1	0	0.464988	-2.543328	0.517933	
8	1	0	2.910809	-2.880991	0.530383	
9	1	0	4.424755	-0.957167	0.109191	
10	1	0	3.457939	1.285782	-0.338278	
11	7	0	0.886383	1.883664	-0.475460	
12	1	0	-1.204605	-1.919001	-1.324398	
13	6	0	-0.910506	-0.297960	0.051793	
14	6	0	-1.548463	0.683718	0.811954	
15	6	0	-2.931292	0.799248	0.798993	
16	6	0	-3.698759	-0.061762	0.023494	
17	6	0	-3.074288	-1.043692	-0.735100	
18	6	0	-1.690794	-1.161703	-0.718629	
19	1	0	-0.955817	1.351961	1.425145	
20	1	0	-3.411936	1.563864	1.398913	
21	1	0	-4.778635	0.033231	0.010510	
22	1	0	-3.665038	-1.715805	-1.347258	
23	6	0	1.342809	3.005868	-0.268739	
24	1	0	0.843621	3.979327	-0.624365	0.1134 amu

Temperature 298.150 Kelvin. Pressure 1.00000 Atm
Zero-point correction= 0.209532 (Hartree/Particle)
Thermal correction to Energy= 0.220305
Thermal correction to Enthalpy= 0.221249
Thermal correction to Gibbs Free Energy= 0.171566
Sum of electronic and zero-point Energies= -555.791653
Sum of electronic and thermal Energies= -555.780880
Sum of electronic and thermal Enthalpies= -555.779936
Sum of electronic and thermal Free Energies= -555.829619

$Ts_{5a\text{-}6a\_Mu}$, UωB97XD/6-311G(d,p)

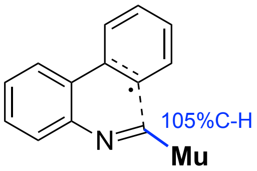

$TS_{5a\text{-}6a\_Mu}$

Mu = 0.1134 amu

Center Number	Atomic Number	Atomic Type	Coordinates (Angstroms) X	Y	Z	
1	6	0	0.745676	-0.403379	0.116961	
2	6	0	1.543623	-1.522529	0.352704	
3	6	0	2.928026	-1.432624	0.322428	
4	6	0	3.539312	-0.216232	0.036449	
5	6	0	2.763820	0.898837	-0.239996	
6	6	0	1.374502	0.818247	-0.195722	
7	1	0	1.062732	-2.466200	0.587111	
8	1	0	3.529799	-2.311137	0.524718	
9	1	0	4.620219	-0.140430	0.011962	
10	1	0	3.215682	1.846944	-0.506147	
11	7	0	0.632931	1.954681	-0.586473	
12	1	0	-0.900802	-2.060182	-1.262388	
13	6	0	-0.726814	-0.459044	0.141590	
14	6	0	-1.420217	0.540020	0.879185	
15	6	0	-2.833608	0.566835	0.830080	
16	6	0	-3.519477	-0.301439	0.003991	
17	6	0	-2.830193	-1.261023	-0.741559	
18	6	0	-1.441609	-1.333171	-0.665394	
19	1	0	-0.920877	0.994879	1.728325	
20	1	0	-3.374013	1.276846	1.445867	
21	1	0	-4.601765	-0.255197	-0.047459	
22	1	0	-3.374106	-1.946524	-1.380561	
23	6	0	-0.536915	2.166617	-0.234814	
24	1	0	-1.204149	3.011543	-0.631525	0.1134 amu

Imaginary = -507.8548 cm^{-1}

Temperature 298.150 Kelvin. Pressure 1.00000 Atm
Zero-point correction= 0.209211 (Hartree/Particle)
Thermal correction to Energy= 0.218968
Thermal correction to Enthalpy= 0.219912
Thermal correction to Gibbs Free Energy= 0.173169
Sum of electronic and zero-point Energies= -555.776500
Sum of electronic and thermal Energies= -555.766743
Sum of electronic and thermal Enthalpies= -555.765798
Sum of electronic and thermal Free Energies= -555.812542

6a-H), UωB97XD/6-311G(d,p)

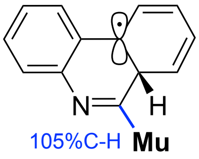

6a-Mu

Mu = 0.1134 amu

Center Number	Atomic Number	Atomic Type	Coordinates (Angstroms) X	Y	Z	
1	6	0	0.757986	-0.417414	0.067925	
2	6	0	1.580573	-1.545425	0.187882	
3	6	0	2.958981	-1.428355	0.146707	
4	6	0	3.551360	-0.178730	-0.027422	
5	6	0	2.755139	0.946452	-0.166403	
6	6	0	1.367206	0.842950	-0.115436	
7	1	0	1.130707	-2.518976	0.347060	
8	1	0	3.576992	-2.312152	0.256421	
9	1	0	4.630682	-0.086413	-0.061340	
10	1	0	3.184177	1.928352	-0.326222	
11	7	0	0.623221	2.022573	-0.327533	
12	1	0	-0.953091	-2.504349	-0.475791	
13	6	0	-0.693788	-0.469340	0.100936	
14	6	0	-1.364802	0.814489	0.505908	
15	6	0	-2.837685	0.845010	0.237846	
16	6	0	-3.517245	-0.274564	-0.097370	
17	6	0	-2.844740	-1.515561	-0.285062	
18	6	0	-1.450897	-1.575958	-0.216335	
19	1	0	-1.227226	0.918437	1.606366	
20	1	0	-3.351647	1.792739	0.363635	
21	1	0	-4.589775	-0.227184	-0.251636	
22	1	0	-3.410631	-2.398955	-0.553607	
23	6	0	-0.618314	2.004036	-0.066461	
24	1	0	-1.215374	2.964955	-0.248468	0.1134 amu

Temperature 298.150 Kelvin. Pressure 1.00000 Atm
Zero-point correction= 0.213668 (Hartree/Particle)
Thermal correction to Energy= 0.223266
Thermal correction to Enthalpy= 0.224210
Thermal correction to Gibbs Free Energy= 0.177807
Sum of electronic and zero-point Energies= -555.814962
Sum of electronic and thermal Energies= -555.805365
Sum of electronic and thermal Enthalpies= -555.804421
Sum of electronic and thermal Free Energies= -555.850823

A DFT study on H-imidoyl radical trans to cis isomerism (Reference of Mu-imidoyl radical)

H-imidoyl: Cis form, UwB97XD/6-311G(d,p)

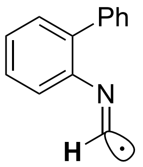

Center Number	Atomic Number	Atomic Type	Coordinates (Angstroms) X	Y	Z
1	6	0	0.554538	-0.447740	0.075098
2	6	0	1.111052	-1.692977	0.373354
3	6	0	2.483671	-1.893977	0.379181
4	6	0	3.336603	-0.838178	0.083725
5	6	0	2.809695	0.410407	-0.206177
6	6	0	1.429569	0.612025	-0.204019
7	1	0	0.443588	-2.512931	0.614917
8	1	0	2.886457	-2.873029	0.610651
9	1	0	4.410361	-0.985604	0.074713
10	1	0	3.462205	1.236818	-0.464702
11	7	0	0.905275	1.875202	-0.563241
12	1	0	-1.224828	-1.963378	-1.248243
13	6	0	-0.920947	-0.283556	0.053840
14	6	0	-1.553244	0.734811	0.768445
15	6	0	-2.935453	0.857414	0.750487
16	6	0	-3.707771	-0.033598	0.015276
17	6	0	-3.089018	-1.052676	-0.697837
18	6	0	-1.706452	-1.177218	-0.676617
19	1	0	-0.958887	1.428142	1.350998
20	1	0	-3.411028	1.653172	1.312566
21	1	0	-4.786988	0.067502	-0.003026
22	1	0	-3.683468	-1.747956	-1.279768
23	6	0	1.266000	3.004968	-0.283894
24	1	0	2.056195	3.372621	0.389404

```
Temperature   298.150 Kelvin.  Pressure   1.00000 Atm
 Zero-point correction=                           0.190119 (Hartree/Particle)
 Thermal correction to Energy=                    0.201256
 Thermal correction to Enthalpy=                  0.202200
 Thermal correction to Gibbs Free Energy=         0.151830
 Sum of electronic and zero-point Energies=           -555.805559
 Sum of electronic and thermal Energies=              -555.794422
 Sum of electronic and thermal Enthalpies=            -555.793477
 Sum of electronic and thermal Free Energies=         -555.843847
```

H-imidoyl: trans to cis TS, UwB97XD/6-311G(d,p)

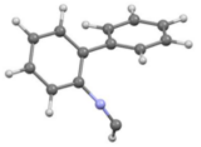

Center Number	Atomic Number	Atomic Type	Coordinates (Angstroms) X	Y	Z
1	6	0	-0.634448	-0.415918	-0.077834
2	6	0	-1.260822	-1.653497	-0.206700
3	6	0	-2.643421	-1.781848	-0.222750
4	6	0	-3.437973	-0.645239	-0.108041
5	6	0	-2.855556	0.600958	0.025837
6	6	0	-1.456700	0.727788	0.052008
7	1	0	-0.637183	-2.534452	-0.313862
8	1	0	-3.096935	-2.759069	-0.335097
9	1	0	-4.518492	-0.730176	-0.123145
10	1	0	-3.460104	1.494807	0.119074
11	7	0	-0.913369	1.964626	0.179504
12	1	0	1.065918	-1.824046	1.460263
13	6	0	0.847095	-0.328624	-0.063926
14	6	0	1.532070	0.543712	-0.913349
15	6	0	2.918951	0.588337	-0.910977
16	6	0	3.645941	-0.235922	-0.059914
17	6	0	2.975441	-1.107545	0.788698
18	6	0	1.587486	-1.152871	0.786214
19	1	0	0.973453	1.185007	-1.585056
20	1	0	3.434641	1.267447	-1.580478
21	1	0	4.729359	-0.197610	-0.058108
22	1	0	3.534106	-1.751105	1.458967
23	6	0	-0.445268	2.999057	0.586716
24	1	0	-0.267949	3.266486	1.645020

Imaginary = -180.7869 cm^{-1}

Temperature 298.150 Kelvin. Pressure 1.00000 Atm
Zero-point correction= 0.189034 (Hartree/Particle)
Thermal correction to Energy= 0.199716
Thermal correction to Enthalpy= 0.200660
Thermal correction to Gibbs Free Energy= 0.151328
Sum of electronic and zero-point Energies= -555.804377
Sum of electronic and thermal Energies= -555.793695
Sum of electronic and thermal Enthalpies= -555.792751
Sum of electronic and thermal Free Energies= -555.842083

Voltammograms of electrochemical measurements

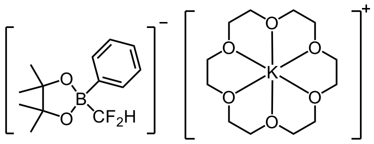

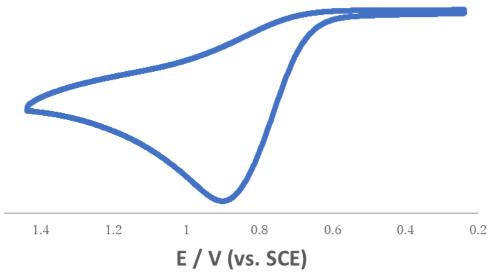

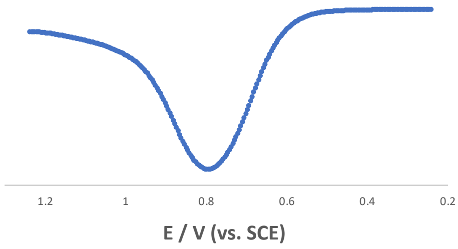

Left: Cyclic Voltammogram (CV) of **1A**. Working electrode: GC, counter electrode: Pt, reference electrode: Ag/Ag^{+} (Fc/Fc^{+} = + 0.072 V). Conditions: 1 mM in acetonitrile, supporting electrolyte: 0.1 M TBAP, scan rate: 50 mV s^{-1}, 298 K.

Right: Differential Pulse Voltammogram (DPV) of **1A**.

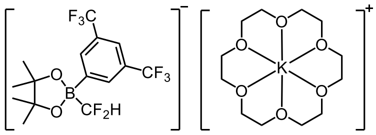

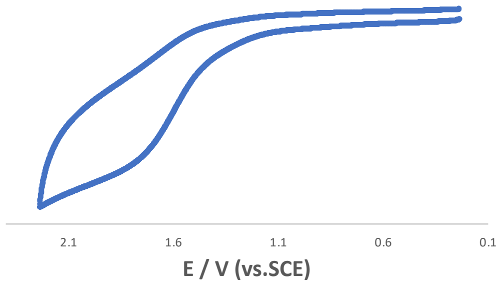

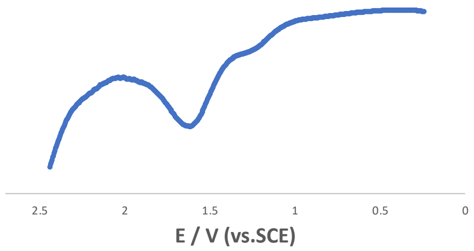

Left: Cyclic Voltammogram (CV) of **1B**. Working electrode: GC, counter electrode: Pt, reference electrode: Ag/Ag^{+} (Fc/Fc^{+} = + 0.072 V). Conditions: 1 mM in acetonitrile, supporting electrolyte: 0.1 M TBAP, scan rate: 50 mV s^{-1}, 298 K.

Right: Differential Pulse Voltammogram (DPV) of **1B**.

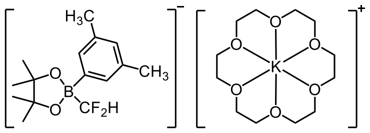

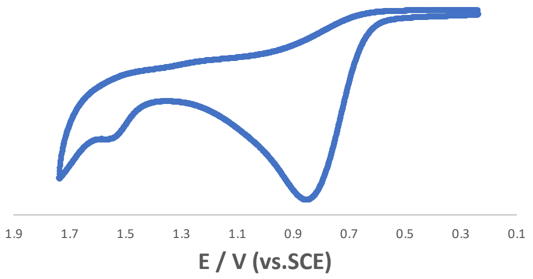

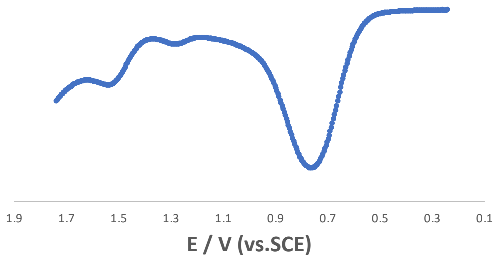

Left: Cyclic Voltammogram (CV) of **1C**. Working electrode: GC, counter electrode: Pt, reference electrode: Ag/Ag$^+$ (Fc/Fc$^+$ = + 0.072 V). Conditions: 1 mM in acetonitrile, supporting electrolyte: 0.1 M TBAP, scan rate: 50 mV s^{-1}, 298 K.

Right: Differential Pulse Voltammogram (DPV) of **1C**.

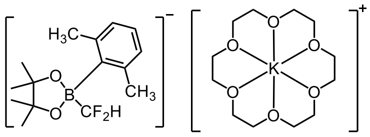

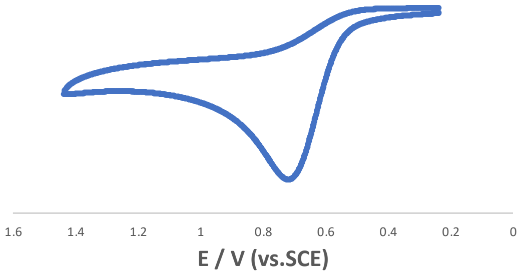

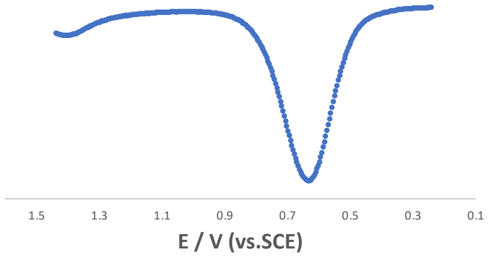

Left: Cyclic Voltammogram (CV) of **1D**. Working electrode: GC, counter electrode: Pt, reference electrode: Ag/Ag$^+$ (Fc/Fc$^+$ = + 0.072 V). Conditions: 1 mM in acetonitrile, supporting electrolyte: 0.1 M TBAP, scan rate: 50 mV s^{-1}, 298 K.

Right: Differential Pulse Voltammogram (DPV) of **1D**.

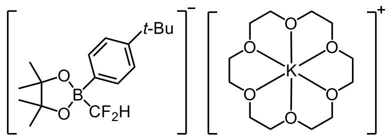

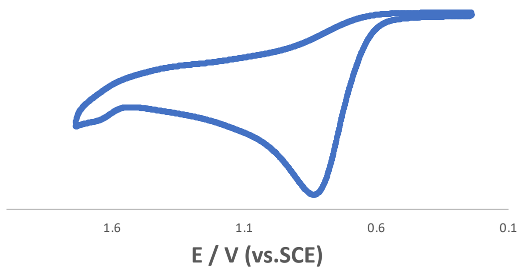

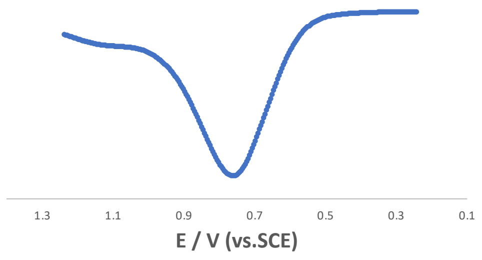

Left: Cyclic Voltammogram (CV) of **1E**. Working electrode: GC, counter electrode: Pt, reference electrode: Ag/Ag^+ (Fc/Fc^+ = + 0.072 V). Conditions: 1 mM in acetonitrile, supporting electrolyte: 0.1 M TBAP, scan rate: 50 mV s^{-1}, 298 K.

Right: Differential Pulse Voltammogram (DPV) of **1E**.

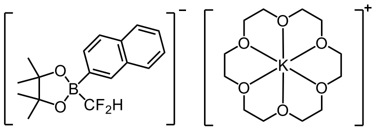

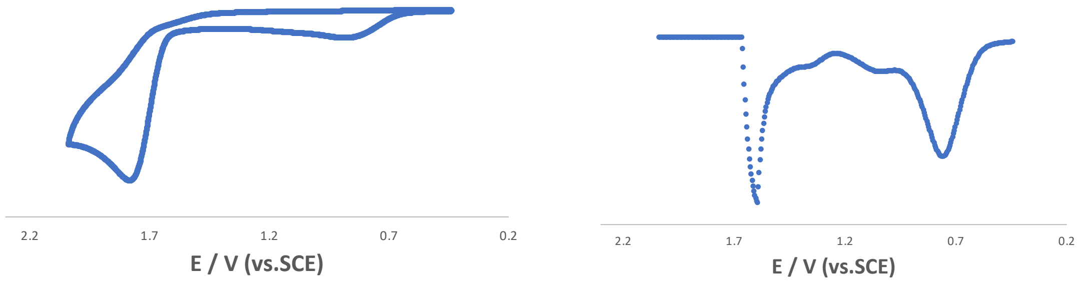

Left: Cyclic Voltammogram (CV) of **1F**. Working electrode: GC, counter electrode: Pt, reference electrode: Ag/Ag^+ (Fc/Fc^+ = + 0.072 V). Conditions: 1 mM in acetonitrile, supporting electrolyte: 0.1 M TBAP, scan rate: 50 mV s^{-1}, 298 K.

Right: Differential Pulse Voltammogram (DPV) of **1F**.

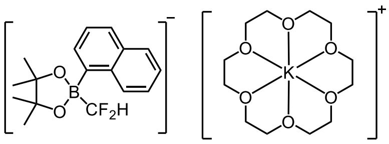

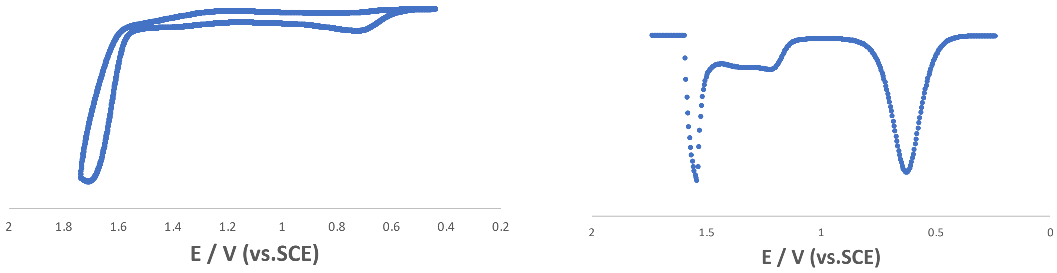

Left: Cyclic Voltammogram (CV) of **1G**. Working electrode: GC, counter electrode: Pt, reference electrode: Ag/Ag^+ (Fc/Fc^+ = + 0.072 V). Conditions: 1 mM in acetonitrile, supporting electrolyte: 0.1 M TBAP, scan rate: 50 mV s^{-1}, 298 K.

Right: Differential Pulse Voltammogram (DPV) of **1G**.

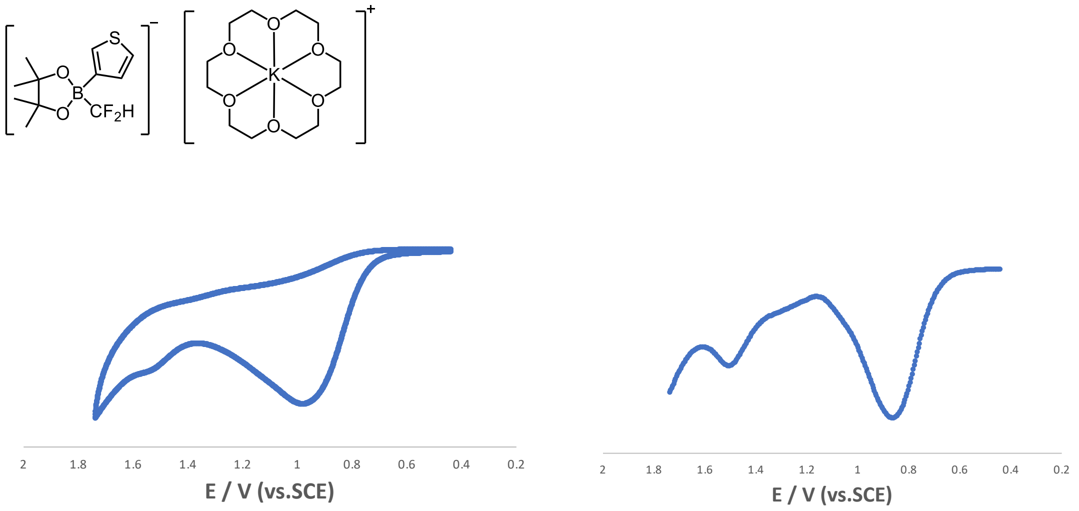

Left: Cyclic Voltammogram (CV) of **1H**. Working electrode: GC, counter electrode: Pt, reference electrode: Ag/Ag^+ (Fc/Fc^+ = + 0.072 V). Conditions: 1 mM in acetonitrile, supporting electrolyte: 0.1 M TBAP, scan rate: 50 mV s^{-1}, 298 K.

Right: Differential Pulse Voltammogram (DPV) of **1H**.

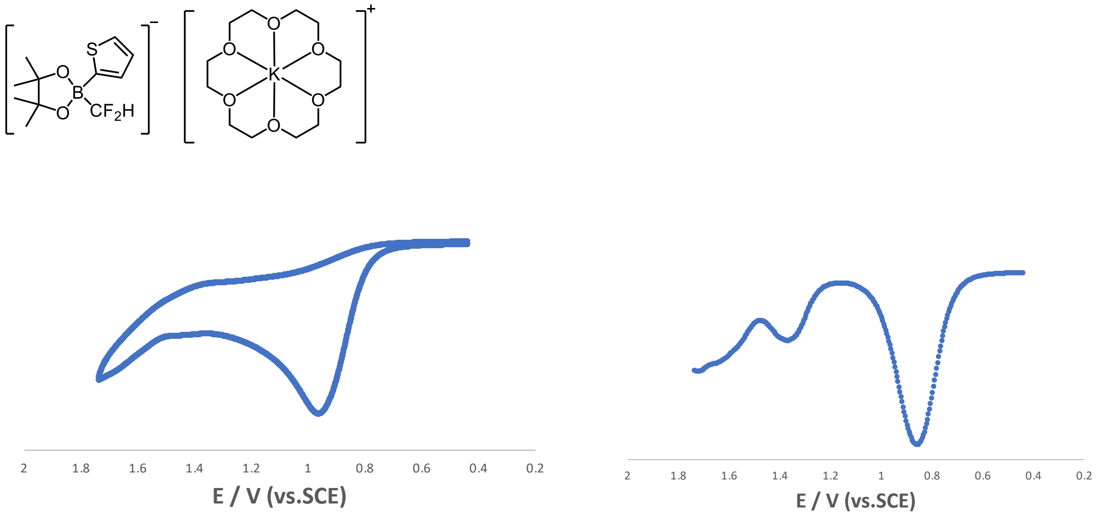

Left: Cyclic Voltammogram (CV) of **1I**. Working electrode: GC, counter electrode: Pt, reference electrode: Ag/Ag^+ (Fc/Fc^+ = + 0.072 V). Conditions: 1 mM in acetonitrile, supporting electrolyte: 0.1 M TBAP, scan rate: 50 mV s^{-1}, 298 K.

Right: Differential Pulse Voltammogram (DPV) of **1I**.

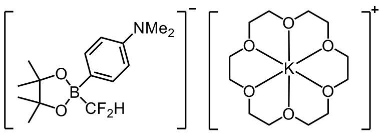

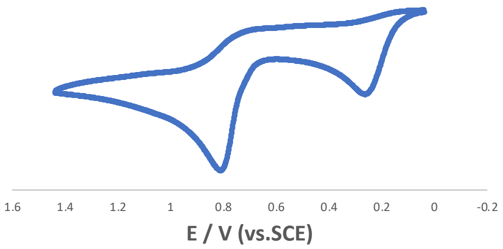

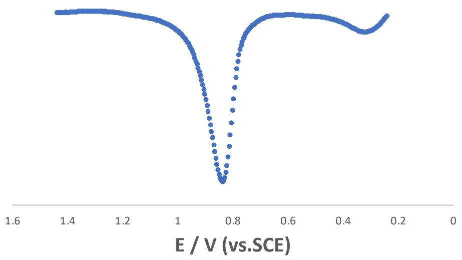

Left: Cyclic Voltammogram (CV) of **1J**. Working electrode: GC, counter electrode: Pt, reference electrode: Ag/Ag^+ (Fc/Fc^+ = + 0.072 V). Conditions: 1 mM in acetonitrile, supporting electrolyte: 0.1 M TBAP, scan rate: 50 mV s^{-1}, 298 K.

Right: Differential Pulse Voltammogram (DPV) of **1J**.

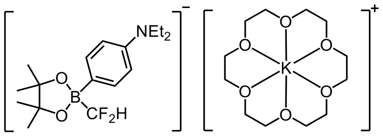

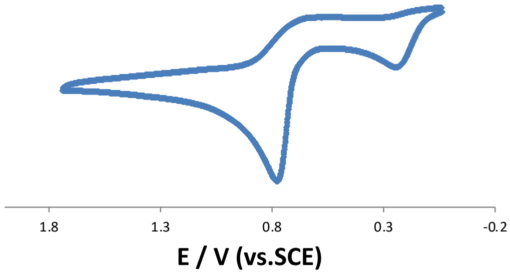

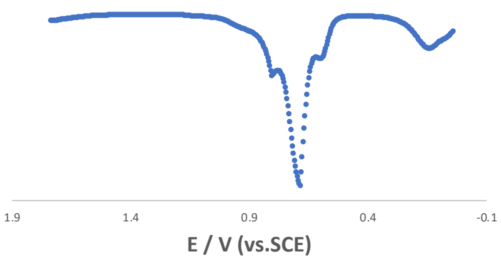

Left: Cyclic Voltammogram (CV) of **1K**. Working electrode: GC, counter electrode: Pt, reference electrode: Ag/Ag^+ (Fc/Fc^+ = + 0.072 V). Conditions: 1 mM in acetonitrile, supporting electrolyte: 0.1 M TBAP, scan rate: 50 mV s^{-1}, 298 K.

Right: Differential Pulse Voltammogram (DPV) of **1K**.

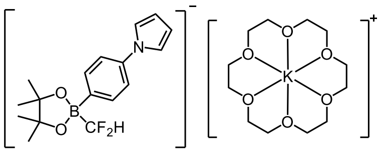

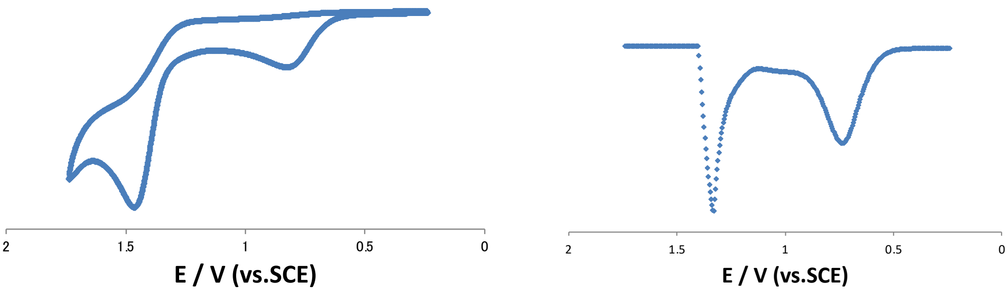

Left: Cyclic Voltammogram (CV) of **1L**. Working electrode: GC, counter electrode: Pt, reference electrode: Ag/Ag^{+} (Fc/Fc^{+} = + 0.072 V). Conditions: 1 mM in acetonitrile, supporting electrolyte: 0.1 M TBAP, scan rate: 50 mV s^{-1}, 298 K.

Right: Differential Pulse Voltammogram (DPV) of **1L**.

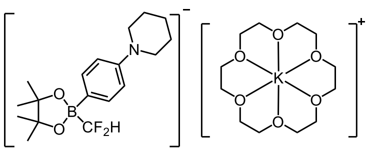

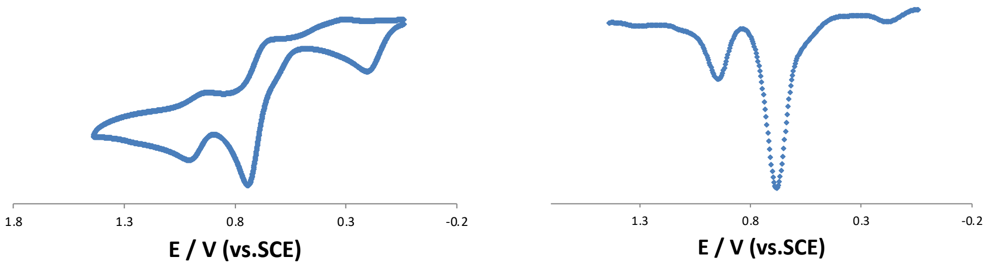

Left: Cyclic Voltammogram (CV) of **1N**. Working electrode: GC, counter electrode: Pt, reference electrode: Ag/Ag^{+} (Fc/Fc^{+} = + 0.072 V). Conditions: 1 mM in acetonitrile, supporting electrolyte: 0.1 M TBAP, scan rate: 50 mV s^{-1}, 298 K.

Right: Differential Pulse Voltammogram (DPV) of **1N**.

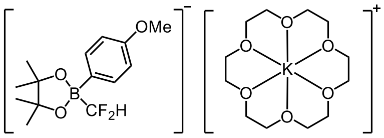

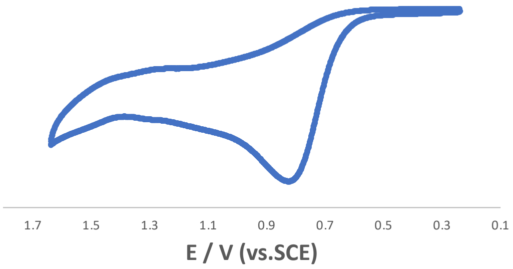

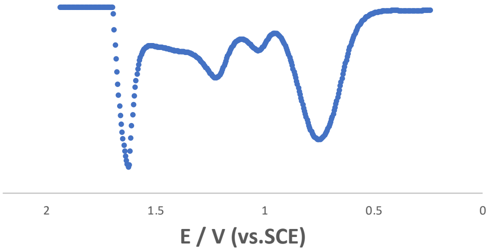

Left: Cyclic Voltammogram (CV) of **1P**. Working electrode: GC, counter electrode: Pt, reference electrode: Ag/Ag^{+} (Fc/Fc^{+} = + 0.072 V). Conditions: 1 mM in acetonitrile, supporting electrolyte: 0.1 M TBAP, scan rate: 50 mV s^{-1}, 298 K.

Right: Differential Pulse Voltammogram (DPV) of **1P**.

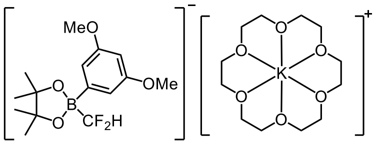

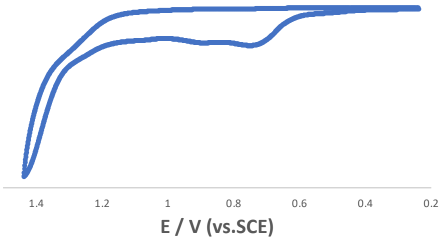

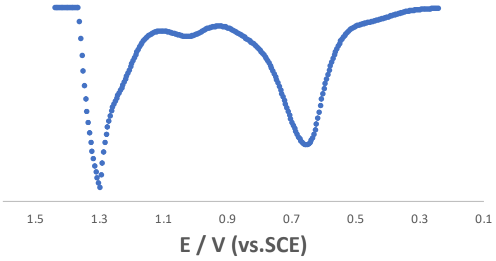

Left: Cyclic Voltammogram (CV) of **1Q**. Working electrode: GC, counter electrode: Pt, reference electrode: Ag/Ag^{+} (Fc/Fc^{+} = + 0.072 V). Conditions: 1 mM in acetonitrile, supporting electrolyte: 0.1 M TBAP, scan rate: 50 mV s^{-1}, 298 K.

Right: Differential Pulse Voltammogram (DPV) of **1Q**.

Copies of NMR spectra

4-Bromo-*N*,*N*-diethylaniline

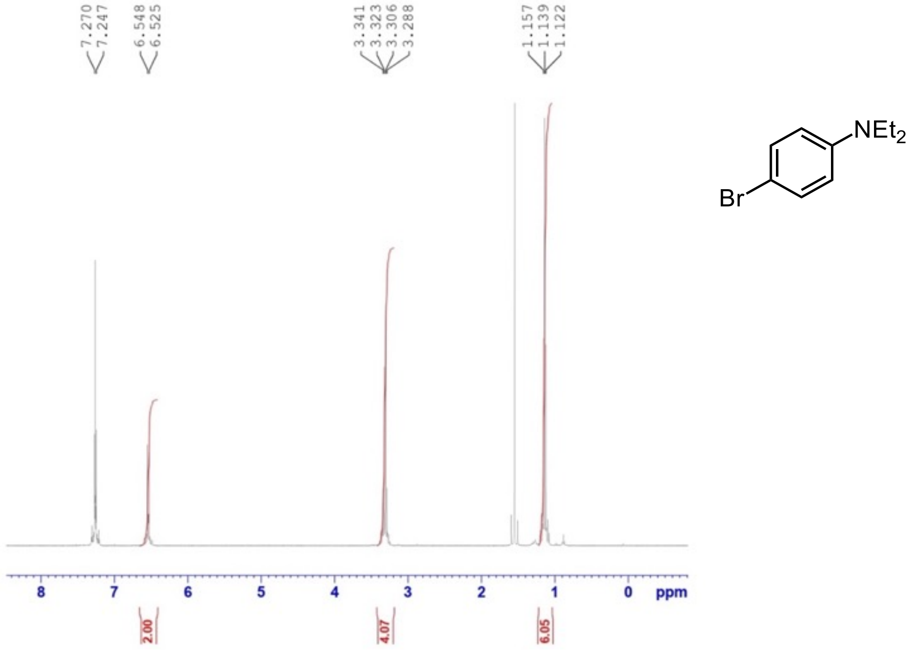

^{1}H NMR (400 MHz, $CDCl_3$)

N,*N*-Diethyl-4-(4,4,5,5-tetramethyl-1,3,2-dioxaborolan-2-yl)aniline (1K')

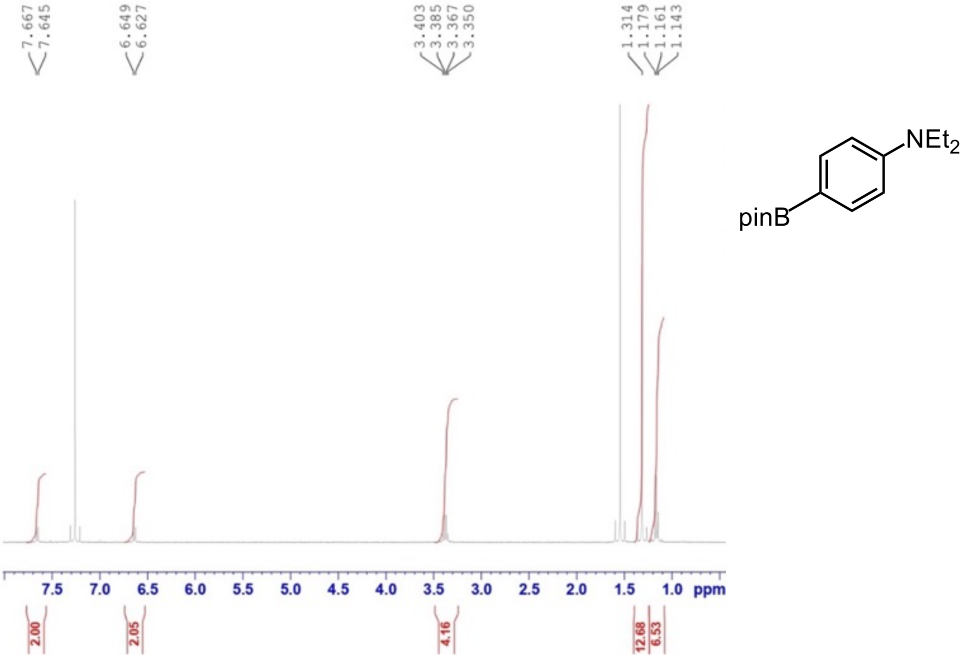

^{1}H NMR (400 MHz, $CDCl_3$)

Difluoromethy trimethylsilane

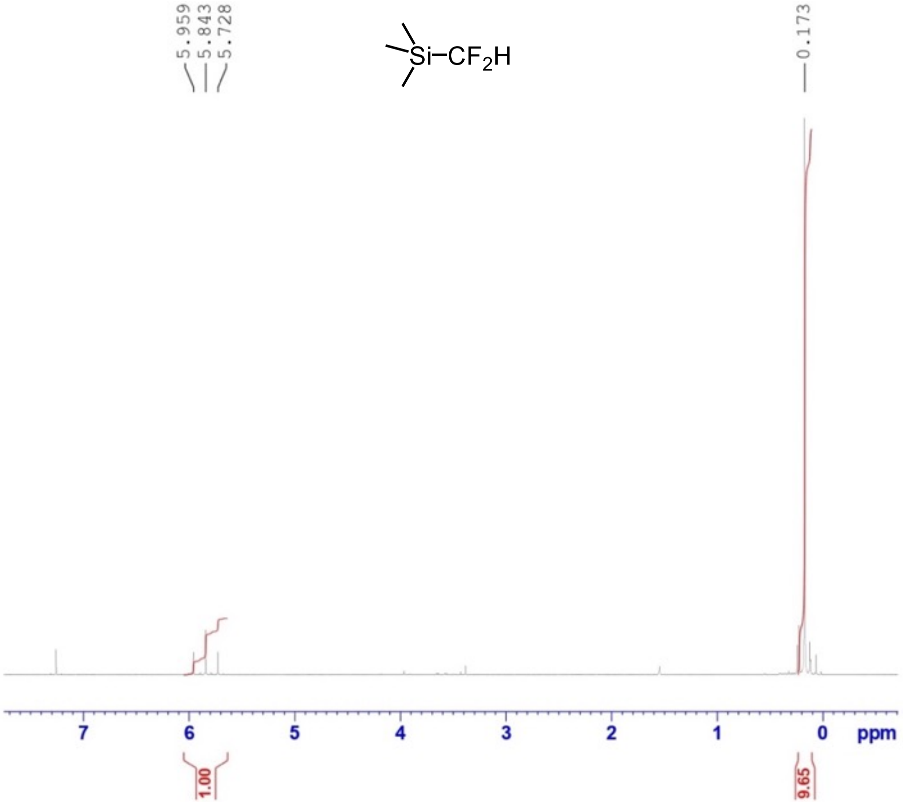

^{1}H NMR (400 MHz, $CDCl_3$)

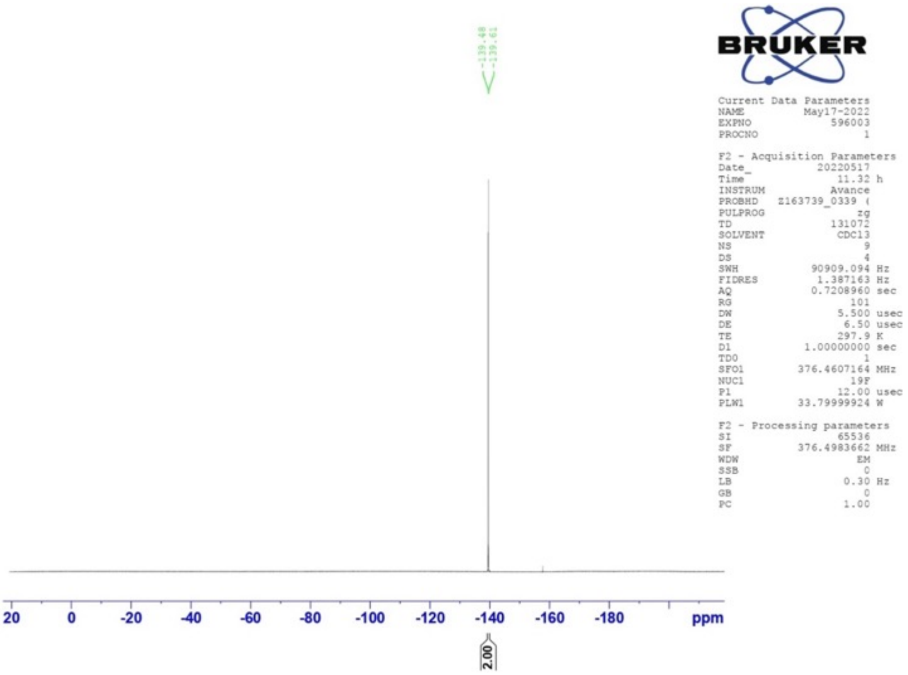

^{19}F NMR (376 MHz, $CDCl_3$)

2-(Difluoromethyl)-4,4,5,5-tetramethyl-2-phenyl-1,3,2-dioxaborolan-2-uide 18-crown-6-ether complex (1A)

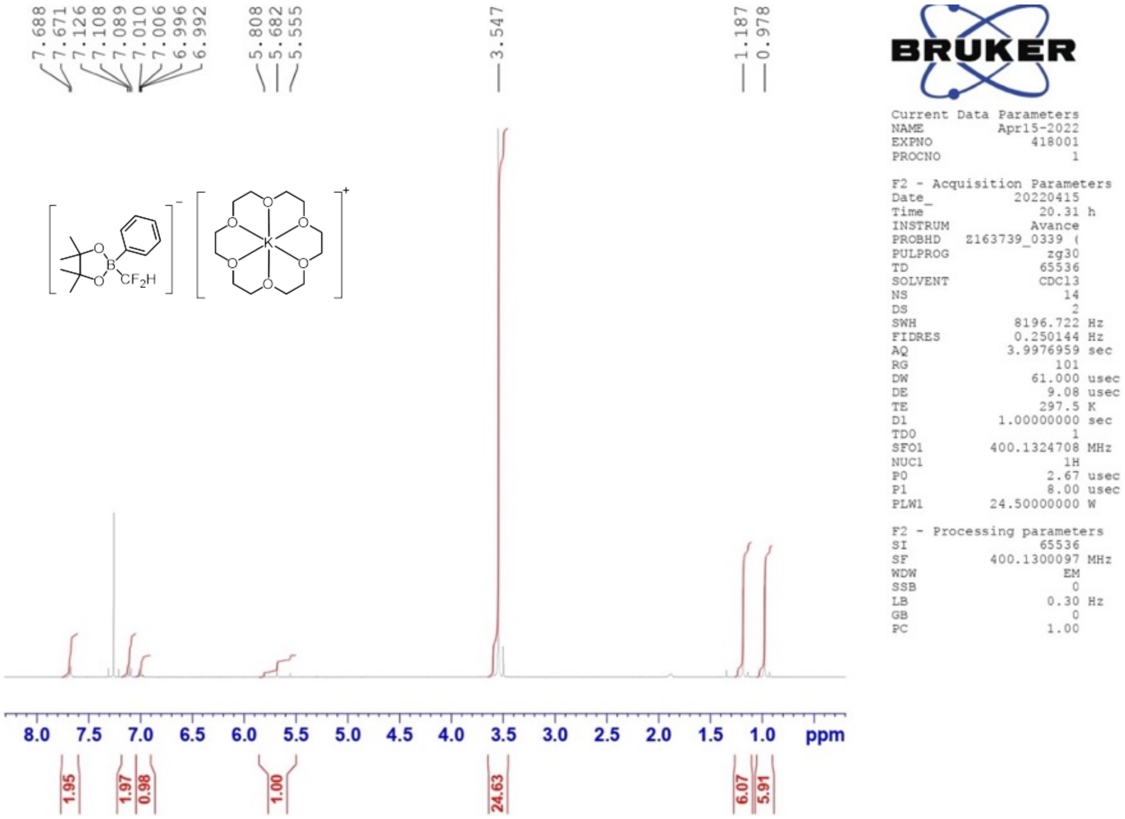

^{1}H NMR (400 MHz, $CDCl_3$)

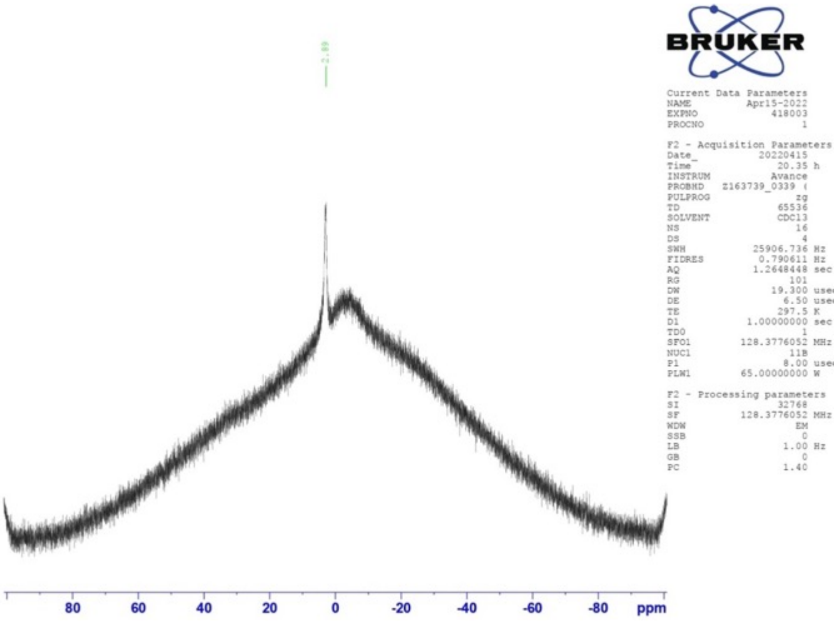

^{11}B NMR (128 MHz, $CDCl_3$)

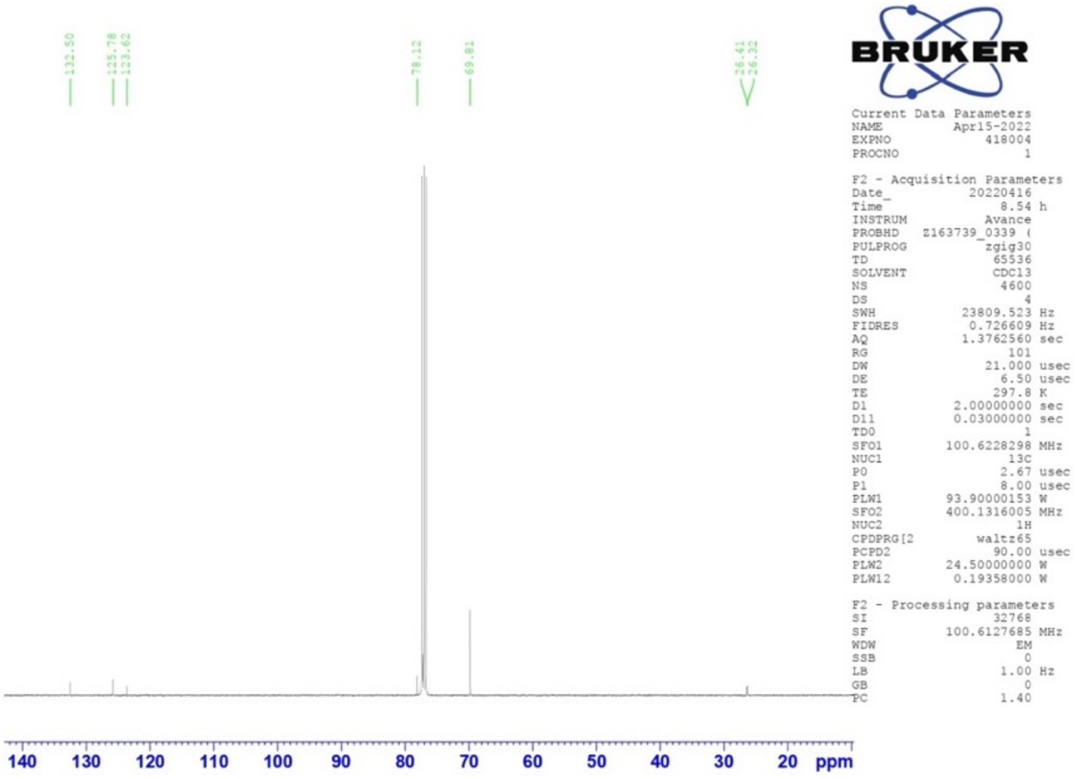

$^{13}C\{^{1}H\}$ NMR (101 MHz, $CDCl_3$)

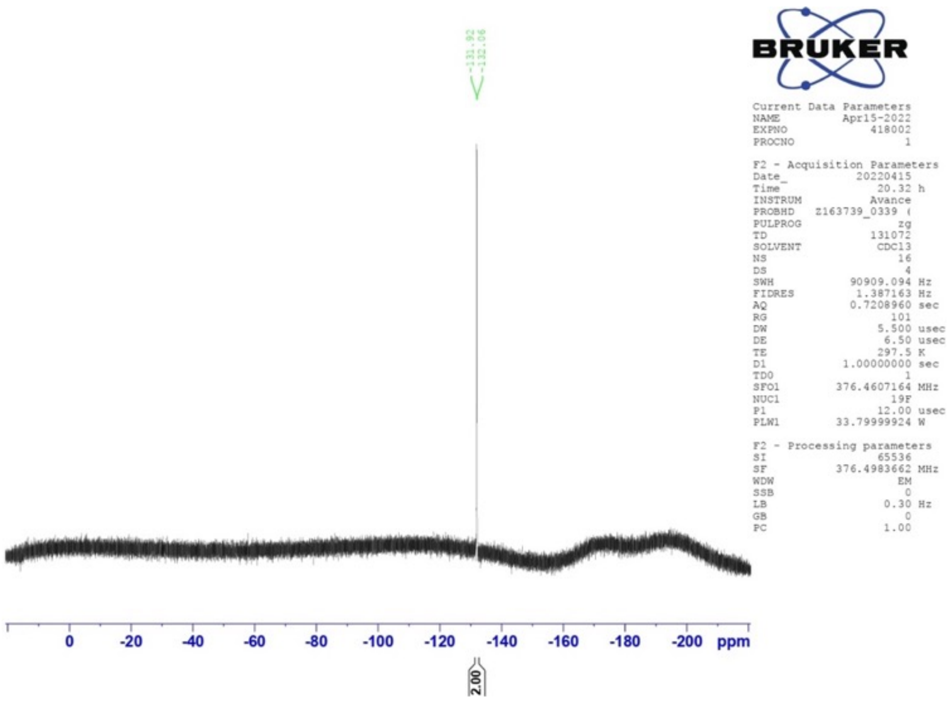

^{19}F NMR (376 MHz, $CDCl_3$)

2-(3,5-Bis(trifluoromethyl)phenyl)-2-(difluoromethyl)-4,4,5,5-tetramethyl-1,3,2-dioxaborolan-2-uide 18-crown-6-ether complex (1B)

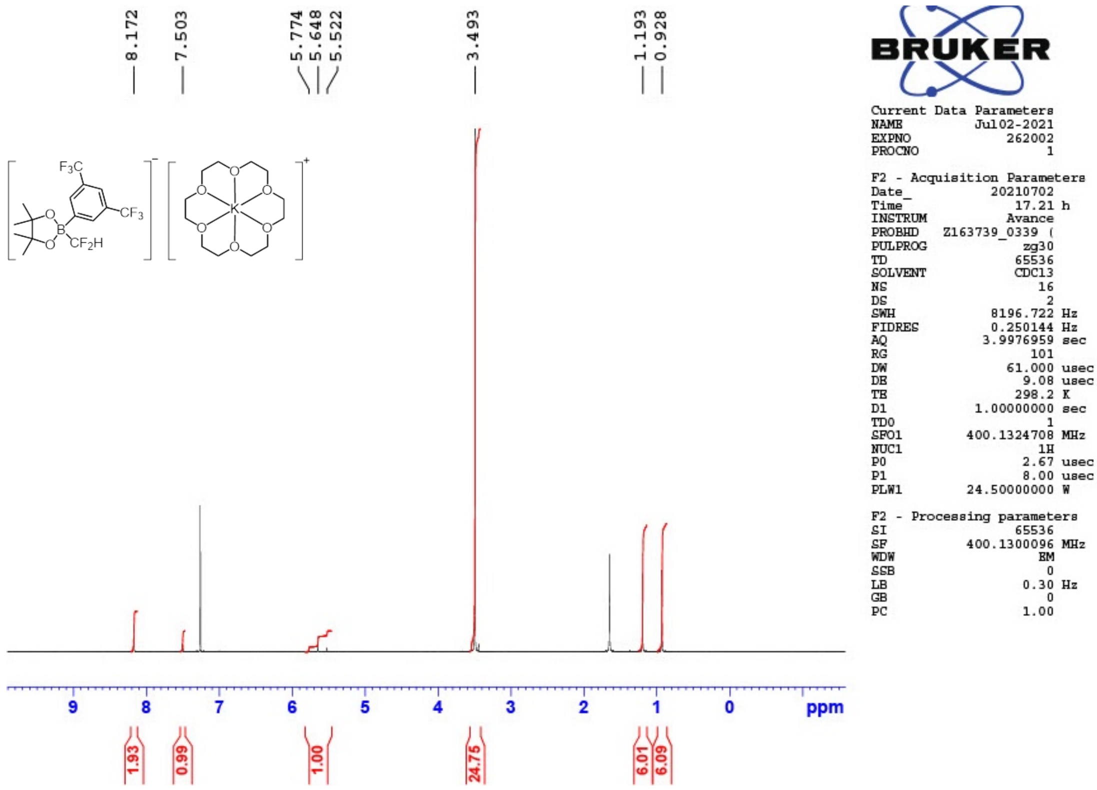

^{1}H NMR (400 MHz, $CDCl_3$)

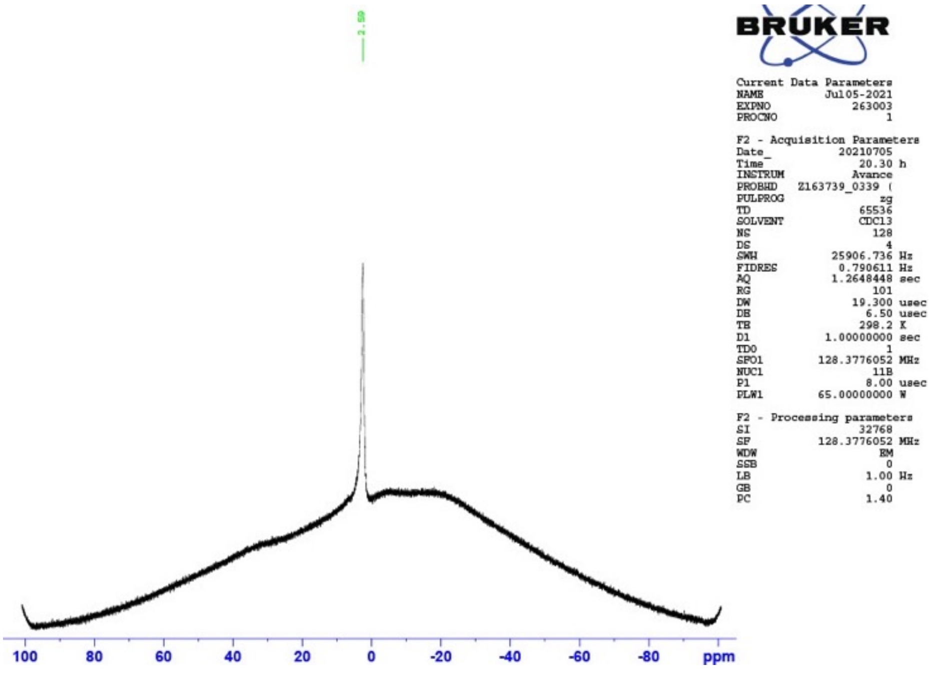

^{11}B NMR (128 MHz, $CDCl_3$)

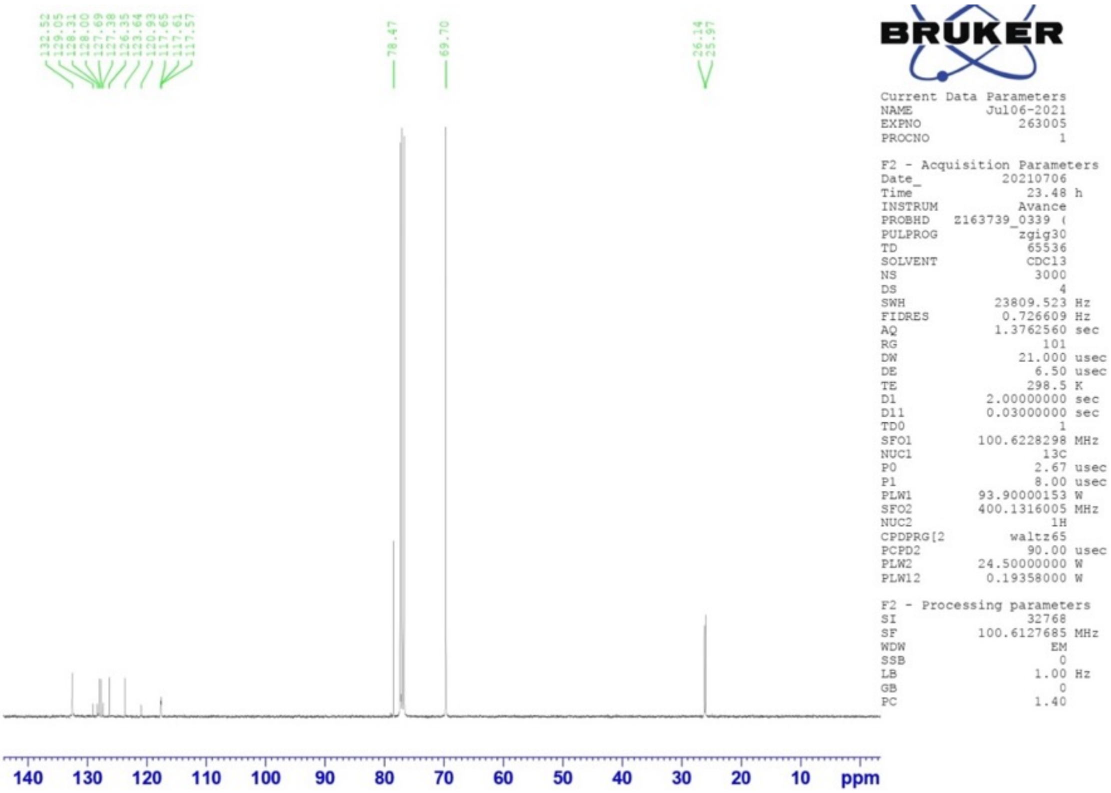

$^{13}C\{^1H\}$ NMR (101 MHz, $CDCl_3$)

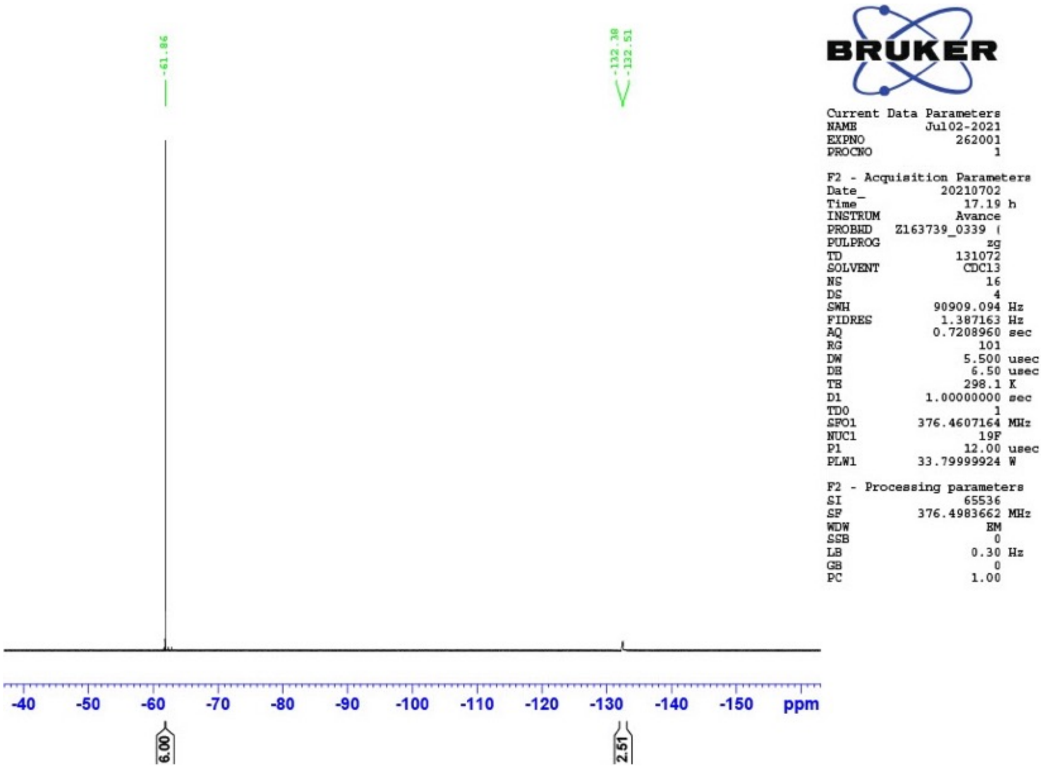

^{19}F NMR (376 MHz, $CDCl_3$)

2-(Difluoromethyl)-2-(3,5-dimethylphenyl)-4,4,5,5-tetramethyl-1,3,2-dioxaborolan-2-uide 18-crown-6-ether complex (1C)

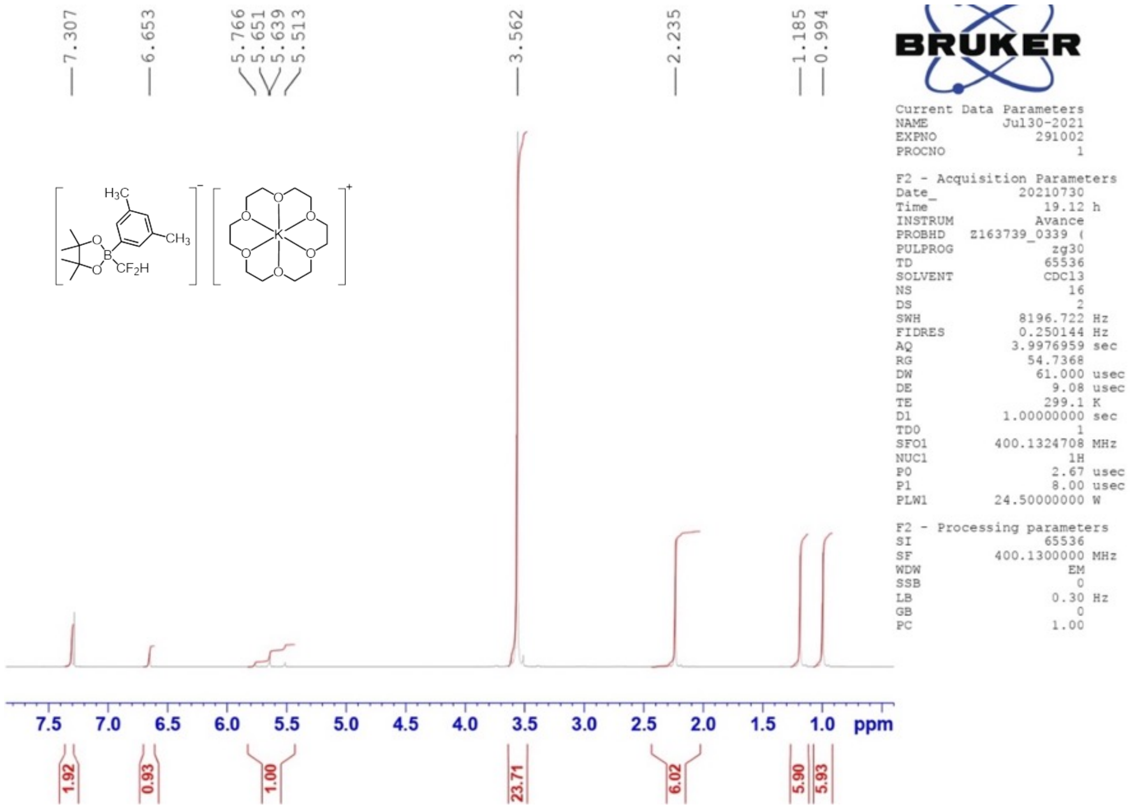

^{1}H NMR (400 MHz, $CDCl_3$)

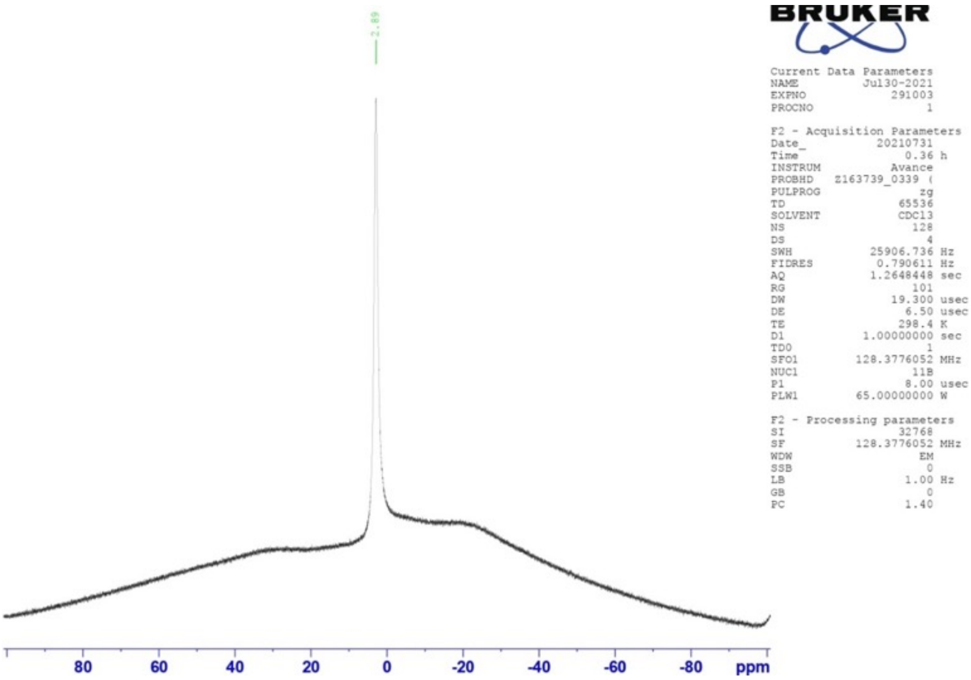

^{11}B NMR (128 MHz, $CDCl_3$)

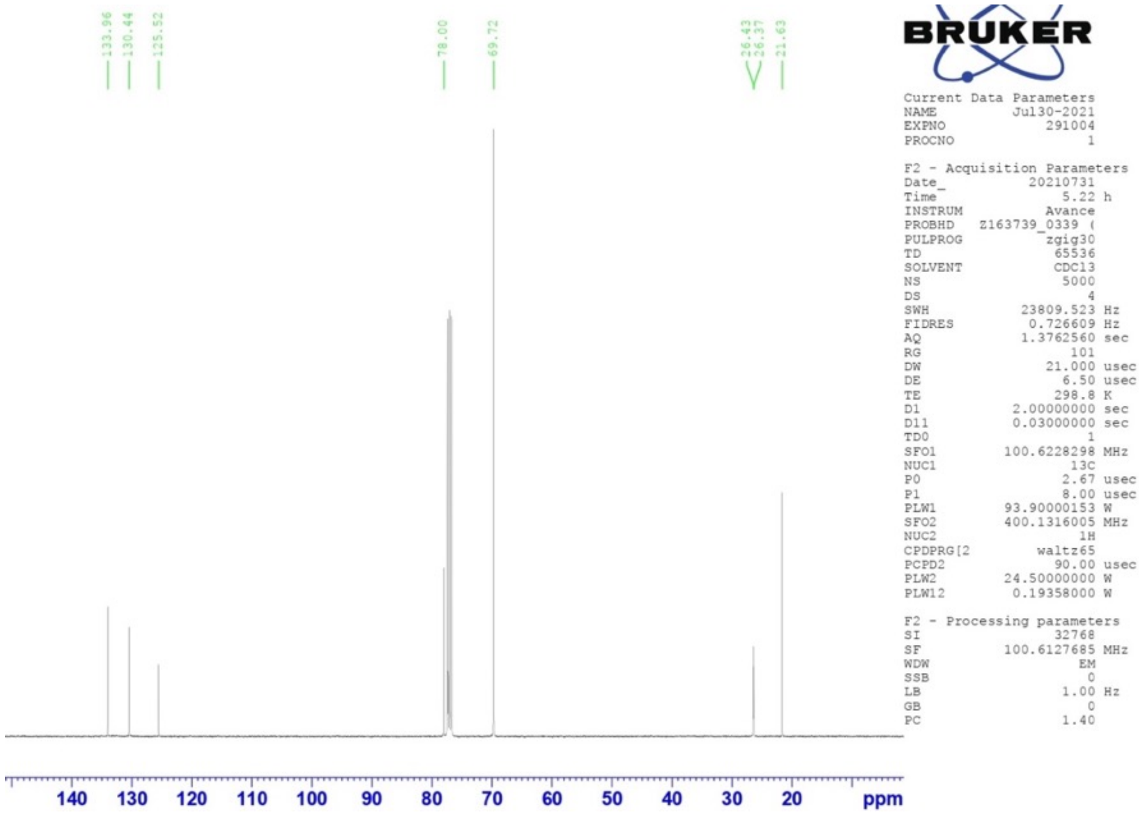

$^{13}C\{^{1}H\}$ NMR (101 MHz, $CDCl_3$)

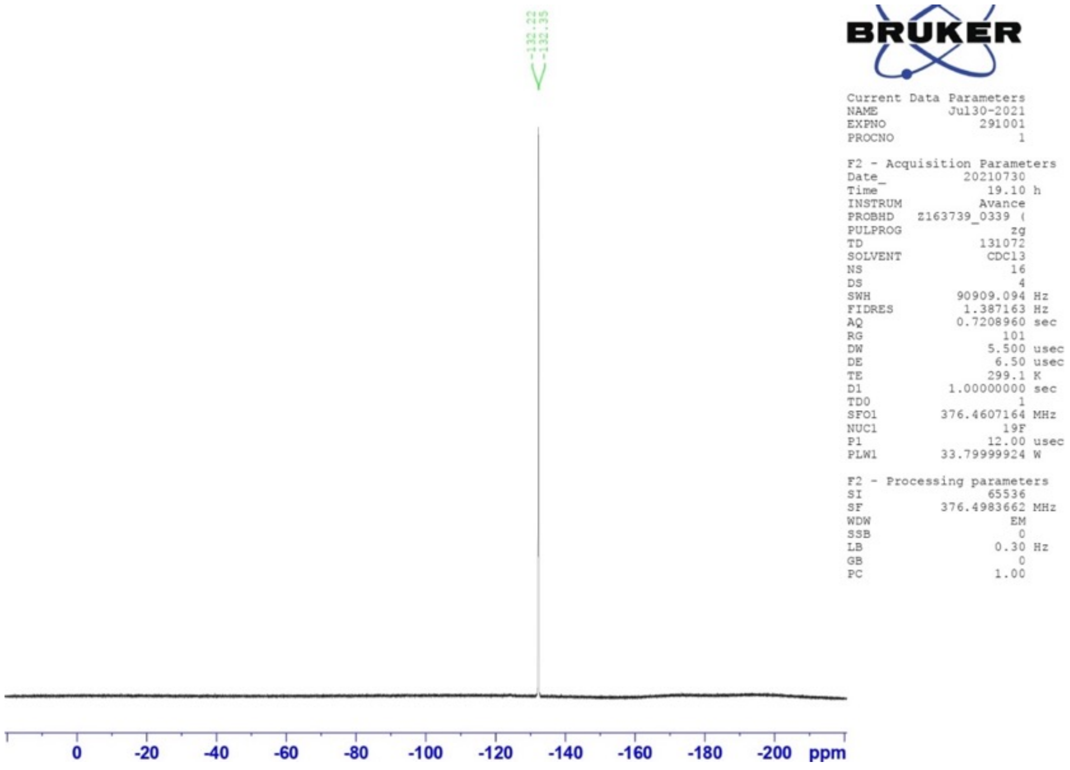

^{19}F NMR (376 MHz, $CDCl_3$)

2-(Difluoromethyl)-2-(2,6-dimethylphenyl)-4,4,5,5-tetramethyl-1,3,2-dioxaborolan-2-uide 18-crown-6-ether complex (1D)

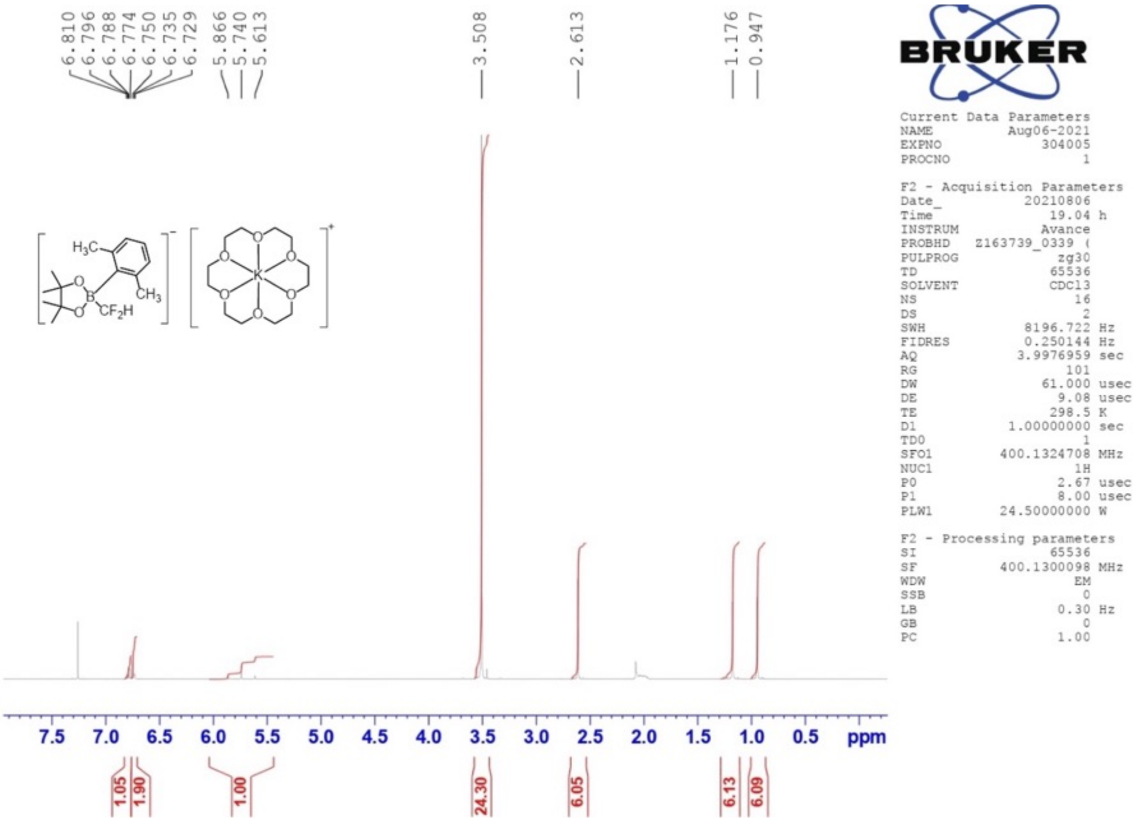

^1H NMR (400 MHz, $CDCl_3$)

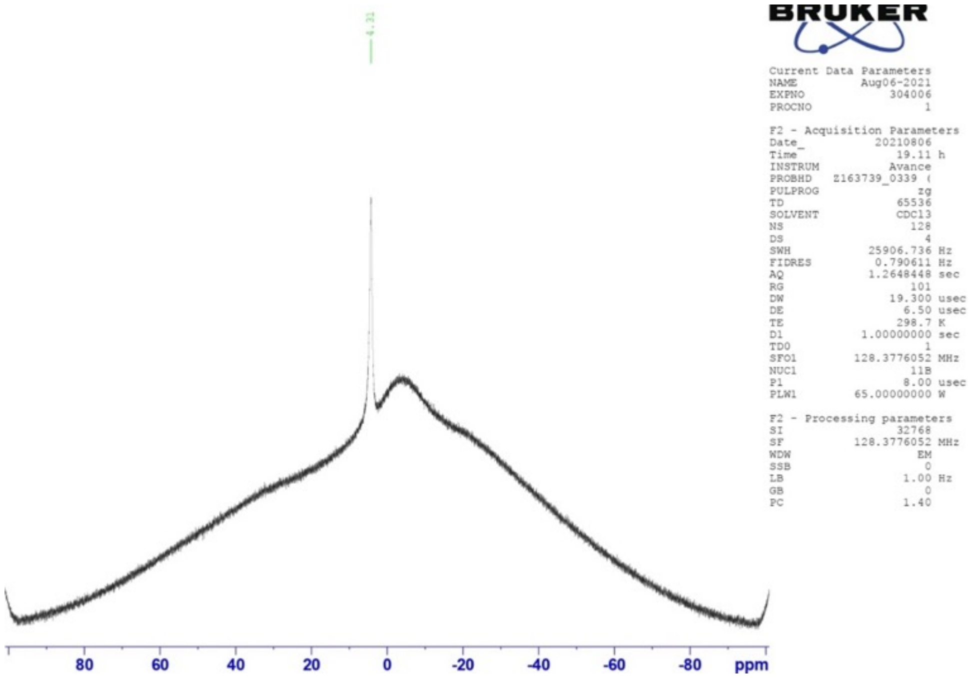

^{11}B NMR (128 MHz, $CDCl_3$)

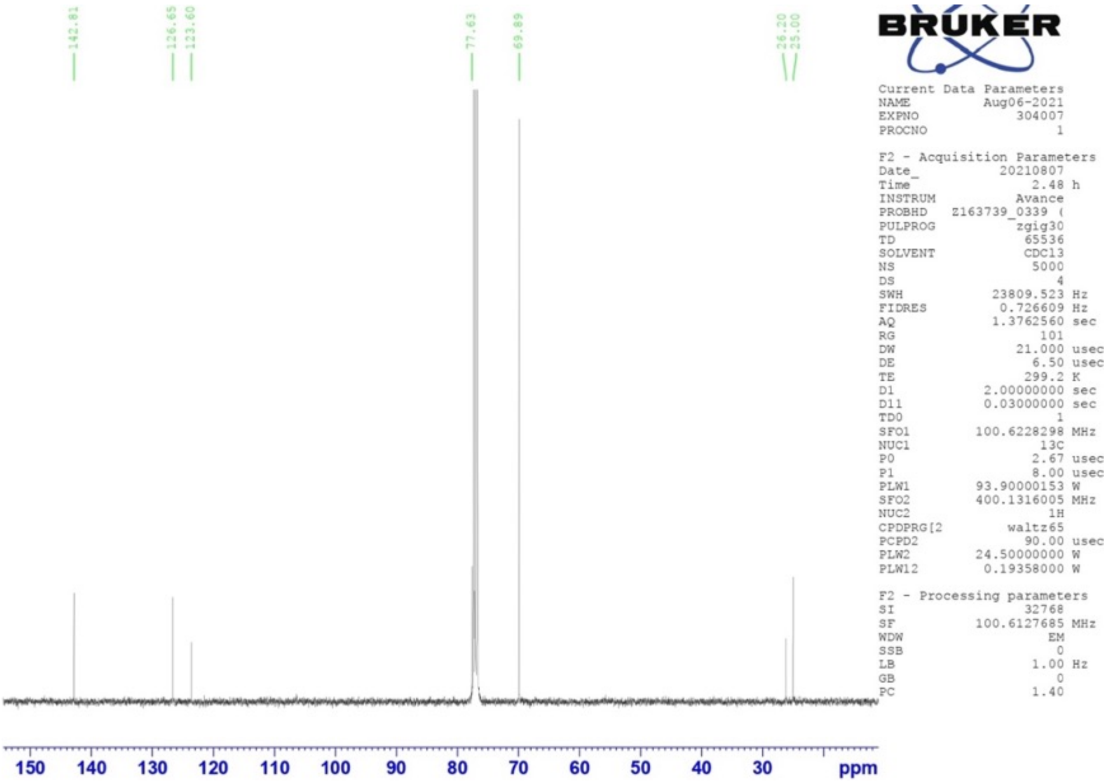

$^{13}C\{^{1}H\}$ NMR (101 MHz, $CDCl_3$)

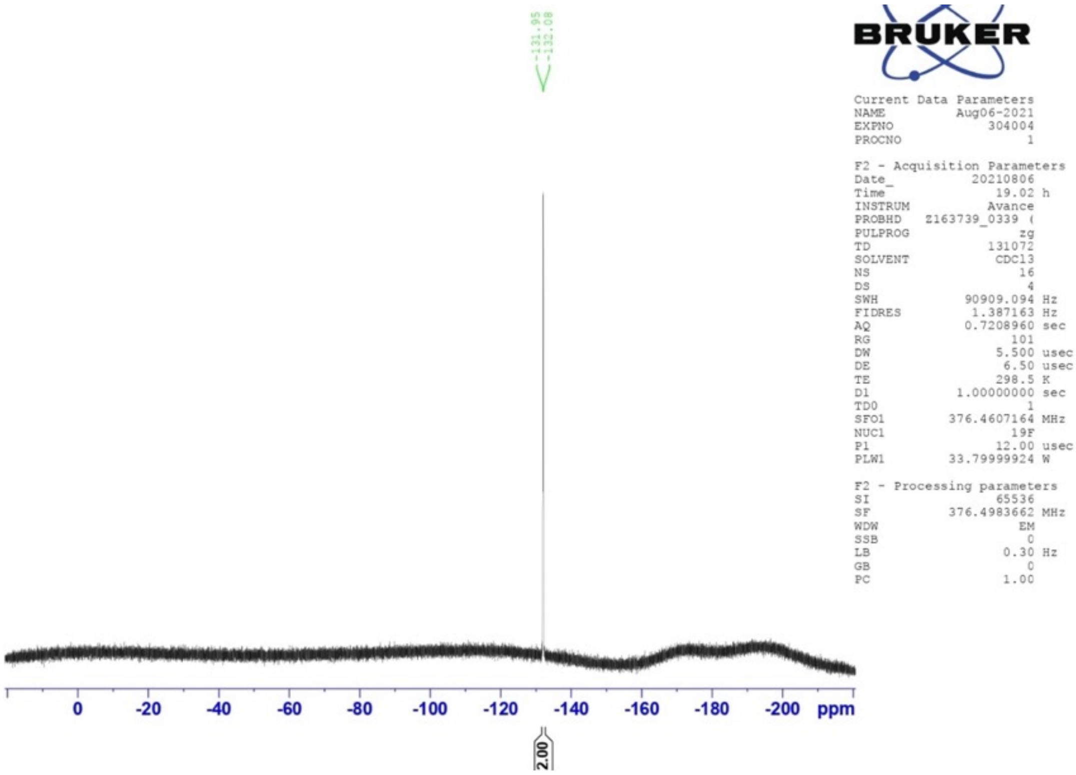

^{19}F NMR (376 MHz, $CDCl_3$)

2-(4-(*tert*-Butyl)phenyl)-2-(difluoromethyl)-4,4,5,5-tetramethyl-1,3,2-dioxaborolan-2-uide 18-crown-6-ether complex (1E)

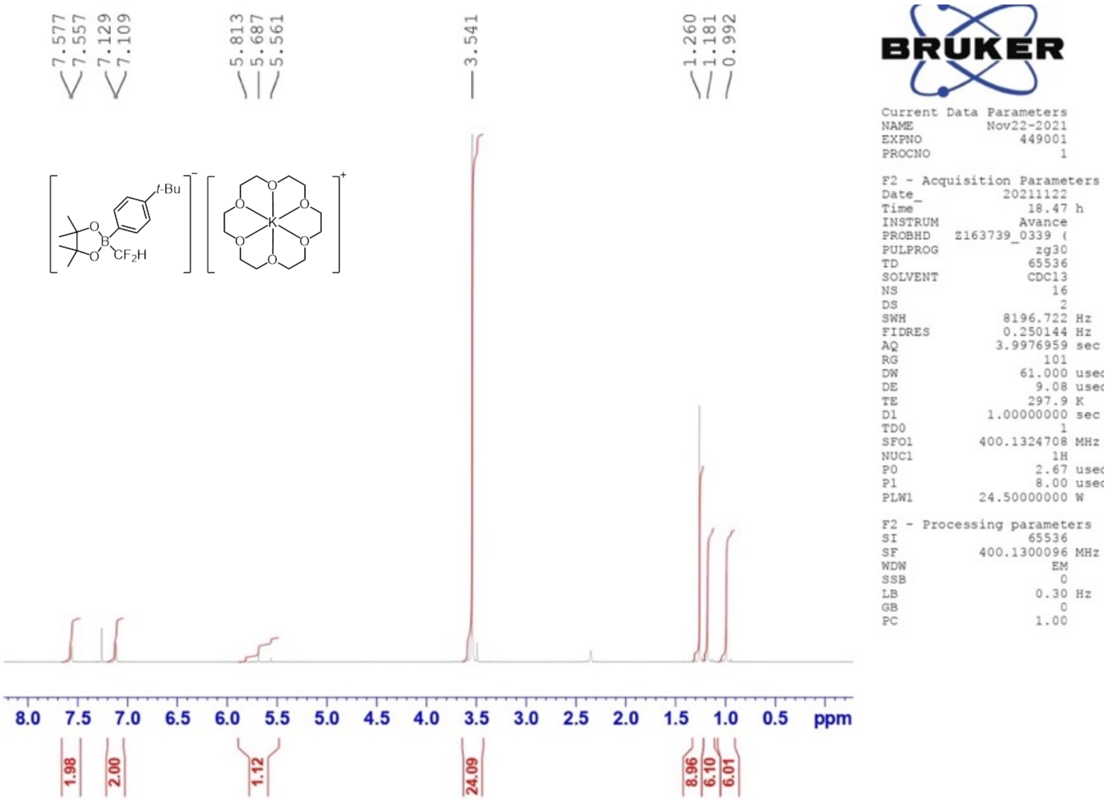

^{1}H NMR (400 MHz, $CDCl_3$)

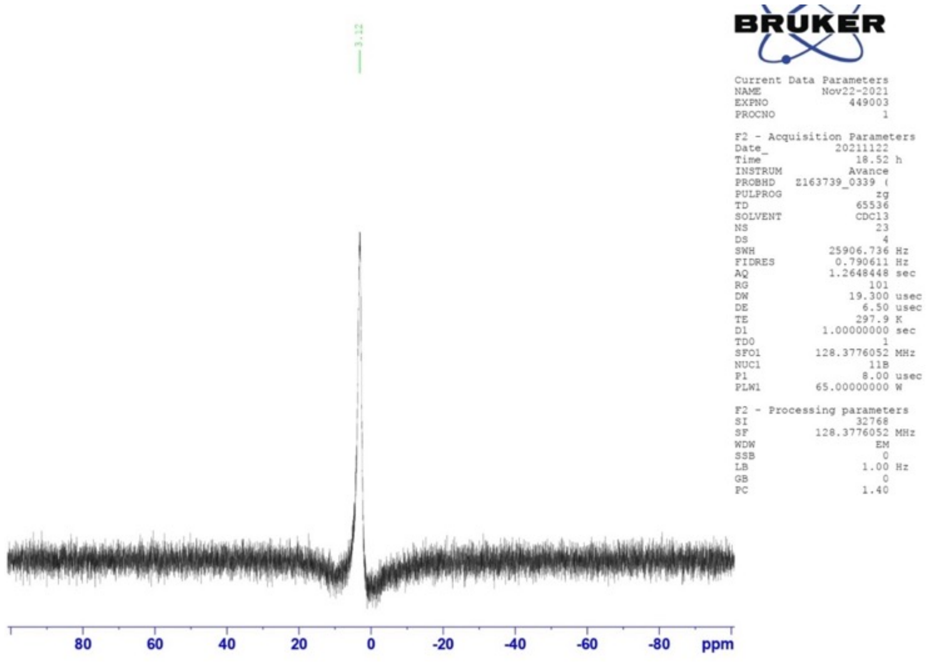

^{11}B NMR (128 MHz, $CDCl_3$)

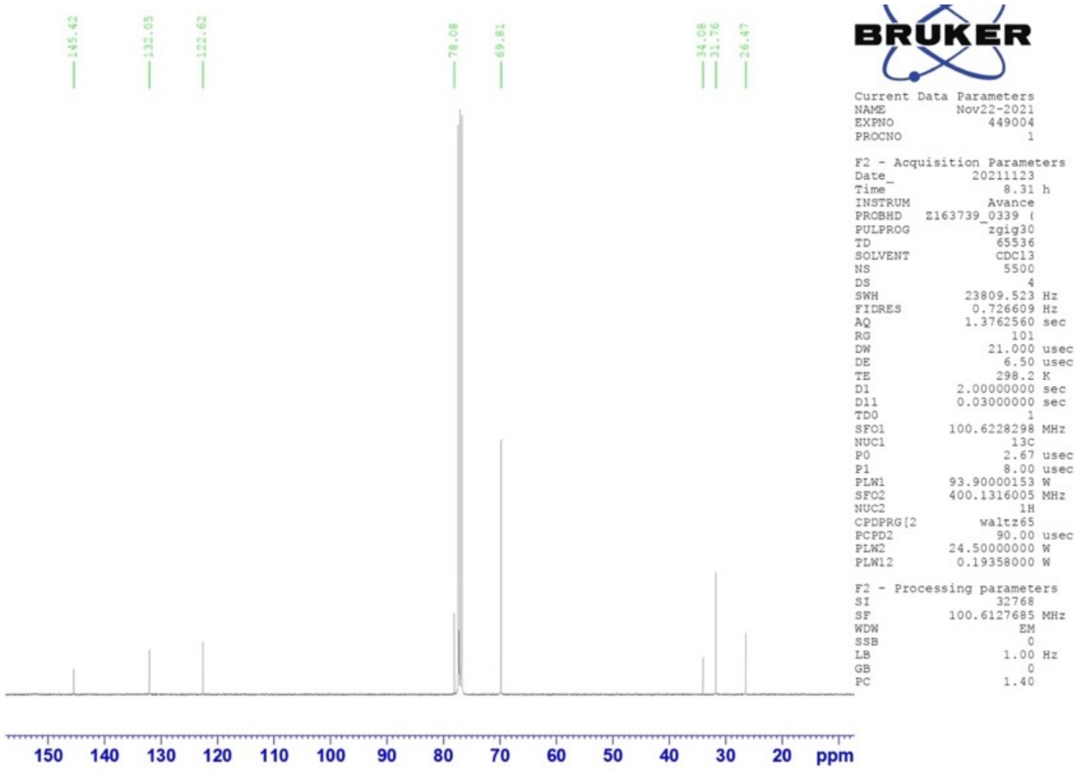

$^{13}C\{^{1}H\}$ NMR (101 MHz, $CDCl_3$)

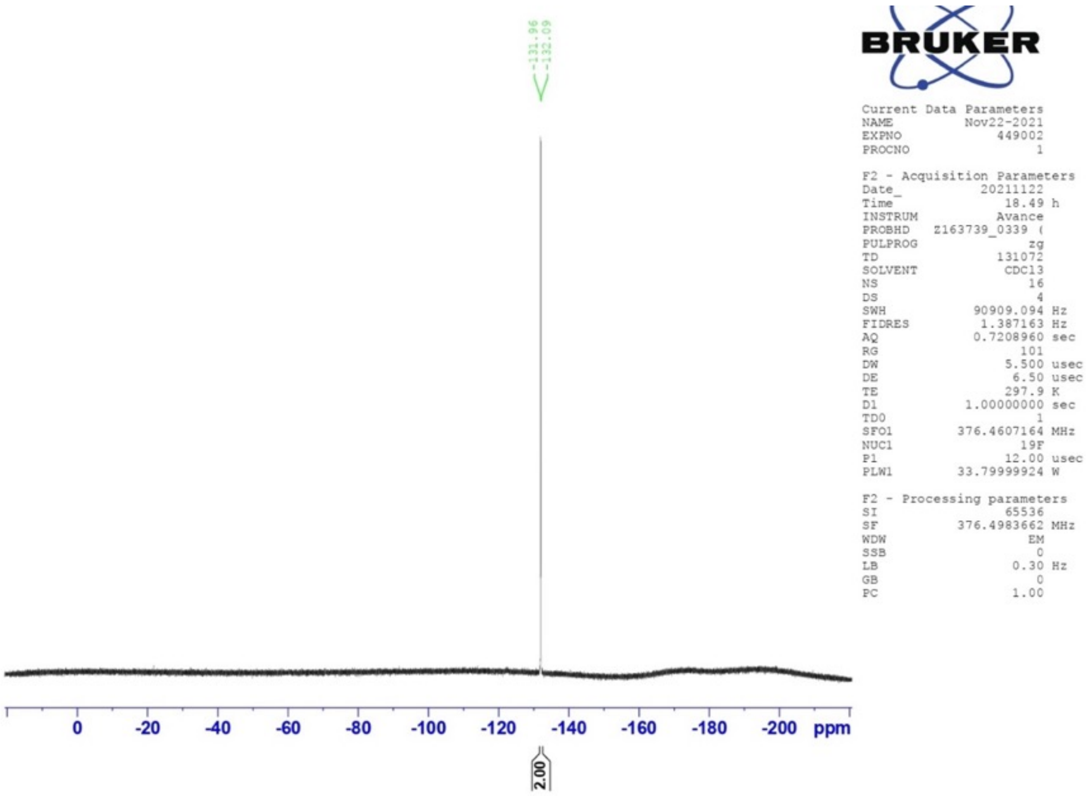

^{19}F NMR (376 MHz, $CDCl_3$)

2-(Difluoromethyl)-4,4,5,5-tetramethyl-2-(naphthalen-2-yl)-1,3,2-dioxaborolan-2-uide 18-crown-6-ether complex (1F)

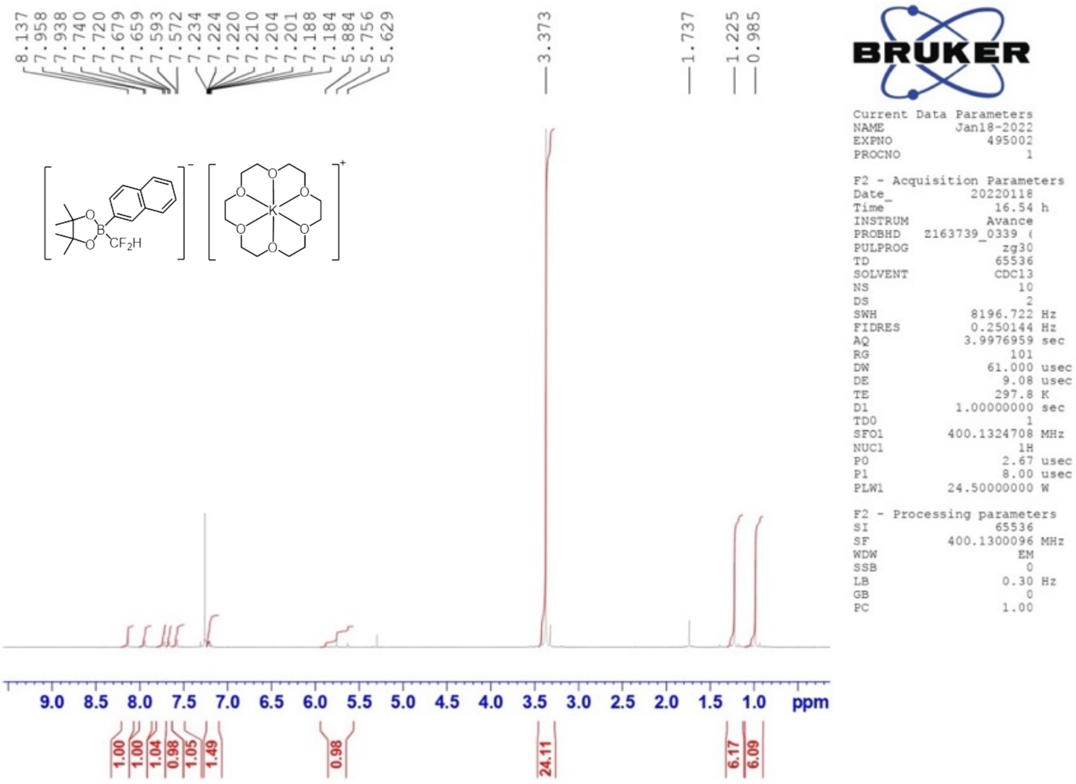

^{1}H NMR (400 MHz, $CDCl_3$)

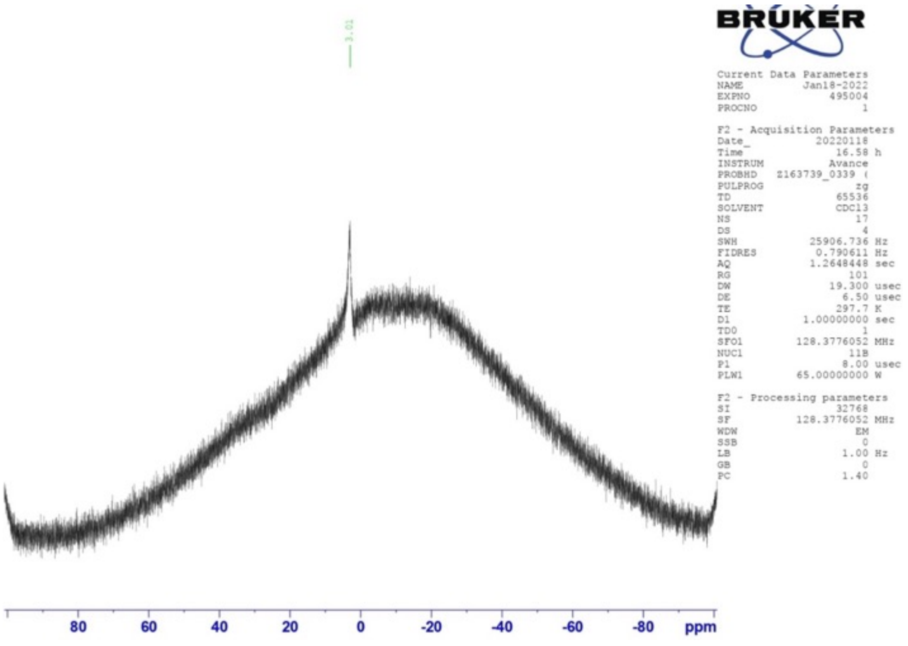

^{11}B NMR (128 MHz, $CDCl_3$)

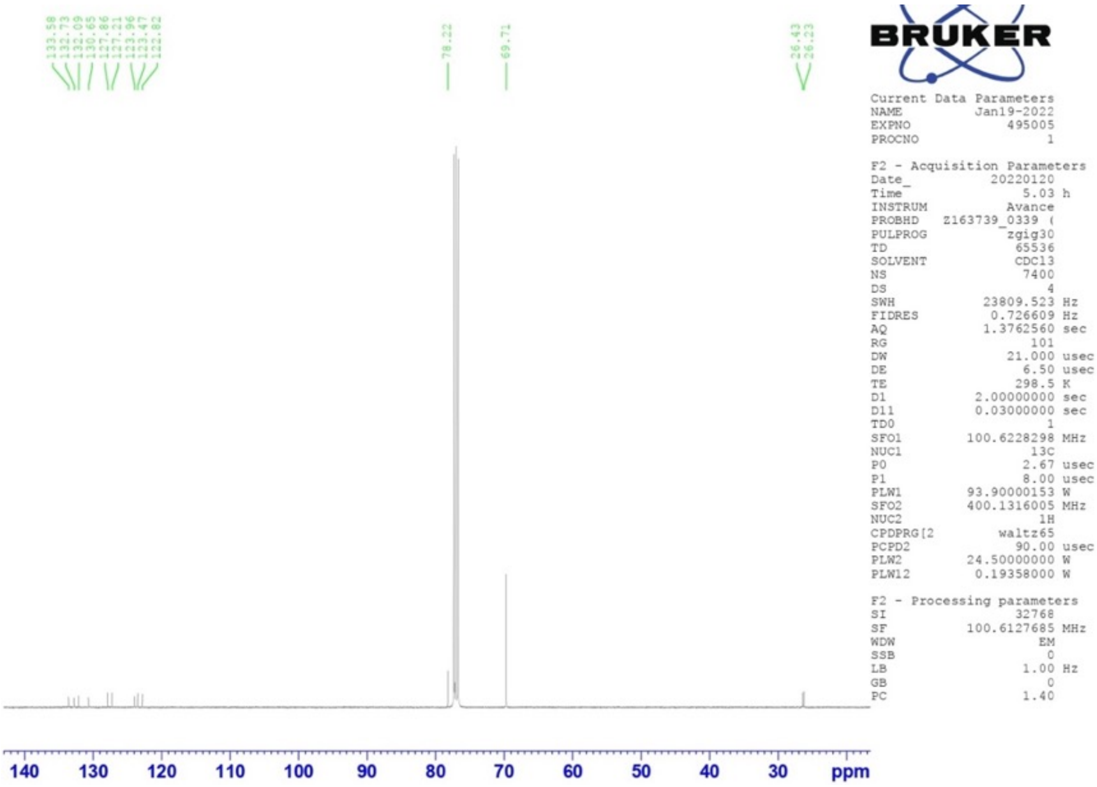

$^{13}C\{^{1}H\}$ NMR (101 MHz, $CDCl_3$)

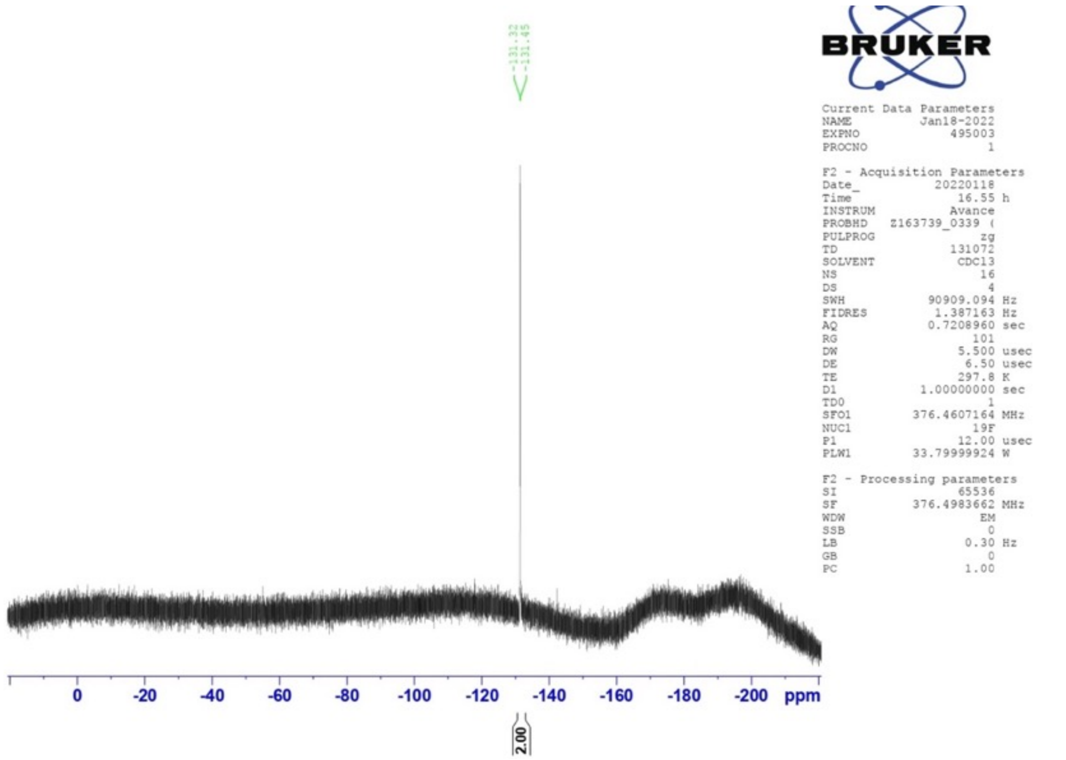

^{19}F NMR (376 MHz, $CDCl_3$)

2-(Difluoromethyl)-4,4,5,5-tetramethyl-2-(naphthalen-1-yl)-1,3,2-dioxaborolan-2-uide 18-crown-6-ether complex (1G)

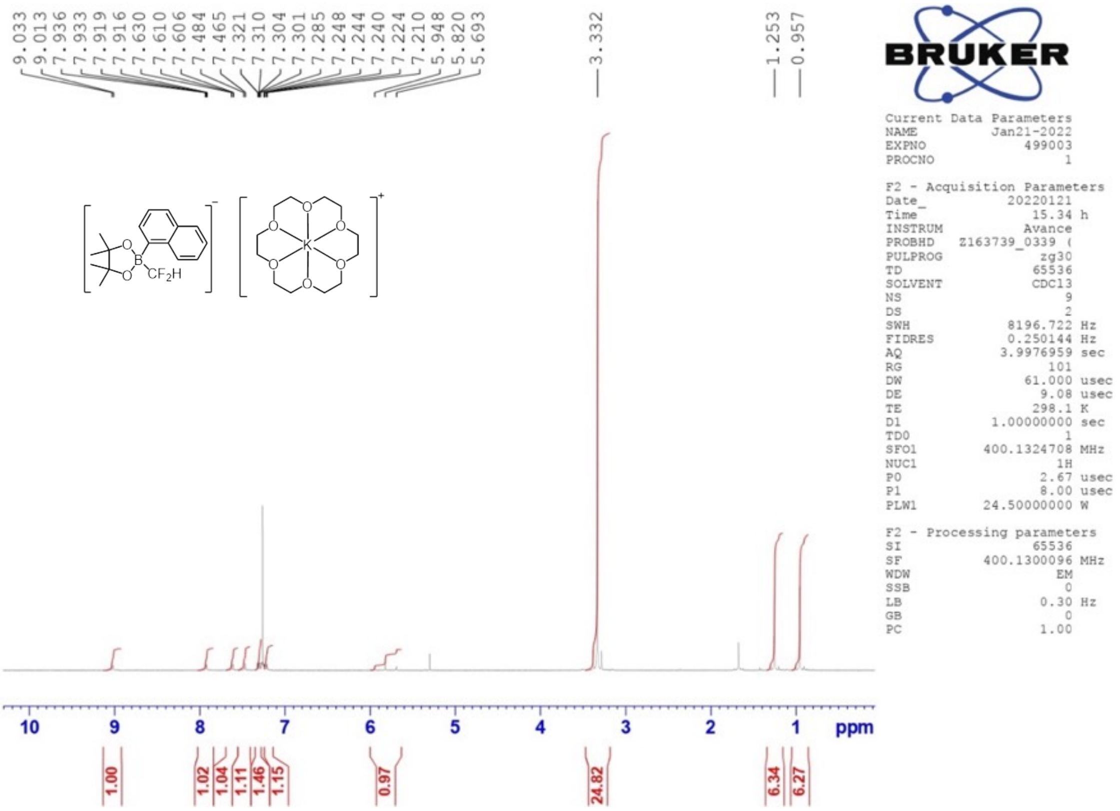

^{1}H NMR (400 MHz, $CDCl_3$)

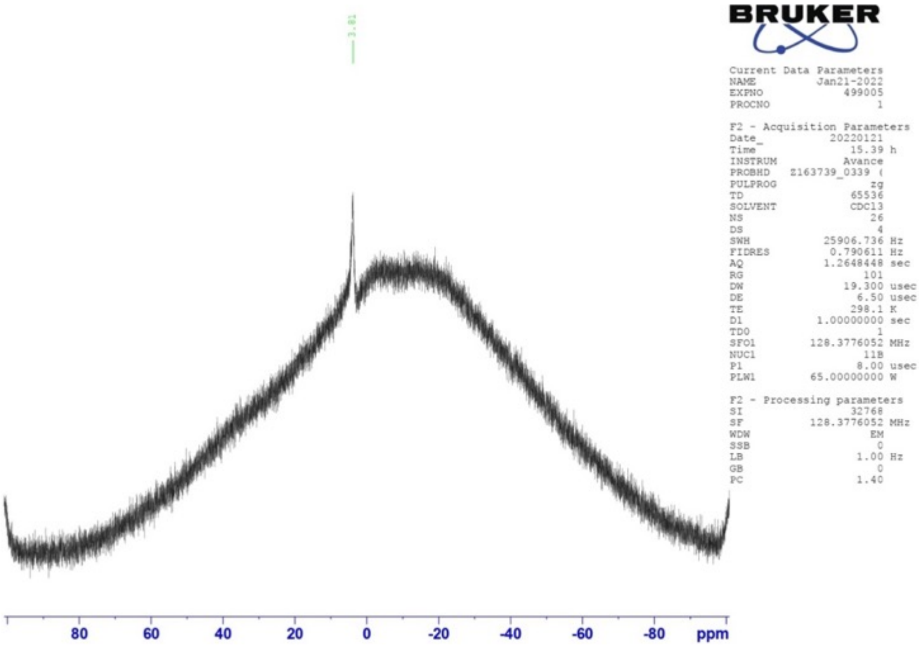

^{11}B NMR (128 MHz, $CDCl_3$)

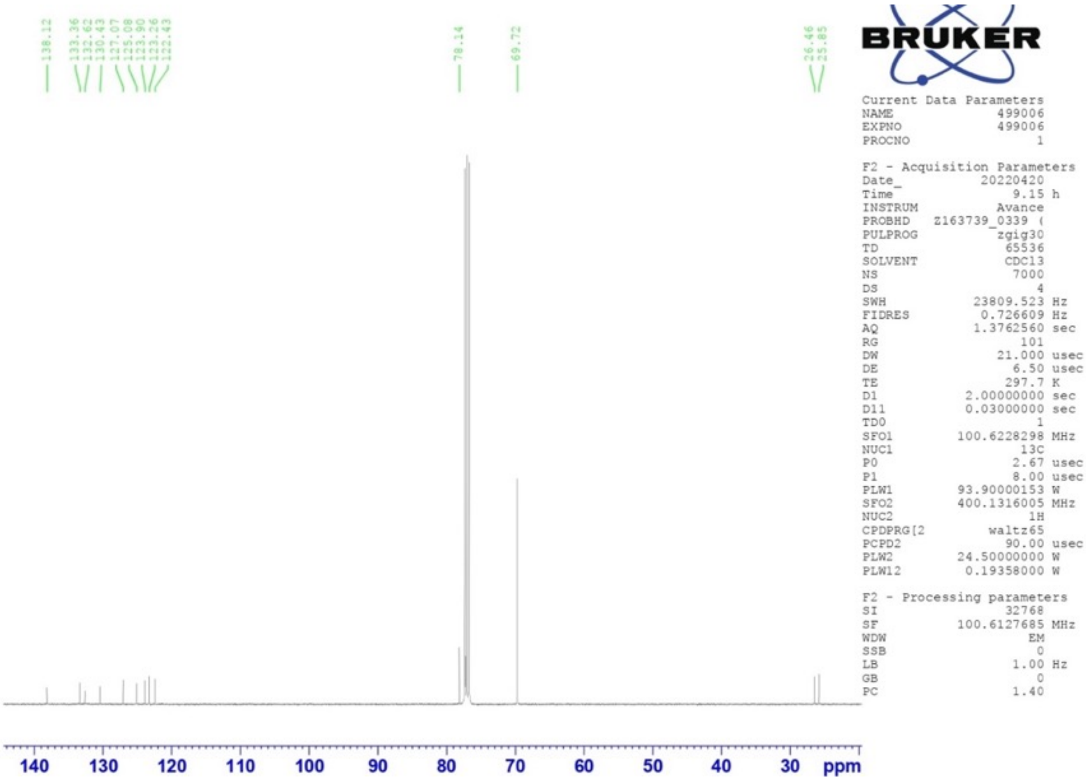

$^{13}C\{^{1}H\}$ NMR (101 MHz, $CDCl_3$)

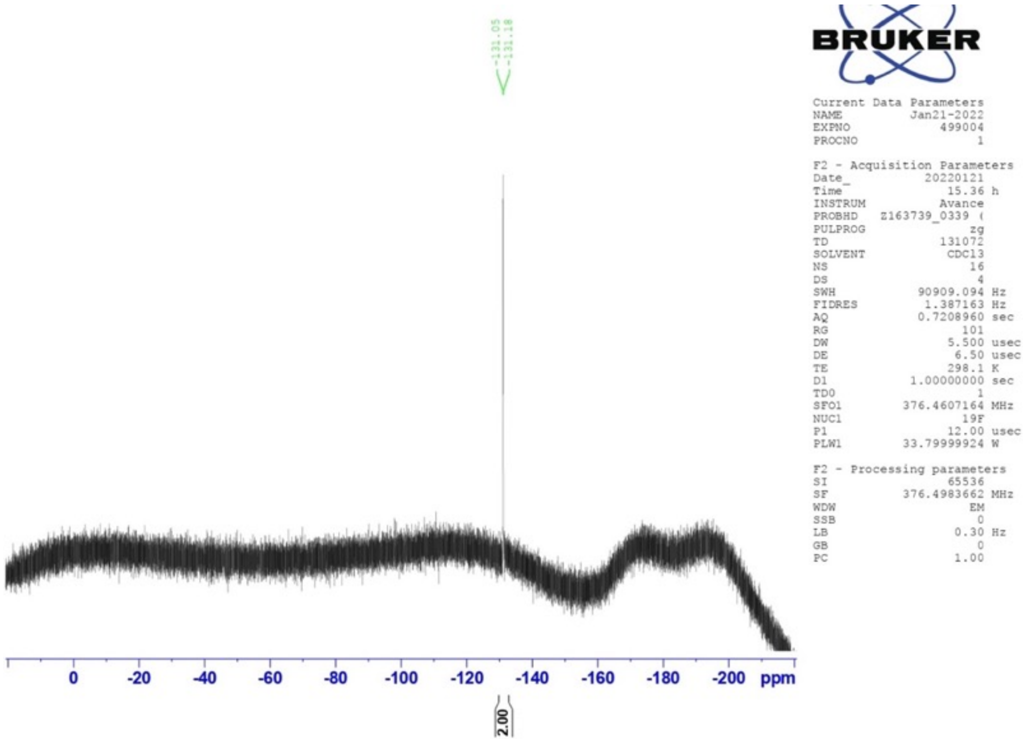

^{19}F NMR (376 MHz, $CDCl_3$)

2-(Difluoromethyl)-4,4,5,5-tetramethyl-2-(thiophen-3-yl)-1,3,2-dioxaborolan-2-uide 18-crown-6-ether complex (1H)

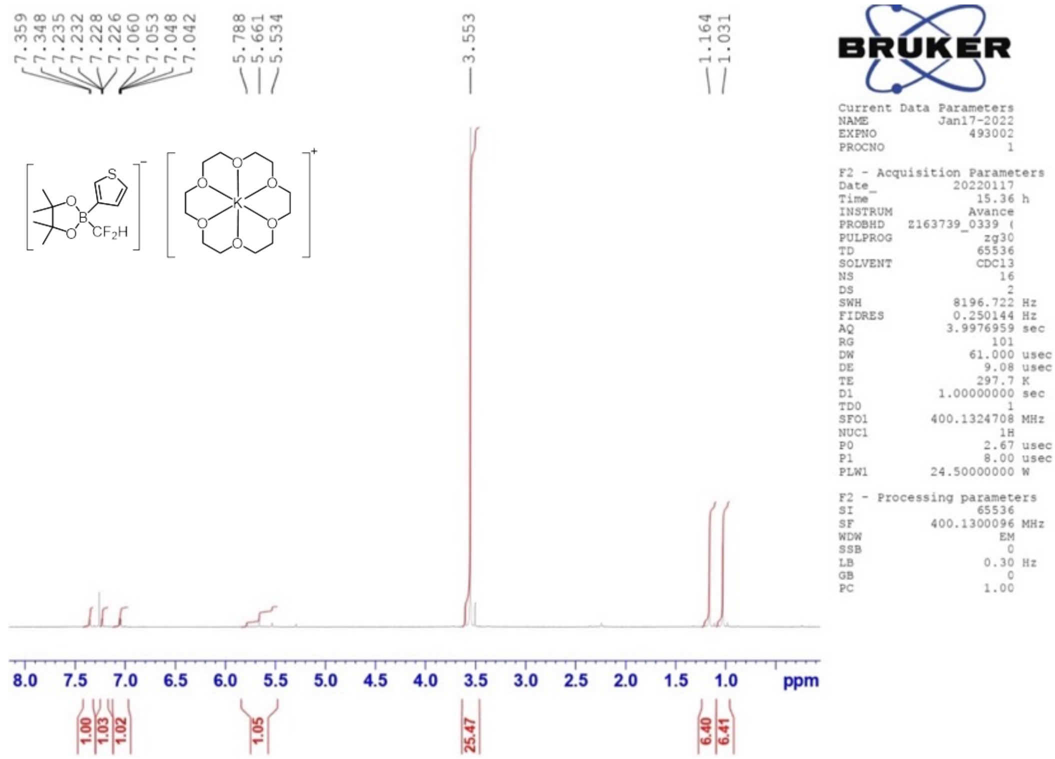

^{1}H NMR (400 MHz, $CDCl_3$)

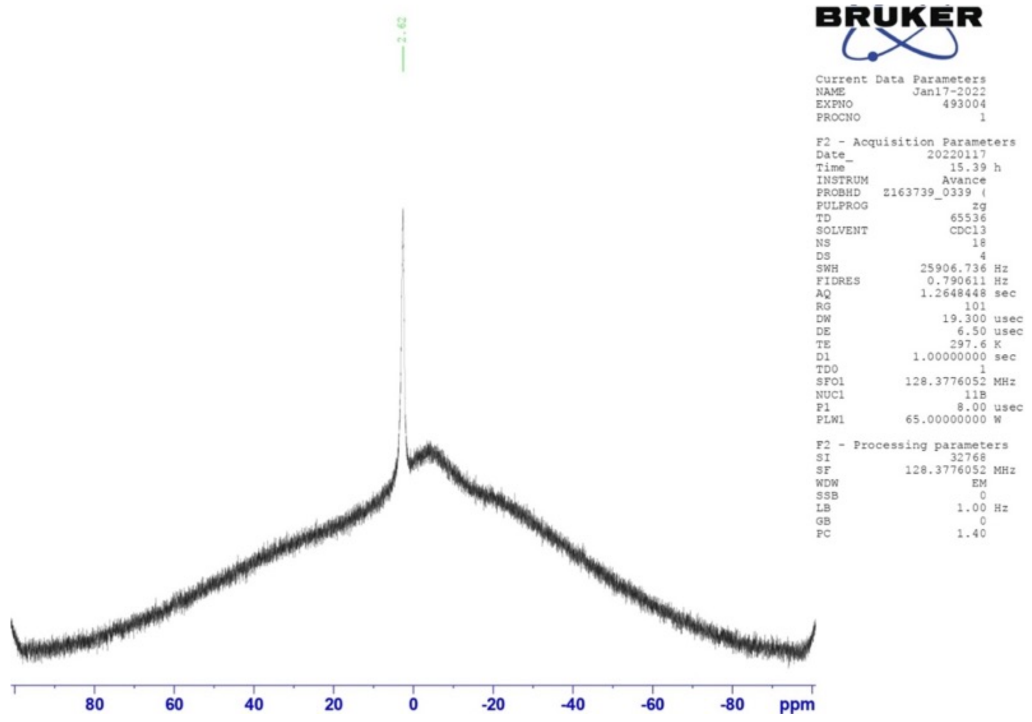

^{11}B NMR (128 MHz, $CDCl_3$)

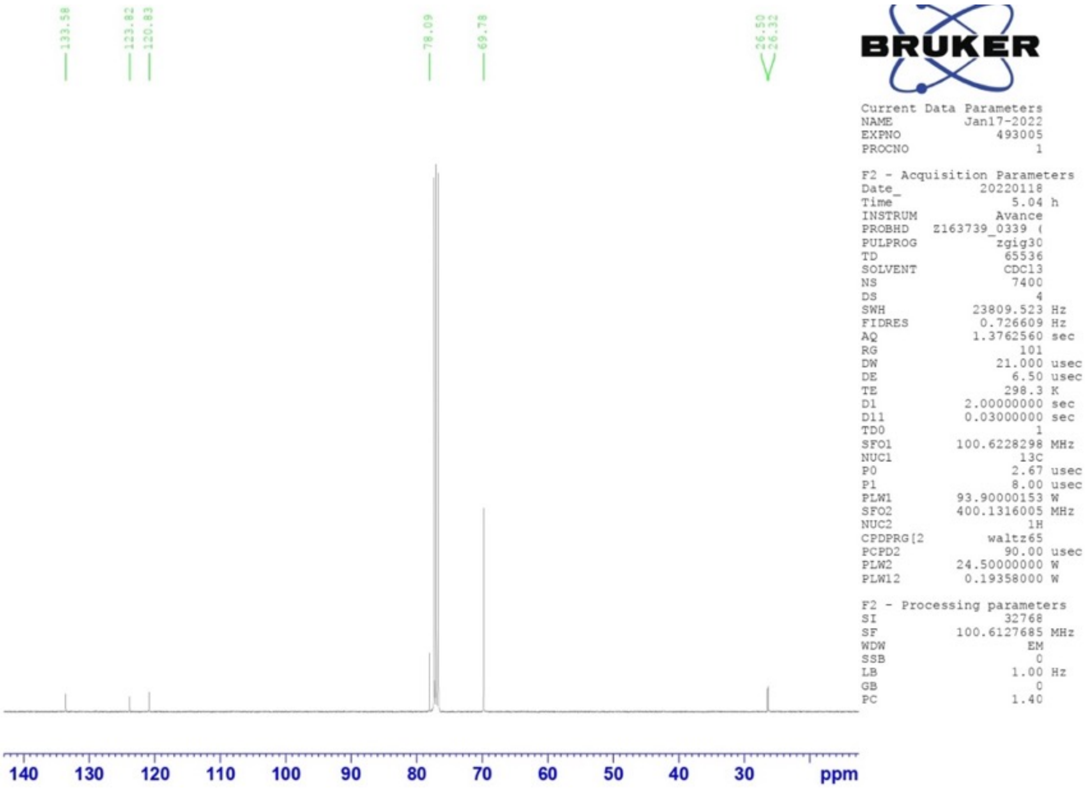

$^{13}C\{^{1}H\}$ NMR (101 MHz, $CDCl_3$)

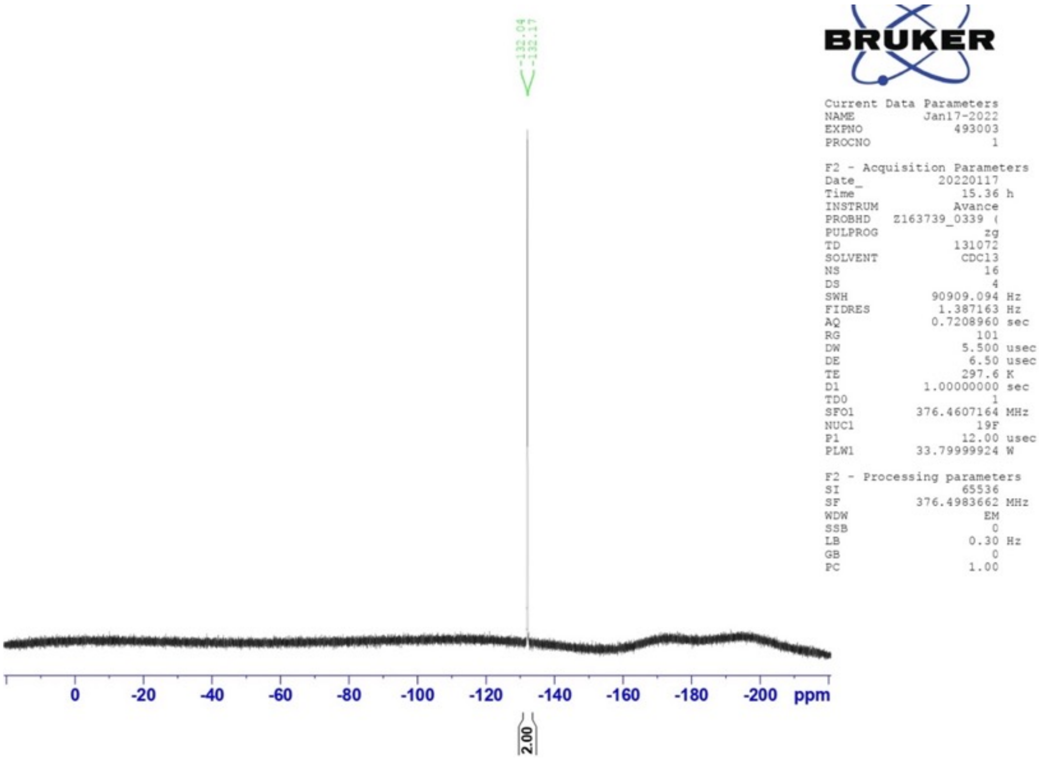

^{19}F NMR (376 MHz, $CDCl_3$)

2-(Difluoromethyl)-4,4,5,5-tetramethyl-2-(thiophen-2-yl)-1,3,2-dioxaborolan-2-uide 18-crown-6-ether complex (1I)

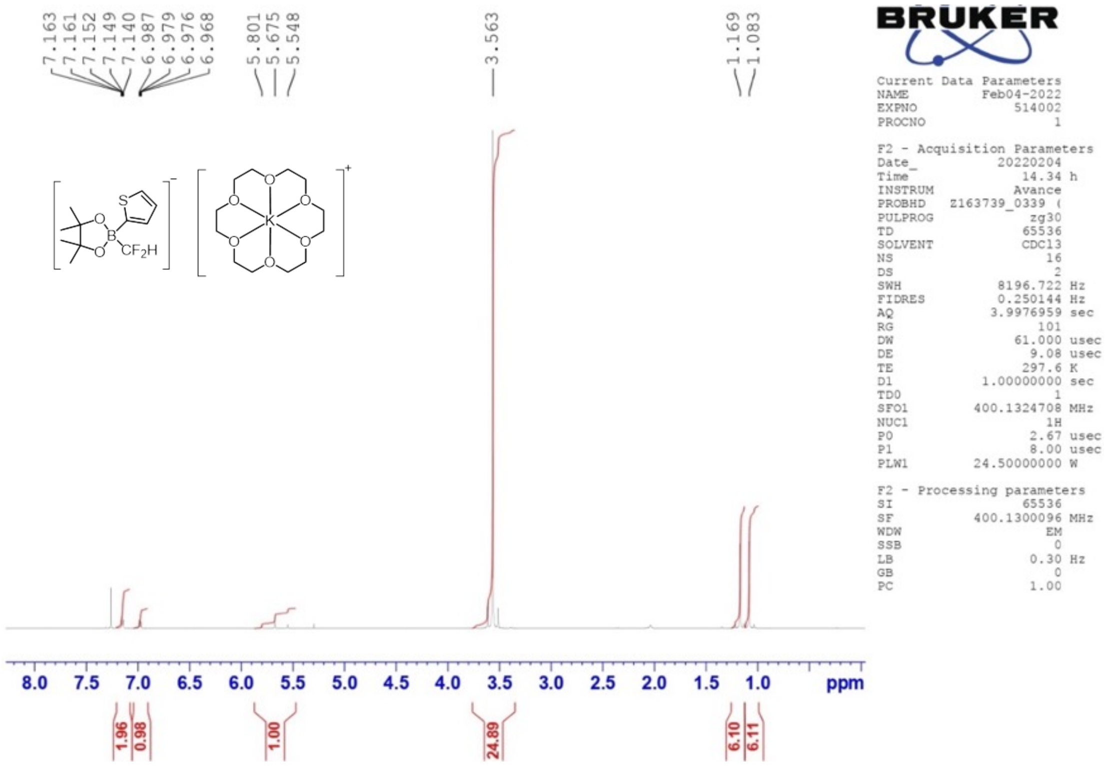

^{1}H NMR (400 MHz, $CDCl_3$)

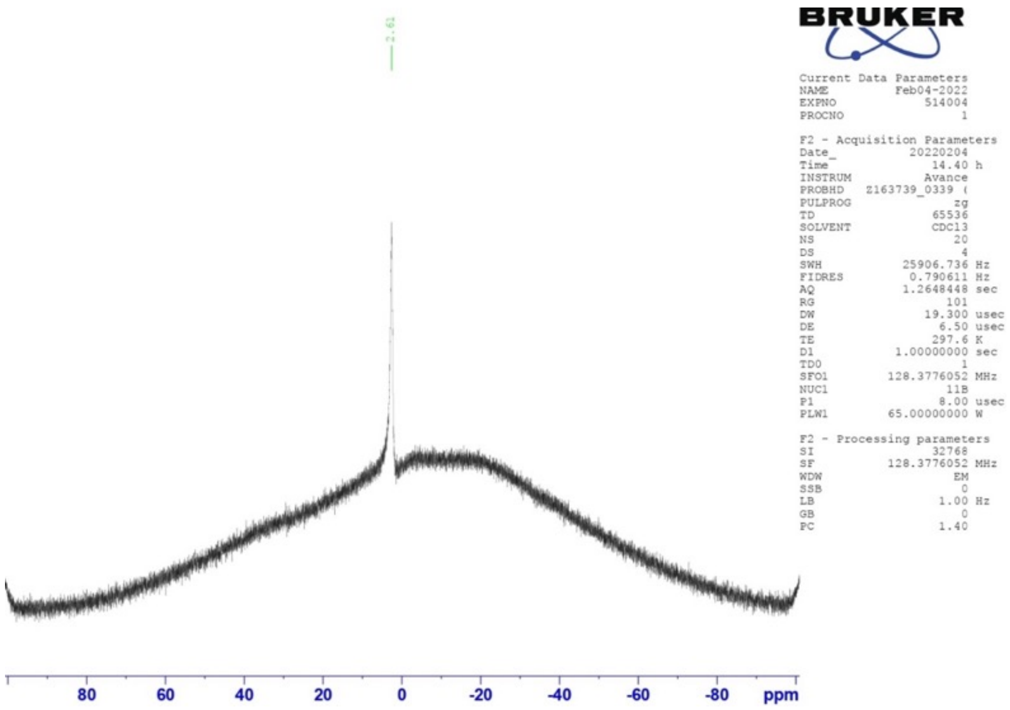

^{11}B NMR (128 MHz, $CDCl_3$)

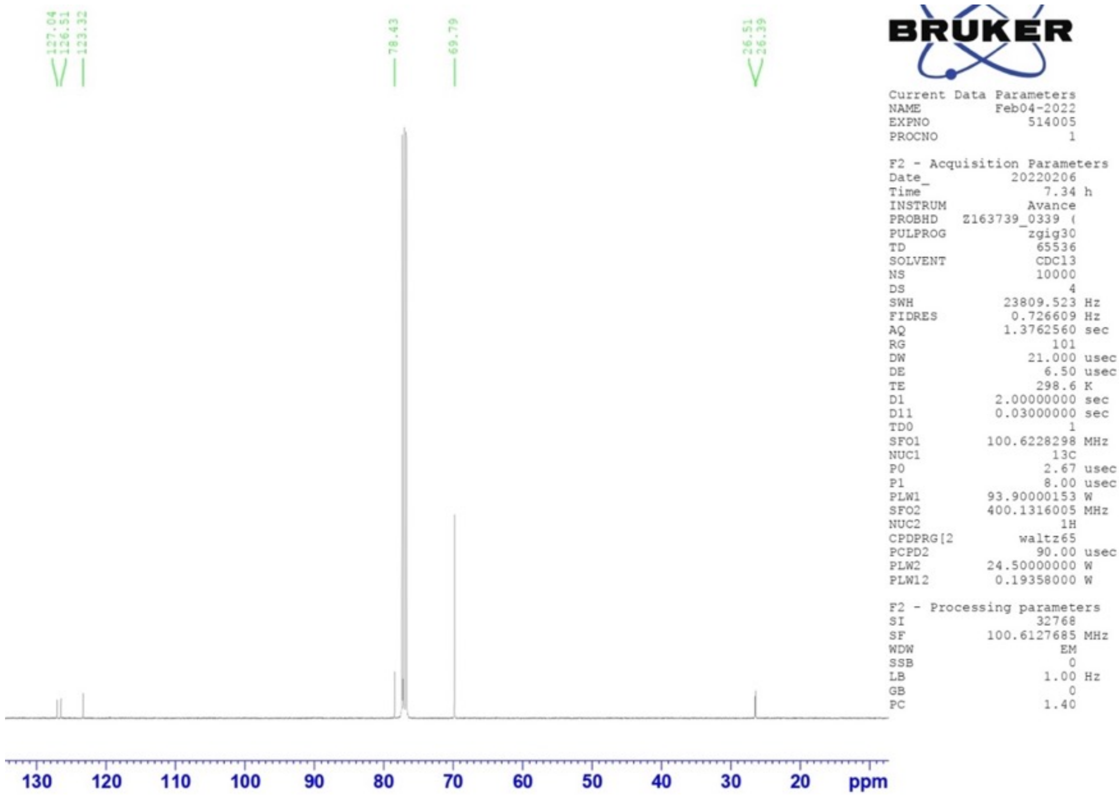

$^{13}C\{^{1}H\}$ NMR (101 MHz, $CDCl_3$)

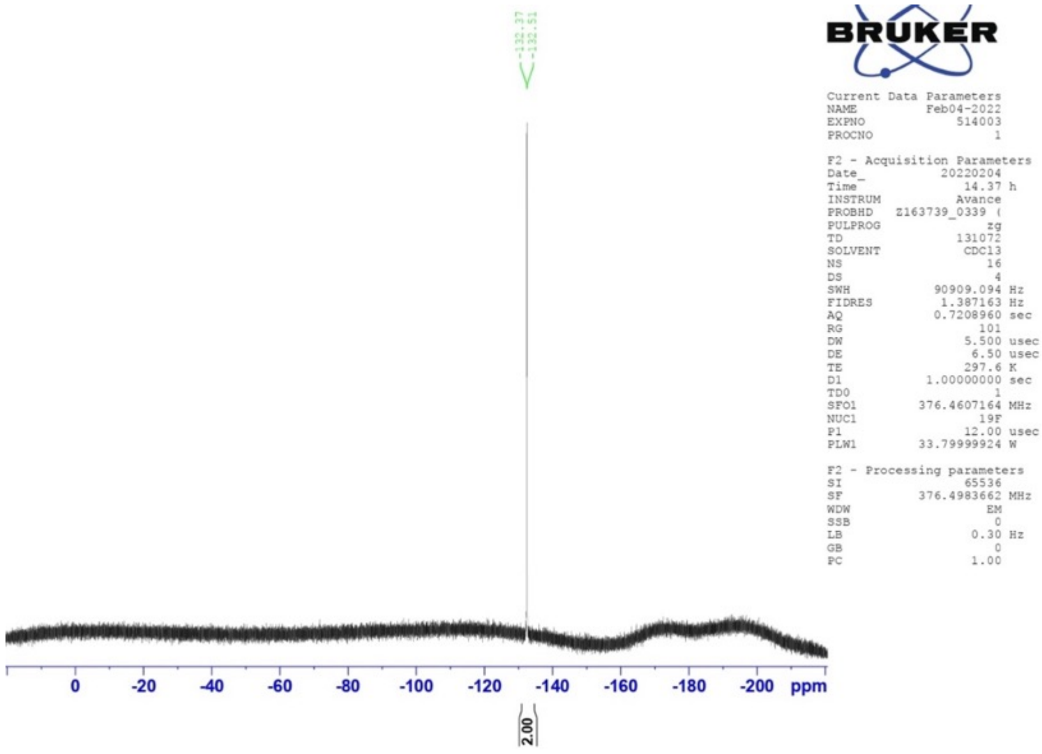

^{19}F NMR (376 MHz, $CDCl_3$)

2-(Difluoromethyl)-2-(4-(dimethylamino)phenyl)-4,4,5,5-tetramethyl-1,3,2-dioxaborolan-2-uide 18-crown-6-ether complex (1J)

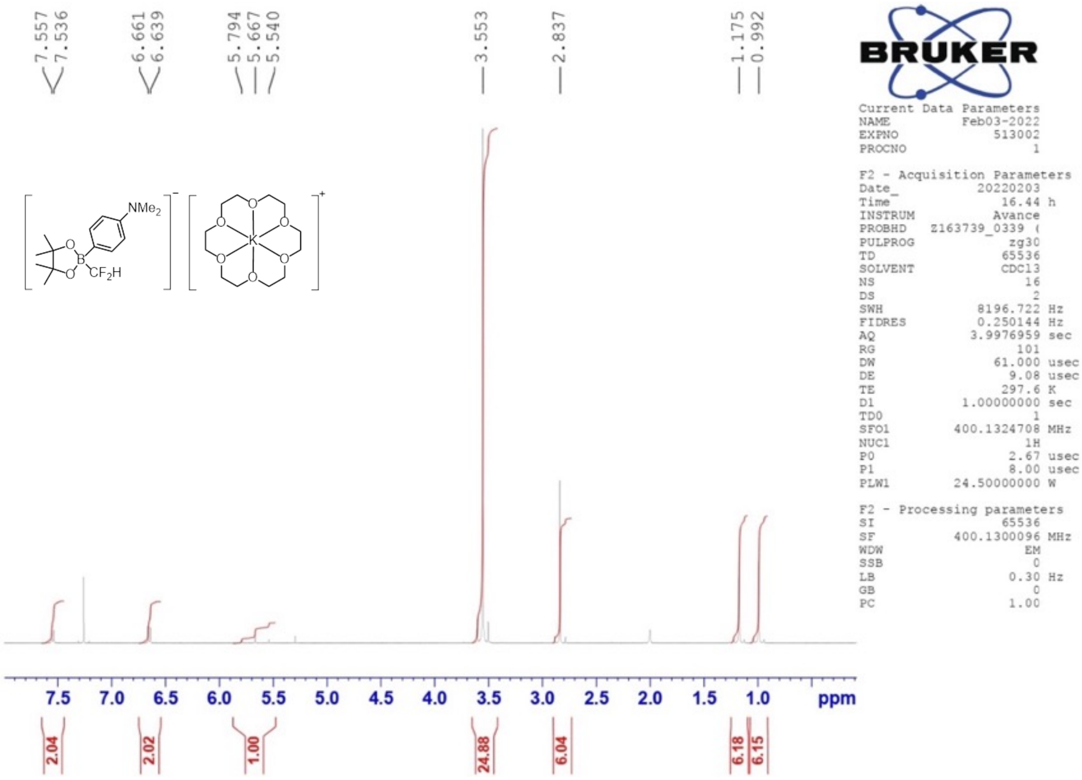

^{1}H NMR (400 MHz, $CDCl_3$)

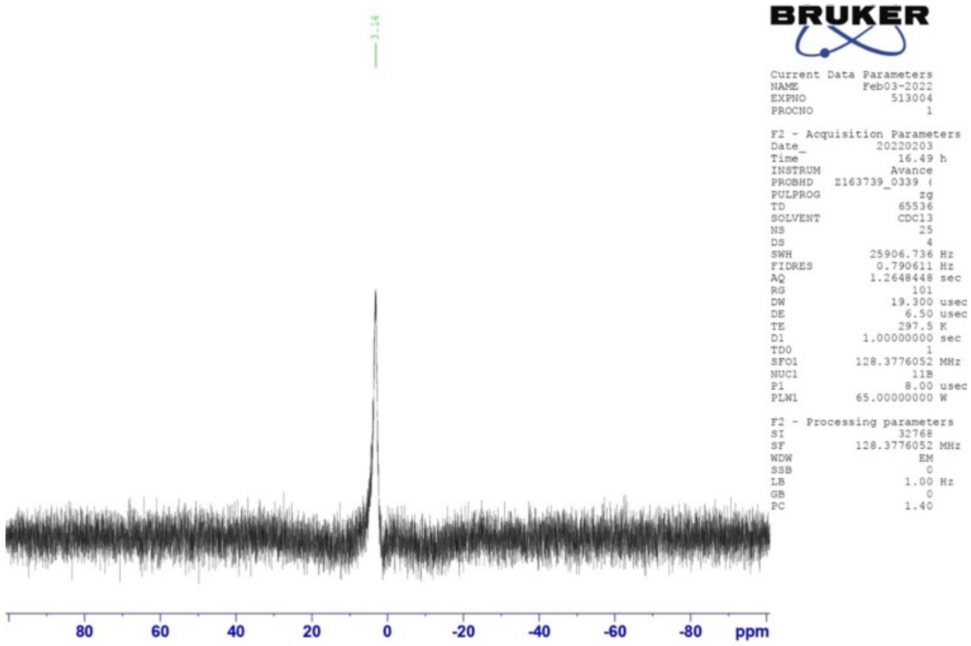

^{11}B NMR (128 MHz, $CDCl_3$)

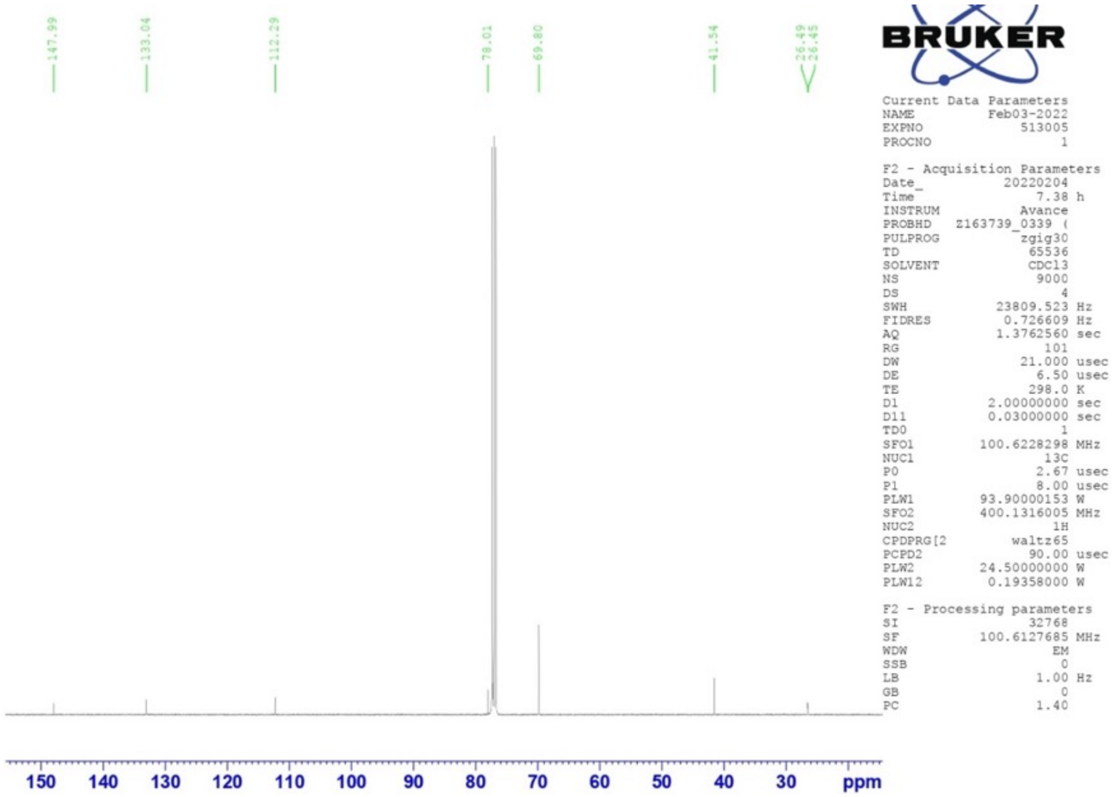

$^{13}C\{^{1}H\}$ NMR (101 MHz, $CDCl_3$)

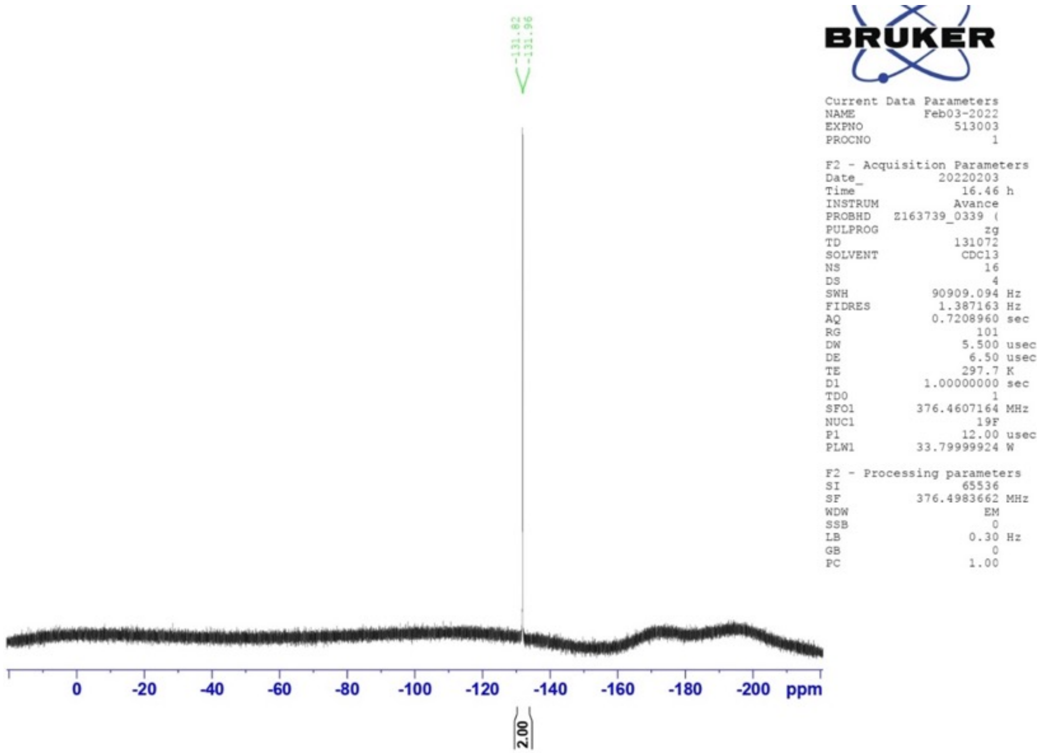

^{19}F NMR (376 MHz, $CDCl_3$)

2-(4-(Diethylamino)phenyl)-2-(difluoromethyl)-4,4,5,5-tetramethyl-1,3,2-dioxaborolan-2-uide 18-crown-6-ether complex (1K)

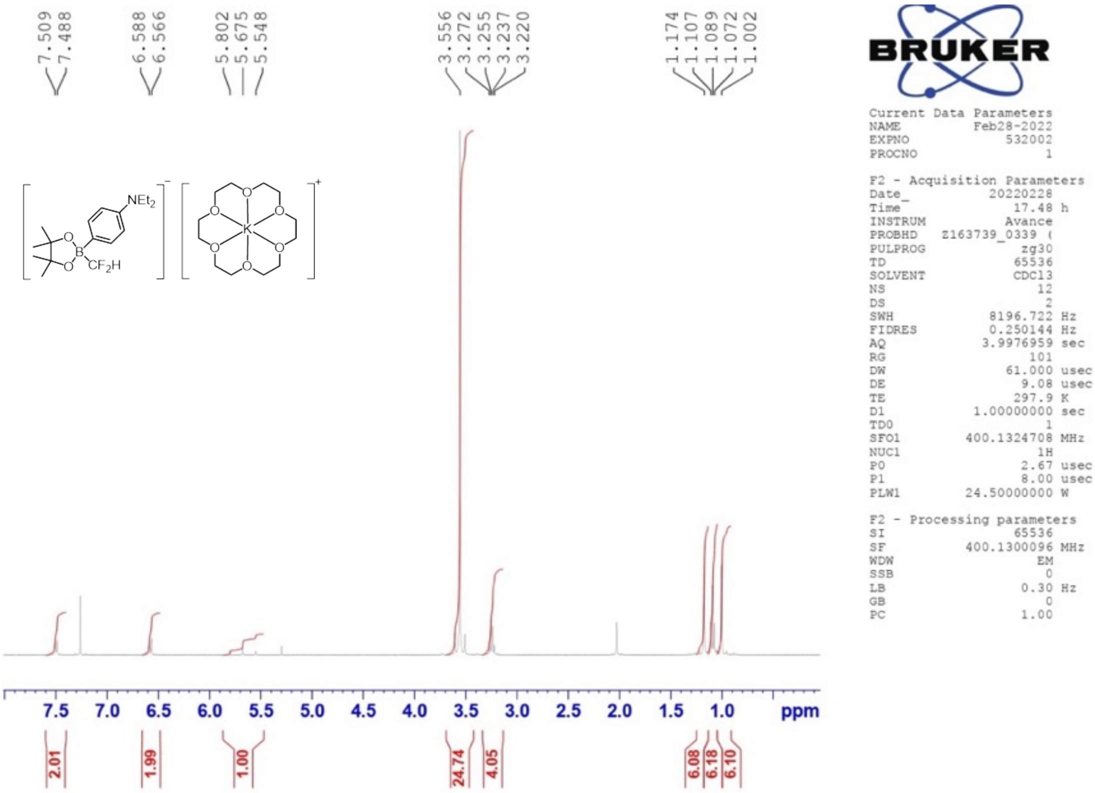

^{1}H NMR (400 MHz, $CDCl_3$)

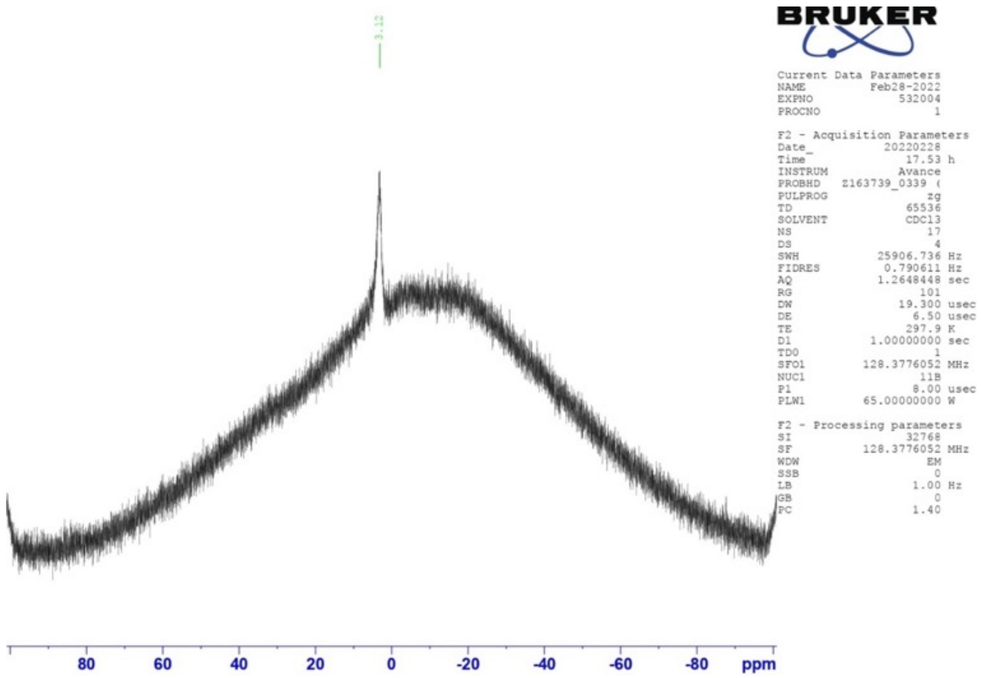

^{11}B NMR (128 MHz, $CDCl_3$)

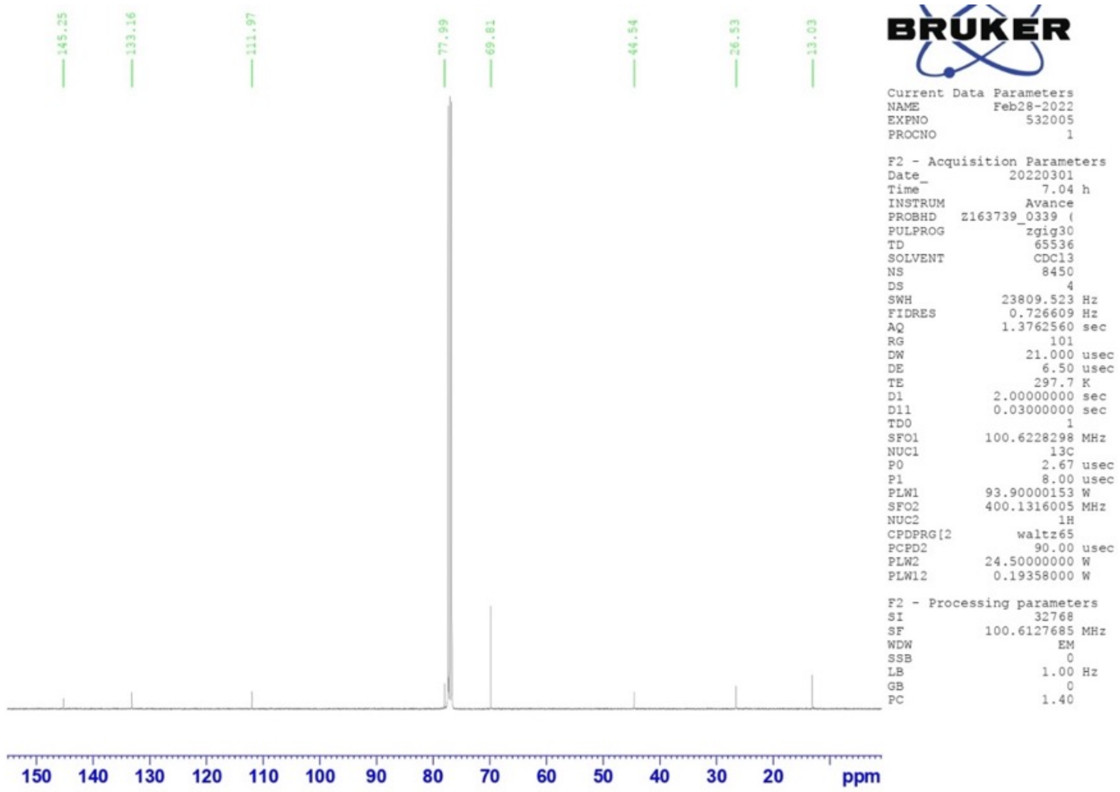

$^{13}C\{^{1}H\}$ NMR (101 MHz, $CDCl_3$)

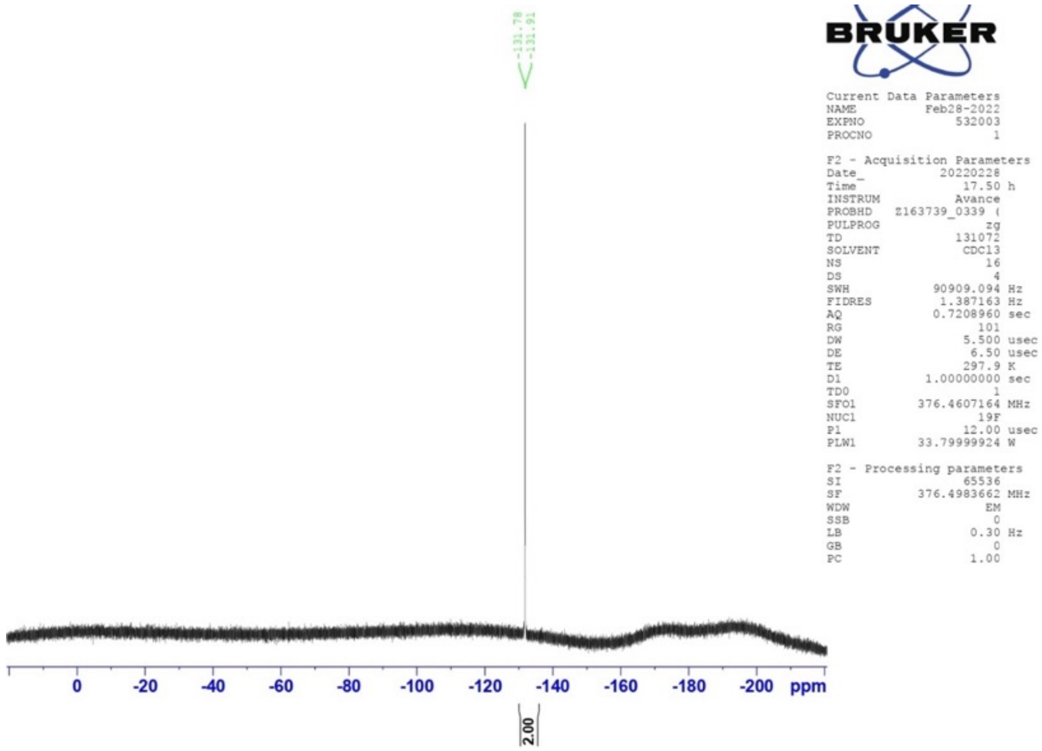

^{19}F NMR (376 MHz, $CDCl_3$)

2-(4-(1*H*-Pyrrol-1-yl)phenyl)-2-(difluoromethyl)-4,4,5,5-tetramethyl-1,3,2-dioxaborolan-2-uide 18-crown-6-ether complex (1L)

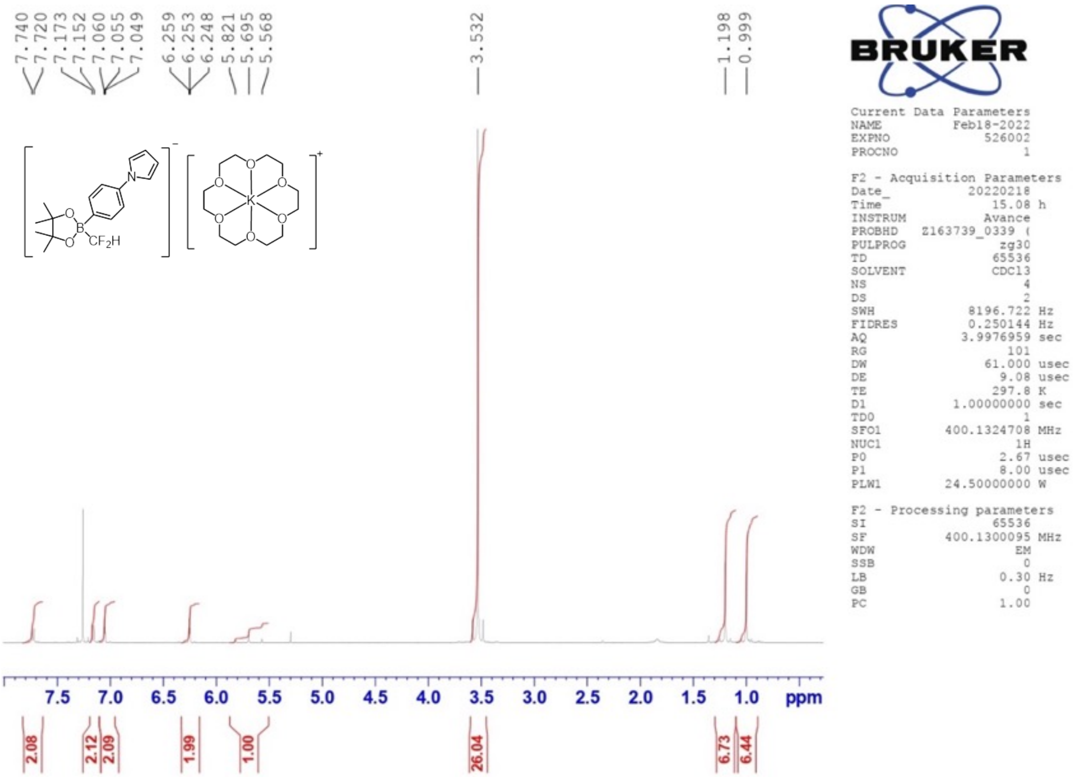

^{1}H NMR (400 MHz, $CDCl_3$)

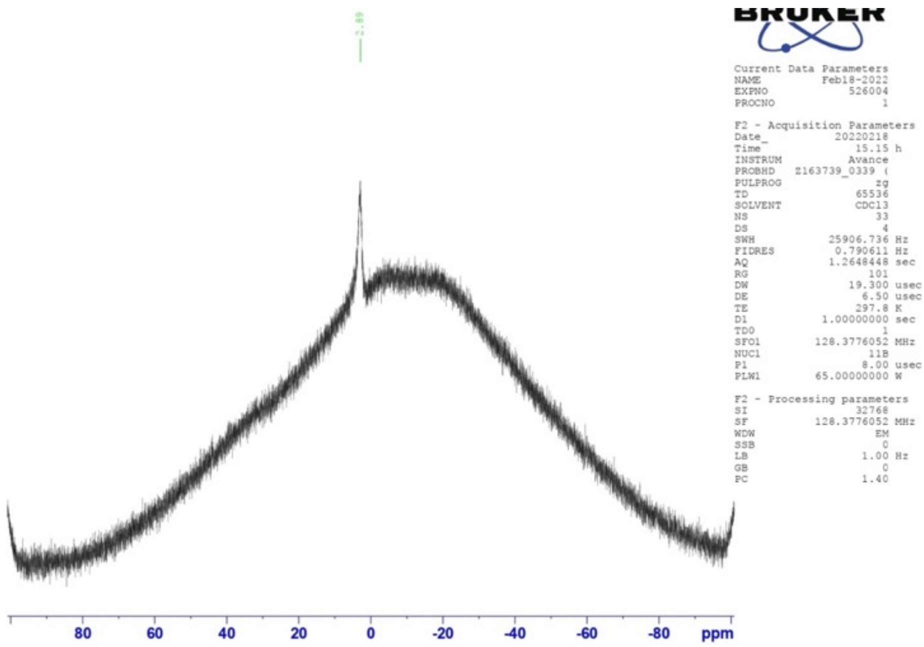

^{11}B NMR (128 MHz, $CDCl_3$)

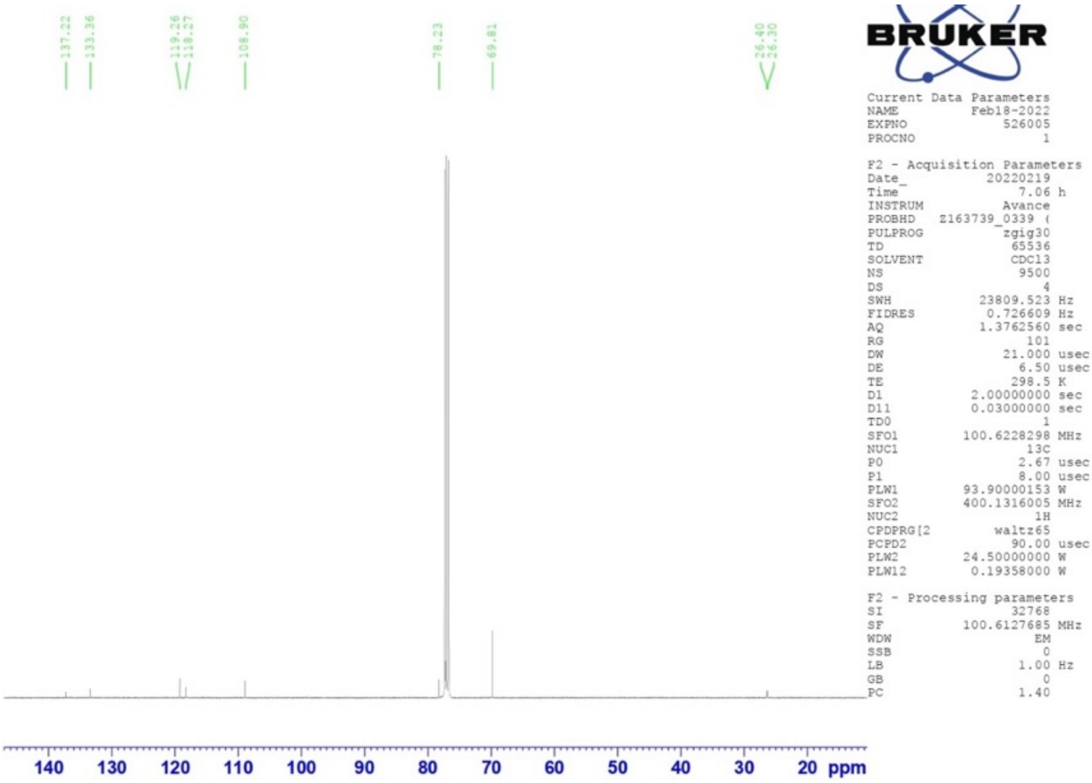

$^{13}C\{^1H\}$ NMR (101 MHz, $CDCl_3$)

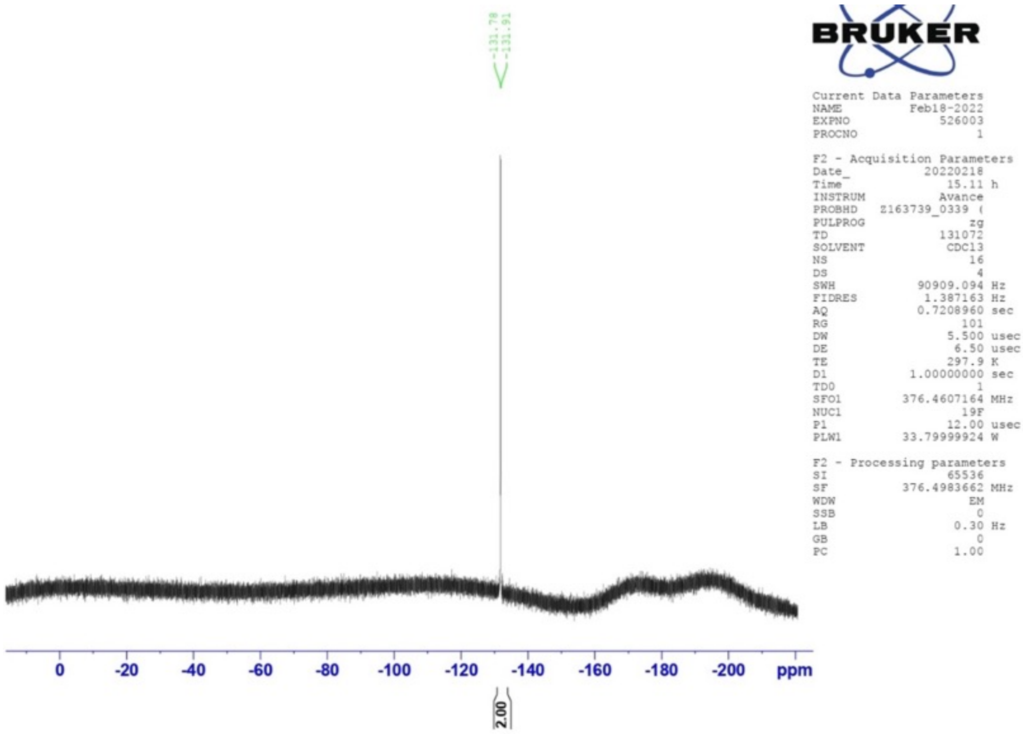

^{19}F NMR (376 MHz, $CDCl_3$)

2-(Difluoromethyl)-4,4,5,5-tetramethyl-2-(4-(piperidin-1-yl)phenyl)-1,3,2-dioxaborolan-2-uide 18-crown-6-ether complex (1N)

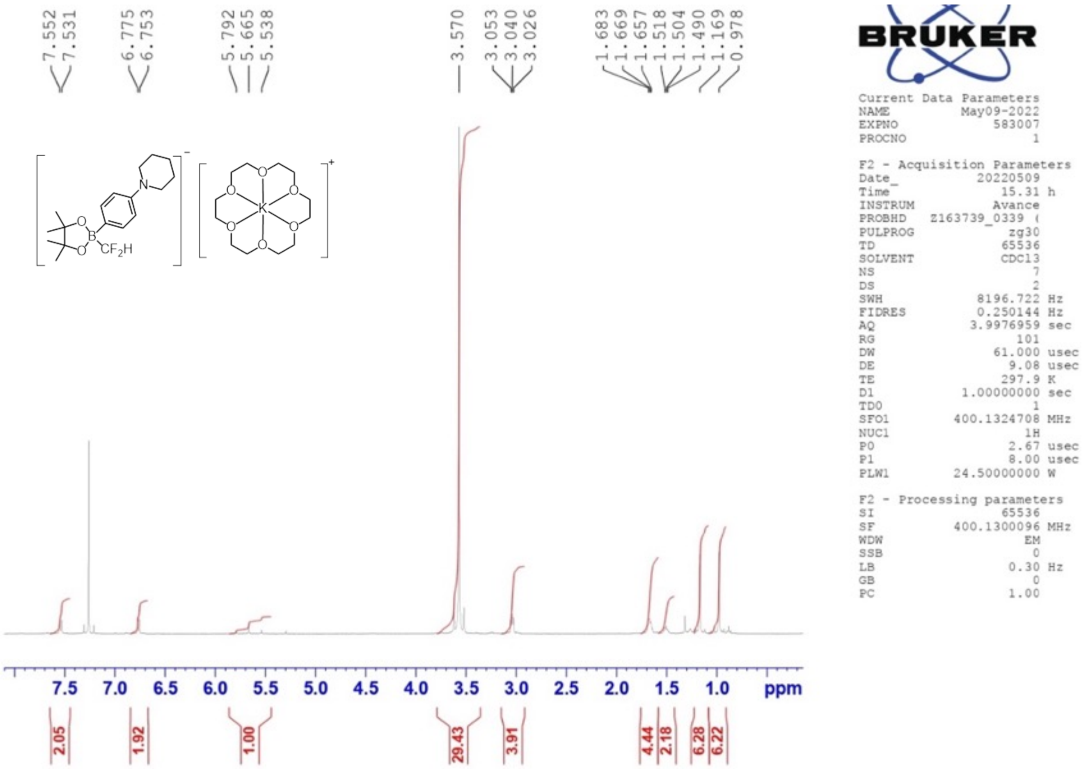

^{1}H NMR (400 MHz, $CDCl_3$)

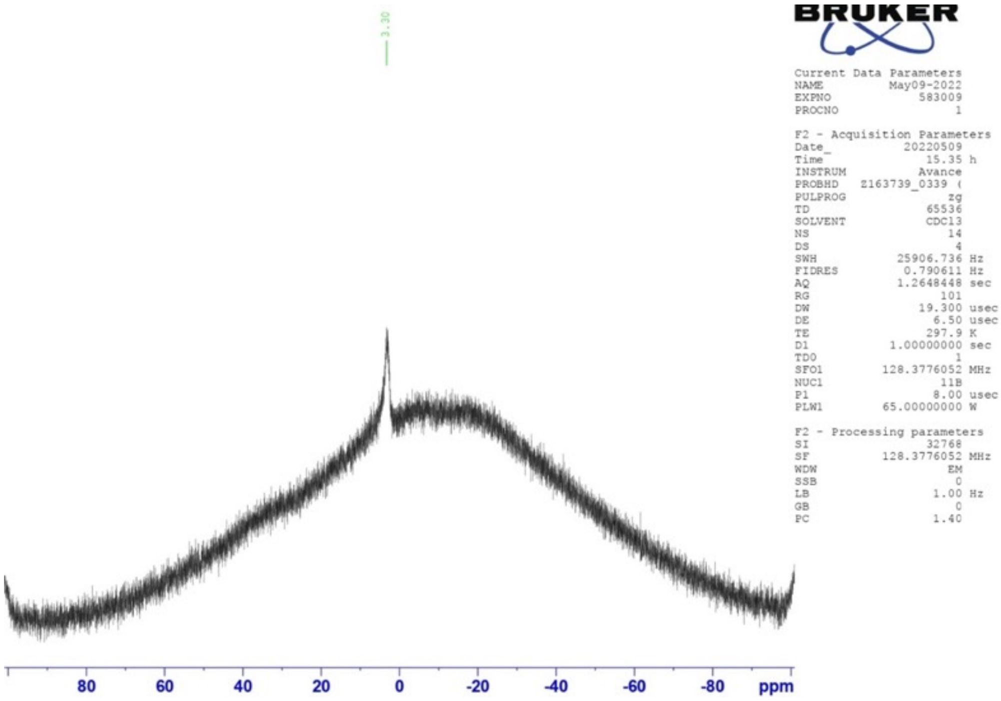

^{11}B NMR (128 MHz, $CDCl_3$)

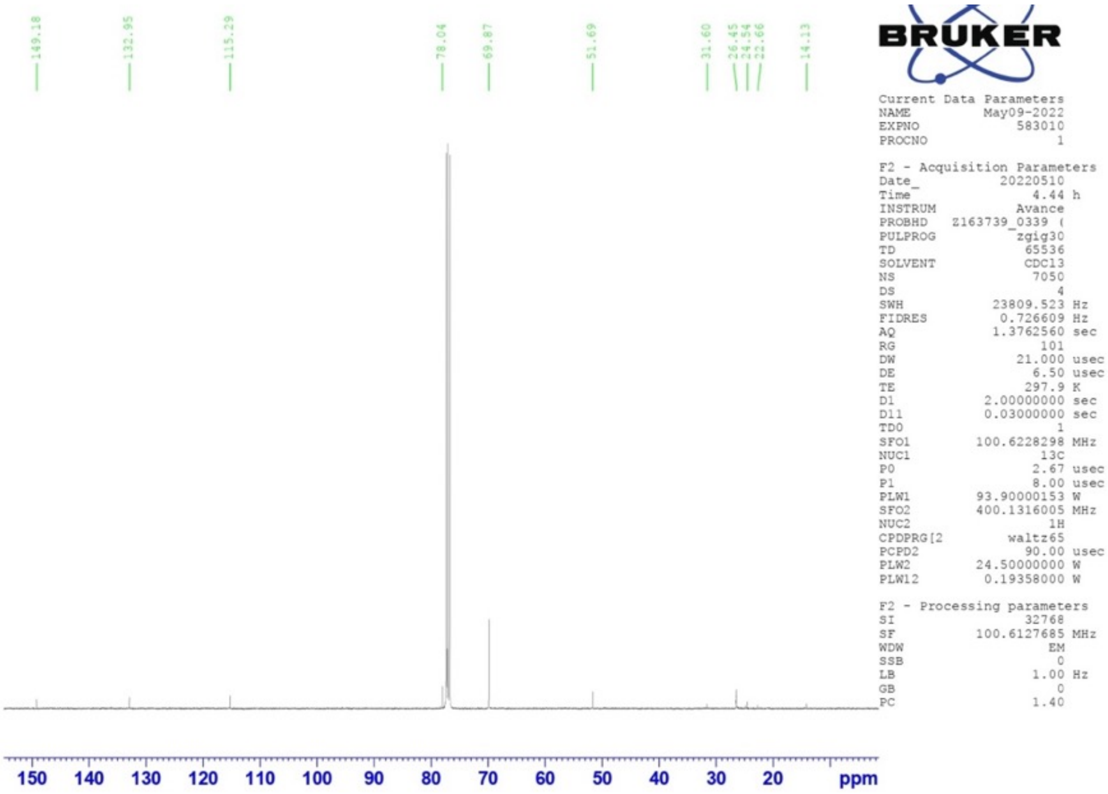

$^{13}C\{^{1}H\}$ NMR (101 MHz, $CDCl_3$)

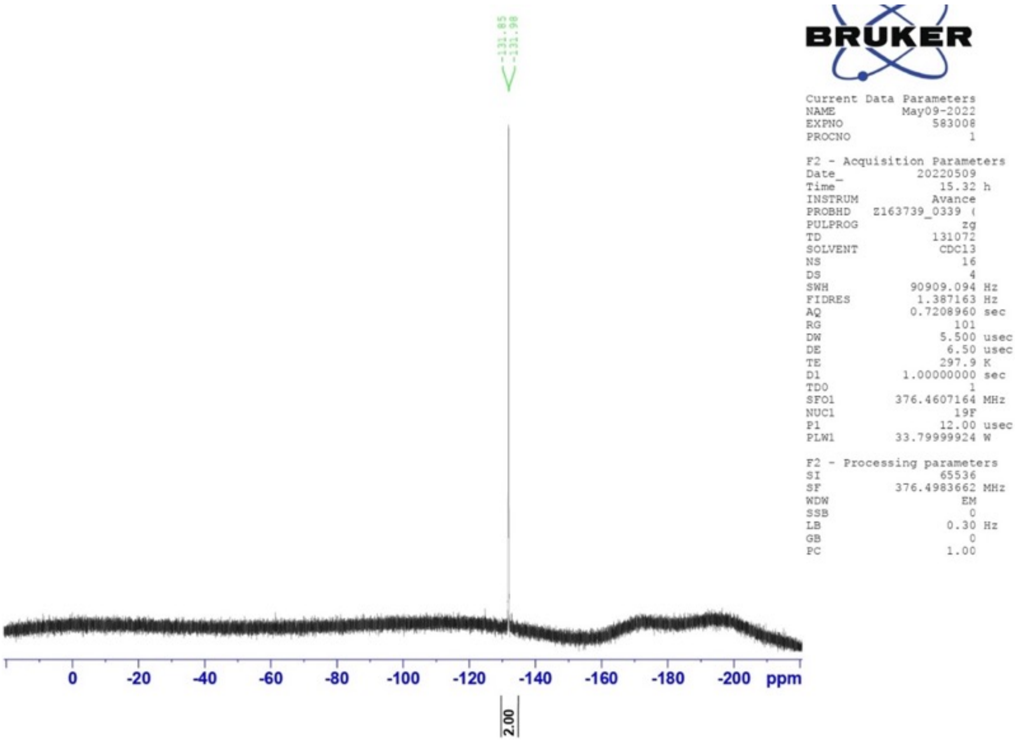

^{19}F NMR (376 MHz, $CDCl_3$)

2-(Difluoromethyl)-2-(4-methoxyphenyl)-4,4,5,5-tetramethyl-1,3,2-dioxaborolan-2-uide 18-crown-6-ether complex (1P)

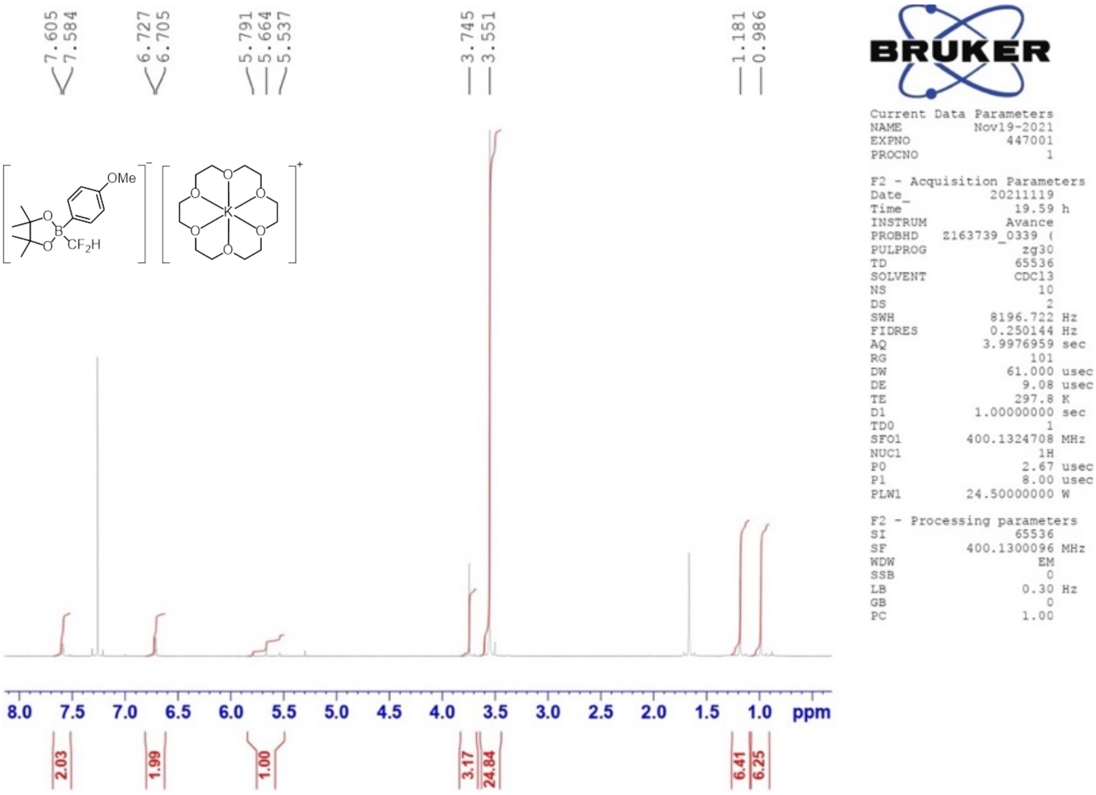

^{1}H NMR (400 MHz, $CDCl_3$)

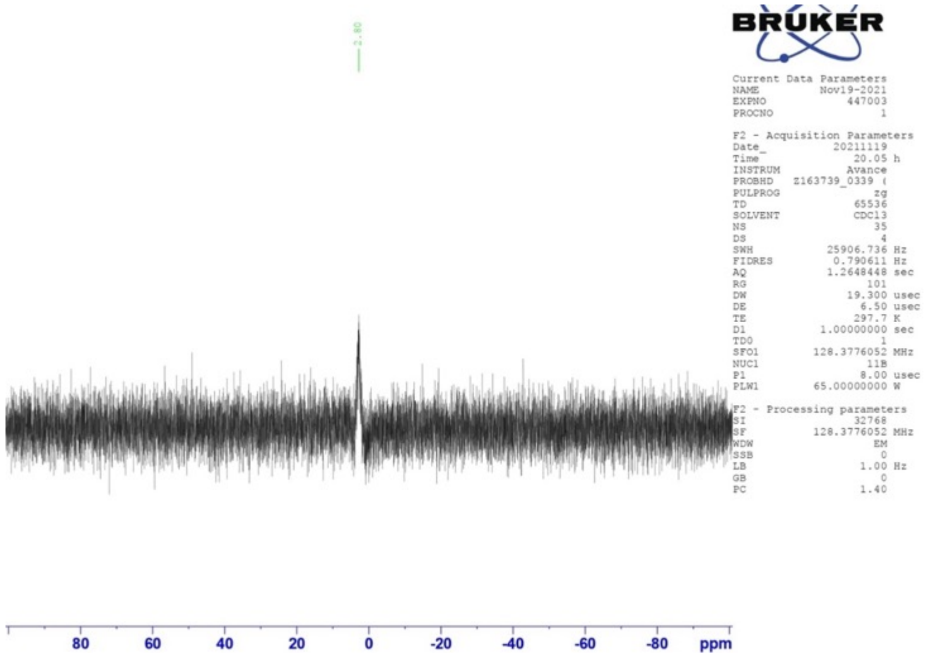

^{11}B NMR (128 MHz, $CDCl_3$)

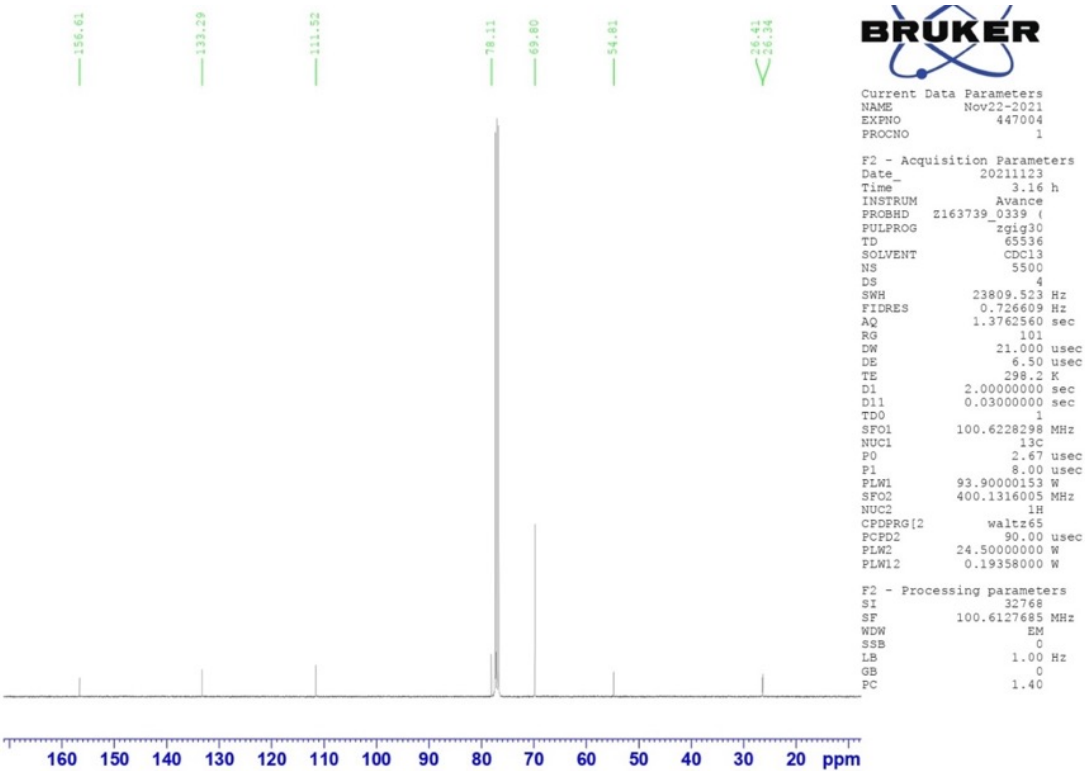

$^{13}C\{^{1}H\}$ NMR (101 MHz, $CDCl_3$)

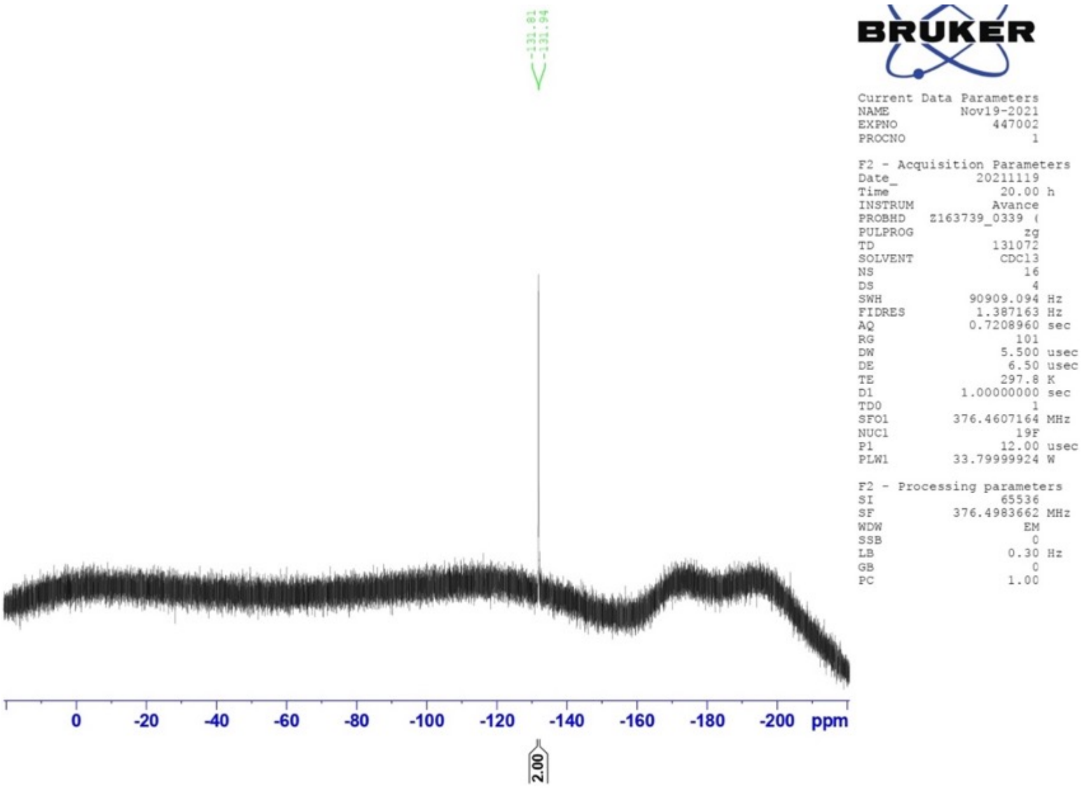

^{19}F NMR (376 MHz, $CDCl_3$)

2-(Difluoromethyl)-2-(3,5-dimethoxyphenyl)-4,4,5,5-tetramethyl-1,3,2-dioxaborolan-2-uide 18-crown-6-ether complex (1Q)

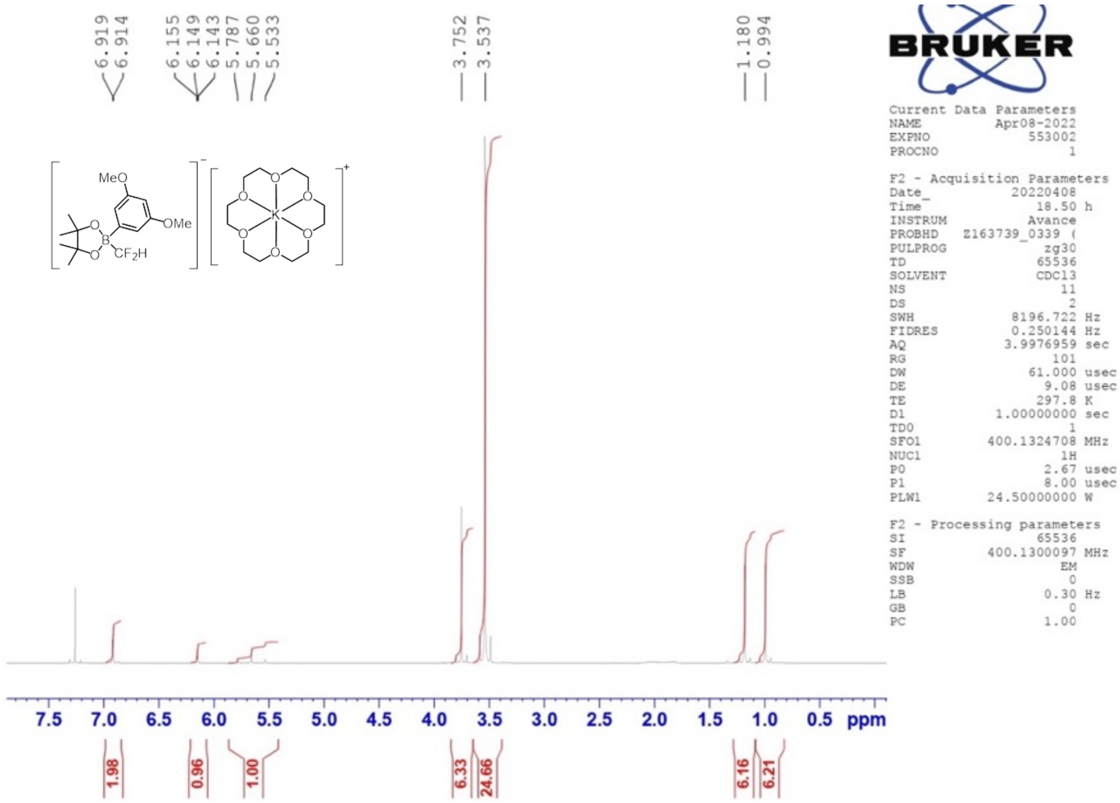

^{1}H NMR (400 MHz, $CDCl_3$)

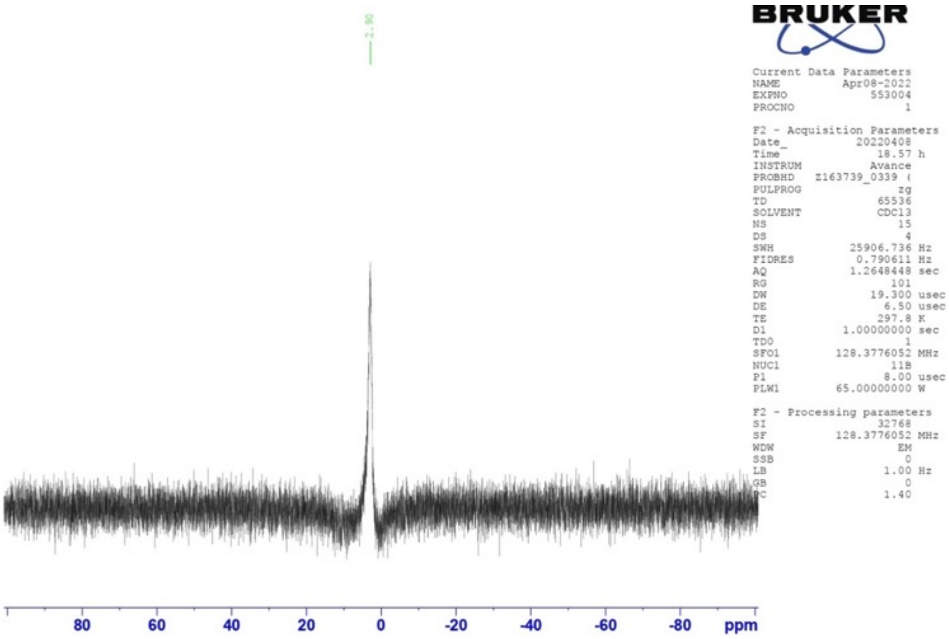

^{11}B NMR (128 MHz, $CDCl_3$)

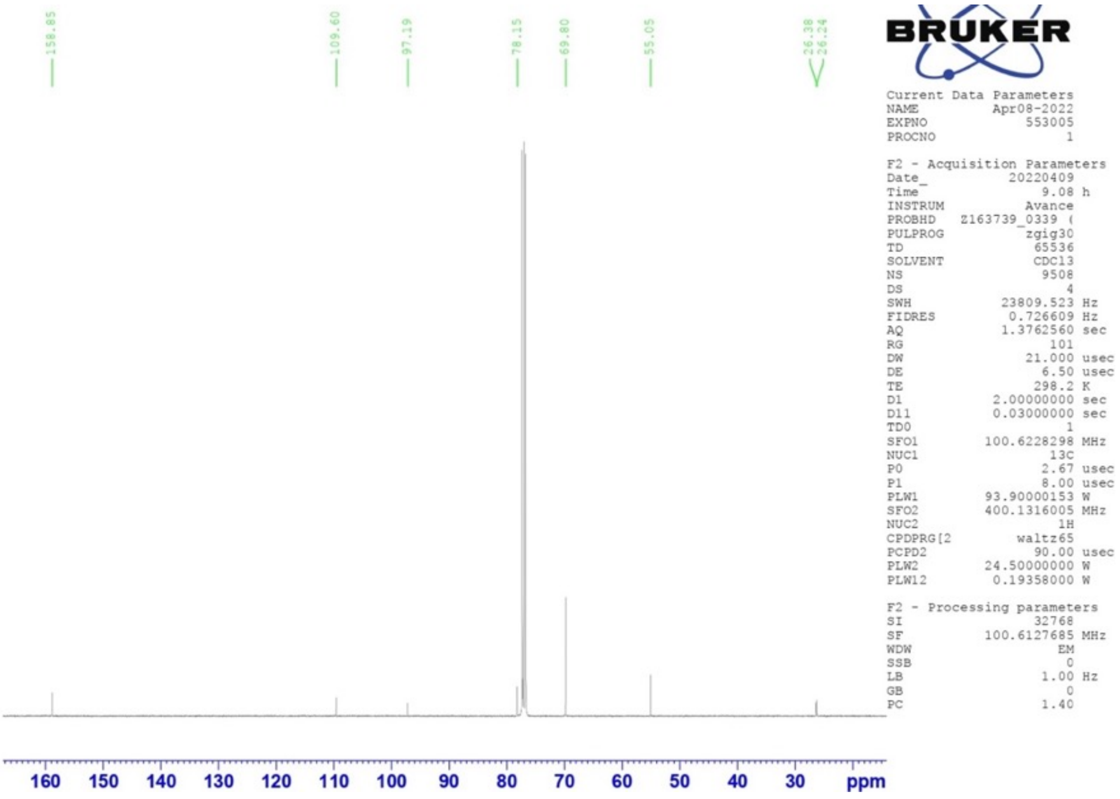

$^{13}C\{^1H\}$ NMR (101 MHz, $CDCl_3$)

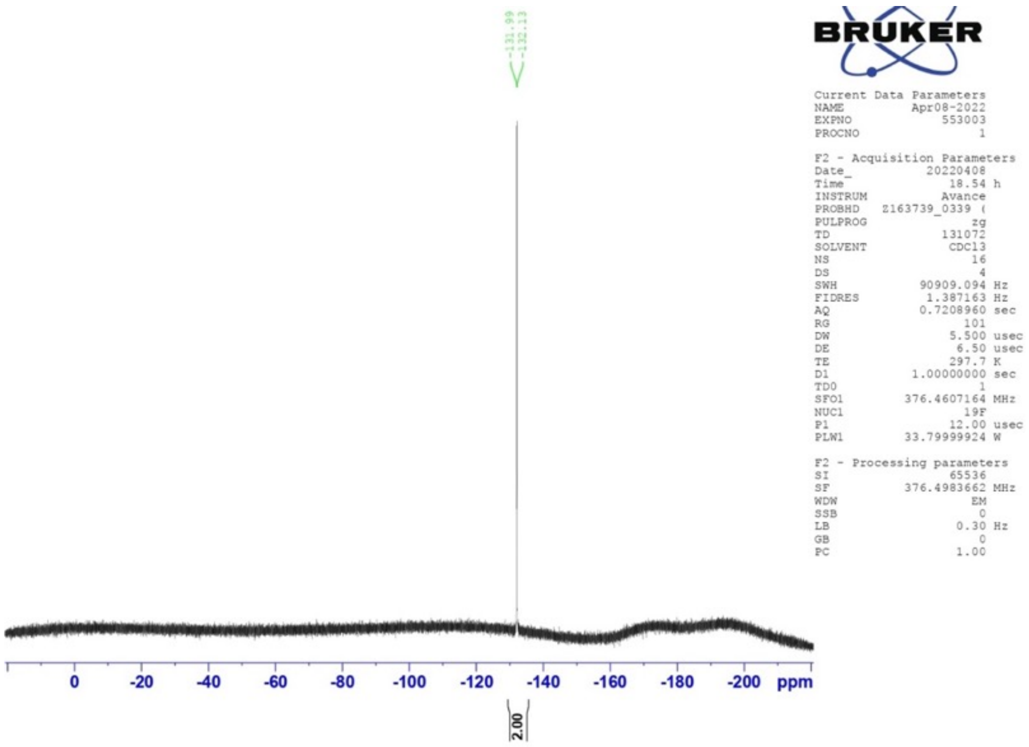

^{19}F NMR (376 MHz, $CDCl_3$)

6-(Difluoromethyl) phenanthridine (3a)

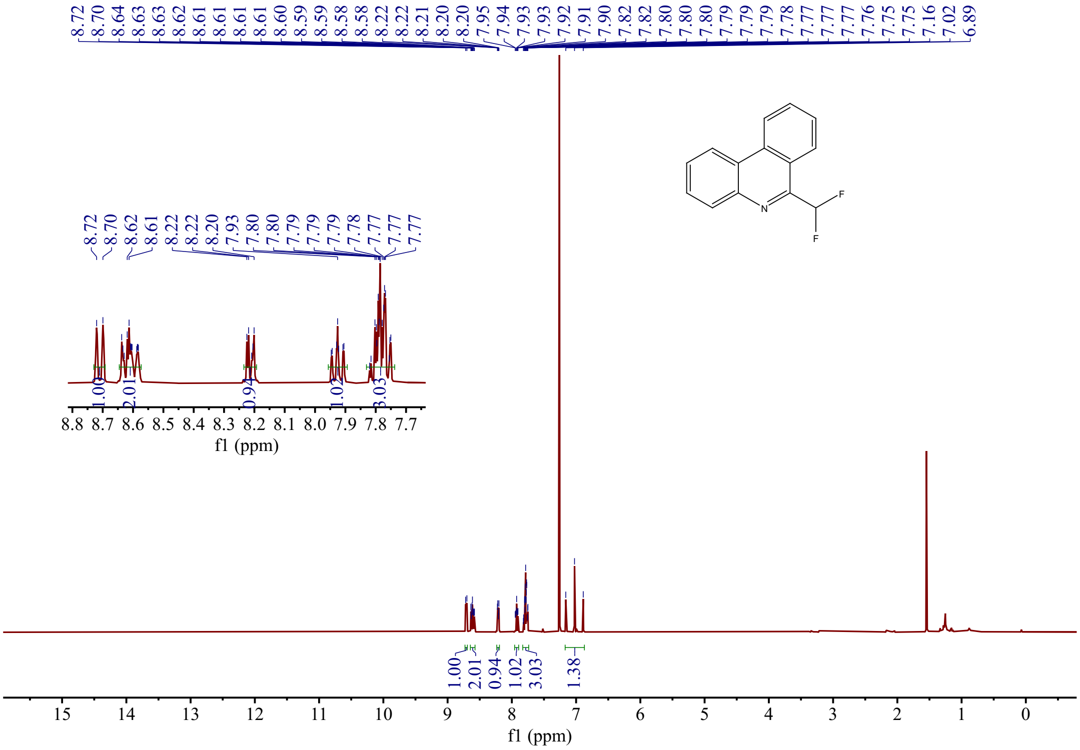

^{1}H NMR (400 MHz, $CDCl_3$)

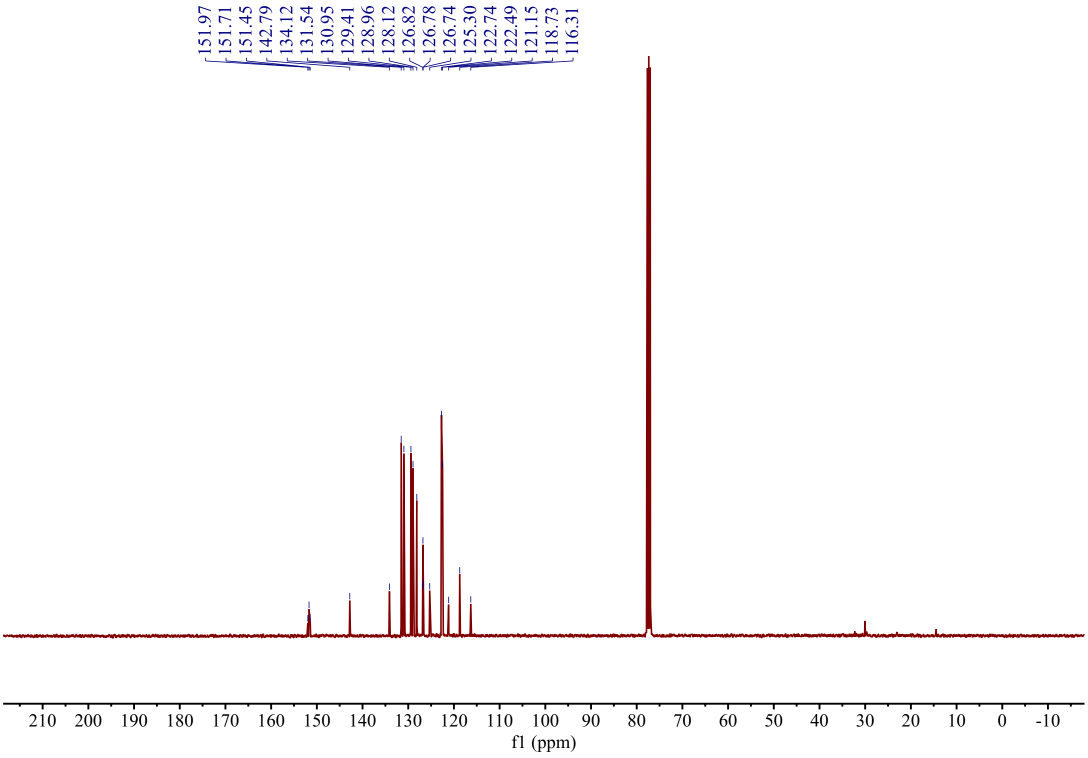

^{13}C{^{1}H} NMR (101 MHz, $CDCl_3$)

6-(Difluoromethyl)-8-methylphenanthridine (3b)

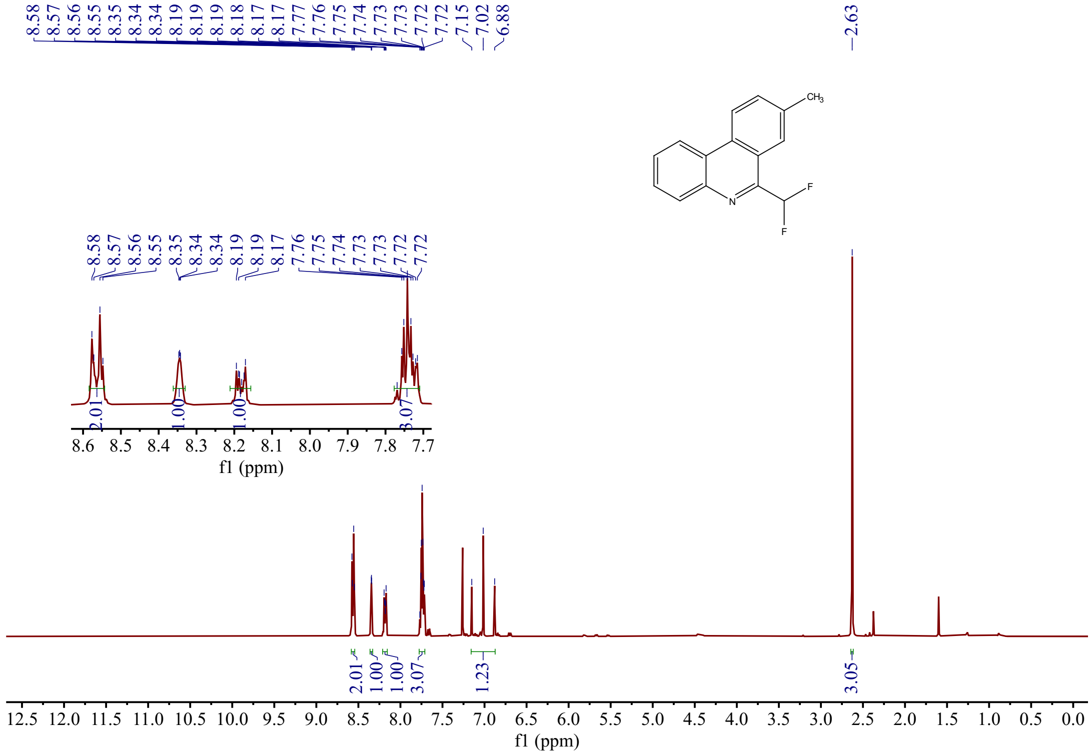

^{1}H NMR ($CDCl_3$, 400 MHz)

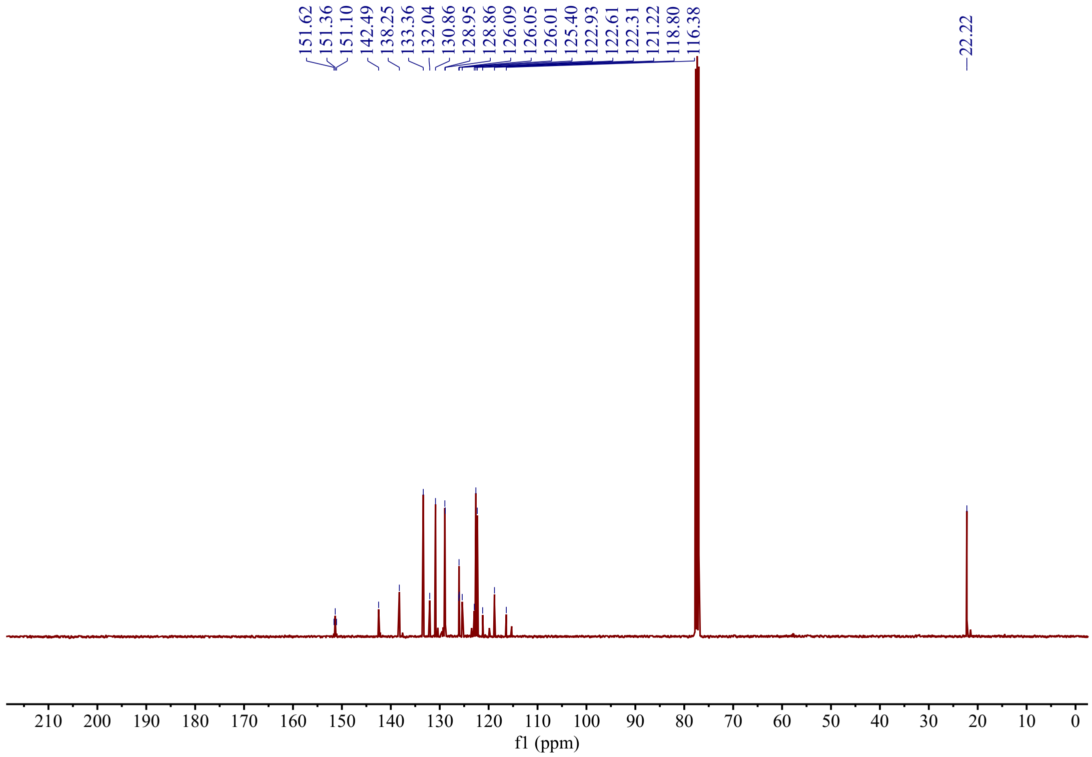

$^{13}C\{^{1}H\}$ NMR ($CDCl_3$, 101 MHz)

8-(*tert*-Butyl)-6-(difluoromethyl)phenanthridine (3c)

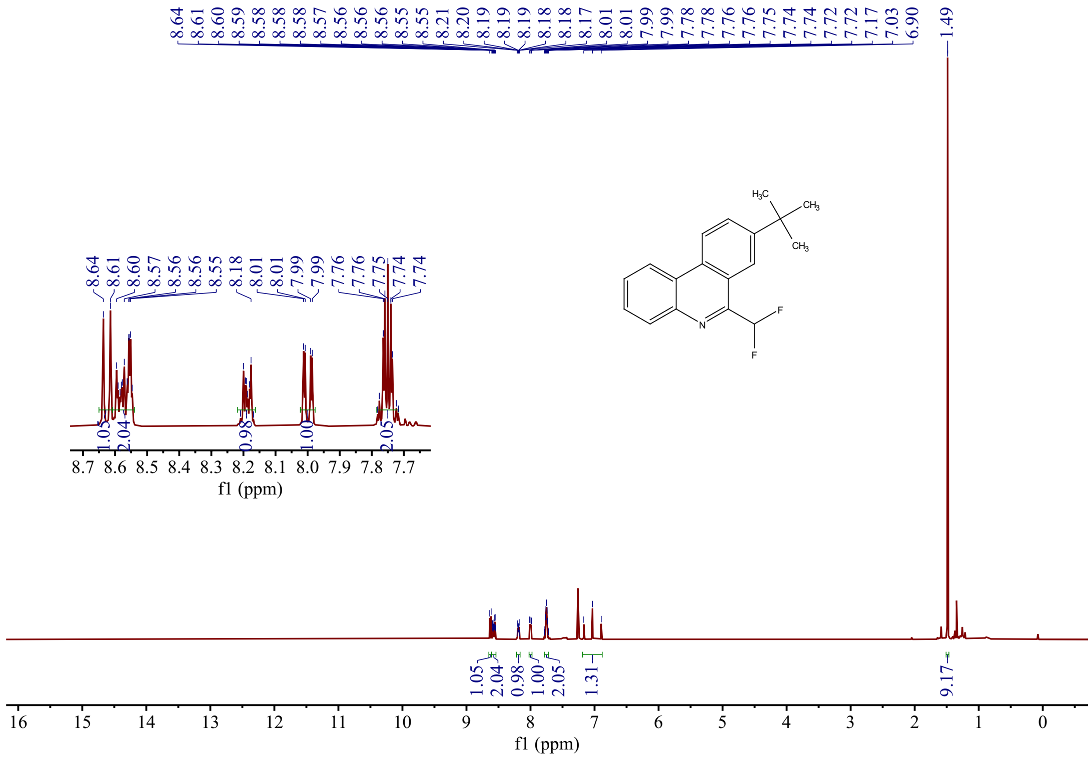

^{1}H NMR ($CDCl_3$, 400 MHz)

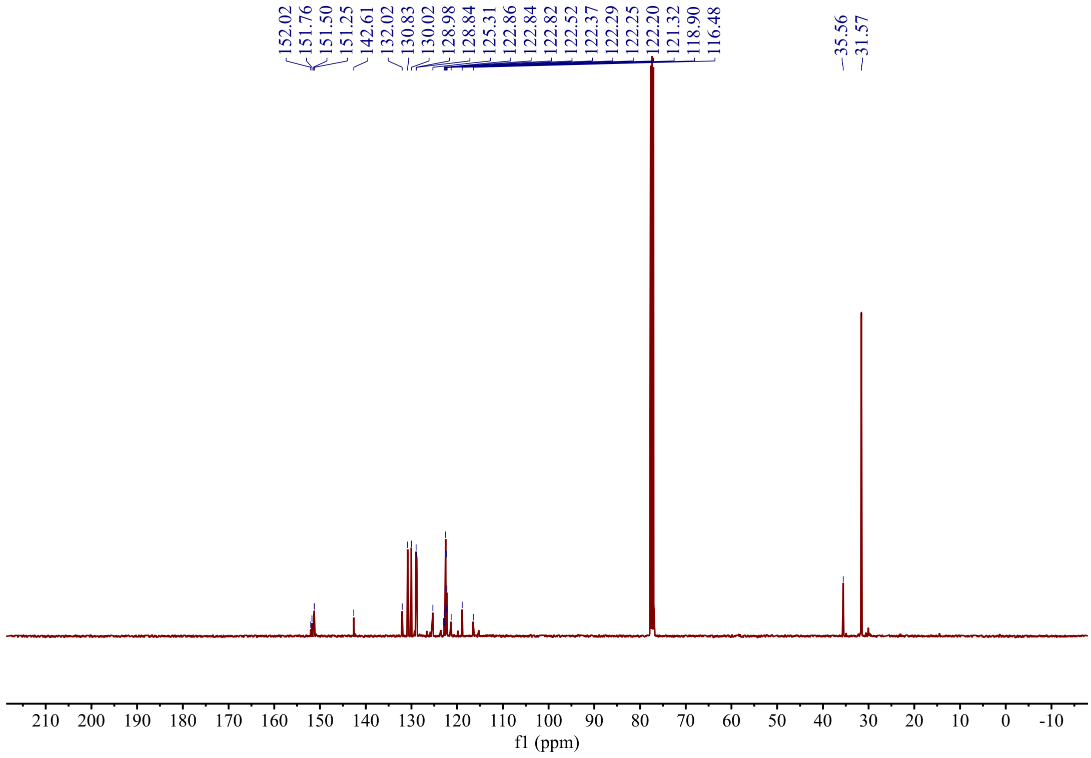

$^{13}C\{^{1}H\}$ NMR ($CDCl_3$, 101 MHz)

6-(Difluoromethyl)-8-methoxyphenanthridine (3d)

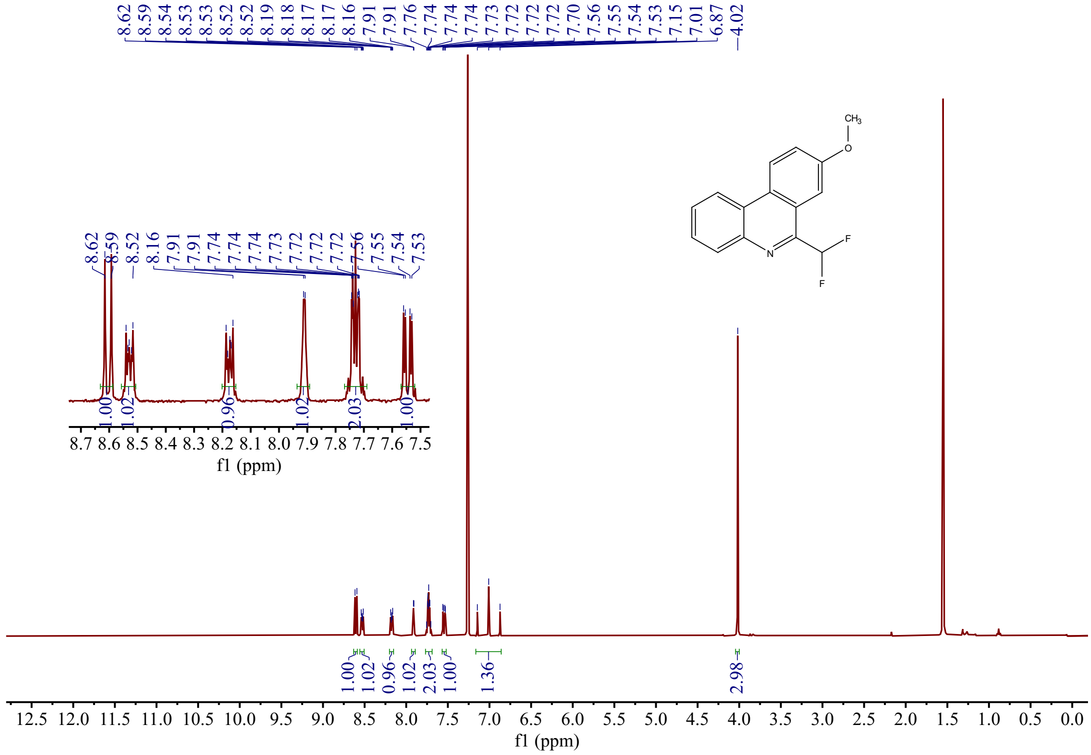

^{1}H NMR ($CDCl_3$, 400 MHz)

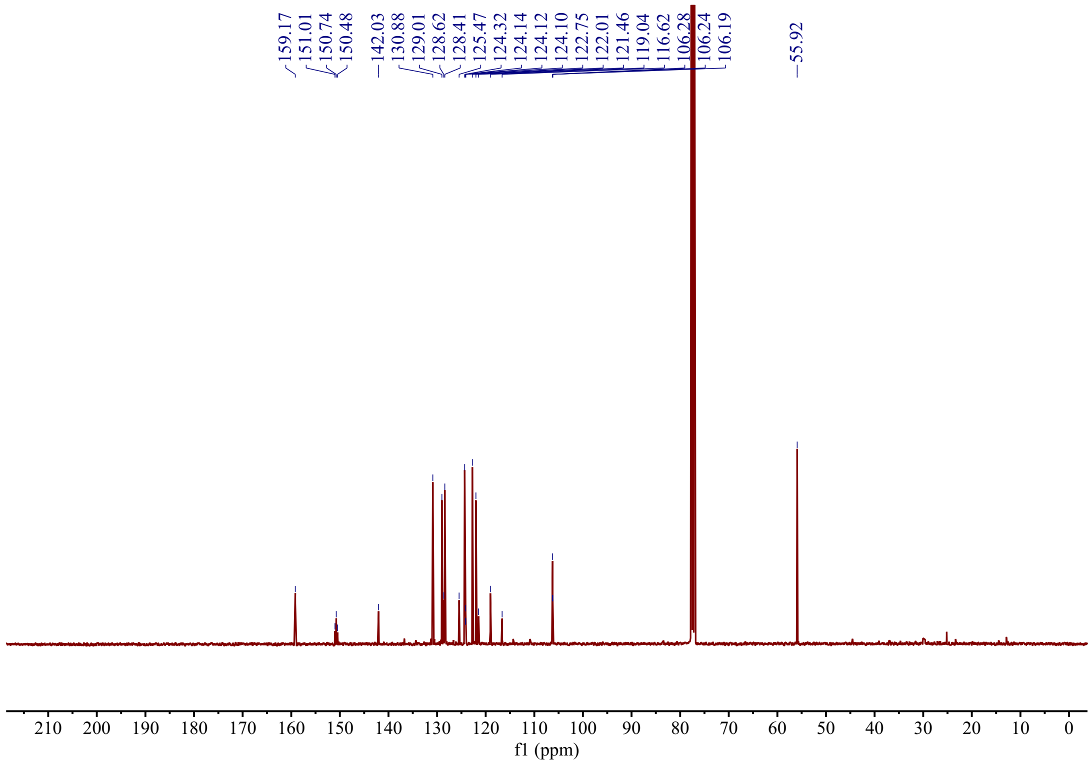

$^{13}C\{^{1}H\}$ NMR ($CDCl_3$, 101 MHz)

6-(Difluoromethyl)-N,N-dimethylphenanthridin-8-amine (3e)

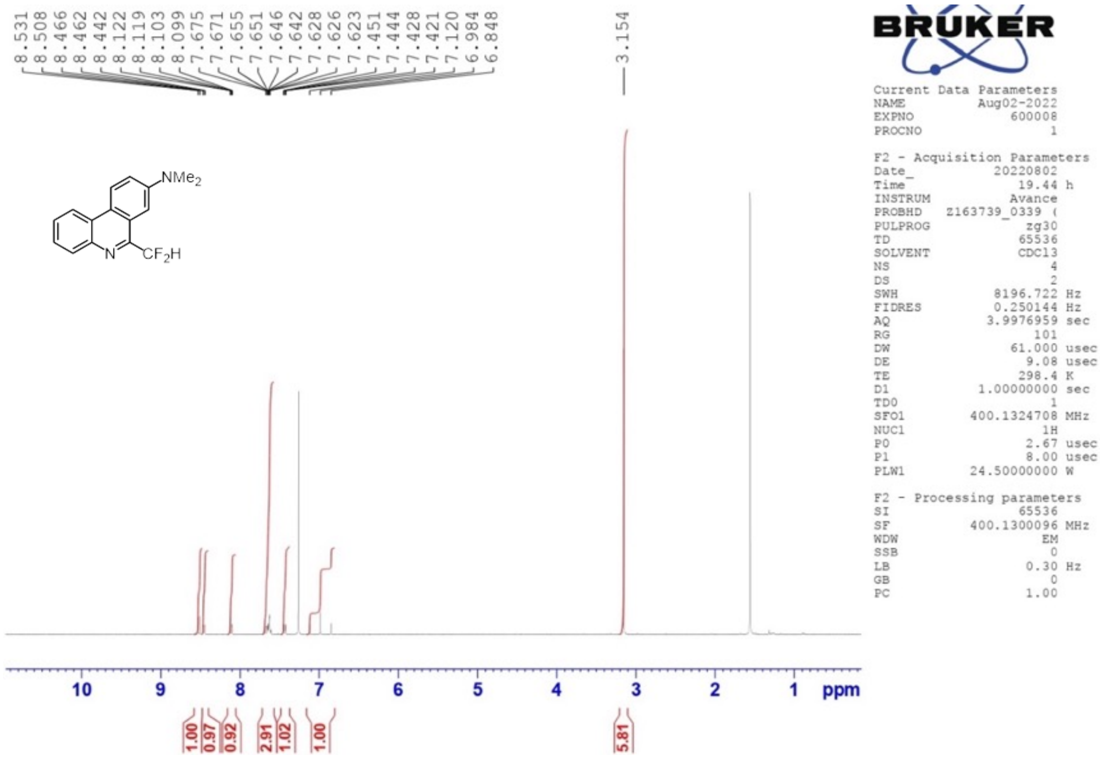

^{1}H NMR (400 MHz, $CDCl_3$)

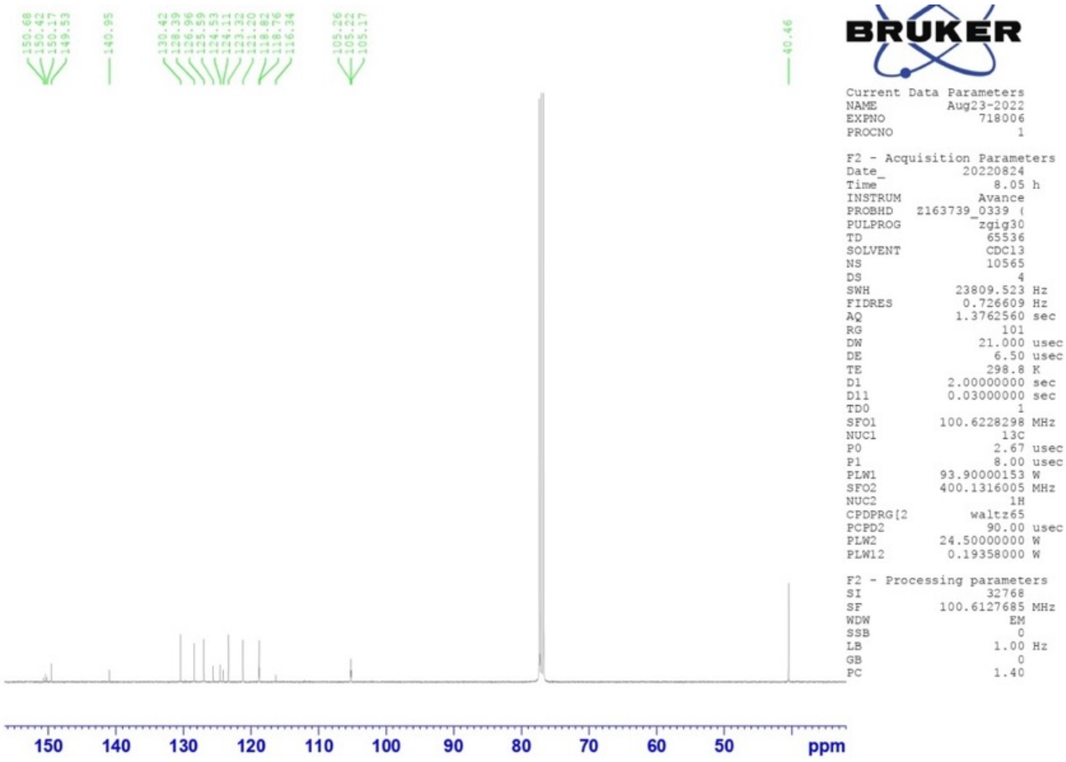

$^{13}C\{^1H\}$ NMR (101 MHz, $CDCl_3$)

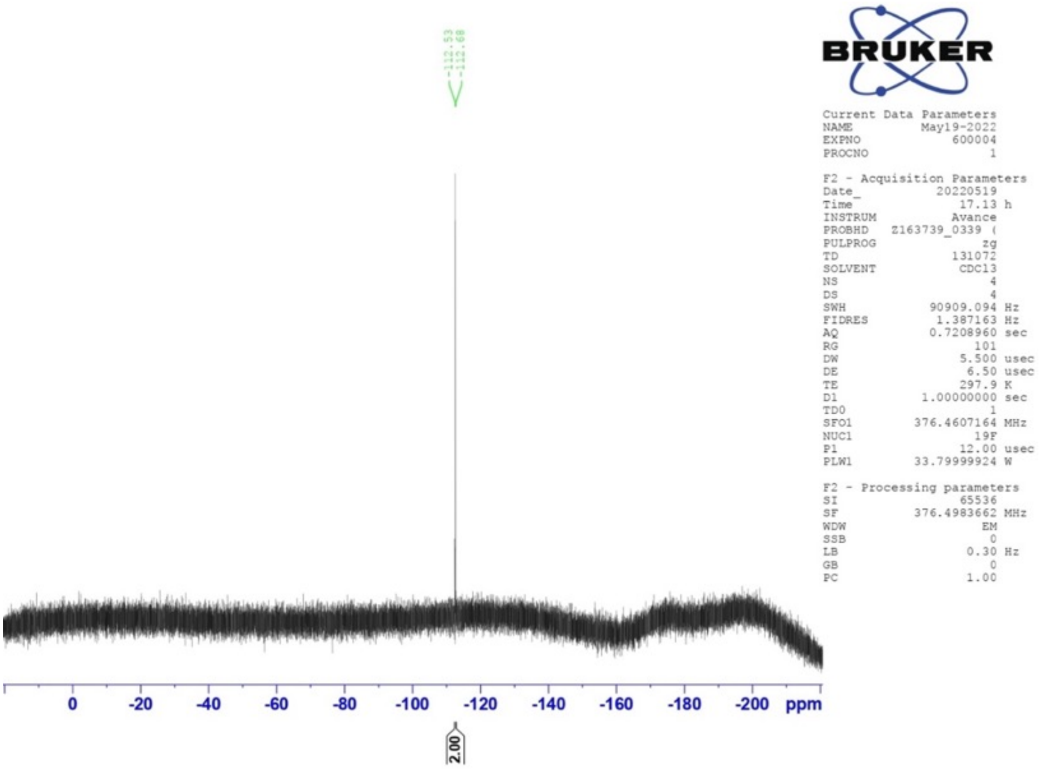

^{19}F NMR (376 MHz, $CDCl_3$)

8-Chloro-6-(difluoromethyl)phenanthridine (3f)

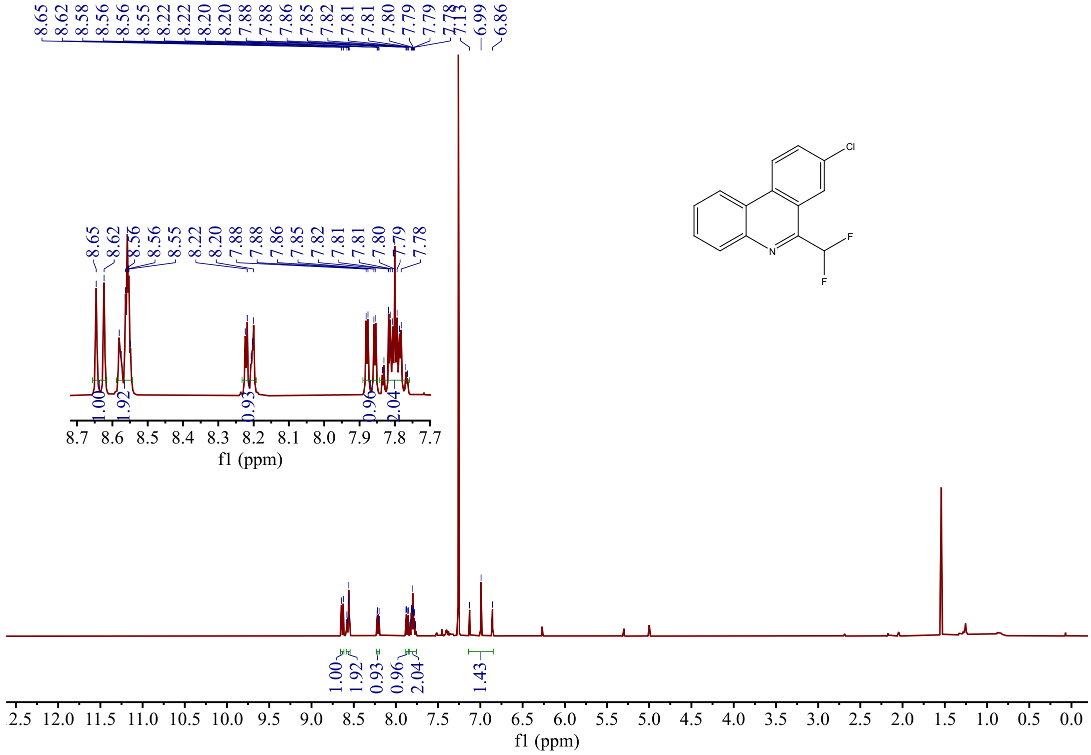

^{1}H NMR ($CDCl_3$, 400 MHz)

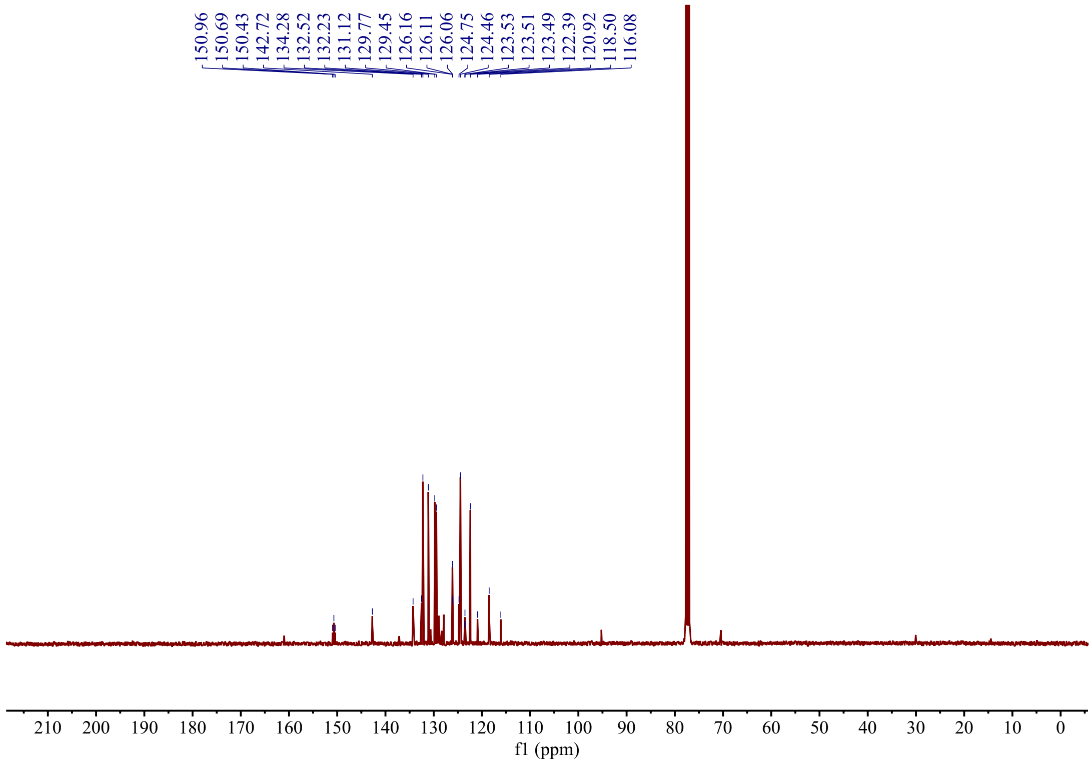

$^{13}C\{^{1}H\}$ NMR ($CDCl_3$, 101 MHz)

6-(Difluoromethyl)-8-(trifluoromethyl)phenanthridine (3g)

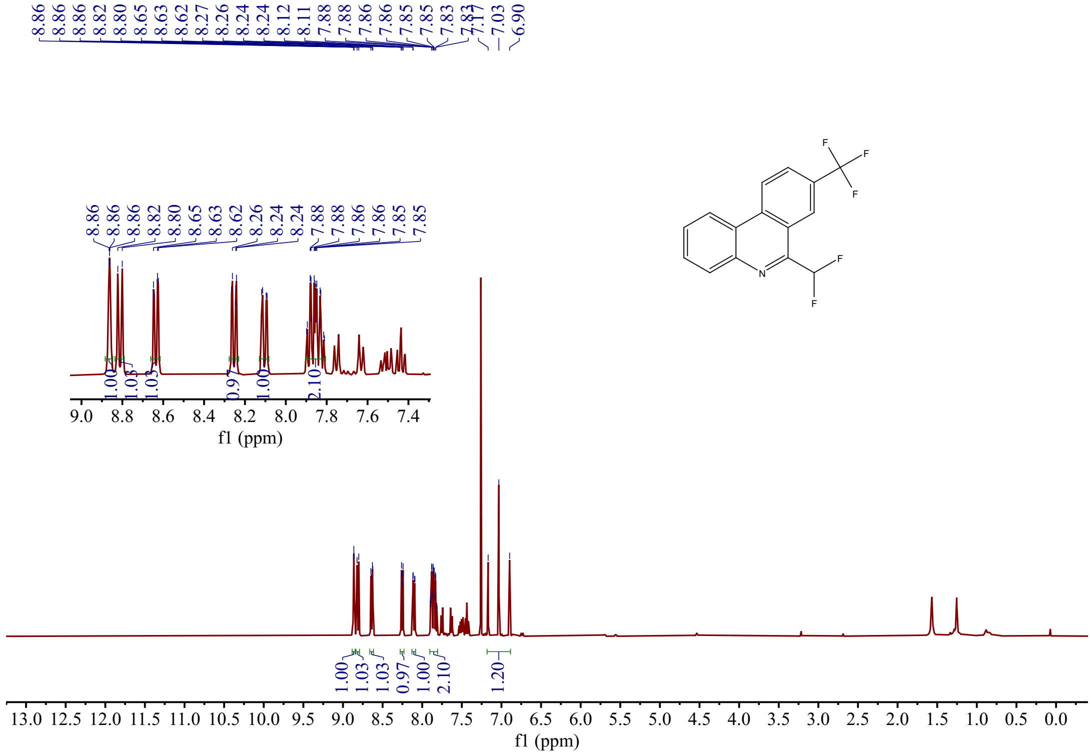

^{1}H NMR ($CDCl_3$, 400 MHz)

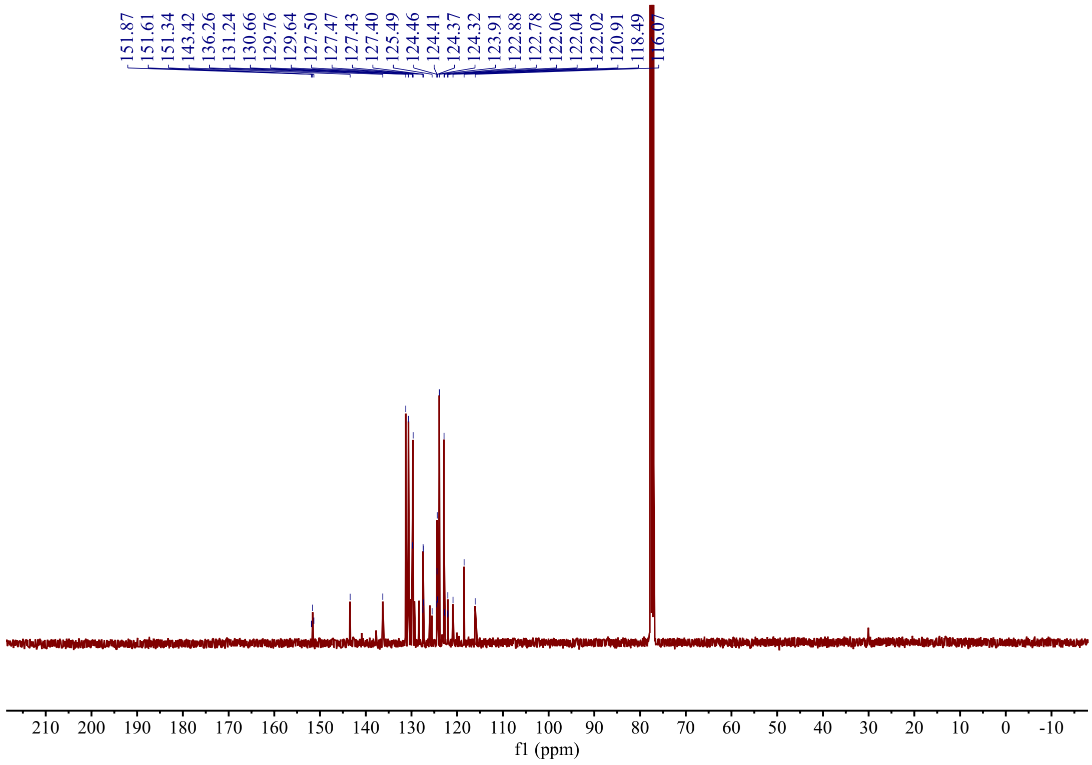

$^{13}C\{^{1}H\}$ NMR ($CDCl_3$, 101 MHz)

6-(Difluoromethyl)-7,9-bis(trifluoromethyl)phenanthridine (3h)

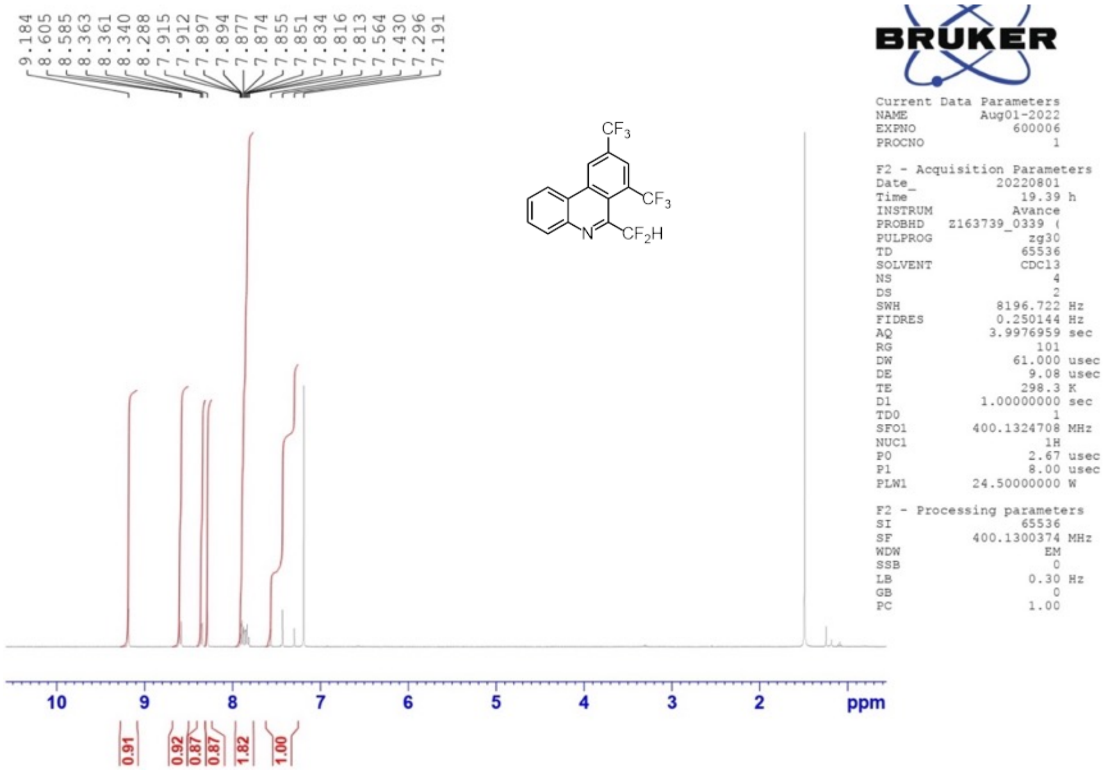

^{1}H NMR (400 MHz, $CDCl_3$)

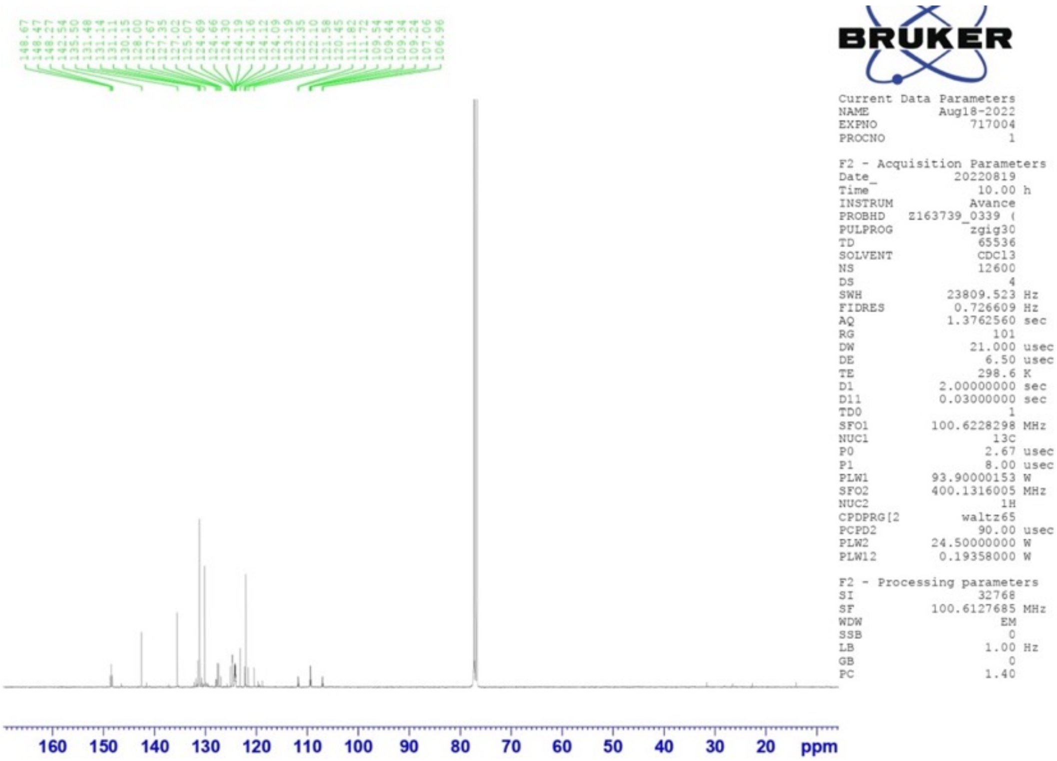

$^{13}C\{^{1}H\}$ NMR (101 MHz, $CDCl_3$)

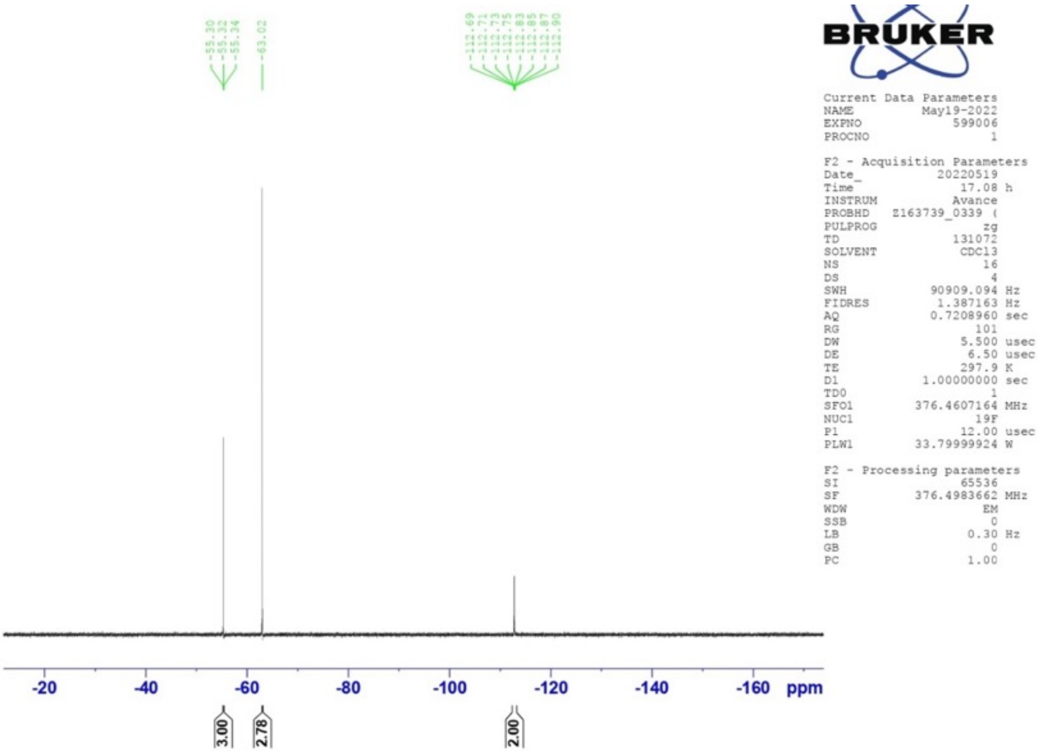

^{19}F NMR (376 MHz, $CDCl_3$)

6-(Difluoromethyl)-3-methylphenanthridine (3i)

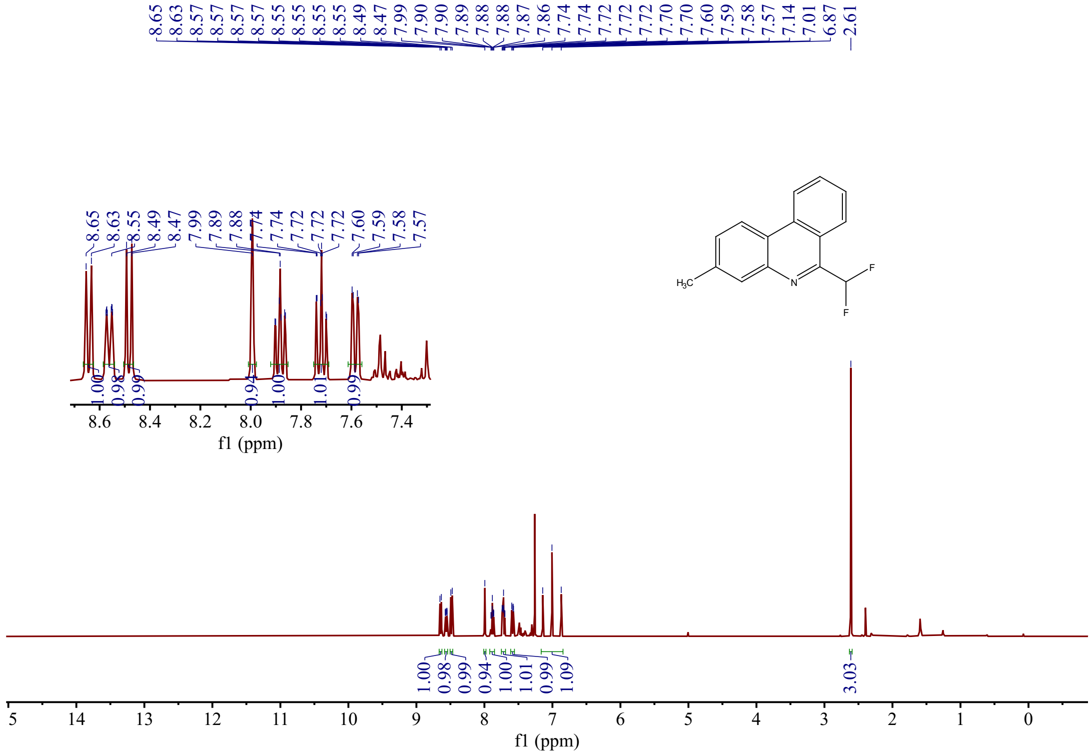

^{1}H NMR ($CDCl_3$, 400 MHz)

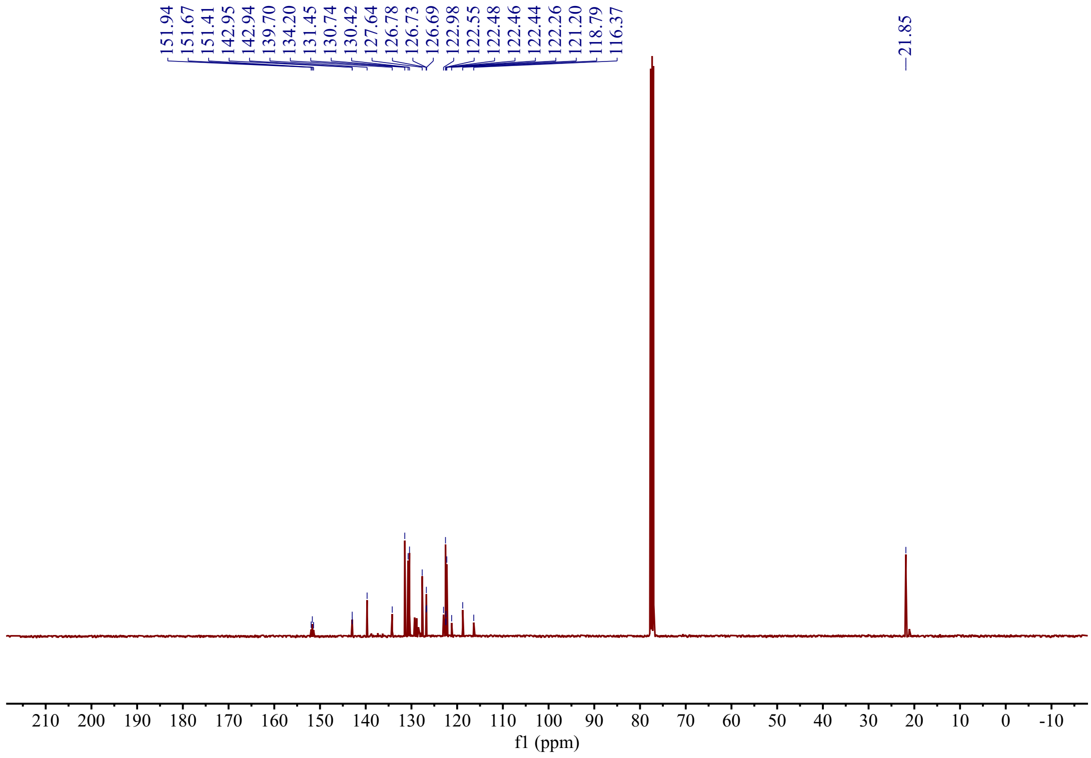

^{13}C{^{1}H} NMR ($CDCl_3$, 101 MHz)

6-(Difluoromethyl)-3-(trifluoromethyl)phenanthridine (3j)

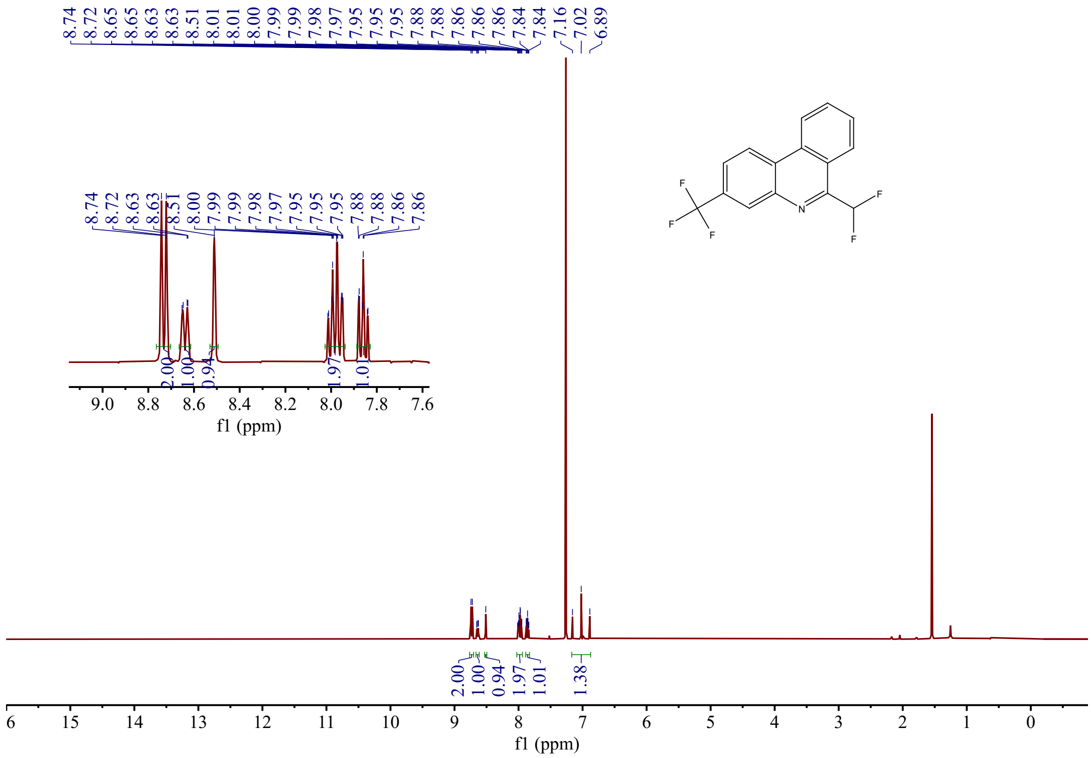

^{1}H NMR ($CDCl_3$, 400 MHz)

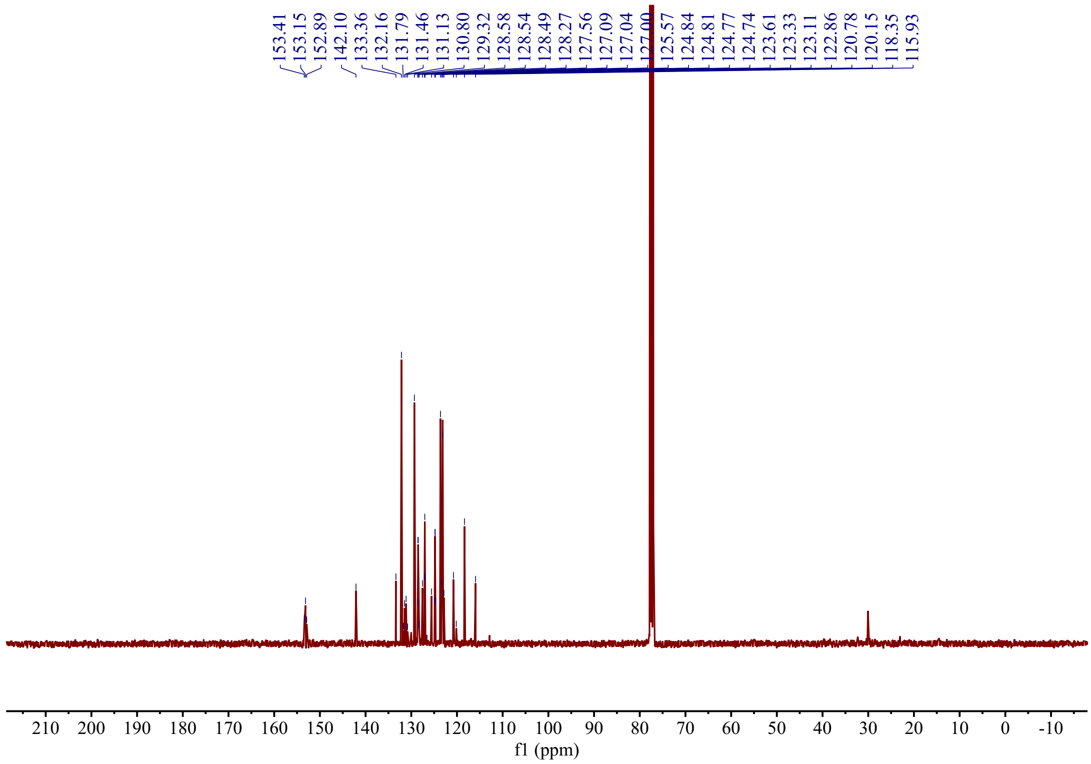

^{13}C{^{1}H} NMR ($CDCl_3$, 101 MHz)

6-(Difluoromethyl)-3,8-dimethylphenanthridine (3k)

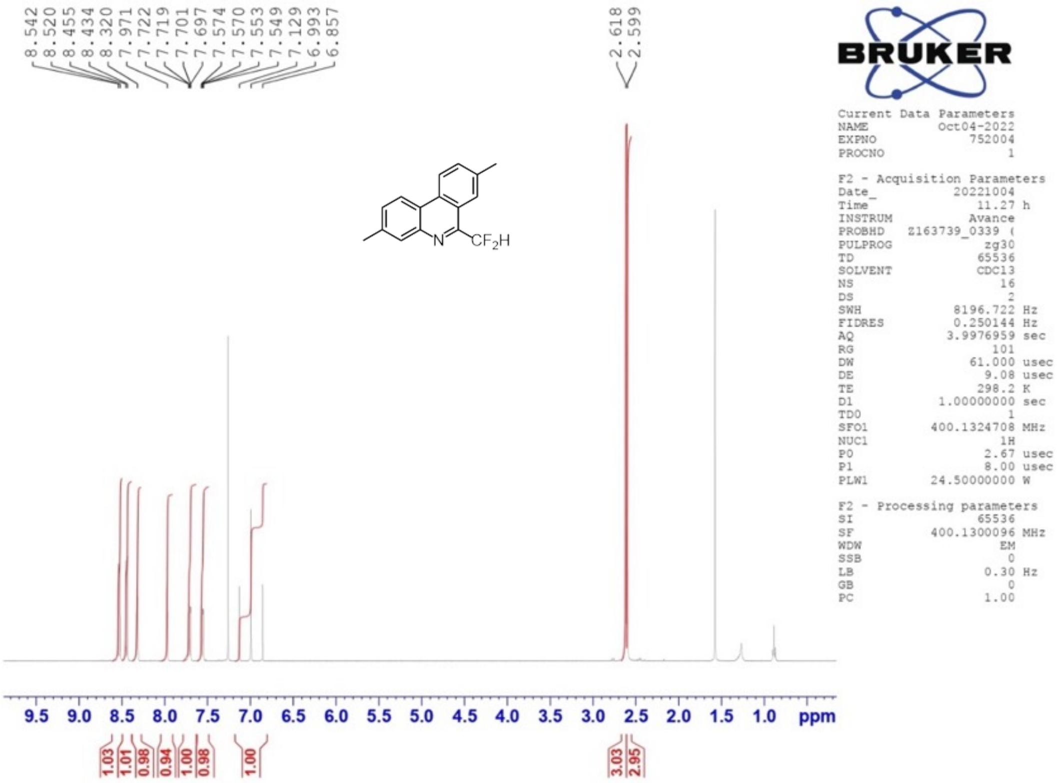

^{1}H NMR (400 MHz, $CDCl_3$)

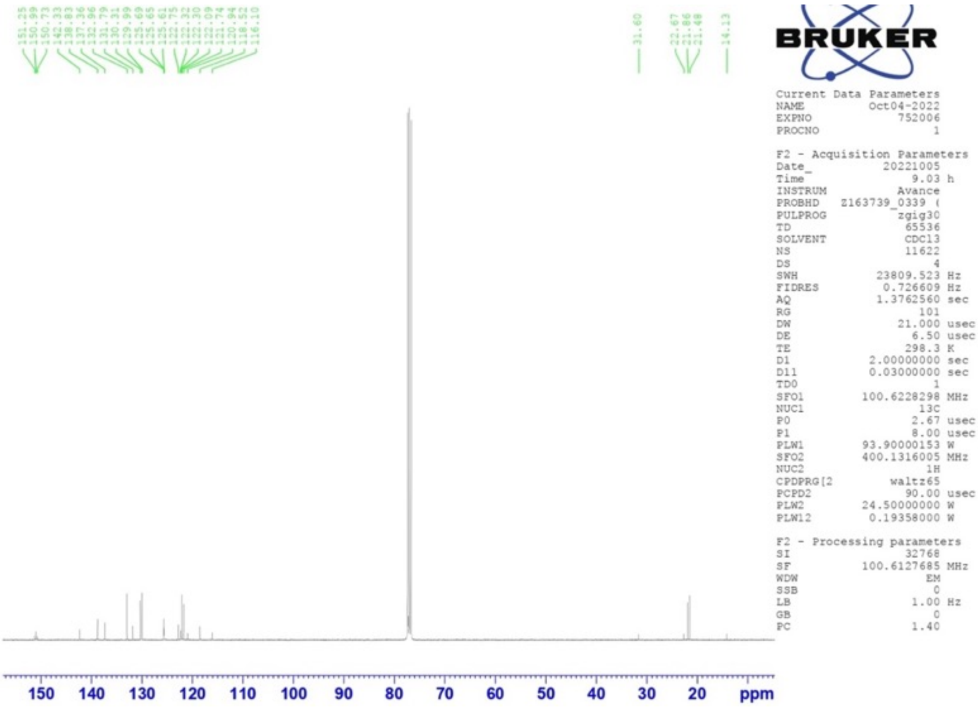

$^{13}C\{^1H\}$ NMR (101 MHz, $CDCl_3$)

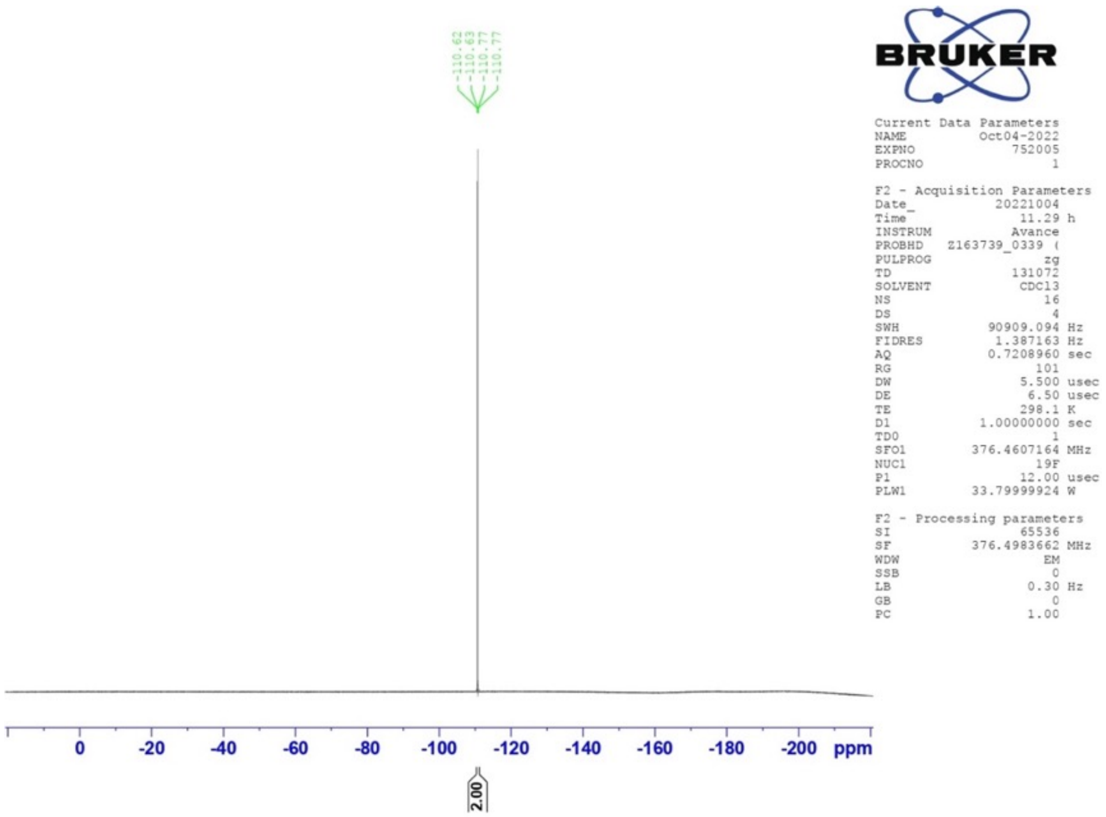

^{19}F NMR (376 MHz, $CDCl_3$)

6-(Difluoromethyl)-3,8-bis(trifluoromethyl)phenanthridine (3l)

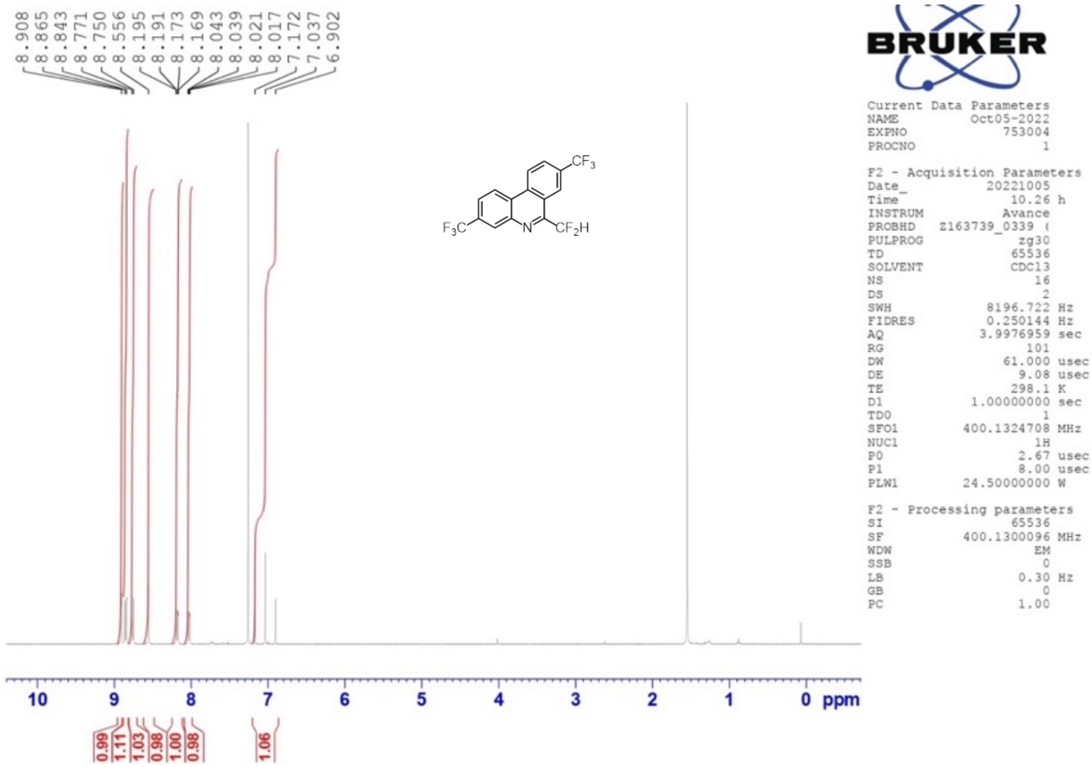

^1H NMR (400 MHz, $CDCl_3$)

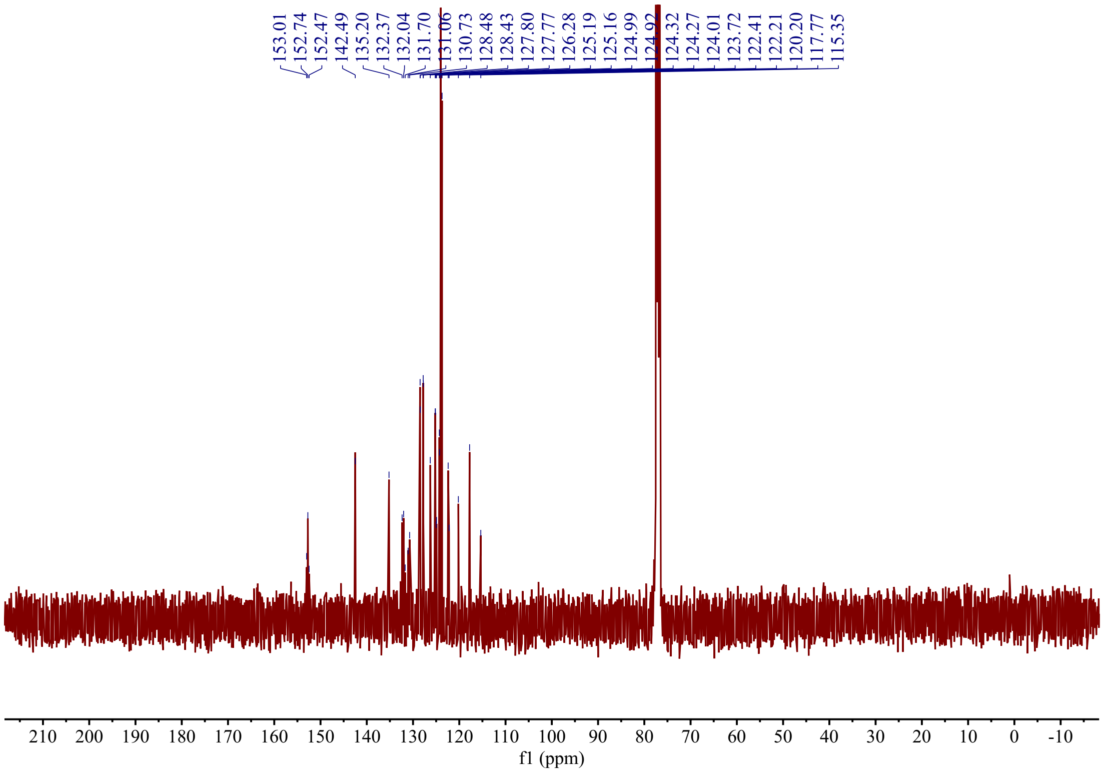

$^{13}C\{^1H\}$ NMR (101 MHz, $CDCl_3$)

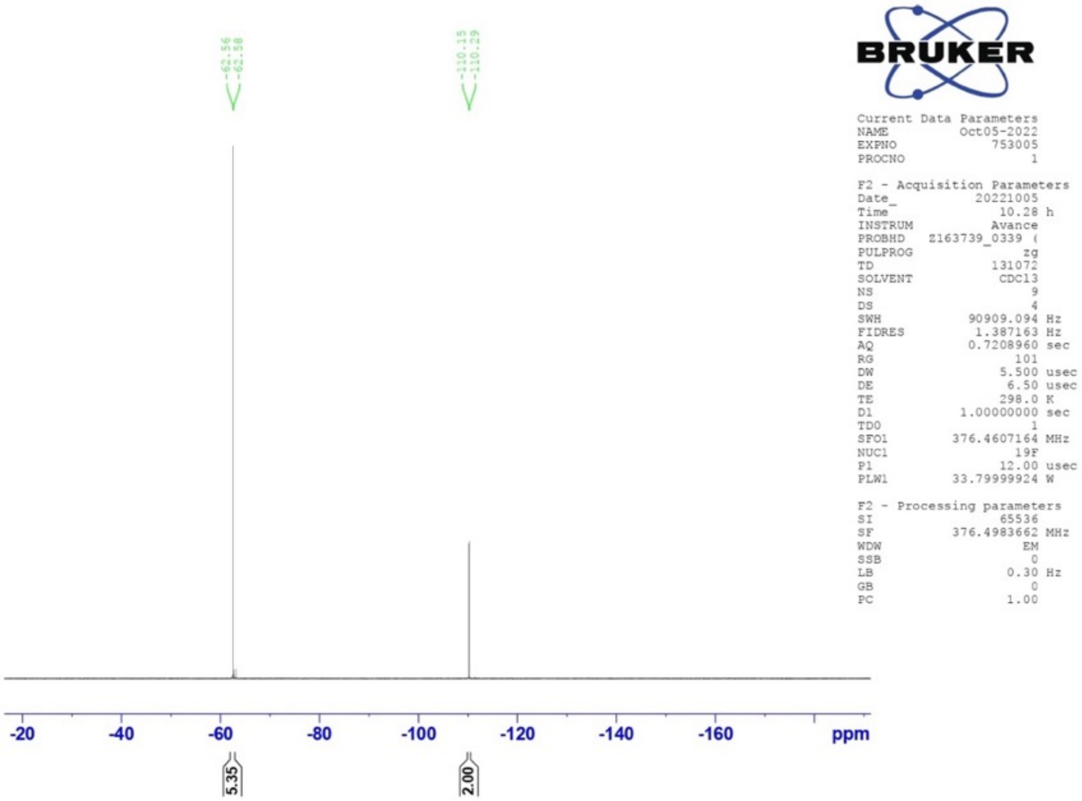

^{19}F NMR (376 MHz, $CDCl_3$)
